# Genomic exploration of the endangered oriental stork, *Ciconia boyciana*, sheds light on migration adaptation and future conservation

**DOI:** 10.1093/gigascience/giae081

**Published:** 2024-10-22

**Authors:** Shangchen Yang, Yan Liu, Xiaoqing Zhao, Jin Chen, Haimeng Li, Hongrui Liang, Jiale Fan, Mengchao Zhou, Shiqing Wang, Xiaotian Zhang, Minhui Shi, Lei Han, Mingyuan Yu, Yaxian Lu, Boyang Liu, Yu Xu, Tianming Lan, Zhijun Hou

**Affiliations:** College of Wildlife and Protected Area, Northeast Forestry University, Harbin 150040, China; College of Life Sciences, Zhejiang University, Hangzhou 310058, China; Center for Biological Disaster Prevention and Control, National Forestry and Grassland Administration, Shenyang 110034, China; Inner Mongolia Academy of Agricultural & Animal Husbandry Sciences, Hohhot 010031, Inner Mongolia, China; Key Laboratory of Black Soil Protection and Utilization (Hohhot), Ministry of Agriculture and Rural Affairs, Hohhot 010031, Inner Mongolia, China; College of Wildlife and Protected Area, Northeast Forestry University, Harbin 150040, China; College of Wildlife and Protected Area, Northeast Forestry University, Harbin 150040, China; Heilongjiang Key Laboratory of Complex Traits and Protein Machines in Organisms, Harbin 150040, China; Center for Biological Disaster Prevention and Control, National Forestry and Grassland Administration, Shenyang 110034, China; College of Wildlife and Protected Area, Northeast Forestry University, Harbin 150040, China; College of Wildlife and Protected Area, Northeast Forestry University, Harbin 150040, China; College of Wildlife and Protected Area, Northeast Forestry University, Harbin 150040, China; Center for Biological Disaster Prevention and Control, National Forestry and Grassland Administration, Shenyang 110034, China; College of Wildlife and Protected Area, Northeast Forestry University, Harbin 150040, China; College of Wildlife and Protected Area, Northeast Forestry University, Harbin 150040, China; Center for Biological Disaster Prevention and Control, National Forestry and Grassland Administration, Shenyang 110034, China; College of Wildlife and Protected Area, Northeast Forestry University, Harbin 150040, China; College of Wildlife and Protected Area, Northeast Forestry University, Harbin 150040, China; Center for Biological Disaster Prevention and Control, National Forestry and Grassland Administration, Shenyang 110034, China; College of Wildlife and Protected Area, Northeast Forestry University, Harbin 150040, China; Heilongjiang Key Laboratory of Complex Traits and Protein Machines in Organisms, Harbin 150040, China; College of Wildlife and Protected Area, Northeast Forestry University, Harbin 150040, China

**Keywords:** oriental stork, comparative genomics, conservation genomics, endangered species, migration

## Abstract

**Background:**

The oriental stork, *Ciconia boyciana*, is an endangered migratory bird listed on the International Union for Conservation of Nature’s Red List. The bird population has experienced a rapid decline in the past decades, with nest locations and stop-over sites largely degraded due to human–bird conflicts. Multipronged conservation efforts are required to secure the future of oriental storks. We propose that a thorough understanding of the genome-wide genetic background of this threatened bird species is critical to make future conservation strategies.

**Findings:**

In this study, the first chromosome-scale reference genome was presented for the oriental stork with high quality, contiguity, and accuracy. The assembled genome size was 1.24 Gb with a scaffold N50 of 103 Mb, and 1.23 Gb contigs (99.32%) were anchored to 35 chromosomes. Population genomic analysis did not show a genetic structure in the wild population. Genome-wide genetic diversity (π = 0.0012) of the oriental stork was at a moderate to high level among threatened bird species, and the inbreeding risk was also not significant (F_ROH_ = 5.56% ± 5.30%). Reconstruction of demographic history indicated a rapid recent population decline likely driven by human activities. Genes that were under positive selection associated with the migratory trait were identified in relation to the long-term potentiation, photoreceptor cell organization, circadian rhythm, muscle development, and energy metabolism, indicating the essential interplay between genetic and ecological adaptation.

**Conclusions:**

Our study presents the first chromosome-scale genome assembly of the oriental stork and provides a genomic basis for understanding a genetic background of the oriental stork, the population’s extinction risks, and the migratory characteristics, which will facilitate the decision of future conservation plans for this species.

## Introduction

Ecosystem degradation and biodiversity decline occur throughout the Anthropocene and have accelerated in the recent decades [1]. Human activities have led to habitat loss, speeding up the pace of the sixth mass extinction. More than 32% of extant species (∼44,000) are threatened with extinction [2]. This global crisis in turn poses a threat to human well-being and calls for more conservation efforts to stop and reverse the current situation.

**Figure 1:**
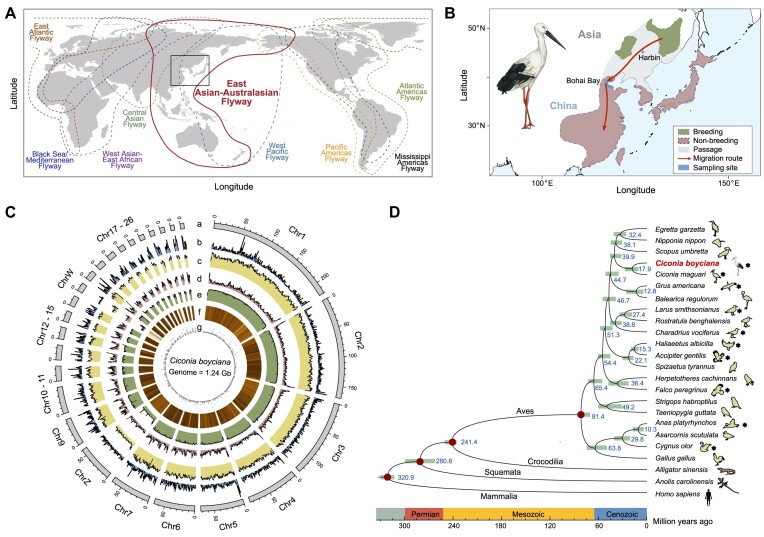
Landscape of the genome assembly and phylogenetic placement of the oriental stork. (A) Nine major flyways across the globe [6]. Black box indicates the range of the oriental storks. (B) Breeding and wintering regions, migratory route, and sampling site (Bohai Bay) of the wild oriental storks used in this study. (C) Genomic features of the oriental stork. (a) The 26 chromosomes larger than 5 Mb. (b) Gene count. (c) Depth of Hi-C reads. (d) GC content density. (e) Depth of WGS reads. (f) Repeat number. (g) Depth of RNA reads. The statistics were calculated using a 500-kbp window. (D) Phylogenetic relationship of the 24 species and the estimated divergence time. Asterisks represented full migrants while others are not migrants.

Birds are effective indicators of the biodiversity condition on earth [3]. Long-term records from BirdLife International have raised significant concern for the world’s birds: populations of 49% bird species (5,412) are declining, including both endangered and unendangered birds, and for many species, the risk of extinction is escalating. Nearly 45% of Important Bird and Biodiversity Areas (IBAs) are identified to be in danger due to pervasive and unsustainable human activities, such as agricultural expansion, logging, and hunting [4–6]. In particular, wetlands along the East Asian–Australasian Flyway (EAAF) (Fig. 1A) are being heavily destroyed, leading to the loss of key wintering sites and bird mortality [7, 8]. The EAAF flyway is used by 492 migratory bird species, and more than 50 million individual birds use the flyway during their annual flight from Arctic Russia and Alaska to Australia and New Zealand [9, 10]. Intensification of human–bird conflicts in Asia has led to a massive decline in the numbers of many migratory waterbirds, including the endangered oriental stork, *Ciconia boyciana* (NCBI:txid52775) [11]; black-faced spoonbill, *Platalea minor* [12]; and the vulnerable white-naped crane, *Antigone vipio* [13]. Migratory birds are wildlife without habitat boundaries and serve as a powerful safeguard for ecosystems. The decline of bird species in the EAAF reduces the energy connectivity and mobility between Arctic nutrient-poor terrestrial system and the southern coastline ecosystem. Although governments have taken actions to protect coastal wetlands and migratory birds, there are still gaps [14]. For example, for most of these threatened migratory birds, the population-level and genome-wide genetic data are lacking, and thus it is difficult to assess their genome-wide genetic background, bringing obstacles to design scientific recovery actions.

The oriental stork is a large wetland shorebird in the EAAF. It has been listed as “Endangered” on the International Union for Conservation of Nature (IUCN) Red List since 1994. The wild bird population comprises a single population with an estimated population size of c. 3,000 individuals (Eastern Asia population) [15]. They are full migrants, breeding in southeastern Siberia, mainly along the Russia–China border, and migrating annually to Bohai Bay (1,500 km) and Poyang Lake (2,600 km) in autumn (Fig. 1B) [16]. Oriental storks were once widely distributed across Northeast Asia, but the wild population dramatically declined in 1868–1935 [17]. In 1970s, wild storks in Japan and Korea disappeared, with the remaining individuals breeding in more constricted areas in the Russian Far East and China. In 1960s, there were more than 1,000 oriental storks breeding in the Heilongjiang Province. However, their numbers decreased to 123 in 1986 and were fewer than 50 in 1990 [18, 19]. Habitat loss is considered the major reason for their population decline. Deforestation, agricultural development, and spring fires have severely destroyed their nest trees in Russia. Reclamation of wetlands and overfishing in the stop-over and wintering sites in China have led to a decreased refueling rate and an increased mortality rate, particularly for juveniles [15, 20].

In this decade, genomic technology has become a promising tool in the field of conservation, and conservation genetics is in transition to conservation genomics with the rapid development of the sequencing technology and plummeting sequencing costs [21–24]. Investigating genetic backgrounds of endangered species by genomic approaches can inform conservation efforts [21–24]. The Iberian lynx (*Lynx pardinus*) is one example of an endangered species that benefited from conservation genomics. Developing a high-quality reference genome and population genomic studies have generated a high-quality variation map and a catalog of deleterious mutations for the Iberian lynx, which are now used for evaluating population fitness and monitoring the genetic diversity of the reintroduced population [25–28]. Other examples include conservation genomics studies of the Florida panther [29], the Indian tiger [30], and the Kākāpō [31]. However, population genetic studies of the oriental stork have been largely underexplored for genome-wide investigation, including their genetic diversity, inbreeding level, and the genomic basis for the adaptation of migration traits.

In this study, the first chromosome-level genome assembly was presented for the oriental stork. The genomes of 29 wild and 15 captive birds were resequenced for extensively exploring the genetic characteristics of this endangered bird. The genetic background of these birds was then systematically investigated to measure possible genome-wide extinction risks. Genomic signatures of evolution and adaptation for the migratory-related characteristics were scanned across the genome and related to population viability.

## Methods

### Samples and ethics statement

Blood samples from 16 captive-born and 3 wild-rescued oriental storks were collected at Harbin North Forest Zoo, Harbin, Heilongjiang, China. The blood sample from a captive-born oriental stork (sample ID: N1170) was used for reference genome assembly. An additional 26 wild-rescued oriental storks were collected around the Bohai Bay, China, and the muscle samples from these individuals were collected after their natural death. Research and blood/tissue collection were approved by the Institutional Review Board of Northeast Forest University (No. 2024WPE05). We also downloaded whole-genome sequencing data generated from 2 bird individuals: 1 from Kanagawa, Japan (*n* = 1) and another from the San Diego Zoo, the United States (*n* = 1). Our final dataset consisted of 46 samples, including 26 wild birds from Bohai Bay, 18 from Harbin North Forest Zoo (3 wild and 15 captive birds), 1 captive bird from Japan, and 1 captive bird from the United States.

### Nucleic acid extraction, library construction, and sequencing

For ONT long-read sequencing, high molecular weight genomic DNA was extracted using the DNeasy Blood and Tissue kit (Qiagen), and 8 to 10 μg DNA was size-selected (>50 kb) according to the manufacturer’s instructions for the ONT library preparation. Then, 800 ng of library DNA was used for sequencing on the PromethION sequencer (Oxford Nanopore Technologies). For Hi-C sequencing, a cross-link process with formaldehyde was first conducted using the blood sample, and then the Hi-C library was constructed following the protocol of Lieberman-Aiden et al. [32]. Total RNA was extracted using TRlzol reagent (Invitrogen). We then used the Agilent 2100 Bioanalyzer system (Agilent) and Qubit 3.0 (Life Technologies) to evaluate the quality and quantity of the extracted RNA. DNA libraries with short insert sizes were prepared according to the manufacturer’s instructions on the MGI platform. These libraries were finally sequenced on the DNBSEQ-T1 sequencer for 100-bp paired-end reads.

### *De novo* assembly, annotation, and assessment

The genome size of the oriental stork was first estimated by the *k*-mer frequency method based on whole-genome sequencing (WGS) data of the assembled individual [33]. The genome was then assembled as per the following steps: (i) *de novo* assembly was conducted using ONT long reads by NextDenovo (v2.5.0) (RRID:SCR_025033). Two core modules were utilized to generate a primary assembly: the NextCorrect module was used to correct raw ONT long reads and extract the consensus sequences. NextGraph module was used for the preliminary assembly. A read cutoff of 1 kb was set and other default parameters were maintained in NextDenovo. (ii) Contigs were polished using NextPolish (v1.4.0) (RRID:SCR_025232) [34] with ONT long reads. (iii) Hi-C reads were mapped to the genome using a Burrows–Wheeler aligner *mem* (BWA, v0.7.17) [35] algorithm with default parameters. A 3-dimensional (3D)–DNA pipeline (v180,922) (RRID:SCR_017227) was applied to generate a chromosome-level genome assembly. (iv) WGS reads were remapped to the assembly [36] to correct mis-sequenced bases introduced by long-read sequencing. (v) BUSCO (RRID:SCR_015008) analysis [37] was performed to evaluate the completeness of our assembly using aves_odb10 database. (vi) WGS, Hi-C, and RNA sequencing (RNA-seq) data were mapped to the final genome to check mapping rate, base coverage, and sequencing depth by the BWA *mem* algorithm with default parameters.

*De novo* and homology-based methods were combined to identify repetitive elements in the genome assembly. First, *de novo* predictions were performed using LTR finder (v1.0.6) (RRID:SCR_015247) [38], MITE-hunter (v4.07) [39], and RepeatModeler2 (v2.0.1) [40] software with default parameters. The results were merged into RepBase as known repeats. Next, RepeatMasker (v4.0.5) (RRID:SCR_012954) [41] was used to identify and classify transposable elements by searching the RepBase library [42]. Tandem repeats were identified using Tandem Repeats Finder (TRF, v4.09) [43].

All repetitive elements were masked across the genome for annotation of the protein-coding genes. A combination of *de novo*, homology-based and transcript mapping methods was used to conduct gene annotation. *De novo* predictions were carried out using SNAP (v1.0) (RRID:SCR_002127) [44], glimmerHMM (v3.0.3) (RRID:SCR_002654) [45], and AUGUSTUS (v2.5.5) (RRID:SCR_008417) [46] software. We used Trimmomatic (v0.27) (RRID:SCR_011848) [47] to filter the RNA data and assembled the data using Trinity (v2.9.0) (RRID:SCR_013048) [48]. The dataset was then mapped to the reference genome to predict gene structure using the Program to Assemble Spliced Alignments (PASA, v2.2.0) (RRID:SCR_014656) [49]. For homology-based prediction, protein sequences from *Gallus gallus, Anas platyrhynchos, Ciconia maguari, Meleagris gallopavo, Pavo muticus, Taeniopygia guttata*, and *Homo sapiens* were aligned to our genome using Blastall (v2.2.26) [50] with an E-value cutoff of 1e-5. The gene models were confirmed using GeneWise (v2.4.1) [51]. The results obtained from the above 3 approaches were finally combined to generate a comprehensive gene set using Maker (v 3.01.03) [52]. These genes were aligned to the databases of SwissProt, TrEMBL, InterPro, Gene Ontology (GO), and KEGG for functional annotation.

### Identification of sex-linked regions

The 2 sex chromosomes (Z and W chromosomes) were identified by checking the sequencing depth of the individual male and female birds. The syntenic relationships with the sex chromosomes of *G. gallus* (GenBank ID: GCA_016,699,485.1) and *T. guttata* (GenBank ID: GCF_003,957,565.2) were then examined. Alternative splicing of each gene on the chromosomes was filtered for the 3 species. The longest protein sequences of *G. gallus* and *T. guttata* were aligned against our assembled Z and W chromosomes using blastp in BLASTtools (v2.2.26) [53] with the parameter of “-evalue 1e-5.” Synteny blocks were then identified using MCScanX [54] and visualized by Circos (v0.69–9) [55] software.

### Phylogeny reconstruction and divergence time estimation

Protein sequences from 24 species (*H. sapiens, Anolis carolinensis, Alligator sinensis, G. gallus, Cygnus olor, Asarcornis scutulata, A. platyrhynchos, T. guttata, Strigops habroptilus, Falco peregrinus, Herpetotheres cachinnans, Spizaetus tyrannus, Accipiter gentilis, Haliaeetus albicilla, Charadrius vociferus, Rostratula benghalensis, Larus smithsonianus, Balearica regulorum, Grus americana, C. maguari, C. boyciana, Scopus umbrette, Nipponia nippon, Egretta garzetta*) were aligned to identify homologous gene families. The longest protein sequence translated from each gene was selected in this alignment by blastp function in the BLASTtools (v2.2.26) [53] with the parameter of “-evalue 1e-5.” A total of 1,800 shared single-copy genes were used to construct a maximum likelihood phylogenetic tree by IQTREE (v1.6.12) [56]. Divergence time among these species was estimated by MCMCTREE (v4.5) in PAML [57] software with multiple fossil time points used for time calibration [58].

### Variants calling and quality control

Whole-genome sequencing data from individual bird samples used in this study were mapped to our assembled reference genome using the BWA *mem* algorithm with default parameters. Read sorting, reordering, and deduplication were carried out using Picard (v2.1.1) (RRID:SCR_006525). Variant calling was performed using DNAseq Haplotyper in Sentieon (v202010.01) [59]. Bam files and genomic variant call format (gVCF) files were generated for each individual, and joint calling was conducted using GVCFtyper algorithm in the Sentieon DNAseq pipeline to generate a combined VCF file covering all individuals. Variants were filtered using the following procedures: (i) indels and multiallelic variants were removed; (ii) hard filtering was completed with the following parameters: QD < 2.0 || FS > 60.0 || MQ < 40.0 || MQRankSum < −12.5 || ReadPosRankSum < −8.0 –filter-name snp_filter; and (iii) genotype missing rate larger than 10% was removed from the variant set. Single-nucleotide polymorphism (SNP) sites on the Z and W chromosomes were also removed for the downstream population genomic analysis.

### Population structure analysis

The VCF files were converted into PLINK format files with VCFtools (v0.1.16) (RRID:SCR_001235) [60]. Principal component analysis (PCA) was then performed with PLINK (v1.9) [61] software. Inference of ancestral components was conducted with ADMIXTURE (v1.3.0) [62], and *K* value was set from 1 to 5 with the “-cv” flag to calculate the cross-validation (CV) error. A phylogenetic tree was constructed using IQTREE (v1.6.12) with 1,000 bootstraps. The tree layout was visualized using the online tool iTOL (RRID:SCR_018174).

### Genetic diversity and inbreeding

Genome-wide heterozygosity (*H*) of each individual genome was calculated by the VCFtools (v0.1.16). Nucleotide diversity (π) was calculated by a nonoverlapping 5-Mb sliding window along all autosomes using VCFtools (v0.1.16). Runs of homozygosity (ROHs) were identified using PLINK (v1.9) with the following parameters: –homozyg-window-snp 20 –homozyg-kb 100 –homozyg-density 50. Inbreeding coefficient was estimated as the proportion of genome present in the ROH region (F_ROH_). Comparison between wild and captive populations was conducted using a 2-sided pairwise *t* test in R (v 4.1.2).

### Mutational load

The alleles in the *C. maguari* genome, the closest relative of oriental stork, were used to serve as the ancestral state of the oriental stork genome. The reference genome of *C. maguari* (GenBank ID: GCA_013,399,255.1) was transformed to a 100-bp FASTQ file by sliding a nonoverlapping window across the genome, and the short reads were then mapped to our assembled genome using BWA *mem* with the following parameter: -B 3. Only reads uniquely mapped to our genome were kept by the SAMtools/BCFtools (RRID:SCR_005227) (v1.3) [63] view function with “-F 4 -q 20.” Finally, a consensus sequence was generated to represent ancestral alleles on the oriental stork genome using SAMtools mpileup function with depth >1×. A new VCF file containing 6,028,662 derived SNPs was obtained after replacing the reference alleles by a custom Perl script.

SnpEff (v4.3) software was used to annotate the derived SNPs into 3 categories: (i) synonymous mutations, (ii) missense mutations, and (iii) loss of function (LoF) mutations. Here, the “stop_gained,” “splice_donor_variant” and “splice_acceptor_variant,” “start_lost,” “stop_lost,” and “splice_region_variant” were considered LoF mutations. Next, the number of SNPs per individual in the homozygous and heterozygous state was counted, respectively. The proportion of homozygous derived alleles was measured with the following formula: 2 × homozygous sites/(2 × homozygous sites + heterozygous sites) [64]. Derived allele frequency was calculated with 15 randomly selected individuals from the wild and captive bird population to avoid the potential bias from sample size.

### Inference of population demography

Combination of pairwise sequentially Markovian coalescent (PSMC, v0.6.5) [65], SMC++ (v1.13.1) [66], and approximate Bayesian computation (ABC) methods was used to track the population dynamics of the wild population over generations. For the PSMC analysis, the bam file of each individual was converted to a fasta format sequence using the SAMtools mpileup function with the depth setting to ≥1/3 and ≤2 of the average sequencing depth. PSMC software was then run with the following parameters: -N25 -t5 -r5 -p 4+25*2+4+6. For the SMC++ method, 2 individuals from the wild population were randomly selected to generate a mask file of uncovered regions by bamCaller.py. SMC++ was applied based on covered sites to infer population history with the following parameters: –cores 8 –knots 24 –timepoints 20 100,000. For the PopSizeABC analysis, SNP sites with a minor allele frequency (MAF) >0.2 were used as the input file for PopSizeABC (v2.1) [67] software with the following parameters: mac (minor allele count threshold for AFS and IBS statistics computation) = 0; mac_ld (minor allele count threshold for LD statistics computation) equals 3, 4, 5, respectively; L (size of each segment, in bp) = 4,000,000; nb_rep (number of simulated datasets) = 500; nb_seg (number of independent segments in each dataset) = 30. The results generated by the 3 methods were visualized with a generation time of 16 years and a mutation rate of 4.0 × 10^−9^ substitutions per site per generation [15].

### Comparative genomic analysis related to migration

To understand the possible genomic basis of the migration characteristics of the oriental stork, comparative genomic analyses were performed with nonmigratory bird species and other migratory birds. Unique adaptive signals detected in the oriental stork when compared with the nonmigratory birds, which were absent in the dataset comparing the oriental stork with other migratory birds, were regarded as the potential genetic basis contributing to their migratory phenotype. Here, we focused on expanded gene families, positively selected genes (PSGs) and rapidly evolving genes (REGs). Treefam (v1.4) [68] and CAFÉ (v4.2.1) [69] were used to identify expanded gene families. PSGs and REGs were identified under a branch model and a branch-site model based on the single-copy genes in the CodeML of PAML (v4.8) [57] with the threshold of the adjusted *P* value for the false discovery rate set as 0.05. GO and KEGG enrichment analyses were performed using the “cluserProfiler” package in R (v4.0.2) [70, 71]. Networks of GO terms were visualized by REVIGO to summarize redundant terms [72].

### Detecting genomic signatures of recent adaptation

SNPs in the wild population were phased by BEAGLE (v5.0) [73] with the default parameters. Recent positive selection signals were detected using the integrated haplotype score (iHS, version 1.3) [74] method, and iHS scores were normalized by subtracting the genome-wide mean iHS score and dividing by the standard deviation (calculated by the software WHAMM). SNPs with the highest or lowest 0.1% standardized iHS scores were considered candidate ancestral or derived alleles under strong positive selection. Four methods were used to select genes that were under recently positive selection: (i) genes in a 5-kb flanking region around the candidate SNPs; (ii) sliding 100-kb windows across the whole genome, with genes selected as candidates if they intersected with the 100-kb windows containing candidate SNPs; (iii) sliding 50 SNP windows across the whole genome, with genes selected as candidates if they intersected with the 50 SNP windows that contained candidate SNPs; and (iv) genes harboring candidate SNPs.

## Results

### Chromosome-level genome assembly and annotation

A reference genome for a female oriental stork was assembled by combining ONT long reads (∼89.81-fold), DNB short reads (∼69.77-fold), and Hi-C reads (∼98.20-fold) (Supplementary Table S1). The genome size of the oriental stork was estimated to be 1.29 Gb (Supplementary Fig. S1), and the final chromosome-scale genome assembly had a size of 1.24 Gb (Fig. 1C, Table 1, and Supplementary Fig. S2). The scaffold N50 of this genome was 102.77 Mb and the contig N50 was 35.79 Mb (Supplementary Table S2). More than 99.32% of all contigs were successfully anchored onto 35 chromosomes. The GC content of the oriental stork genome was 42.40%, very close to that of its related species, *C. maguari* (GenBank ID: GCA_013,399,255.1, 40.90%) and *S. umbrette* (GenBank ID: GCA_013,400,535.1, 41.50%). The chromosome-level genome assembly generated in this study also showed high completeness with a BUSCO score of 97.6% (Supplementary Table S3). Lastly, 99.73%, 99.89%, and 94.80% of the WGS, Hi-C, and RNA-seq reads, respectively, could be successfully mapped onto the final assembly (Supplementary Table S4). The depth ratio of male/female on Chr8 and Chr16 were observed to be about 2 and 0, respectively, consistent with the mapping pattern of Z and W chromosomes (Supplementary Fig. S3A–C). Syntenic analysis with *G. gallus* and *T. guttata* also supported that Chr8 and Chr16 were Z and W chromosomes, respectively (Supplementary Fig. S3D, E).

**Table 1:** Statistics of the genome assembly for the oriental stork

Genomic features	Parameters
Assembled genome size (bp)	1,240,615,254
Contig N50 (bp)	35,788,150
Scaffold N50 (bp)	102,765,642
Longest contig (bp)	131,589,000
Longest scaffold (bp)	220,403,942
GC content (%)	42.40
Percent of repetitive sequences (%)	10.41
Number of gene models	15,609

Approximately 10.41% of genome sequences were identified as repetitive elements (129.09 Mb), including long terminal repeats (LTRs) (3.46%), long interspersed nuclear elements (LINEs) (5.64%), DNA elements (0.73%), short interspersed nuclear elements (SINEs) (0.13%), and unknown repeats (0.10%) (Supplementary Table S5–S7). After masking these repeat elements, a total of 15,609 protein-coding genes were predicted in our assembly, and the average gene length, intron length, and exon length were 24.53 kb, 2.58 kb, and 173.96 bp (9.84 exons per gene), respectively, which were comparable to other avian species (Supplementary Table S8, Supplementary Fig. S4). All predicted genes (100%) were functionally annotated in at least 1 of the 5 databases used in this study (Supplementary Fig. S5, Supplementary Table S9). Additionally, 208 microRNAs (miRNAs), 152 ribosomal RNAs (rRNAs), 440 transfer RNAs (tRNAs), and 274 small nuclear RNAs (snRNAs) were predicted in this study (Supplementary Table S10). A phylogenetic tree was constructed based on 1,800 shared single-copy gene families. Aves and Crocodilian were sister clades that diverged at c. 241.4 million years ago (Mya), and the oriental stork split with *C. maguari* at c. 17.9 Mya (Fig. 1D, Supplementary Fig. S6).

### Population structure, genetic diversity, and inbreeding

In order to assess the genomic background of the oriental stork population, paired-end sequencing data of 46 individual birds (29 wild and 17 captive) were mapped to the assembled reference genome. Average sequencing coverage and depth for the 46 birds were 97.80% and 22.81-fold, respectively (Supplementary Table S11). After filtering low-quality variants and variants in the sex chromosomes, 6,525,198 qualified SNPs were obtained across 33 autosomes.

**Figure 2:**
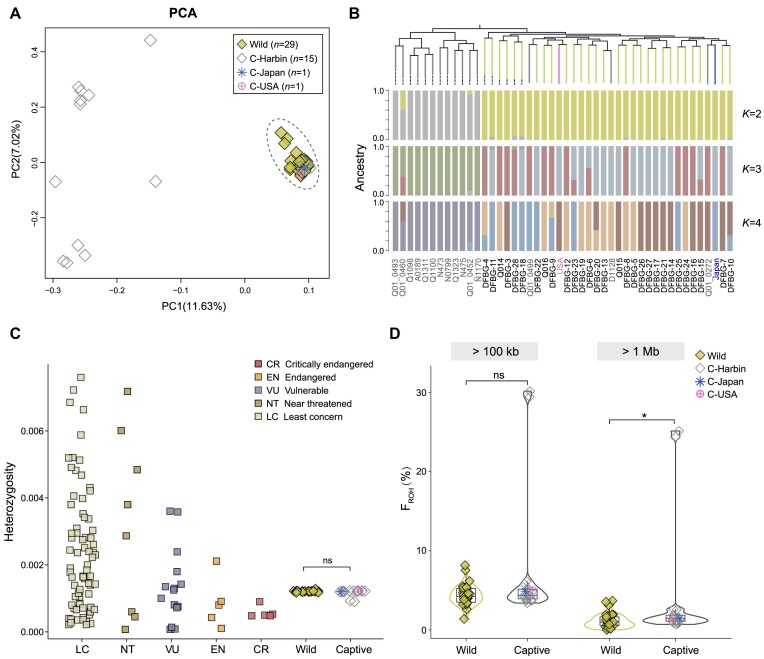
Population genetic structure, genome-wide heterozygosity, and inbreeding level of the oriental storks. (A) PCA result for the 46 birds presented with the first and second principal components. (B) Phylogenetic relationship and admixture analysis of the 46 individuals with a *K* value from 2 to 4. (C) Comparison of whole-genome heterozygosity in bird species with different threatened status defined by IUCN. (D) Individual inbreeding coefficients inferred by F_ROH_. The F_ROH_ for ROH ≥100 kb and ROH ≥1 Mb are shown here (2-sided *t* test, ns = nonsignificant, *P < 0.05).

PCA, admixture, and phylogenetic tree all supported that the wild birds belonged to a single genetic cluster while the captive individuals presented a scattered distribution (Fig. 2A, B). Five captive birds, including the 3 Harbin birds, 1 bird from Japan, and another from the United States, were clustered into the wild population, implying that the 5 birds have a very similar genetic background with the wild population. The lowest CV error for *K* = 2 suggested that there should be 2 dominant ancestral components (Fig. 2B, Supplementary Fig. S7), and admixture analysis for larger *K* values revealed that wild birds might have more complex ancestral components.

Furthermore, the genetic diversity and inbreeding levels were calculated for the oriental stork population to assess the threatened status of this species. Average genome-wide heterozygosity (*H*) of all 46 individual birds was estimated to be 1.20 × 10^−3^ ± 6.32 × 10^−5^, which was at a relatively high level among the endangered avian species (crested ibis: 4.30 × 10^−4^ [64]; saker falcon: 8.00 × 10^−4^ [75]; Chatham Island black robin: 4.80 × 10^−4^ [76]; and kākāpō: 5.00 × 10^−4^ [31]) (Fig. 2C, Supplementary Tables S12 and S13). The *H* of the wild birds was slightly higher than that of the captive birds (*H*_wild_ = 1.21 × 10^−3^ ± 2.04 × 10^−5^; *H*_captive_ = 1.18 × 10^−3^ ± 9.72 × 10^−5^) but with no significant difference. The nucleotide diversity (π) of the wild and the captive populations (π_captive_ = 1.12 × 10^−3^; π_wild_ = 1.02 × 10^−3^) was also higher than that of the brown eared pheasant (*Crossoptilon mantchuricum*, 9.60 × 10^−5^) [77] and the green peafowl (*P. muticus*, 4.70 × 10^−4^) [78] populations (Supplementary Fig. S8).

Although the oriental stork genomes presented a relatively high genome-wide genetic diversity, the average F_ROH_ ≥100 kb across the genome was around 5.56% ± 5.30% (wild: 4.44% ± 1.35%; captive: 7.47% ± 8.19%) (Fig. 2D). ROH fragments larger than 1 Mb were rare in these individuals, with an average value of 2.29% ± 4.86% (wild: 1.18% ± 0.96%; captive: 4.18% ± 7.54%). Significant differences were found between the wild and the captive populations for F_ROH_ ≥1 Mb (*P* = 0.04). Unexpectedly, 2 captive birds seemed to be highly inbred (F_ROH_: ∼30%), which was consistent with their lower *H* than others (Fig. 2C). Overall, the low-level F_ROH_ of oriental storks suggested a surprisingly low inbreeding risk.

### Higher mutational load in the wild population

**Figure 3:**
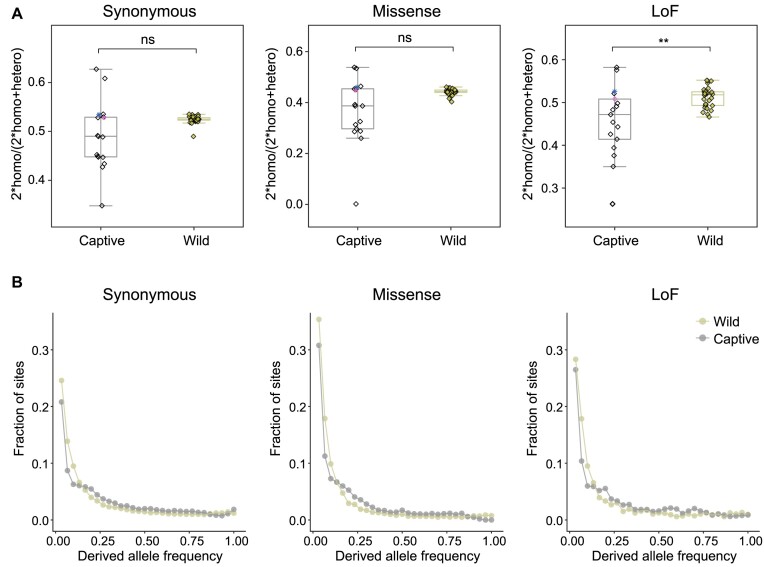
Mutational load of the wild and the captive populations. (A) Statistics of derived alleles, including synonymous, missense, and LoF mutations. The ratio of homozygous derived alleles in each individual genome is shown here (2-sided *t* test, ns = nonsignificant, **P < 0.01). (B) Folded SFS of synonymous, missense, and LoF mutations in the wild and the captive populations. The proportion of loci (y-axis) is shown for each derived allele frequency (x-axis).

Mutational load is a genetic factor associated with the fitness cost of a species. In this study, mutational load was screened across the genome in each bird, and a large number of missense and LoF mutations were identified (Fig. 3A, Supplementary Fig. S9). The average values of the derived mutational load present in both heterozygous and homozygous states in the wild population were higher than that found in the captive individuals (Supplementary Table S14). In particular, homozygous LoFs were significantly increased in the wild population (172.00 ± 8.14) compared to the captive population (139.76 ± 33.64). The frequency of LoF mutations scaled by synonymous mutations was also higher in the wild than the captive population (Supplementary Fig. S10). In total, 71.2% and 62.3% of all genes influenced by missense and LoF mutations were shared by the wild and captive populations. The wild stork population also had more private genes carrying missense mutations (Supplementary Fig. S11A). The unique genes interrupted by LoF mutations in the wild birds were not enriched in any GO terms that were related with life activities or fitness (Supplementary Fig. S11B).

We then preformed folded site frequency spectrum (SFS) analysis for the derived alleles to determine genetic drift (Fig. 3B). Both the wild and the captive populations displayed “L-shaped” lines in the 3 categories of mutations and presented a large fraction of rare derived alleles, indicating a mutation drift equilibrium [30]. However, low-frequency alleles were relatively deficient while medium-frequency alleles were excessive in the captive population when compared to the wild population, indicating that the genetic drift seemed to be slightly stronger in the captive oriental storks [77].

### Historical population dynamics

**Figure 4:**
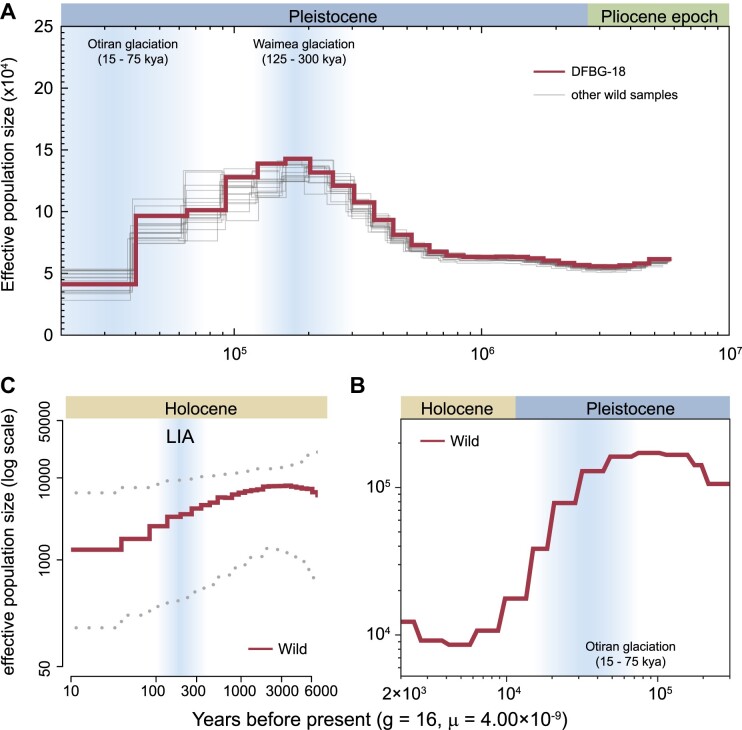
Estimated demographic history of the wild population of oriental stork. (A) Large-scale demographic fluctuation from 6 million years ago (Mya) to 20 ka BP was inferred by PSMC. (B) Recent population history over the past 200–0.3 ka estimated by SMC++ with 29 wild individuals. (C) Recent effective population size for the wild population inferred by PopSizeABC. Dotted lines indicate a 90% confidence interval. Light blue shadows depict several glacial periods, including Waimea glaciation, Otiran glaciation, and the little ice age (LIA).

The historical population dynamics is closely related to the accumulation of genetic load [79]. In order to evaluate the change in effective population sizes (*N*_e_) of oriental storks over their evolutionary history, the demographic trajectory for the last 6 million years (My) was reconstructed in this study. The entire population history of the oriental stork was characterized by 2 population expansions and 2 population declines. Wild oriental storks experienced the first population expansion at around 800–200 thousand years before present (ka BP) after a long period of retaining a steady population size, and then a serious decline occurred ca. 200–6 ka BP (Fig. 4A, B). A slight recovery occurred at 6–3 ka BP. The most recent decrease started at 3 ka BP, and the final *N*_e_ dropped to approximately 1,000 (Fig. 4C). The *N*_e_/*N*_c_ (census size, *N*_c_) ratio was about 0.33 for the contemporary wild population, which fell within the range of most species (0.5–0.10) [80].

### Genomic insights for bird migration

Long-distance migratory birds are under significant selective pressure from their early life [81]. The interplay between genetics, learning, and spatial memory plays a critical role in shaping the complex migration behavior [82, 83]. The migratory ability of the oriental stork is closely related to its survival, and a better understanding of the genomic basis for this biological adaptation is expected to facilitate the future conservation of this bird species.

**Figure 5:**
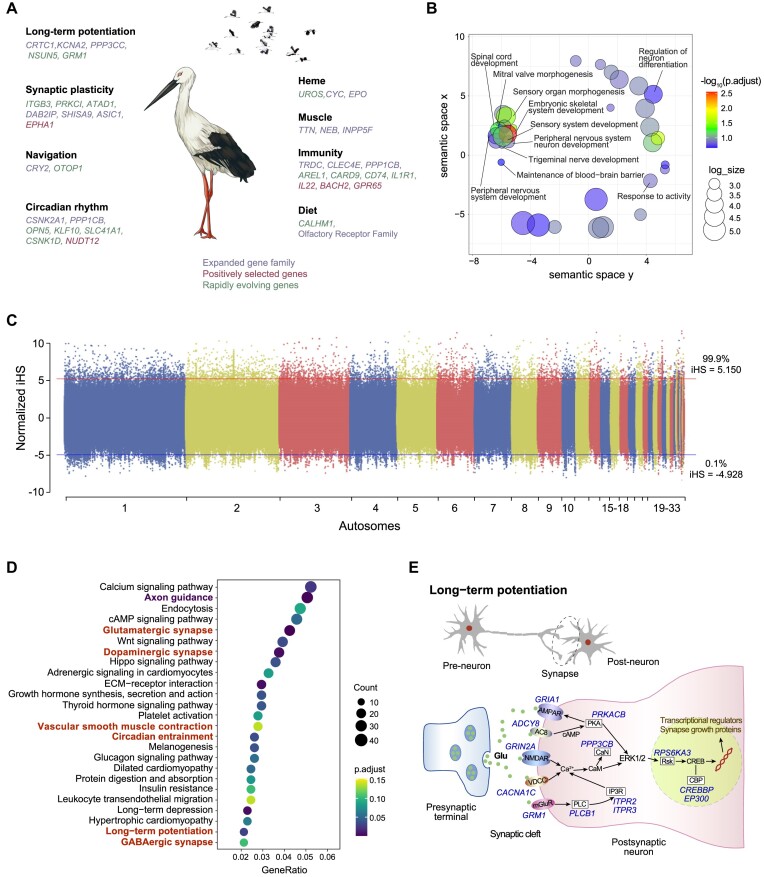
Genomic signatures relevant to migration in the oriental stork. (A) Genes or pathways that were detected may contribute to the migratory traits. (B) GO items representing biological processes by REVIGO for expanded gene families in the oriental stork genome. Semantic similar GO terms clustered together. (C) Normalized iHS scores indicating candidate SNPs under recent positive selection in the wild population. Red and blue lines represented the 99.9th and 0.1th quartile of iHS scores, respectively. (D) KEGG pathway analysis for recently positive selection. (E) Genes with recent positive selective signals that related to the long-term potentiation pathway.

Here, the oriental stork was compared with other avian species with or without the migratory trait (Supplementary Fig. S12). A total of 526 expanded gene families, 107 PSGs, and 308 REGs were identified in the oriental stork genome compared with nonmigratory birds (uM group). Meanwhile, a total of 557 expanded families, 90 PSGs, and 279 REGs were identified in the oriental stork genome when other full-migratory birds (M group) were used for comparisons (Supplementary Fig. S13). Here, only the genes found in the analysis with the uM group but not found in the analysis with the M group were considered candidate genes that were more likely the genomic basis for the migratory traits associated with the oriental stork. Our findings suggest a portion of these genes with functions related to the migration (Fig. 5A, Supplementary Tables S15–S17). GO enrichment analysis of the expanded gene families showed that a series of GO terms were enriched in the sensory system development and peripheral nervous system development (Fig. 5B), which may be important to increase the sensitivity to environmental changes and transmit these signals to the central nervous system. Notably, the trigeminal nerve development (GO:0,021,559) was distinctly enriched in the GO analysis, which was previously shown to be vital for the development of map sense in night-migratory songbirds [84]. Of particular interest, radical pairs of cryptochromes are magnetically sensitive and CRY4 is responsible for the light-dependent magnetic compass in the night-migratory European robin [85]. Here, the CRY2 gene family was found to be expanded in oriental stork but was not found in nonmigratory birds (Supplementary Fig. S14). Long-term potentiation in the hippocampus is closely related to memory and learning, which contributes to the migratory route formation in peregrine falcons [83]. As for the oriental stork, 2 PSGs (*SPG11* and *EPHA1*) and 9 REGs (*ITGB3, NSUN5, KCTD16, PRKCI, ATAD1, EPHA1, GRM1, ADGRL3*, and *NEXMIF*) were found involved in synaptic plasticity. The *NSUN5* gene product is essential for NMDAr-dependent long-term potentiation, and *Nsun5*-KO mice showed spatial cognitive deficits [86]. The *ATAD1* gene encodes ATPase family AAA domain-containing protein 1, which controls AMPA receptor (AMPAR) internalization that regulates synaptic activity. Absence of *ATAD1* would affect the amplitude of miniature excitatory postsynaptic currents and finally cause deficits in learning and memory [87].

For long-distance migrants, their breast or flight muscles comprise red muscles with a high concentration of myoglobin. The muscles are highly vascularized and the cells contain a high concentration of mitochondria. These adaptations enable the birds to carry on oxidative metabolism for prolonged periods of time when the birds are in flight [88, 89]. We identified genes related to heme biosynthesis (REG: *UROS*; expanded gene families: *CYC* and *EPO* genes) and genes that code for proteins involved in muscle development (expanded gene families: *TTN, ENB*, and *INPP5F* genes). In terms of immunity, several genes involved in pathogen clearance were identified, which are of vital importance to the innate immune response.

Additionally, 10,312 SNPs were identified to be under recent positive selection detected by the iHS method based on population genomic sequencing data, and 25.92% of these SNPs were distributed within genic regions (1,453 genes) (Fig. 5C, Supplementary Fig. S15). GO and KEGG enrichment analyses revealed a series of biological functions and pathways associated with the neurons, including learning and memory (GO:0,007,611), synaptic plasticity (GO:0,048,167), axon development (GO:0,061,564), glutamatergic synapse (hsa04724), dopaminergic synapse (hsa04728), and GABAergic synapse (hsa04727) (Fig. 5D, Supplementary Tables S18 and S19). Our finding confirmed the presence of a positively selected SNP in the *ADCY8* gene. The *ADCY8* gene was previously shown to be important in the long-distance migratory peregrine populations [83]. Besides the *ADCY8* gene, there were another 12 positively selected genes functioning in the long-term potentiation pathway: 4 genes (*GRIA1, GRIN2A, CACNA1C*, and *GRM1*) encoding membrane receptors, 5 genes (*PRKACB, PPP3CB, PLCB1, ITPR2*, and *ITPR3*) responsible for intracellular signal transduction, and 3 genes (*RPS6KA3, CREBBP*, and *EP300*) affecting nucleus transcription activity. The discovery of these genes and their associated pathways provides clues to uncover the genetic factors shaping the migratory route of oriental storks (Fig. 5E).

## Discussion

Species are disappearing at an accelerated rate due to pervasive anthropogenic impacts [90, 91]. A record of the past 600 years reflected a peak in bird extinction rates in the 19th century, which occurred first in the Pacific islands [92]. This extinction contrasts the fast substantial diversification of the relatively young avian clade [93]. Conservation efforts have been enhanced with the rapid development of genome sequencing technologies [23, 94]. However, genomic resources and genomic investigations are still lacking for many threatened species [95]. Here we assembled a high-quality reference genome and sequenced the genomes of both wild and captive cohorts of the endangered oriental stork. The findings of our study would make up for the knowledge deficit surrounding the genetic background of both the wild and captive populations of oriental storks. The knowledge from the study will further benefit future conservation plans.

The assembled genome comprised 33 autosomes and the Z and W sex chromosomes. Our assembly was consistent with the previous karyotypical study for the oriental stork (2*n* = 68) [96, 97]. Sex chromosomes and microchromosomes are relatively difficult to be assembled and deserve special attention, and the synteny analysis could help confirm the accuracy of the assembly and unanchored scaffolds like those found in the wood stork (*Mycteria americana*) study [98]. The Z chromosome could not be not completely assembled in this study, because the assembled length was shorter than the reported karyotype. Microchromosomes were clearly divided into 23 contacted scaffold groups, showing the effectiveness of Hi-C data. Our assembled genome can be regarded as a new representative reference genome and provides a valuable genomic resource for further ecological and evolutionary studies and conservation efforts of the oriental stork, as well as other stork species.

The wild population of the oriental stork has experienced a serious decline in the past century, and the population size has started to increase in recent years under conservation efforts. Oriental storks are still classified as an “endangered” species, with no more than 2,500 mature individuals. More than 95% of the individuals live in a single subpopulation [15]. Consistently, population genomic analysis did not show a genetic structure in the wild population. And fortunately, the wild population was found to possess high genetic diversity and low inbreeding level (F_ROH_ <10%), which may result from their migration every year along the EAAF. This contrasts with some geographically isolated and highly inbred animal populations [31, 99]. Interestingly, 2 highly inbred captive birds (F_ROH_ ≈30%) with decreased heterozygosity and a significantly higher level of long ROH fragments (>1 Mb) were identified, which more likely originated from the recent inbreeding. This phenomenon sheds light on the importance of the scientific pedigree management in the small breeding populations with limited founders.

Endangered species with different historical population dynamics usually face different extinction risks [100, 101]. It has been demonstrated that small populations experiencing long-term declines would have low genetic diversity and high levels of inbreeding. In contrast, a recent rapidly decline has less effects on the genetic diversity [102]. For the oriental stork, the wild population increased in size during 6–3 ka BP and started to decline at 3 ka BP, approximately 187 generations (g) ago. The population history may explain the relatively high genetic diversity found in the wild population nowadays. The relatively large *N*_e_ (from 10,000 to 1,000) over the recent 3 ky (thousand years) might be responsible for the low levels of inbreeding and reducing genetic drift in the wild population.

The relatively high genetic diversity and a recent history of population decline in oriental storks has endowed the population with a strong genetic capacity to recover. The rapid recent decline of the oriental stork population might have resulted from both climate change and human activities. Their breeding area, including the Amur River basin, spanned Russia, China, and Mongolia. These areas were originally covered by temperate forests and populated by a relatively small number of nomadic people living on hunting and fishing. Around 3 kya (thousand years ago), the climate in this region turned cold and dry. The forest system was converted to wetlands, and thus, the nest trees for the oriental storks became scarce [103]. As human society moved from the Bronze Age to Iron Age around 2.6 kya, an increase in human activities might have led to habitat loss, causing a decline in the bird population [103]. Over the past several centuries, the intensity of human activities has increased manifold. Colonization of Russia in the 17th century brought modern agriculture and economic development [104]. Agricultural activities in Northeast China started in 2 waves in the Liao-Jin period and in the Qing Dynasty [105]. Human settlements in this region experienced the most rapid expansion in the past 100 years, with a large-scale agricultural reclamation process on the China side of the Amur River basin [106]. Wetlands in the Sanjiang plain have declined in area by 86% from 108,900 km^2^ prior to the 20th century to some 14,800 km^2^ in 2000 [107]. Moreover, the most important stop-over site of the oriental storks, the Bohai Bay, is an area with the most concentrated coastal reclamation activities in China [108]. Natural wetlands experienced striking environmental changes driven by rapid industrialization and urbanization in the past century [109]. In the Japanese and Korean peninsulas, the climate has been changing periodically. In the recent century, rapid industrialization and economic expansion in both Japan and Korea have destroyed many natural habitats. The rapidly industrialized agriculture and heavy use of chemical fertilizers and pesticides have reduced the natural food sources of the oriental stork, contributing to the extinction of this bird species in both Japan and Korea since 1971. In summary, habitat degradation due to recent human activities may explain the serious decline in the oriental stork population and other birds along the EAAF.

Migrants may adaptively adjust migratory routes and wintering areas in response to climate change and anthropogenic influence [110]. Improved evolutionary insights on the migration can facilitate the conservation for migratory birds. Billions of animals migrate annually across the planet, in pursuit of improved foraging opportunities, safety, and reproductive output [111]. Migrants face selective pressure to arrive early to occupy high-quality territories at the stop-over/wintering sites [112]. Efficient migration requires coordinated support of the brain function and physical conditions [83, 85, 113]. Specific phenotypes and physiological functions are often hypothesized to be attributed to the selection of the underlying protein-coding genes [114, 115]. A series of expanded gene families, PSGs, and REGs related to learning and memory, navigation, muscle development, and energy metabolism were identified in the oriental stork. Recent work has revealed that adult birds shape their migratory route through individual exploring and learning over a lifetime [82]. Long-term potentiation is critical for the large-scale spatial memory. For example, the long-distance migratory population of the peregrine falcon experienced distinct selection on the *ADCY8* gene, which regulates downstream memory-related genes [83]. Here, we also found that the *ADCY8* gene, together with other genes along the long-term potentiation pathway, was under positive selection in the oriental storks, and several biological pathways responsible for neuronal synapse formation were overrepresented. These findings provide new insights into the genomic basis of the migratory performance of the oriental stork, as well as the possible flexibility of this species in responding to the environmental changes through learning and memory.

## Supplementary Material

giae081_GIGA-D-23-00340_Original_Submission

giae081_GIGA-D-23-00340_Revision_1

giae081_GIGA-D-23-00340_Revision_2

giae081_Response_to_Reviewer_Comments_Original_Submission

giae081_Response_to_Reviewer_Comments_Revision_1

giae081_Reviewer_1_Report_Original_Submission Ke He -- 1/22/2024 Reviewed

giae081_Reviewer_1_Report_Revision_1 Ke He -- 5/7/2024 Reviewed

giae081_Reviewer_2_Report_Original_Submission Kristina M. Ramstad -- 1/23/2024 Reviewed

giae081_Supplemental_Files

## Data Availability

The final genome assembly data, RNA-seq data, and raw resequencing genome data are available in the NCBI BioProject repository (accession number: PRJNA1036389). All additional supporting data are available in the *GigaScience* repository, GigaDB [116].

## Additional Files

**Supplementary Fig. S1**. The distribution of 21-mer for the genome size estimation of the oriental stork.

**Supplementary Fig. S2**. Heatmap of Hi-C chromosomal interaction density among all 35 chromosomes.

**Supplementary Fig. S3**. Identification of sex chromosomes in the oriental stork genome.

**Supplementary Fig. S4**. Comparison of gene characteristics of avian species and human.

**Supplementary Fig. S5**. Venn diagram representing the functional annotation of the oriental stork gene set.

**Supplementary Fig. S6**. Comparison of the gene repertoires of 24 vertebrate genomes.

**Supplementary Fig. S7**. Cross-validation (CV) error in the ADMIXTURE analysis.

**Supplementary Fig. S8**. Nucleotide diversity (π) across 25 autosomes in wild and captive populations, respectively, by sliding a 5-Mb window.

**Supplementary Fig. S9**. Statistics of the number of heterozygous and homozygous loci for synonymous, missense, and LoF mutations in each individual.

**Supplementary Fig. S10**. Statistics of the frequency of heterozygous and homozygous loci and sites of missense and LoF mutations, scaled by synonymous mutations.

**Supplementary Fig. S11**. Genes harboring putative missense and LoF mutations.

**Supplementary Fig. S12**. The expanded and contracted gene families in each bird genome and the comparison of their gene repertoires.

**Supplementary Fig. S13**. Comparison of detected genomic signals in the oriental stork genome compared with nonmigratory birds and migratory birds, including expanded families, positively selected genes, and rapidly evolving genes.

**Supplementary Fig. S14**. The phylogenetic tree of the *CRY2* gene constructed by the maximum likelihood method. The red clade represented 3 *CRY2* genes in oriental stork.

**Supplementary Fig. S15**. Venn diagram of the 4 methods used to identify genes affected by candidate SNPs under recent selection.

**Supplementary Table S1**. Statistics of sequencing data for genome assembly and gene annotation.

**Supplementary Table S2**. Genomic statistics of the *C. boyciana* assembly.

**Supplementary Table S3**. BUSCO analysis of the whole genome and the gene set of the *C. boyciana* genome.

**Supplementary Table S4**. Statistics of sequencing data mapped to the *C. boyciana* genome that were used for the assembly and gene annotation.

**Supplementary Table S5**. Transposable element (TE) statistics in our assembly.

**Supplementary Table S6**. Statistics of identified repeats by *de novo* method in the *C. boyciana* genome.

**Supplementary Table S7**. Transposable elements in the *C. boyciana* assembly.

**Supplementary Table S8**. Statistics of annotations for the *C. boyciana* gene set.

**Supplementary Table S9**. Statistics on functional annotation of the *C. boyciana* gene set.

**Supplementary Table S10**. Statistics of ncRNA annotation.

**Supplementary Table S11**. List of 46 oriental stork samples used for resequencing.

**Supplementary Table S12**. Whole-genome heterozygosity and F_ROH_ of 46 resequenced samples.

**Supplementary Table S13**. Whole-genome heterozygosity for the published birds presented in Fig. 2C.

**Supplementary Table S14**. Average number of heterozygous and homozygous loci and all sites for derived alleles in wild and captive populations.

**Supplementary Table S15**. Expanded gene families in the *C. boyciana* genome compared with nonmigratory birds.

**Supplementary Table S16**. Rapidly evolving genes in *C. boyciana* genome compared with nonmigratory birds.

**Supplementary Table S17**. Positively selected genes in the *C. boyciana* genome compared with nonmigratory birds.

**Supplementary Table S18**. GO enrichment of genes directly affected by positively selected SNPs.

**Supplementary Table S19**. KEGG pathway enrichment of genes directly affected by positively selected SNPs.

## Abbreviations

ABC: approximate Bayesian computation; BLAST: Basic Local Alignment Search Tool; BP: before present; BUSCO: Benchmarking Universal Single-Copy Orthologs; BWA: Burrows–Wheeler aligner; Chr: chromosome; CRY2: cryptochrome circadian regulator 2; CV: cross-validation; EAAF: East Asian-Australasian Flyway; Gb: gigabase pairs; GO: Gene Ontology; gVCF: genomic variant call format; H: heterozygosity; Hi-C: high-throughput chromosome conformation capture; IBA: Important Bird and Biodiversity Area; iHS: integrated haplotype score; IUCN: International Union for Conservation of Nature; kb: kilobase pairs; KEGG: Kyoto Encyclopedia of Genes and Genomes; LIA: little ice age; LINE: long interspersed nuclear element; LoF: loss of function; LTR: long terminal repeat; MAF: minor allele frequency; Mb: megabase pairs; Mya: million years ago; Nc: the census size; NCBI: National Center for Biotechnology Information; Ne: the effective size; ONT: Oxford Nanopore Technology; PCA: principal component analysis; PSG: positively selected gene; PSMC: pairwise sequentially Markovian coalescent; REG: rapidly evolving gene; RNA-seq: RNA sequencing; ROH: run of homozygosity; SFS: site frequency spectrum; SINE: short interspersed nuclear element; SNP: single-nucleotide polymorphism; WGS: whole-genome sequencing.
