# Supplementary material for: Genomic exploration of the endangered oriental stork, Ciconia boyciana, sheds light on migration adaptation and future conservation
Source: Gigascience. 2024 Oct 22;13:giae081. doi: 10.1093/gigascience/giae081 (PMC11494145; doi:10.1093/gigascience/giae081)
Supplement: giae081_GIGA-D-23-00340_Original_Submission [file giae081_giga-d-23-00340_original_submission.pdf]

GigaScience

Genomic exploration of the endangered oriental stork, Ciconia boyciana, shed lights on migration adaptation and future conservation

--Manuscript Draft--

**Manuscript Number:**	GIGA-D-23-00340
**Full Title:**	Genomic exploration of the endangered oriental stork, Ciconia boyciana, shed lights on migration adaptation and future conservation
**Article Type:**	Research
**Funding Information:**	Leading Talent Project of "Science and Technology Leading Talent Team Project of Inner Mongolia Autonomous Region (2022LJRC0010) — Not applicable<br>Surveillance of Wildlife Diseases from the State Forestry Administration of China (2023057) — Not applicable
**Abstract:**	Abstract<br>Background: The oriental stork, Ciconia boyciana, is an endangered migratory bird classified by IUCN. They experienced a rapidly decline in the past decades, with nest locations and stopover sites largely degraded due to human-bird conflicts. The genome-wide genetic status of this threatened bird population is critical to make future conservation strategies but lack of investigation.<br>Findings: We presented the first chromosome-scale genome for the oriental stork with high quality, contiguity, and accuracy. The assembled genome size was 1.24 Gb with a scaffold N50 of 103 Mb, and 1.23 Gb contigs (99.32%) were anchored to 37 chromosomes. Comparative genomic and nature selection analyses identified essential genomic signals associated with migratory trait, involving long-term potentiation, photoreceptor cell, circadian rhythm, muscle development and energy metabolism. Genetic diversity of oriental stork was still relatively high among all recorded bird species and inbreeding risk was not concerning (FROH = 0.29%). Demographic history reconstruction showed a recently decline trend, explaining for its high diversity and foreshadowing its recovery potential.<br>Conclusions: The first chromosome-scale genome of oriental stork further expands genomic resource of endangered birds, providing a genomic basis for understanding the migratory characteristic. The comprehensive exploration of genome-wide genetic status will improve the making of future conservation plans for the oriental stork.<br>Keywords: oriental stork, comparative genome, population genetics, endangered species, migration
**Corresponding Author:**	Shangchen Yang<br>Zhejiang University<br>Hangzhou City, CHINA
**Corresponding Author Secondary Information:**	
**Corresponding Author's Institution:**	Zhejiang University
**Corresponding Author's Secondary Institution:**	
**First Author:**	Shangchen Yang
**First Author Secondary Information:**	
**Order of Authors:**	Shangchen Yang
	Yan Liu
	Xiaoqing Zhao
	Jin Chen
	Haimeng Li

Powered by Editorial Manager® and ProduXion Manager® from Aries Systems Corporation

	Hongrui Liang
	Mengchao Zhou
	Shiqing Wang
	Xiaotian Zhang
	Minhui Shi
	Lei Han
	Mingyuan Yu
	Yaxian Lu
	Boyang Liu
	Yu Xu
	Tianming Lan
	Zhijun Hou
**Order of Authors Secondary Information:**	
**Additional Information:**	
**Question**	**Response**
Are you submitting this manuscript to a special series or article collection?	No
**Experimental design and statistics**<br><br>Full details of the experimental design and statistical methods used should be given in the Methods section, as detailed in our Minimum Standards Reporting Checklist. Information essential to interpreting the data presented should be made available in the figure legends.<br><br>Have you included all the information requested in your manuscript?	Yes
**Resources**<br><br>A description of all resources used, including antibodies, cell lines, animals and software tools, with enough information to allow them to be uniquely identified, should be included in the Methods section. Authors are strongly encouraged to cite Research Resource Identifiers (RRIDs) for antibodies, model organisms and tools, where possible.	Yes

Powered by Editorial Manager® and ProduXion Manager® from Aries Systems Corporation

Have you included the information requested as detailed in our Minimum Standards Reporting Checklist?	
**Availability of data and materials**<br><br>All datasets and code on which the conclusions of the paper rely must be either included in your submission or deposited in publicly available repositories (where available and ethically appropriate), referencing such data using a unique identifier in the references and in the “Availability of Data and Materials” section of your manuscript.<br><br>Have you have met the above requirement as detailed in our Minimum Standards Reporting Checklist?	Yes

Powered by Editorial Manager® and ProduXion Manager® from Aries Systems Corporation

Manuscript

Click here to access/download;Manuscript;Genomic exploration of the endangered oriental stork-1106.docx

Genomic exploration of the endangered oriental stork, *Ciconia boyciana*, shed lights on migration adaptation and future conservation

Shangchen Yang[1,2†], Yan Liu[3†], Xiaoqing Zhao[4,5†], Jin Chen[1], Haimeng Li[1], Hongrui Liang[3], Mengchao Zhou[1], Shiqing Wang[6], Xiaotian Zhang[3], Minhui Shi[6], Lei Han[1], Mingyuan Yu[3], Yaxian Lu[1], Boyang Liu[1], Yu Xu[3‡], Tianming Lan[1*], Zhijun Hou[1*]

[1]College of Wildlife and Protected Area, Northeast Forestry University, Harbin, 150040, China

[2]College of Life Sciences, Zhejiang University, Hangzhou 310058, China

[3]Center for Biological Disaster Prevention and Control, National Forestry and Grassland Administration, Shenyang 110034, China

[4]Inner Mongolia Academy of Agricultural & Animal Husbandry Sciences, Hohhot 010031, Inner Mongolia, China;

[5]Key Laboratory of Black Soil Protection and Utilization (Hohhot), Ministry of Agriculture and Rural Affairs, P.R. China, Hohhot 010031, Inner Mongolia, China;

[6]College of Life Sciences, University of Chinese Academy of Sciences, Beijing, 100049, China

[*]**Correspondence address.** Tianming Lan, College of Wildlife and Protected Area, Northeast Forestry University, Harbin, China. E-mail: lantianming@genomics.cn; Zhijun Hou, College of Wildlife and Protected Area, Northeast Forestry University, Harbin, China. E-mail: houzhijundb@163.com

[†]These authors contributed equally to this work.

[‡]This author jointly supervised the work.

Abstract

Background: The oriental stork, *Ciconia boyciana*, is an endangered migratory bird classified by IUCN. They experienced a rapidly decline in the past decades, with nest locations and stopover sites largely degraded due to human-bird conflicts. The genome-wide genetic status of this threatened bird population is critical to make future conservation strategies but lack of investigation.

Findings: We presented the first chromosome-scale genome for the oriental stork with high quality, contiguity, and accuracy. The assembled genome size was 1.24 Gb with a scaffold N50 of 103 Mb, and 1.23 Gb contigs (99.32%) were anchored to 37 chromosomes. Comparative genomic and nature selection analyses identified essential genomic signals associated with migratory trait, involving long-term potentiation, photoreceptor cell, circadian rhythm, muscle development and energy metabolism. Genetic diversity of oriental stork was still relatively high among all recorded bird species and inbreeding risk was not concerning (F_{ROH} = 0.29%). Demographic history reconstruction showed a recently decline trend, explaining for its high diversity and foreshadowing its recovery potential.

Conclusions: The first chromosome-scale genome of oriental stork further expands genomic resource of endangered birds, providing a genomic basis for understanding the migratory characteristic. The comprehensive exploration of genome-wide genetic status will improve the making of future conservation plans for the oriental stork.

Keywords: oriental stork, comparative genome, population genetics, endangered species, migration

Introduction

Ecosystem degradation and biodiversity decline occur as a feature throughout the Anthropocene and would likely be accelerated in the coming years[1]. Human-induced habitat loss, overexploitation, and pollution largely speed up the pace of the sixth mass extinction and now more than 32% of all species (~42,100) are threatened with extinction[2]. This global crisis in turn harm to human well-being, and urgently need more conservation efforts to stop and reverse current situation.

Birds are effective wildlife indicator of the biodiversity and earth health[3]. Long-term record from BirdLife International raised significant concern for the world's birds that 49% of bird species (5,412) have declining populations including endangered and common birds, and many are at an escalating risk of extinction. Nearly 45% of Important Bird and Biodiversity Areas (IBAs) are identified in danger due to pervasive and unsustainable human activities, like agricultural expansion and intensification, logging, and hunting[4-6]. In particular, wetlands in the East Asian-Australasian Flyway (EAAF) (Fig. 1A) are being heavily destroyed, directly causing key sites loss and bird mortality[7, 8]. This flyway is used by 492 migratory bird species, totally more than 50 million individuals, from Arctic Russia and Alaska, to Australia and New Zealand [9, 10]. Due to human-bird conflicts in Asia, around half of the migratory waterbirds suffering population falling, cases in point are the critically endangered spoon-billed sandpiper *Calidris pygmaea* [11] and the endangered oriental stork *Ciconia boyciana*[12]. Migratory birds are wildlife without boundaries and serve as a powerful safeguard for distant countries and ecosystems. The EAAF decline largely reduce the energy connectivity and mobility between Arctic nutrient-poor terrestrial system and southern coastline ecosystem. Although government moves to protect coastal wetlands to protect migratory birds, there are many gaps to fill, such as a comprehensive evaluation on the status, trends, and threats[13]. Yet, for most of these threatened migratory birds, there lack species and population-level whole-genome data to further assess their biodiversity and endangered status, bring obstacles to determine targeted recovery actions.

Oriental stork is a large and heavy wader among the 492 migratory bird species sharing the EAAF. It was currently listed as “endangered” on the IUCN Red List, with an estimated population size of c. 3000 individuals by 2018 and a single subpopulation[14]. They breed in southeastern Siberia, primarily along the Russia-China border and annually migrate to Bohai Bay (1,500 km) and Poyang Lake (2,600 km) in autumn (Fig. 1B) [15]. They once suffered a significant and rapid decline due to high risk in their breeding grounds and on passage last century. Deforestation, agricultural development and spring fires severely destroy their nest trees in Russia. Reclamation of wetlands, and overfishing in the stop-over and wintering sites in China lead to decreased refueling rate and increased mortality rate, particularly the juveniles[14, 16]. Whether such decline heavily impacts the oriental stork population and even their survival were still largely underexplored. Future conservation actions need to be enhanced with advanced technologies like high-quality genome assembly to better understand their seasonal migration, recovery potential, and genetic risks.

Here, we presented the first chromosome-level genome for oriental stork, and re-sequenced 29 wild and 15 captive individuals to explore genomic characteristics of this wetland shorebird species. We scanned the whole genome to find genomic signatures of migratory-related evolution and adaptation, which might be vital for the maintenance of population viability. We also systematically investigated the genetic background and genetic risks of this species, including genetic diversity, population demography, inbreeding, deleterious mutations, and so forth. We finally compared genomic parameters between wild and captive individuals to explore possible genetic rescue strategies to recover the population. This study will provide a valuable genomic resource to access the ecological adaption and conservation status of the oriental stork and aid for their conservation.

Methods

Samples and ethics statement

Blood samples from 16 captive-born and 3 rescued wild oriental storks were collected at Harbin North Forest Zoo, Harbin City, Heilongjiang Province, China. The sample used for reference genome assembly was divided into four tubes with 2.5 ml per tube for Oxford Nanopore Technologies (ONT) long-read, Hi-C, whole-genome short-read and transcriptomic sequencing. Muscle samples from 26 rescued wild oriental storks were collected around the Bohai Bay, China. Research and blood/tissue collection were approved by the Institutional Review Board of Northeast Forest University.

Nucleic acid and transcriptome extraction, library construction and sequencing

For ONT library preparation, 8-10 μg of gDNA was size-selected (> 50 kb) according to the manufacturer's instructions and 800 ng DNA libraries were used for sequencing on the PromethION sequencer (Oxford Nanopore Technologies, UK). For Hi-C sequencing, cross-link process with formaldehyde was firstly conducted using the blood sample and then Hi-C library was constructed following the protocol of Lieberman-Aiden et al[17]. Total RNA was extracted using TRlzol reagent (Invitrogen), and Agilent 2100 Bioanalyser system (Agilent) and Qubit 3.0 (Life Technologies) were used for RNA quantity, integrity, and purity evaluation. DNA libraries with short insert sizes were prepared according the manufacturer's instruction of the MGI platform (MGI, Shenzhen, China). All these DNA libraries were finally sequenced on the DNBSEQ-T1 platform for 100-bp paired-end reads.

De novo assembly, annotation and assessment

Genome size of oriental stork was estimated by *k*-mer frequency method using WGS data of the assembled

individual[18]. Firstly, *de novo* assembly was conducted using ONT long reads by NextDenovo(v2.5.0, https://github.com/Nextomics/NextDenovo). Two core modules were utilized to generate a primary assembly: the NextCorrect module was for raw ONT long-reads correction and consensus sequence extraction; the NextGraph module was for preliminary assembly. Here, we set the read cutoff as 1 Kb and maintained other default parameters in NextDenovo. Then, contigs were polished using NextPolish(v1.4.0)[19] with ONT long reads. Hi-C reads were mapped to the genome using Burrows-Wheeler aligner *mem* (BWA, v0.7.17)[20] with default parameters. 3d-DNA pipeline(v180,922) was applied to convert the contigs into a chromosome-level genome. Finally, WGS reads were remapped to the assembly[21] to error-correct the mis-sequenced bases from the long-read based sequencing. Benchmarking Universal Single-Copy Orthologs (BUSCO) analysis[22] was performed to evaluate the completeness of our assembly using aves_odb10 database. Lastly, WGS, Hi-C and RNA-seq data were mapped to the final genome to check mapping rate, bases coverage and sequencing depth by BWA *mem* algorithm with default parameters.

De novo and homology-based methods were combined to identify repetitive elements in the final assembly. Firstly, *de novo* prediction was realized using LTR finder(v1.0.6)[23], MITE-hunter(v4.07)[24] and RepeatModeler2(v2.0.1)[25] software with default parameters. The results were merged into the RepBase as known repeats. Next, RepeatMasker(v4.0.5)[26] was used to identify and classify transposable elements by searching RepBase library[27]. Finally, tandem repeats were identified using Tandem Repeats Finder (TRF, v4.09)[28].

Firstly, all repetitive elements were masked to annotate the protein-coding genes on our assembled genome. Next, we used a combination of de novo, homology-based and transcript mapping methods to conduct gene annotation. De novo prediction was realized using SNAP(v1.0)[29], glimmerHMM(v3.0.3)[30] and AUGUSTUS(v2.5.5)[31] software. RNA data was filtered by Trimmomatic(v0.27)[32] and assembled by Trinity(v2.9.0)[33] and then mapped to the reference genome to predict gene structure using Program to Assemble Spliced Alignments (PASA, v2.2.0)[34].

For homology-based prediction, we used protein sequences from *Gallus gallus*, *Anas platyrhynchos*, *Ciconia maguari*, *Meleagris gallopavo*, *Pavo muticus*, *Taeniopygia guttata* and *Homo sapiens*, to align against our genome by Blastall(v2.2.26)[35] with E-value cut-off of 1e-5 and then gene models were confirmed using GeneWise(v2.4.1)[36]. The results of three approaches above were finally combined to generate a comprehensive gene set by Maker(v 3.01.03)[37]. These genes were aligned in the databases of SwissProt, TrEMBL, InterPro, Gene ontology (GO) and Kyoto Encyclopedia of Genes and Genomes (KEGG) to predict gene function.

Identification of sex-linked regions

Sex chromosomes (Z and W) were firstly identified by checking the different sequencing depth of male and female individuals. Then we examined syntenic relationships with sex chromosomes of *G. gallus* (GenBank ID: GCA_016699485.1) and *T. guttata* (GenBank ID: GCF_003957565.2). Alternative splicing of each gene on the chromosomes were filtered for the three species. We aligned the longest protein sequence of *G. gallus* and *T. guttata* against our assembled Z and W chromosomes using blastp in BLASTtools(v2.2.26)[38] with the parameter of "-evalue 1e-5". And then, synteny blocks were identified using MCScanX[39] and visualized by Circos(v0.69-9)[40] software.

Phylogeny reconstruction and divergence time estimation

We performed a protein alignment of 24 species (*H. sapiens*, *Anolis carolinensis*, *Alligator sinensis*, *G. gallus*, *Cygnus olor*, *Asarcornis scutulata*, *A. platyrhynchos*, *T. guttata*, *Strigops habroptilus*, *Falco peregrinus*, *Herpetotheres cachinnans*, *Spizaetus tyrannus*, *Accipiter gentilis*, *Haliaeetus albicilla*, *Charadrius vociferus*, *Rostratula benghalensis*, *Larus smithsonianus*, *Balearica regulorum*, *Grus americana*, *C. maguari*, oriental stork,

Scopus umbrette, *Nipponia nippon*, *Egretta garzetta*). The longest protein sequence of each gene was aligned together by BLASTtools(v2.2.26)[38] blastp function with "-evalue 1e-5". A total of 1,800 shared single-copy genes were used to construct a phylogenetic tree by IQTREE(v1.6.12)[41] using the maximum-likelihood method. Divergence time among these species was then estimated by MCMCTREE(v4.5) in PAML[42] software with multiple fossil time points used for time calibration[43].

Comparative genomic analysis related to migration

To understand the genome evolution in oriental stork, we conducted comparative genomic analyses with nonmigratory bird species, also compared with other migratory birds. Unique adaptative signals detected in oriental stork with nonmigratory group, which were absent in migrants, were regarded as potential genetic factors contributing to the migratory phenotype. We looked for expanded gene families, positively selected genes (PSGs) and rapidly evolving genes (REGs). We firstly clustered all genes in two groups of species, respectively, by using Treefam(v1.4)[44] and used CAFÉ(v4.2.1)[45] to identify expanded and contracted gene families. PSGs and REGs were identified under a branch model and a branch-site model based on the single-copy gene sets in the CodeML of PAML(v4.8)[42] with the threshold of the false discovery rate adjusted *P*-value set as 0.05. GO and KEGG enrichment analyses were performed using the "cluserProfiler" package in R (v4.0.2)[46, 47]. Networks of GO terms were visualized by REVIGO to summarize redundant terms[48].

Variant calling and filtering

Raw sequencing data of 46 individuals from natural wetland (*n*=26), Harbin North Forest Zoo (3 wild and 15 captive), Kanagawa (hereafter, Japan; *n*=1) and San Diego Zoo (hereafter, USA; *n*=1) were mapped to our assembled genome

using the BWA *mem* with default parameters. Reads sorting, reordering and deduplication were done with Picard(v2.1.1) software and then variant calling was performed using Sentieon(v202010.01)[49] DNAseq Haplotyper. Bam files and genomic Variant Call Format (gVCF) file were generated for each individual and joint calling was conducted using Sentieon DNAseq GVCFtyper to present a combined VCF file covering all individuals. Variants were filtered using the following procedures: 1) InDels and multi-allelic variants were removed; 2) hard filtering with the parameters: QD < 2.0 || FS > 60.0 || MQ < 40.0 || MQRankSum < -12.5 || ReadPosRankSum < -8.0 –filter-name snp_filter; 3) genotype quality of a site lower than 20 were marked as missed site; 4) missing ratio larger than 1% were removed from the variation set. Single-nucleotide polymorphism (SNP) sites on the Z and W chromosomes were also removed for the downstream population genomic analysis.

Population structure analysis

The VCF file was firstly converted into plink format files with VCFtools(v0.1.16)[50] and then PCA was performed with Plink(v1.9)[51] software. Inference of ancestral components in each genome was conducted with ADMIXTURE(v1.3.0)[52] and *K* value was set from one to five with "-cv" flag to calculate the cross-validation error. Phylogenetic tree was constructed using IQTREE(v1.6.12) with 1000 bootstraps. The tree layout was visualized by applying the online tool iTOL(http://itol.embl.de).

Screening of genomic signatures of recent adaptation

SNP sites were split for the wild population and phased by BEAGLE(v5.0)[53] with the default parameters. The major allele in wild population was set as the ancestral state at each site. Recently positive selection was detected using the integrated haplotype score (iHS, version 1.3)[54] method and iHS scores were normalized by subtracting

the genome-wide mean his and dividing by the standard deviation by using the software WHAMM (http://coruscant.itmat.upenn.edu/whamm/index.html). SNPs with the highest or lowest 0.1% standardized iHS scores were considered as candidate ancestral or derived alleles that were under strong positive selection. We applied four approaches parallelly to summing up iHS scores of candidate SNPs in each window: (1) sliding 100-kb windows by 50-kb step on the whole genome; (2) 5-kb flanking regions of each candidate SNP; (3) non-overlapping 50-SNP windows; (4) each gene region. These windows were sorted by the total iHS score and genes intersecting with these windows were considered as potentially influenced ones. Metascape[55] were used to enrich terms and pathways for detected gene set. We also performed GO and KEGG analysis in R environment. For the genes we were interested in, haplotype bifurcation for target SNPs were calculated and showed using rehh[56] package in R.

Genetic diversity and inbreeding

Genome-wide heterozygosity (*H*) of each individual was the proportion of heterozygous SNPs in the successfully assembled autosomal genome, which was calculated by the VCFtools(v0.1.16). Nucleotide diversity (π) was calculated by a non-overlapping 5-Mbp sliding window along all autosomes using VCFtools(v0.1.16). Runs of homozygosity (ROHs) were identified by Plink(v1.9) with the following parameters: --homozyg-window-snp 20 --homozyg-kb 10 --homozyg-density 50. Inbreeding coefficient was estimated as the proportion of genome fraction that in ROH region (F_{ROH}). Comparison between wild and captive populations was conducted using two-sided pairwise *t*-test in R(v 4.1.2).

Mutational load

We used alleles in *C. maguari* genome, the closest relative of oriental stork, to serve as ancestral state of each site.

We applied the same method for alignment as previously described[57]. The reference of *C. maguari* (GenBank ID: GCA_013399255.1) was transformed to a 100 bp FASTQ file by sliding a nonoverlapping window across the genome and then short reads were mapped to our assembled genome using BWA *mem* with the parameter: -B 3. Only reads mapped uniquely to our genome were kept by SAMtools(v1.3)[58] view function with "-F 4 -q 20". Finally, we generated a consensus sequence to represent ancestral alleles on the oriental stork genome using SAMtools(v1.3) mpileup function with depth filter of ≤ 1×. We obtained a new VCF file containing 6,028,662 derived SNPs after replacing the reference alleles by a Perl script.

SnpEff (v4.3) software was used to divide the derived SNPs into three categories: (1) synonymous mutations; (2) missense mutations; (3) loss of function (LoF) mutations. Here, we considered "stop_gained", "splice_donor_variant" and "splice_acceptor_variant", "start_lost", "stop_lost" and "splice_region_variant" as LoF mutations. Next, we counted the number of derived SNPs per individual in homozygous and heterozygous state, respectively. The proportion of homozygous derived SNPs was measured following this formula: 2 × homozygous sites / (2 × homozygous sites + heterozygous sites)[59].

Demographic dynamics

We combined Pairwise Sequentially Markovian Coalescent (PSMC, v0.6.5)[60], SMC++(v1.13.1)[61] and approximate Bayesian computation (ABC) methods to track the demographic dynamics of wild population from ancient time scale to recent days. Firstly, we converted the bam file of each individual to a fasta sequence using SAMtools(v1.3)[58] mpileup function, and whole-genome sequencing depth ≥ 1/3 and ≤ 2 was filtered. PSMC software was then run with the parameters: -N25 -t5 -r5 -p 4+25*2+4+6. Secondly, we randomly selected two individuals from wild population to generate a mask file of uncovered regions by bamCaller.py. SMC++ was applied

based on covered sites to infer population history with the following parameters: --cores 8 --knots 24 --timepoints 20 100000. Thirdly, SNP sites with a MAF > 0.2 were used as an input file for PopSizeABC(v2.1)[62] software with the parameters: mac (minor allele count threshold for AFS and IBS statistics computation) = 0; mac_ld (minor allele count threshold for LD statistics computation) equals 3,4,5 respectively; L (size of each segment, in bp) = 4,000,000; nb_rep (number of simulated data sets) = 500; nb_seg (number of independent segments in each data set) = 30. The output results of three methods were visualized with a generation time of 16 years and the mutation rate of 4.0×10^{-9} substitutions per site per generation[14].

Results

Chromosome-level genome assembly

We assembled a chromosome-level reference genome for oriental stork by combining ONT long reads, DNBSEQ short reads and Hi-C sequencing data (Supplementary Table S1), (Fig. 1C, Table 1). Firstly, genome size was estimated to be 1.29 Gb based on the K-mer frequency method [18](Supplementary Fig. S1). Then we used ONT long reads to produce a draft genome with a contig N50 of 35.79 Mb (Supplementary Table S2) after polishing by WGS data. Using Hi-C reads, we further anchored genomic fragments (99.32% of contigs) onto 37 pseudochromosomes (Supplementary Fig. S2). We finally generated a chromosome-scale genome (genome size: 1.24Gb) with the scaffold N50 being of 102.77 Mb. The GC content of this genome assembly was 42.40%, very close to that of its related species, *C. maguari* (GenBank ID: GCA_013399255.1, 40.90%) and *S. umbrette* (GenBank ID: GCA_013400535.1, 41.50%). Our assembly also showed a high completeness with high Benchmarking Universal Single-Copy Orthologs (BUSCO) value (97.6%) (Supplementary Table S3). Lastly, 99.73%, 99.89% and 94.80% of the WGS, Hi-C and RNA-seq reads could be successfully mapped onto our final assembly

(Supplementary Table S4), further supporting the accuracy and completeness of genome assembly in this study.

Table 1: Statistics of this assembly for oriental stork.

**Genomic features**	**Oriental stork**
Assembled genome size (bp)	1,240,615,254
Contig N50 (bp)	35,788,150
Scaffold N50 (bp)	102,765,642
Longest contig (bp)	131,589,000
Longest scaffold (bp)	220,403,942
GC content (%)	42.40
Percent of repetitive sequences (%)	10.41
Number of gene models	15,609

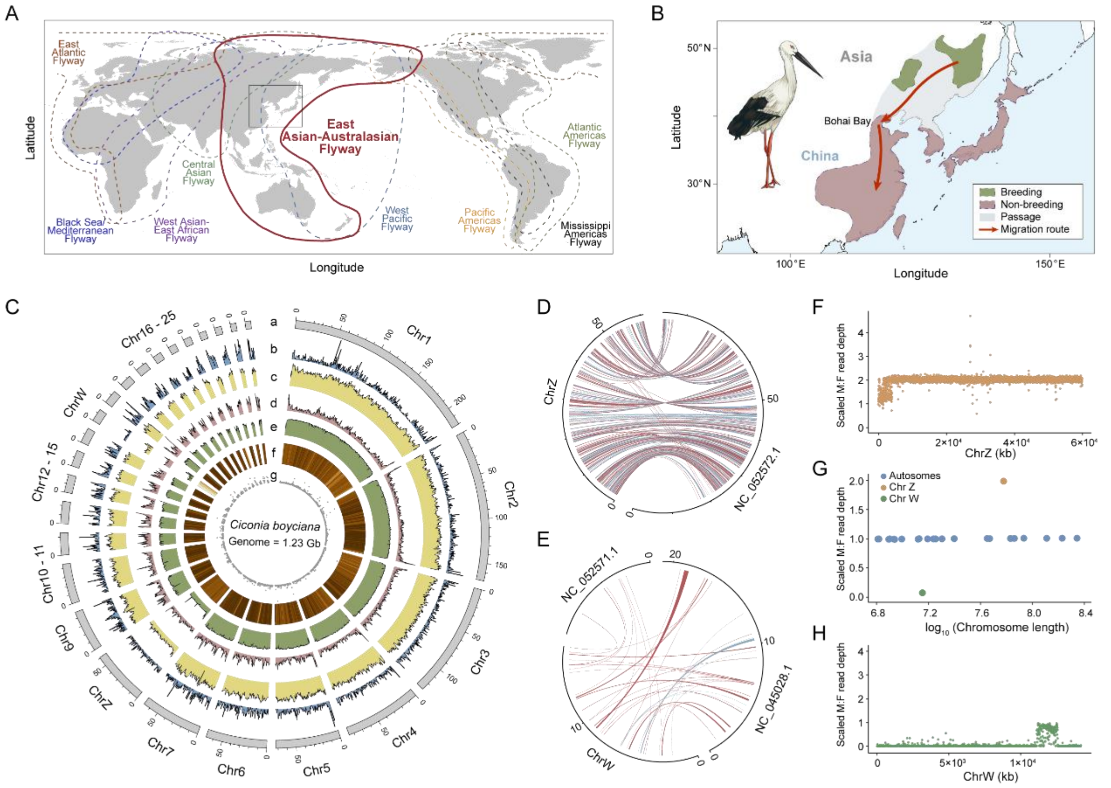

Figure 1: Geographical distribution of oriental storks and landscape of oriental stork genome. (A) Nine major flyways across the globe[6]. Black box indicated the range of oriental storks. (B) Breeding and wintering sites, migratory route and sampling site (Bohai Bay) of oriental storks in this study. (C) Distribution of genomic features in oriental stork. (a) The 26 chromosomes larger than 5 Mb. (b) Gene count. (c) Depth of Hi-C reads. (d) GC content density. (e) Depth of WGS reads. (f) Repeat number. (g) Depth of RNA reads. The statistics were calculated using a 500-kbp window. (D) Synteny analysis of Z chromosome between oriental stork and *G. gallus*. (E) Synteny analysis of W chromosome between oriental stork and *G. gallus*, and *T. guttata*. Red lines indicated genes on the positive strand and blue lines indicated genes on the negative strand of oriental stork genome aligned with that of the other bird genome. (F) The ratio of sequencing depth between male and female individuals in a 10kbp window across the Z chromosome. (G) The ratio of the sequencing depth between male and female individuals of each chromosome-scale scaffold larger than 5 Mb. The orange dot represented the ChrZ, the green dot represented the ChrW, and the blue dots represented autosomes. The expected ratio is 1:1, the Z chromosome is expected to be 2:1 and the W chromosome is 0:1. (H) The ratio of sequencing depth between male and female individuals in a 10kbp window across the W chromosome.

To identify the sex chromosomes, we calculated and compared the average sequencing depth of each large chromosomes of known sex (3 females and 4 males). We observed that scaled depth ratio of male compared with female of Chr8 and Chr37 were about 2 and 0, respectively, consistent with the mapping pattern of Z and W chromosomes (Fig. 1F-H). Further, syntenic analysis identified 432 genes presented high collinearity between Chr8 and Z chromosome (NC_052572.1) of *G. gallus* (Fig. 1D), and 38 genes between Chr37 and published W chromosomes (7 with NC_052571.1 of, *G. gallus*, 31 with NC_045028.1 of *T. guttata*) (Fig. 1E). These results together supported that the Chr8 and Chr37 were corresponded to Z and W chromosomes, respectively.

Genome annotation

We identified 10.41% of genome sequences as repetitive elements (129.09 Mb), including LTRs (3.46%), LINEs (5.64%), DNA elements (0.73%), SINEs (0.13%) and unknown repeats (0.10%) (Supplementary Table S5-S7). After masking these repeat elements, a total of 15,609 protein-coding genes were predicted in our assembly by combing evidence of *de novo* prediction, homology-based alignment and transcript mapping method (Supplementary Table S8). The average gene length, intron length and exon length were 24.53 kb, 2.58 kb and 173.96 bp (9.84 exons per gene), respectively, which were comparable to other avian species (Supplementary Fig. S3). All predicted genes were functionally annotated in at least one of the five databases we used (Supplementary Fig. S4, Supplementary Table S9). Additionally, 208 miRNA, 152 rRNA, 440 tRNA and 274 snRNA were predicted in this study (Supplementary Table S10).

Comparative genomics provides insights into migration

A phylogenetic tree was firstly constructed with another 23 species based on 1800 shared single-copy gene families (Fig. 2A, Supplementary Fig. S5). Aves and Crocodilian were sister clades that diverged c. 241.4 Mya, and the oriental stork split with *C. maguari* c. 17.9 Mya.

To search for genomic imprints of migratory-relevant evolution, we reconstructed two phylogenetic trees for migratory birds and non-migratory birds with oriental stork, respectively (Supplementary Fig. S6). We identified 526 and 467 expanded gene families, 107 and 109 positively selected genes (PSGs), and 308 and 224 rapidly evolving genes (REGs) for oriental stork when compared with non-migratory and migratory birds, respectively. Among them, 557 genes in expanded families, 90 PSGs and 279 REGs were found uniquely when compared with non-migratory group (Supplementary Fig. S7), implying potential migratory-related evolution of protein-coding genes.

Long-distance migration needs multiscale collaborative approaches in nervous system and physical energy supply. Here we presented potentially genetic basis for long-distance migration at several sensory systems that are important for orientation and navigation (Fig.2B; Supplementary Table S11-S13). Firstly, GO enrichment analysis of expanded gene families showed that a series of GO terms were enriched in sensory system development and peripheral nervous system development (Fig. 2C), which may be helpful to increase the sensitivity to environmental changes and transmit these signals to central nervous system. Notably, trigeminal nerve development (GO:0021559) was distinctly enriched here, which was previously proved to be vital for the map sense in night-migratory songbirds[63]. Of particular interest, radical pairs of cryptochromes are magnetically sensitive and CRY4 is responsible for the light-dependent magnetic compass in the night-migratory European robin[64]. Here, we found that *CRY2* gene family was expanded in oriental stork while didn't show in non-migratory birds (Supplementary Fig. S8).

Secondly, for the single-copy genes, we also detected a series of PSGs and REGs likely associated with migratory-

related traits. Long-term potentiation in the hippocampus is closely related to memory and learning, which contributes to the migratory route maintenance [65]. Two PSGs (*SPG11* and *EPHA1*) and nine REGs (*ITGB3*, *NSUN5*, *KCTD16*, *PRKCI*, *ATAD1*, *EPHA1*, *GRM1*, *ADGRL3*, and *NEXMIF*) were found to be involved in synaptic plasticity. *NSUN5* gene is essential in NMDAr-dependent long-term potentiation and *Nsun5*-KO mice showed spatial cognitive deficits[66]. *ATAD1* gene encodes ATPase family AAA domain-containing protein 1, which controls AMPA receptor (AMPAR) internalization that regulates synaptic activity. Absence of *ATAD1* would affect the amplitude of miniature excitatory postsynaptic currents and finally cause deficits in learning and memory[67]. For long distance migrants, their breast or flight muscles are red muscles with a high concentration of the pigment myoglobin, more capillaries and mitochondria, which are slow muscles that could supply long periods of oxidative metabolism[68, 69]. Here, we also identified genomic signals in genes related to heme (*UROS*, *CYC*, and *EPO* gene) and muscle (*TTN*, *ENB*, and *INPP5F* gene) (Fig. 2B; Supplementary Table S11-S13).

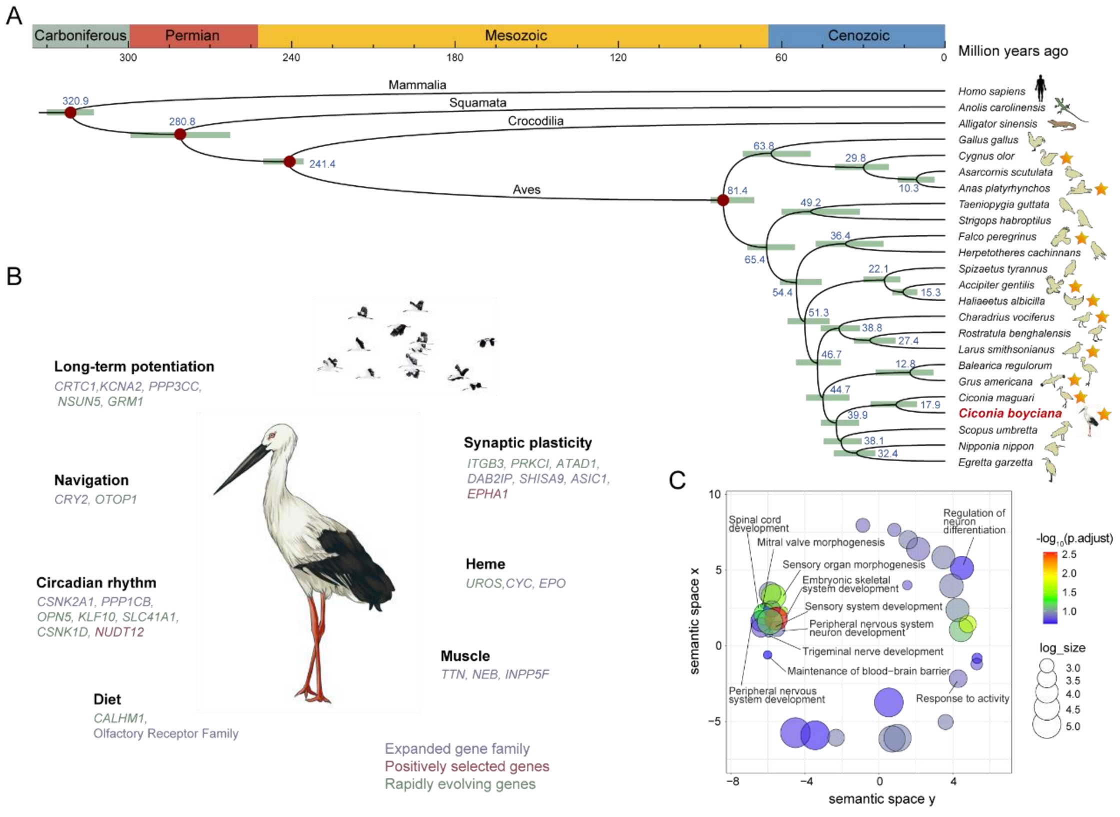

Figure 2: Comparative genomic analysis and potential genic cues relevant to migration. (A) Phylogenetic relationship of 24 species

and the estimated divergence time. Orange stars represented full migrants. (B) Genes that we inferred may contribute to migration in oriental stork. (C) GO items representing biological process by REVIGO for expanded gene families in oriental stork genome. Semantic similar GO terms clustered together.

Population-level nature selection reveal evolutionary adaptation of migration

In order to detect population level variants, we mapped paired-end sequencing data of 46 individuals (29 wild and 17 captive) to the assembled reference genome. Average sequencing coverage and depth for these individuals were 97.80 % and 22.81-fold, respectively (Supplementary Table S14). After filtering low-quality variations (see methods), we obtained 6,525,198 qualified SNPs across autosomes for following downstream analysis.

Although the 29 wild samples were collected from one site, these individuals are representative due to their migratory nature. For the captive individuals, however, they cannot be simply identified as a single population, because the origin of these individuals is complex and may have very different population background. Therefore, nature selection was explored in 29 wild individuals with clear sampling location, not in captive individuals due to their unknown and complex origins. By applying the iHS analysis, we identified 10,312 SNPs under positive selection (Fig. 3A) and 25.92% of them (2,673 SNPs) were directly distributed within gene region (1,453 genes). In addition, 3173 genes could also be identified by three other approaches (see online method and Supplementary Fig. S9). The 1453 genes harboring candidate SNPs were generally related to regulation of membrane potential, sensory organ development, brain development, heart development, regulation of GTPase activity and VEGFA-VEGFR2 signaling pathway (Supplementary Fig. S10). Further, we performed GO and KEGG enrichment analyses for these genes to reveal biological functions (Fig.3B-E; Supplementary Table S15 and S16). Many processes and pathways associated with synapse organization were presented, including glutamatergic synapse(hsa04724), dopaminergic

synapse(hsa04728), GABAergic synapse(hsa04727), axon development (GO:0061564), synaptic plasticity (GO:0048167), learning and memory (GO:0007611) (Fig.3B and F). Consistent physiological symptoms are also embodied in muscle tissue development (Fig.3C), circadian entrainment, including response to light stimulus (Fig.3D) and temperature stimulus (Fig.3E). Noteworthily, we observed that the well-known *ADCY8* gene encompassed a selective SNP, which was also selected in long-distance migratory peregrine populations and involved in migratory route formation[65]. Visualization of long-term potentiation pathway showed that candidate SNPs might affect five membrane receptors, intracellular signal transduction and finally nucleus transcription activities (Fig. 3G). These selective signals indicated the power of nature selection in shaping genotype frequencies in diverse aspects of genetic adaptability essential to a migratory phenotype.

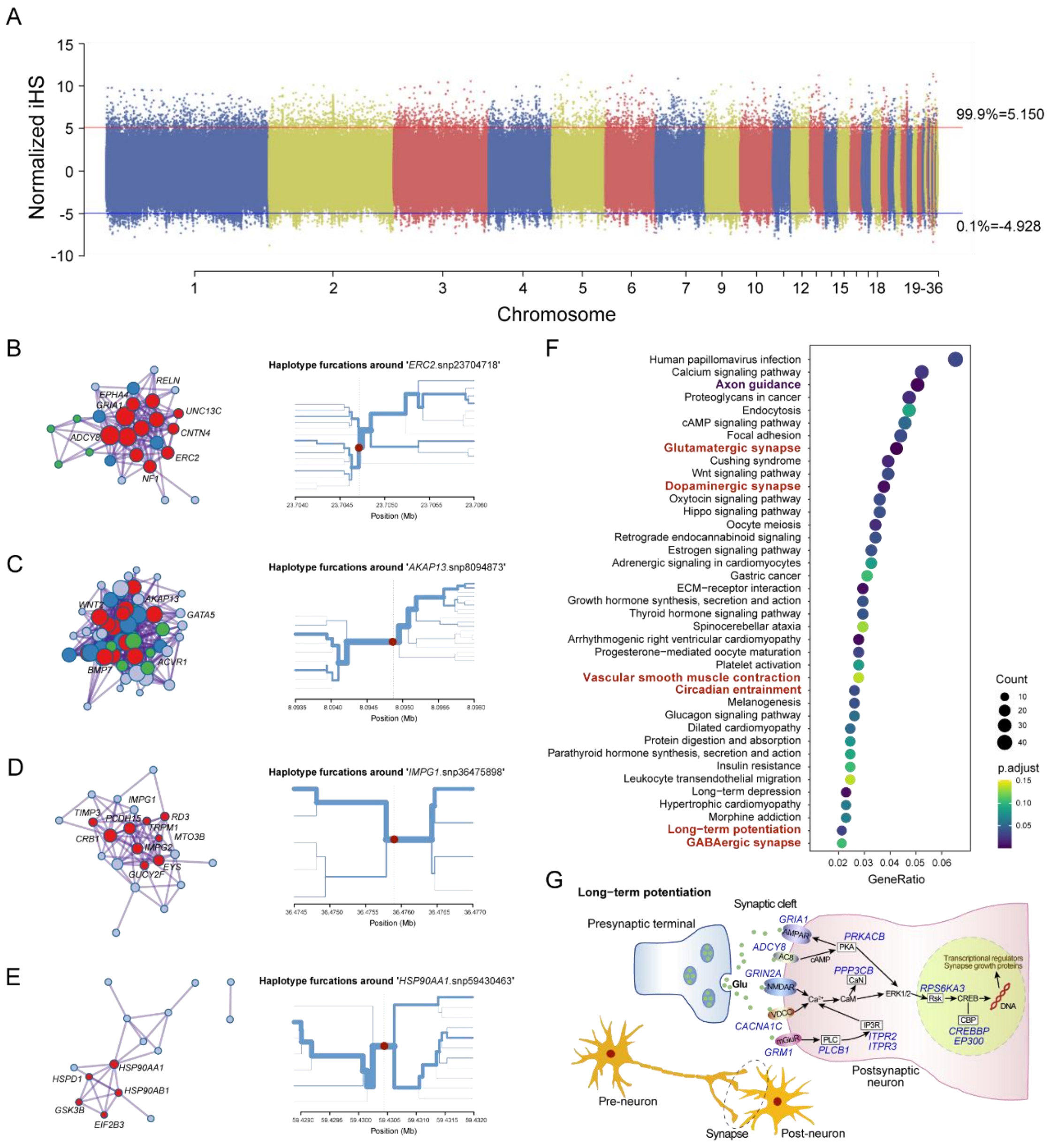

Figure 3: Nature selection in oriental storks. (A) Normalized iHS score indicated candidate SNPs under recently natural selection in the wild population. Red and blue lines represented the 99.9th and 0.1th quartile, respectively. (B-E) Protein interaction networks of GO terms for synapse organization (B), muscle development (C), sensory perception of light stimulus (D) and response to temperature (E) and representative haplotypes bifurcation diagrams around selective SNPs. (F) KEGG pathways for selective genes. (G) Genes harboring selective SNPs that on the long-term potentiation pathway.

Population structure, genetic diversity and inbreeding

Principal component analysis (PCA), admixture and phylogenetic tree all supported that wild individuals form a distinct cluster from captive individuals (Fig. 4A-C). Additionally, five captive individuals from Harbin, Japan and USA clustered into the wild population, implying that these five individuals may originate from this wild population. The lowest CV error for *K*=2 suggested there were two dominant ancestral components (Fig. 4B, Supplementary Fig. S11). Moreover, admixture analysis for larger *K* values revealed that wild individuals had more complex ancestral genetic components.

We further calculated the genetic diversity and inbreeding level for the oriental stork population, which were two essential indexes for assessment of conservation status. Average genome-wide heterozygosity (*H*) of all 46 individuals was estimated to be $1.20\times10^{-3} \pm 6.32\times10^{-5}$, and the *H* for wild individuals ($1.21\times10^{-3} \pm 2.04\times10^{-5}$) was slightly higher than that of captive individuals ($1.18\times10^{-3} \pm 9.72\times10^{-5}$), but with no significant difference (Fig. 4D, Supplementary Table S17). They seemed to have a high heterozygosity when compared with other endangered avian species (crested ibis: 4.30×10^{-4}[59]; saker falcon: 8.00×10^{-4}[70]; Chatham Island black robin: 4.80×10^{-4}[71]; and kākāpō: 5.00×10^{-4}[72]) (Fig. 4D, Supplementary Table S18). Population-level nucleotide diversity (π)(Supplementary Fig. S12) was also higher than crested ibis[59], brown eared pheasant (*Crossoptilon mantchuricum*)[73] and kākāpō[72].

We found few genomic regions were located in runs of homozygosity (ROHs) regions with an average $F_{\mathrm{ROH} \geqslant 100\,\mathrm{kb}}$ value of 0.29% ± 0.31% (wild: 0.30% ± 0.37%; captive: 0.28% ± 0.19%) (Fig.4E). Long ROH larger than 1 Mb was also rare in these individuals with an average value of 0.10% ± 0.23% (wild: 0.10% ± 0.26%; captive: 0.09% ± 0.15%). In addition, no significant difference was found in F_{ROH} between wild and captive populations. Overall, such low level of F_{ROH} suggested a surprisingly low possibility of inbreeding, comparing to many small and isolated

populations of mammals, reptiles and avian[72, 74, 75].

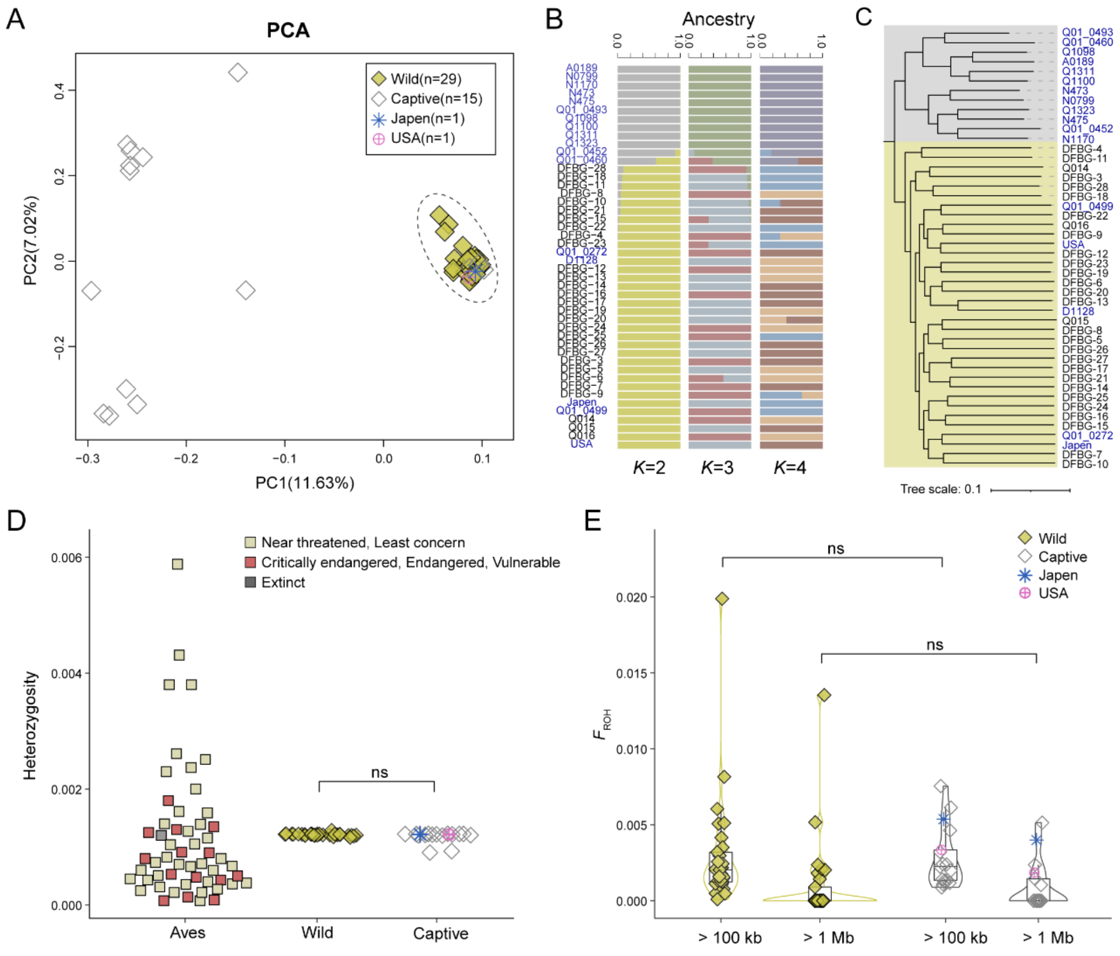

Figure 4: Genetic structure, genome-wide heterozygosity and inbreeding in the wild and captive oriental storks. (A) Principal component analysis of 46 individuals showing the first and second principal components. (B) Admixture analysis of the 46 individuals using the maximum likelihood method with a model with two to four ancestral components. (C) Phylogenetic relationship of 46 re-sequenced individuals. (D) Comparison of whole-genome heterozygosity among oriental storks and other avians of different threatened levels. (E) Individual inbreeding coefficients inferred from the proportion of the genome within ROHs (F_{ROH}). The F_{ROH} for ROH ≥ 100 Kb and ROH ≥ 1 Mb are both shown.

Higher mutational load in the wild population

Mutational load is a genetic factor associated with fitness of a species. Mutational load was calculated in each individual and we generally identified a large number of sites carrying derived synonymous, missense and loss of function (LoF) mutations (Fig. 5A, Supplementary Fig. S13). Average numbers of both heterozygous and homozygous mutational loads in the wild population were higher than that in captive individuals (Supplementary Table S19). In particularly, homozygous LoF were significantly increased in wild population. We further compared the frequency of LoF mutations scaled by synonymous mutations in each individual, and the result also supported higher frequencies in the wild population (Supplementary Fig. S14). As to putatively influenced genes by missense and LoF, respectively, 71.2% and 62.3% of them could be both found in wild and captive individuals, but much more genes were unique in the wild population than in the captive individuals (Fig. 5B). For the unique genes carrying LoF mutations in the wild, we didn't find any GO terms that seemed to influence life activities or fitness (Fig. 5C). Next, we explored the distribution of derived allele frequency of LoF and missense mutations in both captive and wild groups. In captive group, we found more than 30% and 40% LoF and missense mutations were absent, respectively. However, this proportion decreased to nearly 4% and 6% for LoF and missense mutations in the wild population (Fig. 5D). Although most derived alleles were rare both in both wild and captive groups, the fitted line all had a slope smaller than 1, which meant that many non-synonymous derived alleles had a larger frequency and fixed rate in the wild population (Fig. 5E).

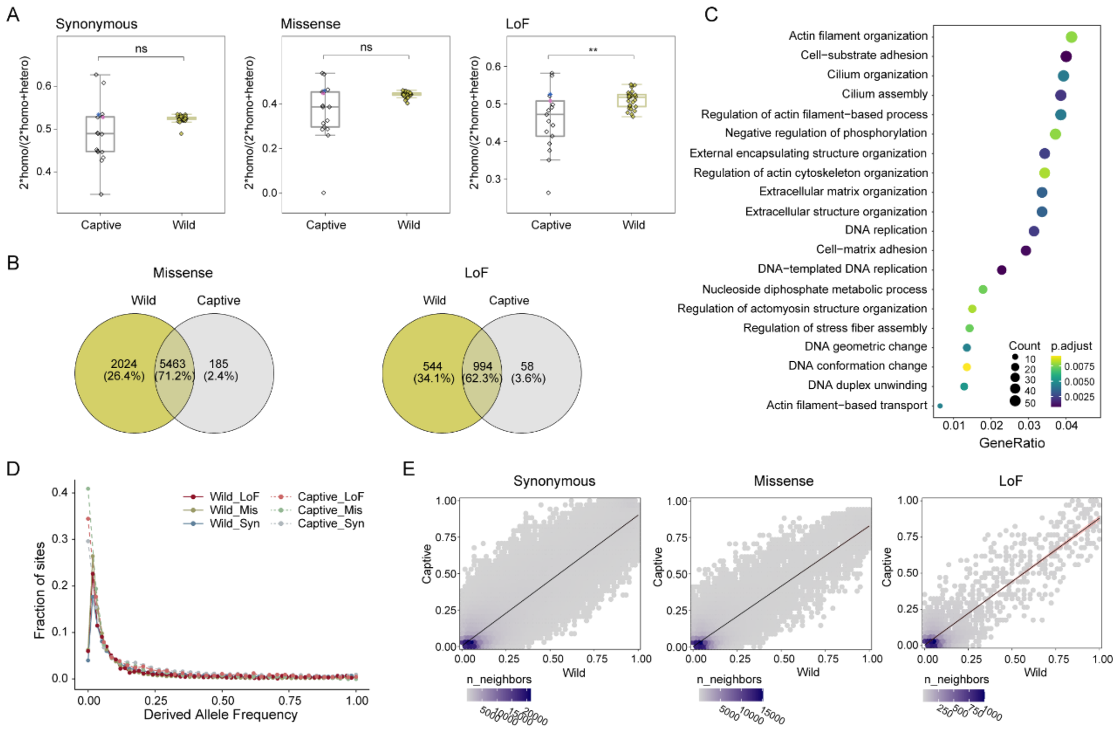

Figure 5: Mutational load of the wild and captive populations. (A) Statistics of derived alleles, including synonymous, missense and LoF mutations. The ratio of homozygous derived alleles in each individual genome were shown here. (B) Genes of the wild and captive populations that missense and LoF mutations located in, respectively. (C) GO enrichment for the unique genes with LoF mutations in the wild population. (D) Site-frequency spectrum for synonymous, missense and LoF mutations of the wild and captive populations. The proportion of loci (y axis) is shown for each possible derived allele frequency (x axis). (E) Two-dimensional unfold site frequency spectrum for synonymous, missense and LoF mutations in wild (x axis) and captive (y axis) populations. The number of derived mutations is color-coded according to the scale plot below.

Demographic trajectory

In order to evaluate the change of effective population sizes (*N*e) of oriental storks over its evolutionary history, we reconstructed their demographic trajectory since 6 million years before present (BP) till present day. The whole population history could be divided into four phases and characterized by two expansion and two declines. Wild

population experienced the first increasing at around 800-200 ka BP after a long steady period and then a serious decreasing occurred ca. 200-6 ka BP (Fig. 6A and B). After that, a slight recovery occurred in the following several thousand years, sometime between 3 and 10 ka BP. Recent population fluctuation also implied a trend of slow decline since 3 ka BP with a final *Ne* around 1,000 (Fig. 6C).

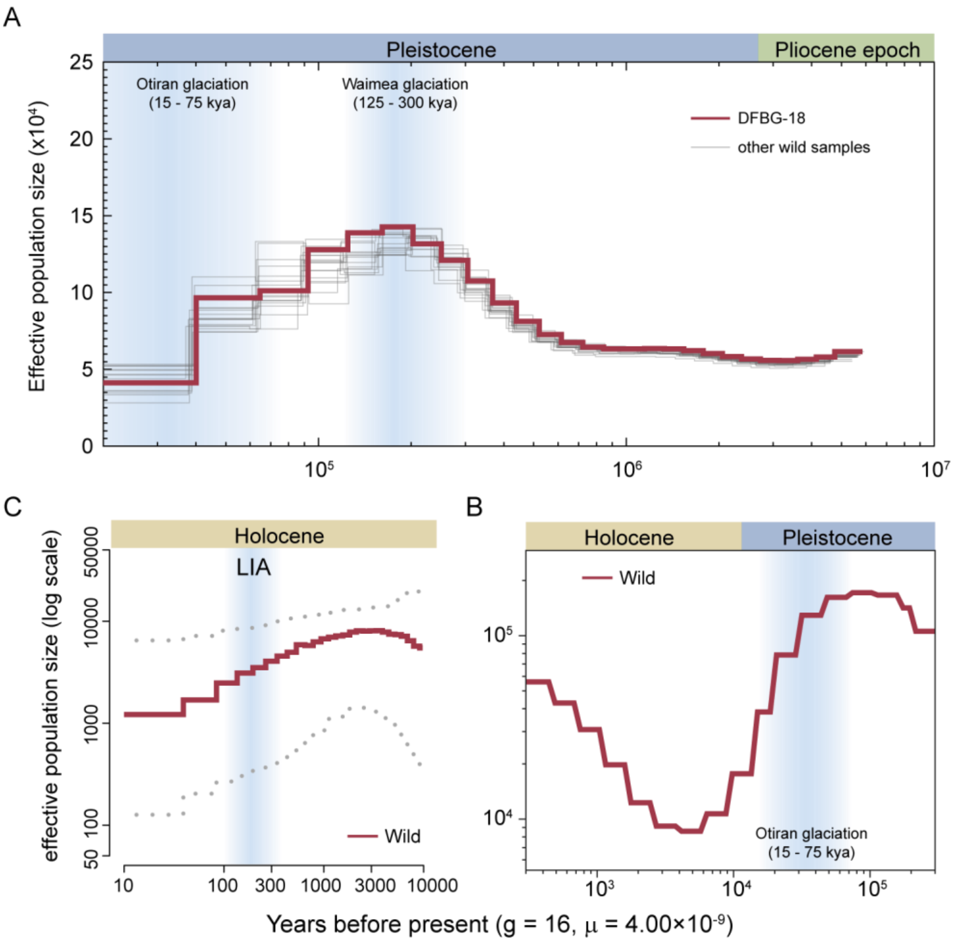

Figure 6: Estimated demographic history for the wild population of oriental stork. (A) Large-scale demographic fluctuation from 6 million years ago (Mya) to 20 ka BP inferred by PSMC for each wild individual. (B) Recent population history over the past 200-0.3 ka estimated by SMC++ with 29 wild individuals. (C) Recent effective population size for the wild inferred by PopSizeABC. Dotted lines indicate a 90% confidence interval. Light-blue shadows depict several glacial periods including Waimea glaciation, Otiran glaciation and the little ice age (LIA).

Discussion

Species are disappearing at an accelerated rate with the pervasive anthropogenic impacts [76, 77]. Record for the past 600 years reflected a peak of bird extinction rate in nineteenth century, firstly occurred at the Pacific islands [78], although avian is a relatively young clade undergoing fast substantial diversification[79]. It was long accepted that endangered species are easy to fall in the vortex of extinction because of small population size, high inbreeding and reduced fitness. Recent genomic studies provided new evidences that endangered or critically endangered species are not always doomed to extinction due to genetic factors[80]. In contract, LC ("least concern") species with abundance size nowadays may exhibit low genetic diversity and limited adaptive potential [81]. Such contradiction explained the necessity to introduce genomic parameters when assess the endangered status of a certain species. Genomic resources are crucial for understanding genetic basis of special biology characteristics[82, 83], genetic background and genetic risks of threatened species/populations [84-89]. Here we assembled a high-quality and chromosome-level genome for the oriental stork, filling the gaps to make targeted recovery plans to save this endangered stork. This assembly with 37 pseudo-chromosomes becomes a new representatively reference genome and provided a solid foundation for further evolutionary and genetic studies of the oriental stork, as well as for other stock species.

Billions of animals, including insects, birds, mammals, turtles and fish, migrate annually across the planet, in pursuit of improved foraging opportunities, safety and reproductive output[90]. Successful migration requires coordinated function of many traits, which involves brain function for visual, memory, navigation and social behavior, together with physiological conditions for ATP-production, lipid trafficking, innate immunity[64, 65, 91]. This biological phenomenon, thus, have an important but underappreciated evolutionary meaning in shaping species and populations, and ecosystems. Migratory characteristic is essential to the survival and recovery of wild oriental stork population. Specific phenotypes and physiological functions are often hypothesized to be attributed to the evolution of the

underlying protein-coding genes[92, 93]. Comparative genomic and population nature selection analyses for oriental stork provided important genetic cues for us to better understand their adaptation of the migration. We detected a series of expanded gene families, PSGs, REGs and candidate SNPs that related to long-term potentiation, photoreceptor cell, circadian rhythm, muscle development and energy metabolism, which may play important roles in learning and memory of migratory route, orientation and navigation, and continuous energy generation and supply during long-distance flight. These genomic signatures provide new insights into potential genomic basis and selective pressure on shaping the adaptive capability of migratory birds.

One important reason for its "endangered" status is that more than 95% individuals live in one subpopulation[14]. Our genetic structure analyses of the wild were consistent with the IUCN report with all samples restricted into a tight cluster, while the captive individuals were distinct from the wild individuals, with a much more scattered distribution in the cluster result. We infer those captive individuals have complex sources, further genetic drift and founder effect of different breeding lineages lead to a more-dispersed PCA result in captive individuals. In the contrary with its endangered status, high genomic heterozygosity and very low-level inbreeding (F_{ROH}) were found in both the wild and captive individuals, implying that the population decline hasn't caused severe genomic meltdown so far. We supposed three reasons to explain this phenomenon: (1) free mating without geographic boundaries helped to reserve the overall genetic diversity and avoid inbreeding; (2) the ancestral population of this species was large and no isolated small populations due to the situation in (1), genetic drift always acted on a relative large population, resulting in a slowly decrease of high diversity even though experiencing population decline; (3) no severe decline or bottleneck was observed in its population history. Genomic erosion often happens in populations that experienced long-term decline or strong bottleneck. It was evidenced in a critically endangered (CR) songbird, Yellow-breasted Bunting (*Emberiza aureola*), that only long-term population decline would result in low genetic diversity while a population undergoing recent rapid decline could still have high genetic diversity[94]. According

to the estimation of demographic history, oriental storks experienced a recovery over the past 10-3 ka BP and started to decrease again after 3 ka BP, approximately 187 generations(g) ago. This generation window was comparable with Yellow-breasted Bunting (100-200 g), much later than kākāpō (2,000 g), crested ibis (10,000 g), and brown eared pheasant (20,000 g). Therefore, the relatively short population declining history maybe another reason accounting for the remained high diversity in oriental storks. Potential risk accompanied with high nucleotide diversity was that deleterious derived alleles were abundant, especially in the wild population. Although most mutations were rare in population with low frequency, the population depression would be exacerbated by inbreeding and genetic drift if the wild population continues decreasing.

In summary, our results revealed a low extinction risk threatened by genetic factors and a high recovery potential of oriental stork, which brought great hope on "downlisted" from "EN" category. Based on the understanding of their evolutionary (migration for the oriental stork) and population genetic status, we emphasized the importance of protecting their original habitats and stopover sites to help natural recovery, and no requirement for captive-rearing and releasing, or genetic rescue by translocation. Consistently, thanks for the jointing efforts of Chinese and Russian governments, the wild oriental stork population size had risen to over 9,600 individuals in 2022 (unpublished data from the United Crane Conservation Committee of the China Wildlife Conservation Association) [95].This success on stork conservation and our genomic analysis together supported the priority of retaining and restoring habitats when saving species of short-term decline.

Conclusions

We assembled a high-quality chromosome-scale genome for the oriental stork, which provided a foundation to understand the biological traits and genetic status of this endangered bird. Comparative genomics highlights

comprehensive genomic signatures under evolution and selection in this species that related with migratory characteristics. Population genomic analysis reveals high genetic diversity and low inbreeding, which promise a possible population recovery in the future. Our study presented scientific and valuable genomic insights for global migratory bird protection to support the post-2020 biodiversity conservation framework.

Data Availability

The final genome assembly data, RNA-seq data and raw resequencing genome data are available in the NCBI BioProject repository (accession number: PRJNA1036389).

Additional Files

Supplementary Fig. S1. The distribution of 21-mer for estimation the genome size of oriental stork.

Supplementary Fig. S2. Heatmap of Hi-C chromosomal interaction density among all 37 chromosomes.

Supplementary Fig. S3. Comparison of gene characteristics of avian species and human.

Supplementary Fig. S4. Venn diagram representing the functional annotation of oriental stork gene set.

Supplementary Fig. S5. Comparison of the gene repertoires of 24 vertebrate genomes.

Supplementary Fig. S6. The expanded and contracted gene families in each bird genome and the comparison of their gene repertoires.

Supplementary Fig. S7. Comparison of detected genomic signals in oriental stork genome compared with unmigratory birds and migratory birds, including expanded families, positively selected genes and rapidly evolving

genes.

Supplementary Fig. S8. The phylogenetic tree of CRY2 gene constructed by the maximum likelihood method. The red clade represented three CRY2 gene in oriental stork.

Supplementary Fig. S9. Venn diagram for four methods to identify genes affected by candidate SNPs under recent selection.

Supplementary Fig. S10. Network plot of GO terms enriched by genes harbored selective SNPs.

Supplementary Fig. S11. Cross validation (CV) error in the ADMIXTURE analysis.

Supplementary Fig. S12. Nucleotide diversity (π) across 25 autosomes in wild and captive populations, respectively by sliding a 5-Mb window.

Supplementary Fig. S13. Statistics of the number of heterozygous and homozygous for synonymous, missense and LoF mutations in each individual.

Supplementary Fig. S14. Statistics of the frequency of heterozygous, homozygous and site for missense and LoF mutations, scaled by synonymous mutations.

Supplementary Table. S1. Statistics of sequencing data for genome assembly and gene annotation.

Supplementary Table. S2. Genomic statistics of the *C. boyciana* assembly.

Supplementary Table. S3. BUSCO analysis of the whole genome and the gene set of the *C. boyciana* genome.

Supplementary Table. S4. Statistics of sequencing data mapped to the *C. boyciana* genome which were used for the assembly and gene annotation.

Supplementary Table. S5. Transposable elements (TEs) statistics in our assembly.

Supplementary Table. S6. Statistics of identified Repeats by *De* novo method in *C. boyciana* genome.

Supplementary Table. S7. Transposable elements in the *C. boyciana* assembly.

Supplementary Table. S8. Statistics of annotations for the *C. boyciana* gene set.

Supplementary Table. S9. Statistics on functional annotation of the *C. boyciana* gene set.

Supplementary Table. S10. Statistics of ncRNA annotation.

Supplementary Table. S11. Expanded gene families in *C. boyciana* genome compared with unmigratory birds.

Supplementary Table. S12. Rapidly evolving genes in *C. boyciana* genome compared with unmigratory birds.

Supplementary Table. S13. Positively selected genes in *C. boyciana* genome compared with unmigratory birds.

Supplementary Table. S14. List of 46 oriental stork samples used for re-sequencing.

Supplementary Table. S15. GO enrichment of genes directly affected by positively selected SNPs.

Supplementary Table. S16. KEGG pathway enrichment of genes directly affected by positively selected SNPs.

Supplementary Table. S17. Whole-genome heterozygosity and F_{ROH} of 46 re-sequenced samples.

Supplementary Table. S18. Whole-genome heterozygosity for published birds presented in Fig. 4D.

Supplementary Table. S19. Average number of heterozygous, homozygous and all sites for derived alleles in wild and captive populations.

Abbreviations

IBA: Important Bird and Biodiversity Area; EAAF: East Asian-Australasian Flyway; IUCN: International Union for Conservation of Nature; ONT: Oxford Nanopore Technology; Hi-C: high-throughput chromosome

conformation capture; WGS: whole genome sequencing; Kb: kilobase pairs; BUSCO: Benchmarking Universal Single-Copy Orthologs; RNA-seq: RNA sequencing; BWA: Burrows-Wheeler aligner; GO: Gene ontology; KEGG: Kyoto Encyclopedia of Genes and Genomes; BLAST: Basic Local Alignment Search Tool; Chr: chromosome; PSG: positively selected gene; REG: rapidly evolving gene; gVCF: genomic Variant Call Format; SNP: single-nucleotide polymorphism; PCA: principal component analysis; iHS: integrated haplotype score; *H*: heterozygosity; Mb: megabase pairs; ROH: run of homozygosity; LoF: loss of function; PSMC: Pairwise Sequentially Markovian Coalescent; ABC: approximate Bayesian computation; MAF: minor allele frequency; Gb: gigabase pairs; LTR: long terminal repeat; LINE: long interspersed nuclear element; SINE: short interspersed nuclear element; *CRY2*: Cryptochrome Circadian Regulator 2; BP: before present; LIA: little ice age; NCBI: National Center for Biotechnology Information.

Competing Interests

The authors declare no competing interests.

Funding

This work was financially supported by funding from Surveillance of Wildlife Diseases from the State Forestry Administration of China (2023057) and the Leading Talent Project of "Science and Technology Leading Talent Team Project of Inner Mongolia Autonomous Region (2022LJRC0010).

Acknowledgments

We thank all staffs for their work in the collection of animal samples and the China National GeneBank for producing the sequencing data.

Authors' Contributions

T.L. and Z.H. conceived and designed the research. H.L., M.Z., S.W. and X.Z. organized and collected the samples.

M.S., L.H., M.Y., Y.L. and B.L. prepared the sequencing library. J.C. and H.L. performed genome assembly and annotation. S.Y., Y.L. and X.Z. conducted comparative genomic analysis and population genetic analysis. S.Y. wrote the manuscript. Y.X. and T.L. extensively revised the manuscript. T.L. and Z.H. supervised the study. All authors have read and approved the final manuscript.

References

1. Johnson CN, Balmford A, Brook BW, Buettel JC, Galetti M, Guangchun L, et al. Biodiversity losses and conservation responses in the Anthropocene. Science. 2017;356 6335:270-5. doi:doi:10.1126/science.aam9317.
2. Paez S, Kraus RHS, Shapiro B, Gilbert MTP, Jarvis ED, Group VGPC, et al. Reference genomes for conservation. 2022;377 6604:364-6. doi:doi:10.1126/science.abm8127.
3. Gregory RD, Noble D, Field R, Marchant J, Raven M and Gibbons D. Using birds as indicators of biodiversity. Ornis hungarica. 2003;12 13:11-24.
4. Kirby JS, Stattersfield AJ, Butchart SHM, Evans MI, Grimmett RFA, Jones VR, et al. Key conservation issues for migratory land- and waterbird species on the world's major flyways. Bird Conservation International. 2008;18 S1:S49-S73. doi:10.1017/S0959270908000439.
5. International B. State of the World's Birds 2018—taking the pulse of the planet. Cambridge, UK: BirdLife International. 2018.
6. Boere GC and Piersma T. Flyway protection and the predicament of our migrant birds: A critical look at international conservation policies and the Dutch Wadden Sea. Ocean & Coastal Management. 2012;68:157-68. doi:https://doi.org/10.1016/j.ocecoaman.2012.05.019.
7. Si Y, Xu F, Wei J, Zhang L, Murray N, Yang R, et al. A systematic network-based migratory bird monitoring and protection system is needed in China. Sci Bull (Beijing). 2021;66 10:955-7.
8. Ma Z, Melville DS, Liu J, Ying C, Yang H, Ren W, et al. Rethinking China's new great wall. Science. 2014;346 6212:912-4.
9. Li J, Hughes AC and Dudgeon D. Correction: Mapping wader biodiversity along the East Asian-Australasian flyway. PLoS One. 2019;14 4:e0215877. doi:10.1371/journal.pone.0215877.
10. Bamford M, Watkins D, Bancroft W, Tischler G and Wahl J. Migratory shorebirds of the East Asian-Australasian flyway : Population estimates and internationally important sites. . Canberra: Wetlands International, Oceania. 2008:pp 237.
11. Clark NA, Anderson GQA, Li J, Syroechkovskiy EE, Tomkovich PS, Zöckler C, et al. First formal estimate of the world population of the Critically Endangered spoon-billed sandpiper Calidris pygmaea. Oryx. 2018;52 1:137-46. doi:10.1017/S0030605316000806.
12. Zheng H, Shen G, Shang L, Lv X, Wang Q, McLaughlin N, et al. Efficacy of conservation strategies for endangered oriental white storks (Ciconia boyciana) under climate change in Northeast China. Biological Conservation. 2016;204:367-77. doi:https://doi.org/10.1016/j.biocon.2016.11.004.
13. Xu W, Xiao Y, Zhang J, Yang W, Zhang L, Hull V, et al. Reply to Yang et al.: Coastal wetlands are not

well represented by protected areas for endangered birds. PNAS. 2017;114 28:E5493-E. doi:doi:10.1073/pnas.1706515114.

14. International B. The IUCN Red List of Threatened Species 2018: e.T22697695A131942061. 2018; doi:https://dx.doi.org/10.2305/IUCN.UK.2018-2.RLTS.T22697695A131942061.en.

15. Garidi, Fan SJ, Cao L, Zhang BX, Wang YX, Zhu BG, et al. Migration strategy of the Bohai Bay wintering population of juvenile Oriental Storks (Ciconia boyciana). Biodiversity Science. 2022;30 5:21232. doi:10.17520/biods.2021232.

16. Van den Bossche W, Berthold P, Darman Y, Andronov V, Parilov M and Querner U. Satellite-tracking helps to discover stopover sites of the threatened Oriental White Stork (Ciconia boyciana). Microwave Telemetry, Inc Newsletter. 2001;2 1:3-4.

17. Lieberman-Aiden E, Berkum NV, Williams L, Imakaev M, Ragoczy T, Telling A, et al. Comprehensive Mapping of Long-Range Interactions Reveals Folding Principles of the Human Genome. Science. 2009;326 5950:289.

18. Lander ES and Waterman MS. Genomic mapping by fingerprinting random clones: a mathematical analysis. Genomics. 1988;2 3:231-9. doi:10.1016/0888-7543(88)90007-9.

19. Hu J, Fan J, Sun Z and Liu S. NextPolish: a fast and efficient genome polishing tool for long-read assembly. Bioinformatics. 2020;36 7:2253-5. doi:10.1093/bioinformatics/btz891.

20. Li H and Durbin R. Fast and accurate long-read alignment with Burrows–Wheeler transform. Bioinformatics. 2010;26 5:589-95. doi:10.1093/bioinformatics/btp698.

21. Durand NC, Shamim MS, Machol I, Rao SS, Huntley MH, Lander ES, et al. Juicer provides a one-click system for analyzing loop-resolution Hi-C experiments. Cell systems. 2016;3 1:95-8.

22. Manni M, Berkeley MR, Seppey M, Simão FA and Zdobnov EM. BUSCO Update: Novel and Streamlined Workflows along with Broader and Deeper Phylogenetic Coverage for Scoring of Eukaryotic, Prokaryotic, and Viral Genomes. Molecular biology and evolution. 2021;38 10:4647-54. doi:10.1093/molbev/msab199.

23. Xu Z and Wang H. LTR_FINDER: an efficient tool for the prediction of full-length LTR retrotransposons. Nucleic Acids Research. 2007;35 suppl_2:W265-W8. doi:10.1093/nar/gkm286 %J Nucleic Acids Research.

24. Han Y and Wessler SR. MITE-Hunter: a program for discovering miniature inverted-repeat transposable elements from genomic sequences. Nucleic Acids Research. 2010;38 22:e199-e. doi:10.1093/nar/gkq862 %J Nucleic Acids Research.

25. Flynn JM, Hubley R, Goubert C, Rosen J, Clark AG, Feschotte C, et al. RepeatModeler2 for automated genomic discovery of transposable element families. Proceedings of the National Academy of Sciences. 2020;117 17:9451-7.

26. Tarailo-Graovac M and Chen N. Using RepeatMasker to identify repetitive elements in genomic sequences. Current protocols in bioinformatics. 2009;Chapter 4:Unit 4.10. doi:10.1002/0471250953.bi0410s25.

27. Jurka J, Kapitonov VV, Pavlicek A, Klonowski P, Kohany O and Walichiewicz J. Repbase Update, a database of eukaryotic repetitive elements. Cytogenetic and genome research. 2005;110 1-4:462-7. doi:10.1159/000084979.

28. Benson G. Tandem repeats finder: a program to analyze DNA sequences. Nucleic acids research. 1999;27 2:573-80. doi:10.1093/nar/27.2.573.

29. Korf I. Gene finding in novel genomes. BMC Bioinformatics. 2004;5 1:59. doi:10.1186/1471-2105-5-59.

30. Majoros WH, Pertea M and Salzberg SL. TigrScan and GlimmerHMM: two open source ab initio

eukaryotic gene-finders. Bioinformatics. 2004;20 16:2878-9. doi:10.1093/bioinformatics/bth315 %J Bioinformatics.

31. Keller O, Kollmar M, Stanke M and Waack S. A novel hybrid gene prediction method employing protein multiple sequence alignments. Bioinformatics. 2011;27 6:757-63. doi:10.1093/bioinformatics/btr010.

32. Bolger AM, Lohse M and Usadel B. Trimmomatic: a flexible trimmer for Illumina sequence data. Bioinformatics. 2014;30 15:2114-20. doi:10.1093/bioinformatics/btu170 %J Bioinformatics.

33. Haas BJ, Papanicolaou A, Yassour M, Grabherr M, Blood PD, Bowden J, et al. De novo transcript sequence reconstruction from RNA-seq using the Trinity platform for reference generation and analysis. Nature Protocols. 2013;8 8:1494-512. doi:10.1038/nprot.2013.084.

34. Haas BJ, Salzberg SL, Zhu W, Pertea M, Allen JE, Orvis J, et al. Automated eukaryotic gene structure annotation using EVidenceModeler and the Program to Assemble Spliced Alignments. Genome Biology. 2008;9 1:R7-R22. doi:10.1186/gb-2008-9-1-r7.

35. Mount DW. Using the Basic Local Alignment Search Tool (BLAST). CSH protocols. 2007;2007:pdb.top17. doi:10.1101/pdb.top17.

36. Birney E, Clamp M and Durbin R. GeneWise and Genomewise. Genome research. 2004;14 5:988-95. doi:10.1101/gr.1865504.

37. Campbell MS, Holt C, Moore B and Yandell M. Genome Annotation and Curation Using MAKER and MAKER-P. Current Protocols Bioinformatics. 2014;48 1:4.11.1-4..39. doi:https://doi.org/10.1002/0471250953.bi0411s48.

38. Altschul SF, Gish W, Miller W, Myers EW and Lipman DJ. Basic local alignment search tool. Journal of molecular biology. 1990;215 3:403-10.

39. Wang Y, Tang H, Debarry JD, Tan X, Li J, Wang X, et al. MCScanX: a toolkit for detection and evolutionary analysis of gene synteny and collinearity. Nucleic acids research. 2012;40 7:e49. doi:10.1093/nar/gkr1293.

40. Krzywinski M, Schein J, Birol I, Connors J, Gascoyne R, Horsman D, et al. Circos: An information aesthetic for comparative genomics. Genome Research. 2009;19:1639-45.

41. Lam-Tung N, Schmidt HA, Arndt VH, Quang MB and Evolution. IQ-TREE: A Fast and Effective Stochastic Algorithm for Estimating Maximum-Likelihood Phylogenies. Molecular Biology and Evolution. 2015;32 1:268-74.

42. Yang Z. PAML 4: Phylogenetic Analysis by Maximum Likelihood. Molecular Biology and Evolution. 2007;24 8:1586–91.

43. Benton MJ and Donoghue PC. Paleontological evidence to date the tree of life. Molecular biology and evolution. 2007;24 1:26-53. doi:10.1093/molbev/msl150.

44. Li H, Coghlan A, Ruan J, Coin LJ, Hériché J-K, Osmotherly L, et al. TreeFam: a curated database of phylogenetic trees of animal gene families. Nucleic Acids Research. 2006;34 suppl_1:D572-D80. doi:10.1093/nar/gkj118 %J Nucleic Acids Research.

45. De Bie T, Cristianini N, Demuth JP and Hahn MW. CAFE: a computational tool for the study of gene family evolution. Bioinformatics. 2006;22 10:1269-71. doi:10.1093/bioinformatics/btl097 %J Bioinformatics.

46. Team RDC. R: A language and environment for statistical computing. R Foundation for Statistical Computing. 2012.

47. Wu T, Hu E, Xu S, Chen M, Guo P, Dai Z, et al. clusterProfiler 4.0: A universal enrichment tool for interpreting omics data. Innovation (Camb). 2021;2 3:100141. doi:10.1016/j.xinn.2021.100141.

48. Supek F, Bošnjak M, Škunca N and Šmuc T. REVIGO summarizes and visualizes long lists of gene

ontology terms. PLoS One. 2011;6 7:e21800. doi:10.1371/journal.pone.0021800.

49. Freed D, Aldana R, Weber JA and Edwards JS. The Sentieon Genomics Tools - A fast and accurate solution to variant calling from next-generation sequence data. bioRxiv. 2017:115717. doi:10.1101/115717 %J bioRxiv.

50. Danecek P, Auton A, Abecasis G, Albers CA, Banks E, DePristo MA, et al. The variant call format and VCFtools. Bioinformatics. 2011;27 15:2156-8. doi:10.1093/bioinformatics/btr330.

51. Chang CC, Chow CC, Tellier LC, Vattikuti S, Purcell SM and Lee JJ. Second-generation PLINK: rising to the challenge of larger and richer datasets. Gigascience. 2015;4:7. doi:10.1186/s13742-015-0047-8.

52. Alexander DH, Novembre J and Lange K. Fast model-based estimation of ancestry in unrelated individuals. Genome Research. 2009;19 9:1655-64.

53. Browning BL, Zhou Y and Browning SR. A One-Penny Imputed Genome from Next-Generation Reference Panels. The American Journal of Human Genetics. 2018;103 3:338-48. doi:10.1016/j.ajhg.2018.07.015.

54. Voight BF, Kudaravalli S, Wen X and Pritchard JK. A map of recent positive selection in the human genome. PLoS Biology. 2006;4 3:e72. doi:10.1371/journal.pbio.0040072.

55. Zhou Y, Zhou B, Pache L, Chang M, Khodabakhshi AH, Tanaseichuk O, et al. Metascape provides a biologist-oriented resource for the analysis of systems-level datasets. Nature communications. 2019;10 1:1523. doi:10.1038/s41467-019-09234-6.

56. Gautier M and Vitalis R. rehh: an R package to detect footprints of selection in genome-wide SNP data from haplotype structure. Bioinformatics. 2012;28 8:1176-7. doi:10.1093/bioinformatics/bts115.

57. Khan A, Patel K, Shukla H, Viswanathan A, van der Valk T, Borthakur U, et al. Genomic evidence for inbreeding depression and purging of deleterious genetic variation in Indian tigers. 2021;118 49:e2023018118. doi:doi:10.1073/pnas.2023018118.

58. Li H, Handsaker B, Wysoker A, Fennell T, Ruan J, Homer N, et al. The Sequence Alignment/Map format and SAMtools. Bioinformatics. 2009;25 16:2078-9. doi:10.1093/bioinformatics/btp352.

59. Feng S, Fang Q, Barnett R, Li C, Han S, Kuhlwilm M, et al. The Genomic Footprints of the Fall and Recovery of the Crested Ibis. Current Biology. 2019;29:340-9. doi:10.1016/j.cub.2018.12.008.

60. Li H and Durbin R. Inference of human population history from individual whole-genome sequences. Nature. 2011;475 7357:493-6. doi:10.1038/nature10231.

61. Terhorst J, Kamm JA and Song YS. Robust and scalable inference of population history from hundreds of unphased whole genomes. Nature Genetics. 2016;49 2:303-9. doi:10.1038/ng.3748.

62. Boitard S, Rodriguez W, Jay F, Mona S and Austerlitz F. Inferring Population Size History from Large Samples of Genome-Wide Molecular Data - An Approximate Bayesian Computation Approach. PLoS Genetics. 2016;12 3:e1005877. doi:10.1371/journal.pgen.1005877.

63. Kishkinev D, Chernetsov N, Heyers D and Mouritsen H. Migratory reed warblers need intact trigeminal nerves to correct for a 1,000 km eastward displacement. PLoS One. 2013;8 6:e65847.

64. Xu J, Jarocha LE, Zollitsch T, Konowalczyk M, Henbest KB, Richert S, et al. Magnetic sensitivity of cryptochrome 4 from a migratory songbird. Nature. 2021;594 7864:535-40. doi:10.1038/s41586-021-03618-9.

65. Gu Z, Pan S, Lin Z, Hu L, Dai X, Chang J, et al. Climate-driven flyway changes and memory-based long-distance migration. Nature. 2021;591 7849:259-64. doi:10.1038/s41586-021-03265-0.

66. Zhang T, Chen P, Li W, Sha S, Wang Y, Yuan Z, et al. Cognitive deficits in mice lacking Nsun5, a cytosine-5 RNA methyltransferase, with impairment of oligodendrocyte precursor cells. Glia. 2019;67 4:688-702. doi:https://doi.org/10.1002/glia.23565.

67. Zhang J, Wang Y, Chi Z, Keuss MJ, Pai YM, Kang HC, et al. The AAA+ ATPase Thorase regulates AMPA receptor-dependent synaptic plasticity and behavior. Cell. 2011;145 2:284-99. doi:10.1016/j.cell.2011.03.016.

68. Cassens RG and Cooper CC. Red and White Muscle. In: Chichester CO, Mrak EM and Stewart GF, editors. Advances in Food Research. Academic Press; 1971. p. 1-74.

69. Barge L and Mark EW. Muscle fiber types in a migratory and a non-magratory avian species. In: 2012.

70. Zhan X, Pan S, Wang J, Dixon A, He J, Muller MG, et al. Peregrine and saker falcon genome sequences provide insights into evolution of a predatory lifestyle. Nature Genetics. 2013;45 5:563-6. doi:10.1038/ng.2588.

71. von Seth J, van der Valk T, Lord E, Sigeman H, Olsen R-A, Knapp M, et al. Genomic trajectories of a near-extinction event in the Chatham Island black robin. BMC Genomics. 2022;23 1:747. doi:10.1186/s12864-022-08963-1.

72. Dussex N, van der Valk T, Morales HE, Wheat CW, Díez-del-Molino D, von Seth J, et al. Population genomics of the critically endangered kākāpō. Cell Genomics. 2021;1 doi:10.1016/j.xgen.2021.100002.

73. Wang P, Burley JT, Liu Y, Chang J, Chen, Lu Q, et al. Genomic Consequences of Long-Term Population Decline in Brown Eared Pheasant. Molecular Biology and Evolution. 2021;38 1:263-73. doi:10.1093/molbev/msaa213.

74. Seth Jv, Dussex N, Diez-Del-Molino D, van der Valk T, Kutschera VE, Kierczak M, et al. Genomic insights into the conservation status of the world's last remaining Sumatran rhinoceros populations. Nature Communications. 2021;12:2393. doi:10.1038/s41467-021-22386-8.

75. Yang S, Lan T, Zhang Y, Wang Q, Li H, Dussex N, et al. Genomic investigation of the Chinese alligator reveals wild-extinct genetic diversity and genomic consequences of their continuous decline. Molecular Ecology Resources. 2022:1-18. doi:https://doi.org/10.1111/1755-0998.13702.

76. Di Marco M, Venter O, Possingham HP and Watson JEM. Changes in human footprint drive changes in species extinction risk. Nature Communications. 2018;9 1:4621. doi:10.1038/s41467-018-07049-5.

77. Pimm SL, Jenkins CN, Abell R, Brooks TM, Gittleman JL, Joppa LN, et al. The biodiversity of species and their rates of extinction, distribution, and protection. Science. 2014;344 6187:1246752. doi:doi:10.1126/science.1246752.

78. Lees AC, Haskell L, Allinson T, Bezeng SB, Burfield IJ, Renjifo LM, et al. State of the World's Birds. Annual Review of Environment and Resources. 2022;47 1:231-60. doi:10.1146/annurev-environ-112420-014642.

79. Green RE, Braun EL, Armstrong J, Earl D, Nguyen N, Hickey G, et al. Three crocodilian genomes reveal ancestral patterns of evolution among archosaurs. Science. 2014;346 6215:1254449. doi:10.1126/science.1254449.

80. Robinson JA, Kyriazis CC, Nigenda-Morales SF, Beichman AC, Rojas-Bracho L, Robertson KM, et al. The critically endangered vaquita is not doomed to extinction by inbreeding depression. Science. 2022;376 6593:635-9. doi:doi:10.1126/science.abm1742.

81. Westbury MV, Petersen B, Garde E, Heide-Jørgensen MP and Lorenzen ED. Narwhal Genome Reveals Long-Term Low Genetic Diversity despite Current Large Abundance Size. iScience. 2019;15:592-9. doi:10.1016/j.isci.2019.03.023.

82. Guang X, Lan T, Wan Q-H, Huang Y, Li H, Zhang M, et al. Chromosome-scale genomes provide new insights into subspecies divergence and evolutionary characteristics of the giant panda. Sci Bull (Beijing). 2021;66 19:2002-13. doi:https://doi.org/10.1016/j.scib.2021.02.002.

83. Lan T, Li H, Yang S, Shi M, Han L, Sahu SK, et al. The chromosome-scale genome of the raccoon dog:

Insights into its evolutionary characteristics. iScience. 2022;25 10:105117. doi:10.1016/j.isci.2022.105117.

84. Lan T, Li H, Zhang L, Shi M, Liu B, Cui L, et al. Population genomics reveals extensive inbreeding and purging of mutational load in wild Amur tigers. bioRxiv. 2023; doi:10.1101/2023.05.09.539923.

85. Zhang L, Lan T, Lin C, Fu W, Yuan Y, Lin K, et al. Chromosome-scale genomes reveal genomic consequences of inbreeding in the South China tiger: A comparative study with the Amur tiger. Molecular ecology resources. 2022; doi:10.1111/1755-0998.13669.

86. Yang S, Lan T, Zhang Y, Wang Q, Li H, Dussex N, et al. Genomic investigation of the Chinese alligator reveals wild-extinct genetic diversity and genomic consequences of their continuous decline. Molecular ecology resources. 2022; doi:10.1111/1755-0998.13702.

87. Wang Q, Lan T, Li H, Sahu SK, Shi M, Zhu Y, et al. Whole-genome resequencing of Chinese pangolins reveals a population structure and provides insights into their conservation. Communications biology. 2022;5 1:821. doi:10.1038/s42003-022-03757-3.

88. Dussex N, van der Valk T, Morales HE, Wheat CW, Díez-del-Molino D, von Seth J, et al. Population genomics of the critically endangered kākāpō. Cell Genomics. 2021;1 1:100002. doi:10.1016/j.xgen.2021.100002.

89. Khan A, Patel K, Shukla H, Viswanathan A, van der Valk T, Borthakur U, et al. Genomic evidence for inbreeding depression and purging of deleterious genetic variation in Indian tigers. Proceedings of the National Academy of Sciences of the United States of America. 2021;118 49 doi:10.1073/pnas.2023018118.

90. Bauer S and Hoye BJ. Migratory animals couple biodiversity and ecosystem functioning worldwide. Science. 2014;344 6179:1242552. doi:10.1126/science.1242552.

91. Flack A, Aikens EO, Kölzsch A, Nourani E, Snell KRS, Fiedler W, et al. New frontiers in bird migration research. Current biology. 2022;32 20:R1187-R99. doi:10.1016/j.cub.2022.08.028.

92. Shao Y, Wang X-B, Zhang J-J, Li M-L, Wu S-S, Ma X-Y, et al. Genome and single-cell RNA-sequencing of the earthworm Eisenia andrei identifies cellular mechanisms underlying regeneration. Nature Communications. 2020;11 1:2656. doi:10.1038/s41467-020-16454-8.

93. Cole TL, Zhou C, Fang M, Pan H, Ksepka DT, Fiddaman SR, et al. Genomic insights into the secondary aquatic transition of penguins. Nature Communications. 2022;13 1:3912. doi:10.1038/s41467-022-31508-9.

94. Wang P, Hou R, Wu Y, Zhang Z, Que P and Chen P. Genomic status of yellow-breasted bunting following recent rapid population decline. iScience. 2022;25 7:104501. doi:https://doi.org/10.1016/j.isci.2022.104501.

95. Yang Z, Chen L, Jia R, Xu H, Wang Y, Wei X, et al. Migration routes of the endangered Oriental Stork (Ciconia boyciana) from Xingkai Lake, China, and their repeatability as revealed by GPS tracking. Avian Research. 2023;14:100090. doi:https://doi.org/10.1016/j.avrs.2023.100090.

Figure
Click here to access/download;Figure;Figure1-1102.pdf

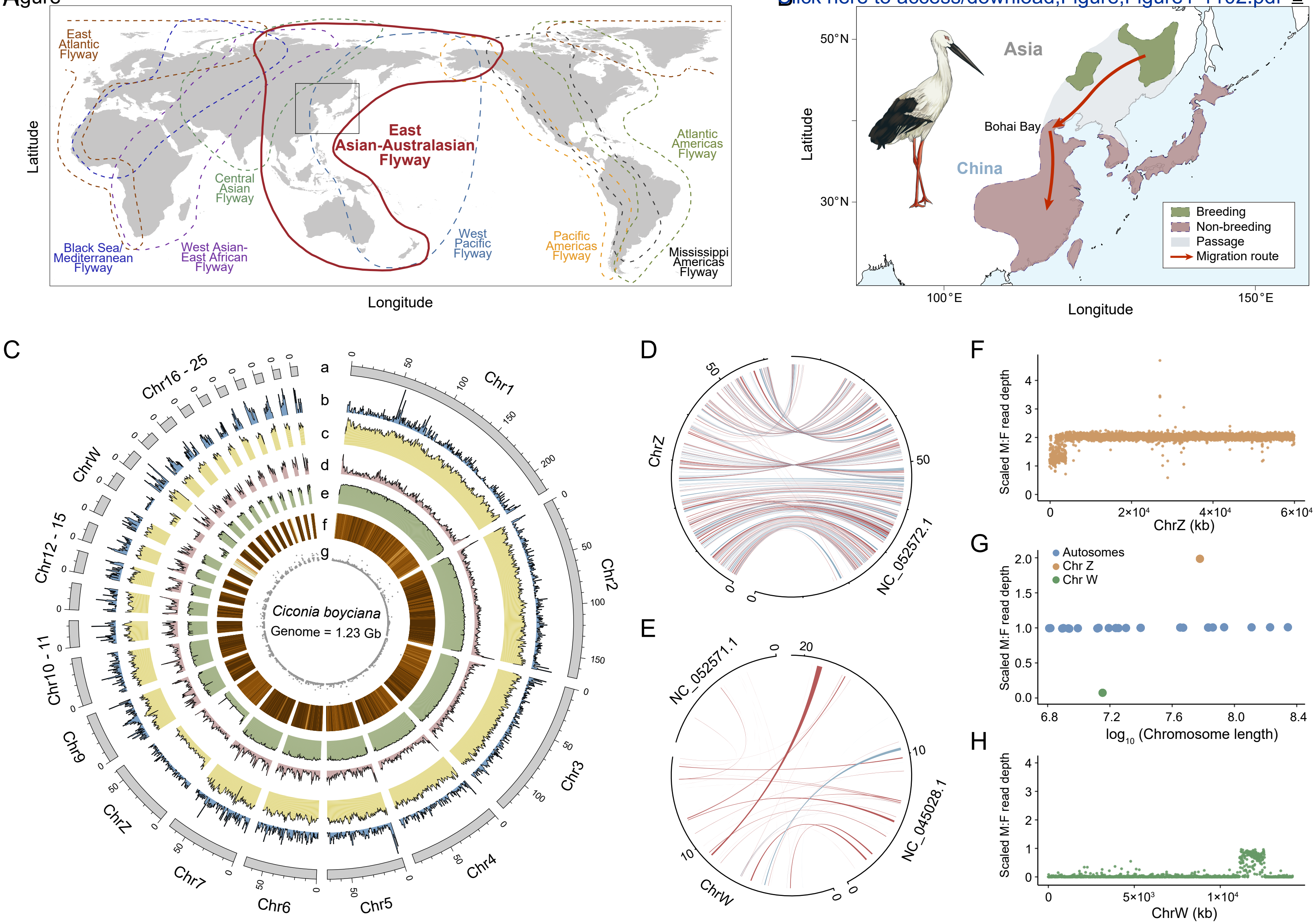

Figure
Click here to access/download;Figure;Figure2.pdf

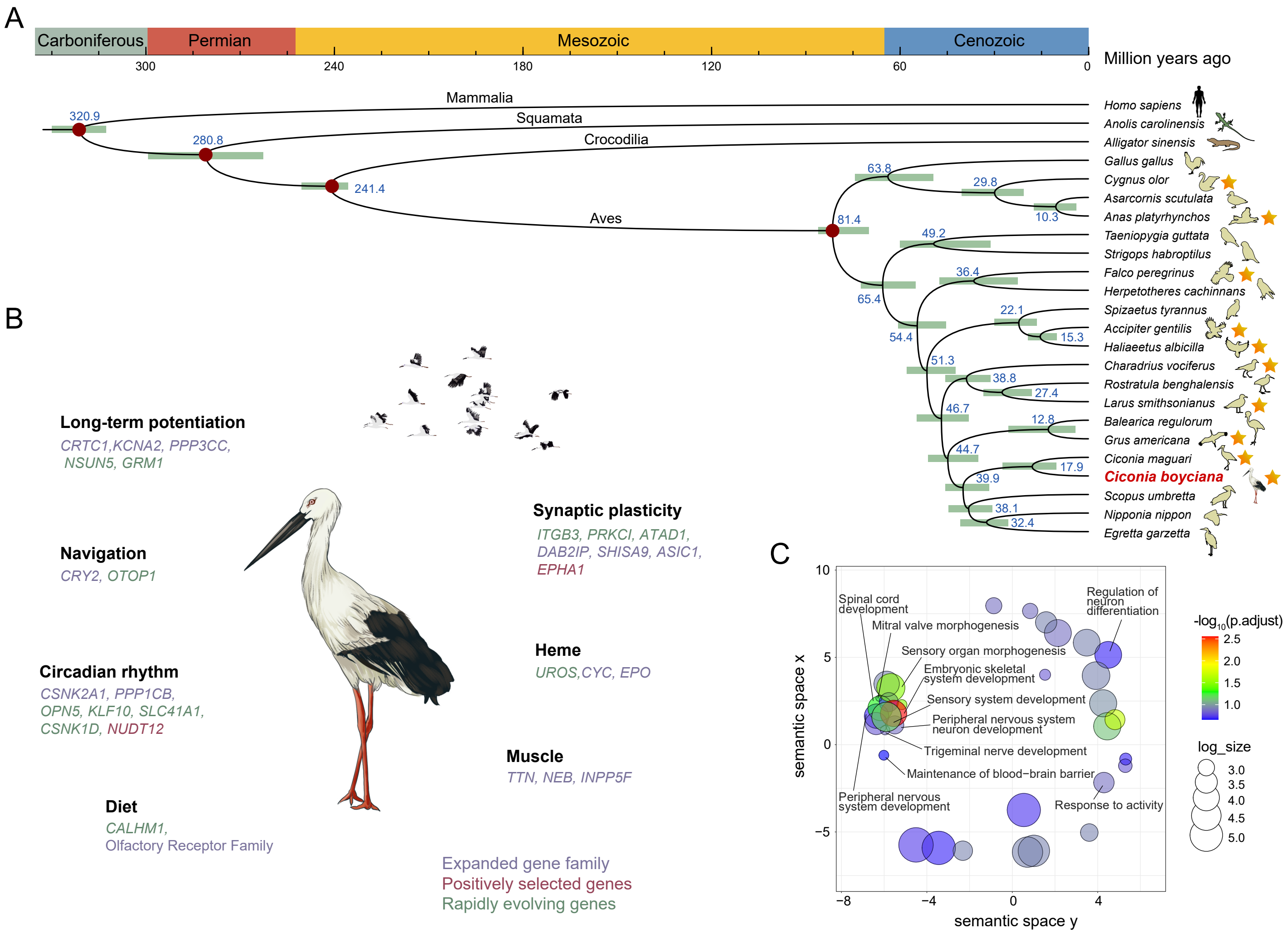

Figure

Click here to access/download;Figure;Figure3.pdf

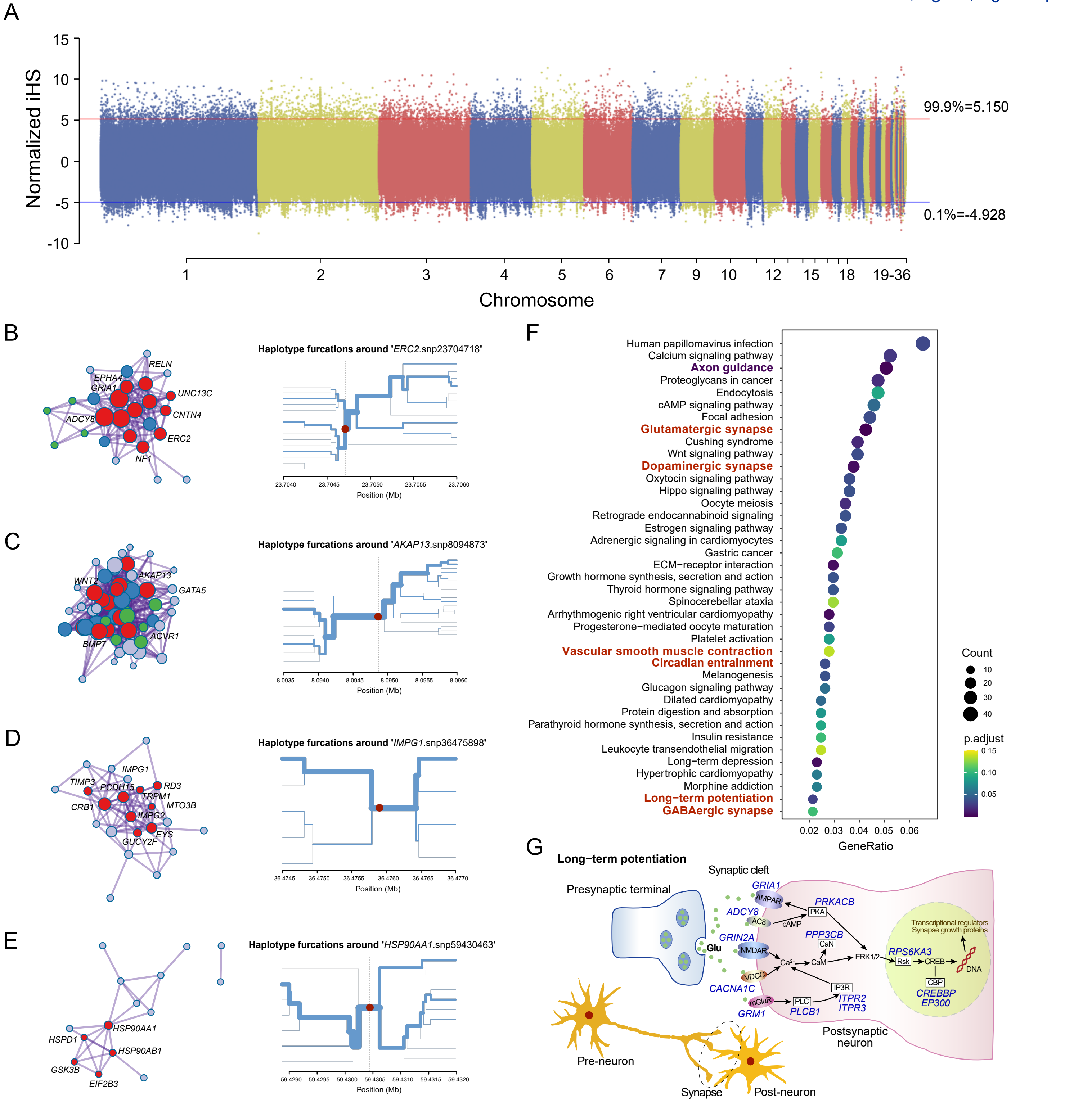

Figure

Click here to access/download;Figure;Figure4.pdf

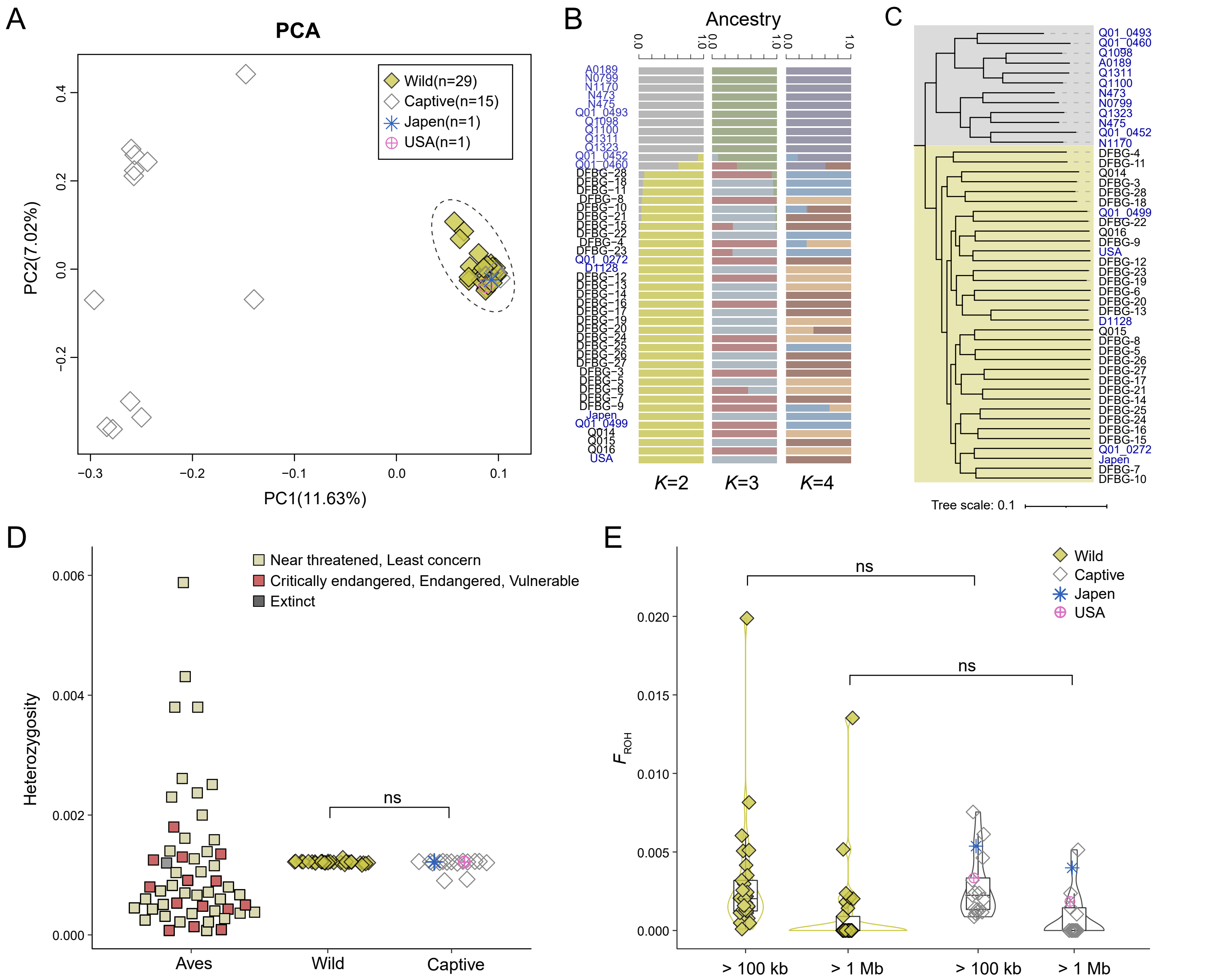

Figure Click here to access/download;Figure;Figure5.pdf

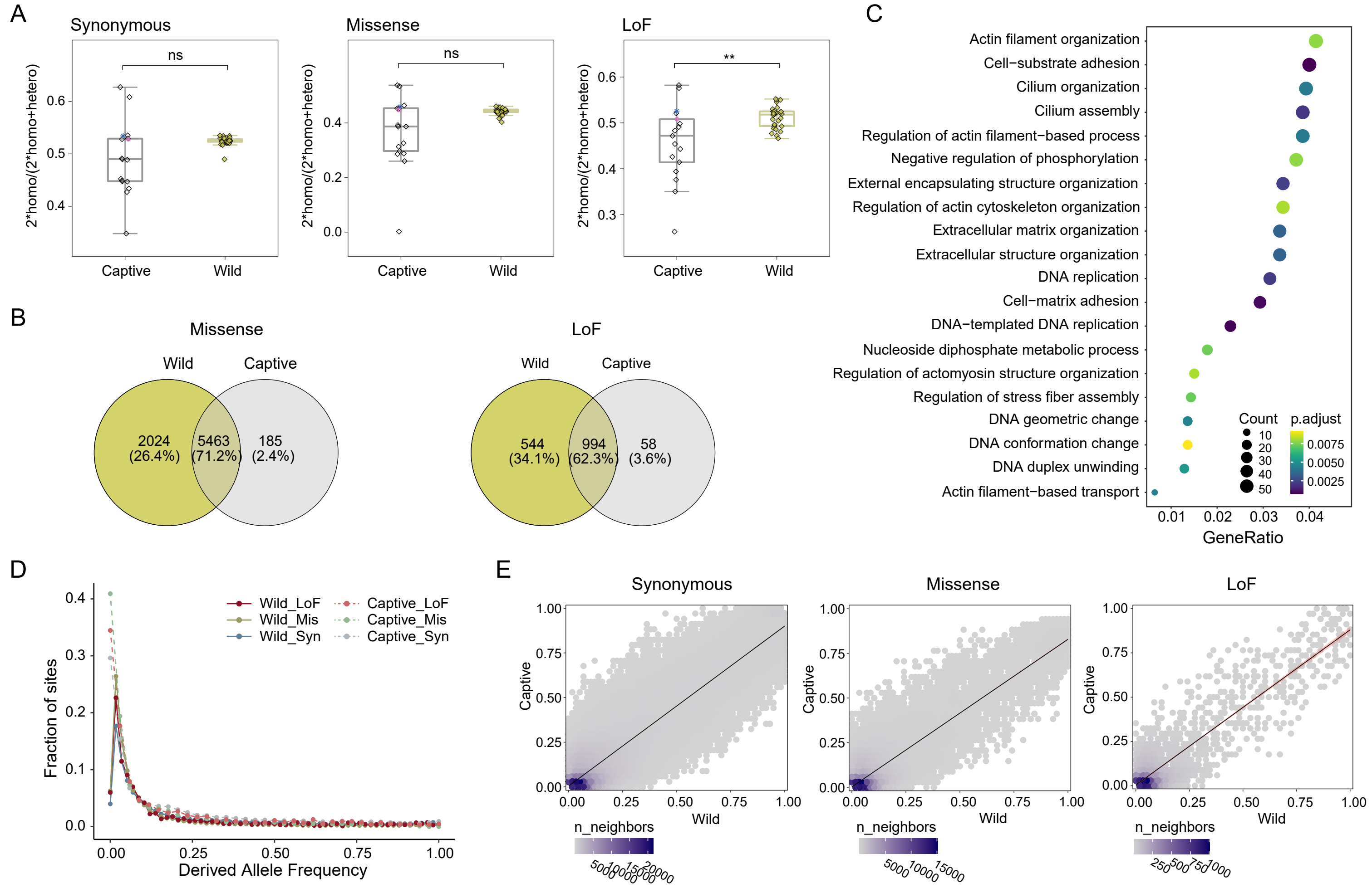

Figure

Click here to access/download;Figure;Figure6.pdf

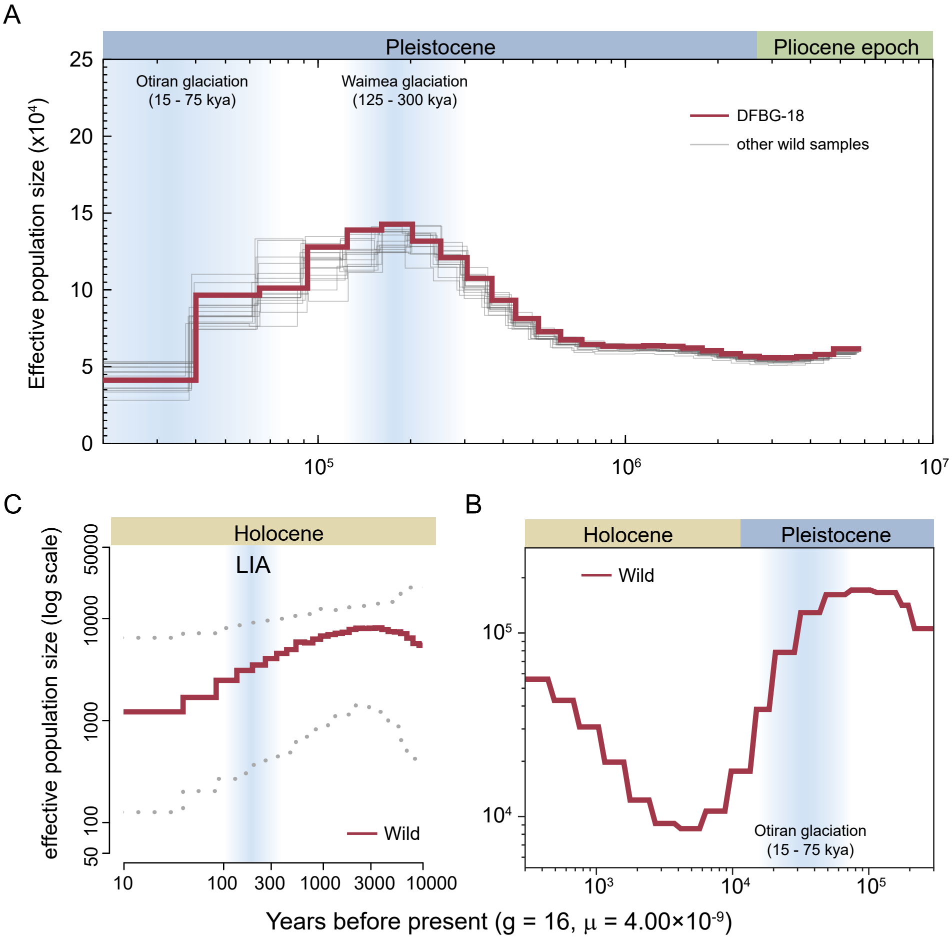

Click here to access/download
Supplementary Material
Supplementary-tables.xlsx

Click here to access/download
Supplementary Material
Supplementary-Figures.docx

Cover letter
Click here to access/download;Personal Cover;Cover letter-1106.docx

November 06, 2023

Dr. Scott Edmunds
Editor-in-Chief
GigaScience

Dear Editor:

We hereby submit a manuscript entitled "**Genomic exploration of the endangered oriental stork, *Ciconia boyciana*, shed lights on migration adaptation and future conservation**" to be considered for publication in ***GigaScience***.

The decline of East Asian-Australasian Flyway (EAAF) seriously impacts the function and service of ecosystem due to human-bird conflict and climate change. Genomic resources and comprehensive assessment of endangered birds in EAAF are almost vacant, bringing difficulties in understanding their true threatened status and taking further conservation actions. Here, we focus on the well-known endangered migratory oriental stork, *Ciconia boyciana*, filling species and population-level genomic data gaps to benefit future bird protection. We present a high-quality chromosome-level genome assembly of the oriental stork and successfully identify Z and W chromosomes. We find a series of genomic signals related to the migratory trait, which imply an integrate work of brain synapses, photoreceptor cell, circadian rhythm, muscle tissue and energy metabolism. We also collect samples from wild and captive individuals and find that in fact oriental storks have a relative-high genetic diversity and low inbreeding level while wild population have a higher mutational load than captive-born animals. Their short-term decline hasn't resulted in serious genomic consequences till now and propose natural recovery hope.

I am authorized on behalf of all the authors of this article to confirm that no author has any conflict of interest to disclose. All authors have approved the version submitted for publication. The work in this article is original and has not been published previously, and the article is not under consideration by any other journal. We have discussed this manuscript with **Scott Edmunds and Hongling Zhou**, this July in BGI center. We thank you for your kind consideration of our article. Looking forward to hearing from you.

Sincerely,

Zhijun Hou, PhD.

Professor

College of Wildlife and Protected Area

Northeast Forestry University

Email: houzhijundb@163.com
