# Supplementary material for: Genomic exploration of the endangered oriental stork, Ciconia boyciana, sheds light on migration adaptation and future conservation
Source: Gigascience. 2024 Oct 22;13:giae081. doi: 10.1093/gigascience/giae081 (PMC11494145; doi:10.1093/gigascience/giae081)
Supplement: giae081_GIGA-D-23-00340_Revision_1 [file giae081_giga-d-23-00340_revision_1.pdf]

GigaScience

Genomic exploration of the endangered oriental stork, Ciconia boyciana, shed lights on migration adaptation and future conservation

--Manuscript Draft--

Manuscript Number:	GIGA-D-23-00340R1
Full Title:	Genomic exploration of the endangered oriental stork, Ciconia boyciana, shed lights on migration adaptation and future conservation
Article Type:	Research
Funding Information:	Surveillance of Wildlife Diseases from the State Forestry Administration of China (2023057) — Not applicable<br>Leading Talent Project of “Science and Technology Leading Talent Team Project of Inner Mongolia Autonomous Region (2022LJRC0010) — Not applicable
Abstract:	Abstract<br>Background: The oriental stork, Ciconia boyciana, is an endangered migratory bird on the IUCN Red List. They experienced a rapidly decline in the past decades, with nest locations and stop-over sites largely degraded due to human-bird conflicts. The genome-wide genetic status of this threatened bird population is critical to make future conservation strategies but lack of investigation.<br>Findings: We presented the first chromosome-scale genome for the oriental stork with high quality, contiguity, and accuracy. The assembled genome size was 1.24 Gb with a scaffold N50 of 103 Mb, and 1.23 Gb contigs (99.32%) were anchored to 35 chromosomes. We didn't find a genetic structure in the wild population. Genome-wide genetic diversity (π = 0.0012) of the oriental stork was at a moderate to high level among threatened bird species and inbreeding risk was not concerning (FROH = 5.56±5.30%). Demographic history reconstruction indicated a rapid recent decline likely driven by human activities. Genomic selective signals of the migratory trait were identified in genes related to the long-term potentiation, photoreceptor cell, circadian rhythm, muscle development and energy metabolism, indicating the essential interplay between genetic and ecological adaptation.<br>Conclusions: The first chromosome-scale genome of the oriental stork expands genomic resource of endangered birds, providing a genomic basis for understanding its genetic background, extinction risk and the migratory characteristic, which will further facilitate the decision of future conservation plans for such a migratory bird.<br>Keywords: oriental stork, comparative genome, population genetics, endangered species, migration
Corresponding Author:	Shangchen Yang<br>Zhejiang University<br>Hangzhou City, CHINA
Corresponding Author Secondary Information:	
Corresponding Author's Institution:	Zhejiang University
Corresponding Author's Secondary Institution:	
First Author:	Shangchen Yang
First Author Secondary Information:	
Order of Authors:	Shangchen Yang
	Yan Liu
	Xiaoqing Zhao
	Jin Chen
	Haimeng Li

Powered by Editorial Manager® and ProduXion Manager® from Aries Systems Corporation

	Hongrui Liang
	Mengchao Zhou
	Shiqing Wang
	Xiaotian Zhang
	Minhui Shi
	Lei Han
	Mingyuan Yu
	Yaxian Lu
	Boyang Liu
	Yu Xu
	Tianming Lan
	Zhijun Hou
**Order of Authors Secondary Information:**	
**Response to Reviewers:**	Response to the editor:<br>Thank you very much for giving us another chance to submit our revised manuscript. We appreciate the valuable comments and suggestions from the two reviewers, which grasped the main points and improved our study so much. We have prepared a point-by-point response as follows and extensively revised our manuscript according to the points reviewers concerned. We hope this revised manuscript could satisfy the reviewers and we are looking forward to your further information.<br><br>Reviewer reports:<br><br>Reviewer #1: minor<br><br>Response to reviewer 1: Thank you very much for the detailed suggestions you proposed. These comments are very important, like introducing the population dynamic of the wild population. We believe our manuscript is clearer after this revision. Thank you.<br><br>Question 1. Line 57, I don't think it is a good idea to mention critically endangered spoon-billed sandpiper Calidris pygmaea here, it makes it easy to think that that's a species worth more attention than your bird, Ciconia boyciana.<br><br>Response: Thank you for pointing out this issue, we agree. We selected other bird species with less endangered status than the Ciconia boyciana and reorganized the description to highlight the bird in our study. Please see Line 56-58.<br><br>Question 2. Line 65, what about the population number before 2018? maybe you can use decades of data to show us how the species is changing, as you mention in Line 71 "such decline".<br><br>Response: Yes, you are right, thanks for your suggestion. It is very important to record the wild population size for endangered species conservation. We first discussed the wild population with scientists of Northeast Forestry University who are working on field investigation, but they told us the information of historical population dynamics over the recent decades are very limited. We then searched the literatures for answers: Oriental storks were widely distributed in Northeast Asia in the history, while in 1868-1935, wild individuals began to decrease[1]. In 1970s, wild populations in Japan and Korea were extinct. And the breeding areas in the Russian Far East, Heilongjiang and Jilin Provinces also became narrow. In 1960s, there were more than 1000 oriental storks breeding in Heilongjiang Province. However, this number decreased to 123 in 1986 and less than 50 in 1990. In late 1980s, there were no storks breeding in Jilin Province[2, 3]. The population size is estimated at 3,000 individuals from 1990s-2010s [4].

However, we did not find continuous yearly records for the overall number of wild oriental storks worldwide. We added more detailed description about the threatened state of oriental stork. Please see Line 68-71.

Question 3. Line 87, the sample? we cannot get the detail information for individual of the reference genome assembly, is it captive-born or rescued wild one? Furth more, the detail information about individuals were listed in Line 167-168, maybe it can be removed to "Samples and ethics statement" part

Response: Thank you, yes, it would be much better if we clarify the sample information used in this study in the Method part. The individual for assembly is a captive-born oriental stork (sample ID: N1170) collected in Harbin North Forest Zoo. In the revised version, we added this information, please see Line 88. And we also moved the description of re-sequenced individuals from Line 167-168 to the "Samples and ethics statement" part, please see Line 93-95.

Question 4. Line 134 Identification of sex-linked regions, I am not sure what is the significance of this part of the analysis? it is interesting to see the phylogeny reconstruction and demographic dynamics, maybe the results (270-276) can be less?

Response: Thank you, we simplified the results of sex-linked regions, please see Line 256-258. Sex chromosomes are relative difficult to be assembled compared to autosomes, because Z and W chromosomes have more complex regions and a large number of repetitive sequences regions. However, accurate identification of sex chromosomes is very important, and many analyses of population re-sequencing need remove the variants on the sex chromosomes, including genetic structure, inbreeding, genetic diversity and demographic dynamics. Some parameters of sex chromosomes are different from autosomes such as selection stress, recombination rate, …. In our practice, especially for large sex chromosomes, SNPs on them would substantially change the genetic structure results. Also, the sex chromosomes could be used in other studies related with sex determination and reproduction. So, this is a very basic but may be valuable genomic resource for other people to cite.
In this study, we identified sex chromosomes and remove these sex-linked regions when carrying out population genomic analysis to avoid bias. As your comment, we simplified the description of the results and moved the figures (previous Fig. 1D-H) to the supplementary materials (Supplementary Fig. S3), please see Line 241-244.

Question 5. Line 295-299, the authors compared the expanded gene families/ PSGs, and REGs in migratory and non-migratory birds, but only numbers listed in the paragraph. Is it possible to find some common feature, e.g. both expanded gene families in migratory but not in non-migratory? maybe it can be more meaningful.

Response: Yes, thank you, we agree. We revised the description and added supplementary figure S13. In fact, we thought exactly the same. What we identified is the genes selected in migratory but not in non-migratory, we just use the oriental stork as a target species.
The expanded gene family/PSGs/REGs identified in the oriental storks when compared to non-migratory birds represent a gene set including migratory-relevant genes (set A) and other unique traits-related (set B) in the oriental stork. Then, we compared the oriental stork with other migratory birds, the potential migratory-relevant genes would not be identified as expanded gene family/PSGs/REGs only in the oriental stork genome, because migratory genes may be shared in the migratory birds, commonly selected in the migratory. So, we used the comparison with other migratory birds to remove the false-positive (set B). The remained genes may represent the genetic mechanisms associated with migration in the oriental stork. Please see Line 343-349 and Supplementary Fig. S13.

Question 6. Line 300. the authors listed and discussed about "collaborative approaches in nervous system and physical energy supply", and listed some other genes inferred may contribute to migration in oriental stork in Fig2B, what about the immune genes? The birds also need to adjust their immune system for migratory.

Response: Yes, thank you, we added. We found several immune genes in the

Powered by Editorial Manager® and ProduXion Manager® from Aries Systems Corporation

expanded gene families, and we also found some immune related genes that were under positive selection or rapidly evolved, please see Line 370-371. We added them to the Supplementary Table S15-S17, and Fig. 5A.

Question 7. Line 364 In fig4, there are 12 diamond shapes outside the wild circle, however, 16 captive-born individuals from Harbin, and the authors wrote "five captive individuals from Harbin, Japan and USA clustered into the wild population" . The numbers are correct. I am wondering do the authors took these five ones as wild-source or captive-source in the following steps? I have no idea about them.

Response: Thank you, we revised and described more clearly, please see Line 271-273. Here the five captive individuals clustered into the wild population are captive-source, they are all captive-born. The PCA result just implied that their genetic background was very similar to the wild population. In population genomic analysis, we divided the samples into groups according to their origins (wild or captive-born), instead of the genetic background.

Question 8. Line 373, "They seemed to have a high heterozygosity when compared with other endangered avian species". There still have some critically endangered/endangered/vulnerable species with higher values than Ciconia boyciana, and the readers cannot distinguish the endangered, endangered, vulnerable from the figure, maybe more comment or distinguish method will be clearer.

Response: Yes, thank you, we revised. In this version, we tried to include more threatened bird species and split the CR, EN and VU to distinguish the three threatened categories, please see Fig. 2C and Supplementary Table S13.

Question 9. Line 376, crested ibis, brown eared pheasant, and kakapo, please list the detail numbers.

Response: Thank you, we revised. Because the calculation method for the crested ibis is not a common method in Feng’s paper [5], it is difficult to compare the value, so we deleted the crested ibis. For the kakapo, Nicolas et al [6] just give a figure to show the low genetic diversity of the kakapo population, we cannot extract the exact π value, so here we also deleted the kakapo. Finally, we used the genetic diversity of the brown eared pheasant and the green peafowl instead, please see Line 282-284.

Question 10. It is interesting to get the results "captive individuals have complex sources" line 465. Maybe more information about pedigree can be useful, if the zoo have these information.

Response: Thank you. We have discussed with the staff and researchers in Harbin North Forest Zoo, who were in charge of animal keeping and sample collection. The captive-born individuals increased quickly in recent thirty years, from 1 (in 1975) to 383 (in 2006) (Response Figure 1), due to the success of assist-breeding technology. For the following large generations, there weren’t clear pedigree. They told us that till now, they have bred 10-13 generations stem from the wild founders.

Response Figure 1. The number of captive oriental storks in all zoos from 1975 to 2006. (In the uploaded supplementary materials: Response to reviewer comments)

We also searched the literature but failed to find any pedigree records. We just found two origin information of the wild founders. The first reproduction in the Harbin Forest Zoo was in 1991 between two wild individuals from the Sanjiang Plain, Heilongjiang Province (130.12°-135.09°E, 43.83°-48.67°N)[7]. And in 2008, there had another ten wild individuals from Honghe National Nature Reserve (133°37′-133°45′E, 47°43′-47°52′N), Heilongjiang Province [8].

Reviewer #2: The paper describes the first reference genome built for the Oriental stork. The authors have then resequenced storks to asks a number of important

	questions about the species and its conservation. The Methods are sound and the findings are interesting. However, the paper suffers from a number of shortcomings that together would qualify as major revision. The paper will also need a thorough revision for proper grammar. More specific comments are below.<br><br>Response to reviewer 2: Thank you very much for your support. And we have made an extensive revision as your comments and we believe the current manuscript has been improved a lot with your help. We learned a lot from the questions you raised, particularly for the microchromosomes, Ne/Nc and genetic drift. The most importantly, we thank for the suggestion on the whole manuscript organization and the discussion section. We hope this revision could address the questions. We also invited a native speaker to polish the language (Dr. Sunil).<br><br>Question 1. The paper often lacks clear links between the specific analyses and, therefore, the questions being addressed<br>The authors have done a tremendous amount of work on a very thorough analysis. At present, however, the paper reads like a collection of disparate analyses with tenuous links between many of them. For example, how does the gene ontology analysis relate to the demographic modeling and how do these relate to stork conservation?<br>Response: Thank you very much, this is a very important suggestion which would much improve our manuscript. We reorganized our analysis and made each part more correlated and make this study more like a conservation-oriented study.<br>Firstly, only with a high-quality chromosome-level reference genome can we conduct a precise assessment on many genetic parameters in conservation genetics. So, the first foundational part was the reference genome assembly.<br>Secondly, our central part of this manuscript was to assess the genetic status and extinction risk of the oriental stork population, which was measured by population structure, demographic history, genetic diversity, inbreeding level and mutational load. So, these analyses are the second large part that was close related to conservation genomics.<br>Thirdly, conservation and evolution are closely connected. Migratory birds are under selective pressure from the early life, particularly juveniles need to response to the condition change in the first fall. They need to migrate efficiently to arrive early to the stop-over/wintering sites because the habitat/food resources are limited. Behavior study has proved that learning plays an important role in shaping the migratory route through a lifetime [9]. We thought that it was essential to understand the genomic basis of the migratory adaptation. Because the ecological adaptation is vital for the population survival, which may have evolved for millions of years. Therefore, the investigation on the migratory trait of the oriental stork is the third part in our study.<br>We reorganized each section and made the manuscript clearer.<br><br><br>Question 2. The paper needs a more fully developed Discussion section<br><br>Response: Thank you, we extensively revised the Discussion section and made it more thoughtful and more reasonable, please see Line391-458. Especially, we added the detailed development history of the southeast Sibera and the Bohai Bay, to connect genomic result with ecological information, please see Line427-443.<br><br><br>Question 3. Are all individuals migratory? Or is this species a partial migrants like the American wood stork? See Picardi et al 2020 https://doi.org/10.1002/ecs2.3054<br><br>Response: Thank you for this question. The oriental stork, Ciconia boyciana, is a full-migratory avian, assessed by BirdLife International (https://www.iucnredlist.org/species/22697695/131942061) and other researches by GPS/GSM tracking (Response Figure 2) [10, 11]. We didn’t find any studies claiming that some oriental storks wintered in their breeding area in northeastern Asia.<br><br>Response Figure 2. Individual autumn migration routes and stop-over sites of 18 oriental storks derived from GPS/GSM telemetry devices [10]. (In the uploaded supplementary materials: Response to reviewer comments)<br><br>Question 4. The authors argue that drift is weak in this large single population of storks.

What is the Ne/Nc ratio? Does that support their argument?

Response: Thank you. We redo the related analysis and added more analysis. The Ne of the wild populations has been about 1,000 for the past decades (Fig. 4C). And in recent twenty years, the census size (Nc) was 3,000. The Ne/Nc ratio is 0.33, which fell within the range of most species [12], please see Line 329-330.
We also calculated the folded SFS of derived alleles, which showed a "L-shaped" in the wild and captive populations (Fig. 3B). This result also proved that singletons were abundant in the population, and so genetic drift seems not concerning [13]? But we noticed that low-frequency alleles were deficient while the medium-frequency was excessive in the captive population compared to the wild population. Thus, the genetic drift seemed to be slightly stronger in the captivity, please see Line 311-316. This point can also be reflected by the dispersal PCA points while a unified ancestral component in the Admixture plot of captive individuals.

Question 5. Lines 443-445- what gaps will your genome fill that will allow for targeted recovery plans?

Response: Thank you for this question. Conservation efforts for the threatened species can be enhanced by a high-quality reference genome. We can obtain a more precise and more detailed measures for many key genetic factors in conservation genomics [14]. For example, the evaluation of inbreeding by measuring runs of homozygosity (ROH) highly depends on the high-quality reference genome with outstanding contiguity. This is due to the ROH in small populations with high-level inbreeding often spans over several millions of base pairs [15-17], and hardly be detected based on fragmented genomes assembled from short reads. Additionally, the genetic diversity(π) could also be promoted by a higher-quality reference genome, and we could often detect more variants across the genome based on the Long-read assembled genome than the Short-read assembled genome, because long reads could 1) span much more complex genomic regions[14] and 2) generate much longer contigs than short reads[18, 19]. Many genomic regions that cannot be assembled from short reads can be assembled from long reads, and these regions may contain important variants. Additionally, longer contigs facilitate a higher number of reads aligning accurately to the reference genome. Both of the two aspects could contribute to enhancing the accuracy of genetic diversity calculations. As we mentioned in Q4 of reviewer 1, the high-quality genome could be also well used to the identification of sex chromosomes [20]. As for our genome, we comprehensively assessed the genetic diversity and inbreeding, revealing a relatively positive genetic status with high heterozygosity, low inbreeding, and recent population decline. We found two captive-born oriental storks with higher inbreeding levels (~30%) than others (<10%). And recent inbreeding (reflected by long ROHs, > 1 Mb) was significantly more serious in captive-born oriental storks than the wild (Fig. 2D). These findings provided an alarming that captive-breeding programs should be more cautious on the pedigree management.

Question 6. The authors may wish to cite Flamio, R, Jr & Ramstad, KM. 2023. Chromosome-level genome of the wood stork (Mycteria americana) provides insight into avian chromosome evolution. Journal of Heredity, doi:10.1093/jhered/esad077. How does your genome compare? And how did you treat microchromosomes?

Response: Thank you for this question. We checked the Hi-C heatmap, fortunately all chromosomes were successfully assembled including 23 microchromosomes (Response Figure 3). Perhaps due to the high sequencing depth of Hi-C data (~98.20X), we didn't observe obvious blank gaps within large scaffolds that could be anchored onto chromosomes (Response Figure 4). There were still some small scaffolds having limited contact with other chromosomes, we just leave them (0.68% of the total length of the assembled scaffolds) as unanchored genomic fragments.
We didn't pay special attention to microchromosomes because this study did not discuss the avian chromosome evolution. We applied the same methods to the microchromosomes as the macrochromosomes in each analysis. But we thought that the microchromosomes deserved more detailed description on results and discussion, we referred to this the wood stork study and added some sentences, please see Line 401-406.

Response Figure 3. Heatmap of Hi-C chromosomal interaction density among all 35 chromosomes (33 autosomes + ZW, 2n=68). (In the uploaded supplementary materials: Response to reviewer comments)

Response Figure 4. Heatmap of Hi-C chromosomal interaction density among 23 microchromosomes.

Additional comments:
Question 7. Please provide additional information on the captive storks sampled. How many generations of captive breeding did they stem from?

Response: Thank you. We have discussed with the staff and researchers in the Harbin North Forest Zoo, who were in charge of animal keeping and sample collection. They told us that the captive-born oriental storks were 10-13 generations stem from the wild founders. We added the information of captive samples in supplementary table S11.

Question 8. Lines 86 & 89 - what does 'rescued' mean in this context?

Response: Thank you, we revised the description here and added the detailed rescue situation. The three wild samples from Harbin North Forest Zoo (Line 87), were successfully rescued from the wild and now live in the veterinary hospital of the zoo. The 26 wild individuals in Line89 were found to be ill/starving/dying when they were rescued in Bohai Bay in migratory season of 2022, and fail to be rescued and were dead then, so we could collect the muscle samples after death. We added the rescue information in supplementary table S11.

Question 9. Clarify if the selection analysis was to identify loci under selection in Oriental storks, storks more broadly, or migratory birds. Lines 156-158 could be expanded and the text throughout modified to be very specific in this regard.

Response: Thank you, we revised. The comparative genomic analysis was to identify loci under positive selection in oriental stork. We redescribed these sentences to make it more readable and clearer.

Question 10. Reduce the number of or simplify the figures where possible

Response: Thank you, we revised and simplified the Figures 1, 3, 4 and 5. Other subfigures were moved to supplementary materials.

Question 11. Lines 242-247 - this text should be moved to the Methods section

Response: Thank you, we polished these sentences. We maintained the important assembly results here and moved other description to the Methods section.

Question 12. Table 1 caption - change 'of this' to 'the genome'

Response: Corrected, thank you.

Question 13. Figure 4A - what does this analysis look like if you include only the wild individuals?

Response: Thank you, we tested. We just used the 29 individuals from the wild to conduct the PCA analysis, please see Response Figure 3 (from left to right: PC1PC2, PC1PC3, and PC2PC3). We didn’t observe a population structure within the wild individuals, all wild individuals were scattered in the PCA plot. In fact, we think this is consistent with the actual population survey, because there is just one Eastern Asia

population of wild oriental stork, and they share the same breeding areas and migratory routes [21].

Response Figure 5. The PCA analysis of the 29 wild oriental storks. (In the uploaded supplementary materials: Response to reviewer comments)

--
Please also take a moment to check our website at https://www.editorialmanager.com/giga/l.asp?i=154222&l=K4I56MXD for any additional comments that were saved as attachments. Please note that as GigaScience has a policy of open peer review, you will be able to see the names of the reviewers.

____
In compliance with data protection regulations, you may request that we remove your personal registration details at any time. (Use the following URL: https://www.editorialmanager.com/giga/login.asp?a=r). Please contact the publication office if you have any questions.

1.Ma X. Research on captive oriental white stork (Ciconia boyciana) in energy of digestion and fledgling growth. Northeast Forestry University, 2007.
2.Liu Z and Li X. The research progress of oriental white stork. Territory & Natural Resources Study. 2008; 01:77-8. doi:10.16202/j.cnki.tnrs.2008.01.001.
3.Zeng S, Cheng L and Li X. The numerical distribution and conservation of oriental white stork in China. Territory & Natural Resources Study. 2003; 01:71-2. doi:10.16202/j.cnki.tnrs.2003.01.035.
4.International B. The IUCN Red List of Threatened Species 2018: e.T22697695A131942061. 2018; doi:https://dx.doi.org/10.2305/IUCN.UK.2018-2.RLTS.T22697695A131942061.en.
5.Feng S, Fang Q, Barnett R, Li C, Han S, Kuhlwilm M, et al. The Genomic Footprints of the Fall and Recovery of the Crested Ibis. Current Biology. 2019;29:340-9. doi:10.1016/j.cub.2018.12.008.
6.Dussex N, van der Valk T, Morales HE, Wheat CW, Díez-del-Molino D, von Seth J, et al. Population genomics of the critically endangered kākāpō. Cell Genomics. 2021;1 doi:10.1016/j.xgen.2021.100002.
7.LI L, Wei H and Gao Z. Preliminary observation on the reproduction ecology of captive oriental white stork. Chinese Journal of Wildlife. 1996; 06:14-7. doi:10.19711/j.cnki.issn2310-1490.1996.06.004.
8.Zan S, Zhou L, Jiang H, Zhang B, Wu Z and Hou Y. Genetic structure of the oriental white stork (Ciconia boyciana): implications for a breeding colony in a non-breeding area. Integrative zoology. 2008;3 3:235-44. doi:10.1111/j.1749-4877.2008.00096.x.
9.Aikens EO, Nourani E, Fiedler W, Wikelski M and Flack A. Learning shapes the development of migratory behavior. Proceedings of the National Academy of Sciences. 2024;121 12:e2306389121. doi:doi:10.1073/pnas.2306389121.
10.Fan S, Zhao Q, Li H, Zhu B, Dong S, Xie Y, et al. Cyclical helping hands: seasonal tailwinds differentially affect migrating Oriental Storks (Ciconia boyciana) travel speed. Avian Research. 2020;11 1:10. doi:10.1186/s40657-020-00196-8.
11.Yang Z, Chen L, Jia R, Xu H, Wang Y, Wei X, et al. Migration routes of the endangered Oriental Stork (Ciconia boyciana) from Xingkai Lake, China, and their repeatability as revealed by GPS tracking. Avian Research. 2023;14 2:100090. doi:10.1016/j.avrs.2023.100090.
12.Hare MP, Nunney L, Schwartz MK, Ruzzante DE, Burford M, Waples RS, et al. Understanding and estimating effective population size for practical application in marine species management. Conservation biology. 2011;25 3:438-49. doi:10.1111/j.1523-1739.2010.01637.x.
13.Wang P, Burley JT, Liu Y, Chang J, Chen, Lu Q, et al. Genomic Consequences of Long-Term Population Decline in Brown Eared Pheasant. Molecular Biology and Evolution. 2021;38 1:263-73. doi:10.1093/molbev/msaa213.
14.Formenti G, Theissinger K, Fernandes C, Bista I, Bombarely A, Bleidorn C, et al.

The era of reference genomes in conservation genomics. Trends in ecology & evolution. 2022;37 3:197-202. doi:10.1016/j.tree.2021.11.008.
15.Saremi NF, Supple MA, Byrne A, Cahill JA, Coutinho LL, Dalen L, et al. Puma genomes from North and South America provide insights into the genomic consequences of inbreeding. Nature communications. 2019;10 1:4769. doi:10.1038/s41467-019-12741-1.
16.Xie HX, Liang XX, Chen ZQ, Li WM, Mi CR, Li M, et al. Ancient Demographics Determine the Effectiveness of Genetic Purging in Endangered Lizards. Molecular biology and evolution. 2022;39 1 doi:10.1093/molbev/msab359.
17.Dussex N, Van Der Valk T, Morales HE, Wheat CW, Díez-del-Molino D, Von Seth J, et al. Population genomics of the critically endangered kākāpō. Cell Genomics. 2021;1 1.
18.Zhang L, Lan T, Lin C, Fu W, Yuan Y, Lin K, et al. Chromosome-scale genomes reveal genomic consequences of inbreeding in the South China tiger: A comparative study with the Amur tiger. Molecular ecology resources. 2022; doi:10.1111/1755-0998.13669.
19.Yang S, Lan T, Zhang Y, Wang Q, Li H, Dussex N, et al. Genomic investigation of the Chinese alligator reveals wild-extinct genetic diversity and genomic consequences of their continuous decline. Molecular ecology resources. 2022; doi:10.1111/1755-0998.13702.
20.Paez S, Kraus RHS, Shapiro B, Gilbert MTP, Jarvis ED, Group VGPC, et al. Reference genomes for conservation. 2022;377 6604:364-6. doi:doi:10.1126/science.abm8127.
21.Ga R. The distribution and population size and trend of Oriential Stork (Ciconia boyciana) and the habitat selection of the overwintering group in Bohai Bay. Master, Inner Mongolia Normal University, 2021.

Additional Information:	
**Question**	**Response**
Are you submitting this manuscript to a special series or article collection?	No
**Experimental design and statistics**<br><br>Full details of the experimental design and statistical methods used should be given in the Methods section, as detailed in our Minimum Standards Reporting Checklist. Information essential to interpreting the data presented should be made available in the figure legends.<br><br>Have you included all the information requested in your manuscript?	Yes
**Resources**<br><br>A description of all resources used, including antibodies, cell lines, animals and software tools, with enough information to allow them to be uniquely identified, should be included in the Methods section. Authors are strongly encouraged to cite Research Resource	Yes

Identifiers (RRIDs) for antibodies, model organisms and tools, where possible.<br><br>Have you included the information requested as detailed in our Minimum Standards Reporting Checklist?	
**Availability of data and materials**<br><br>All datasets and code on which the conclusions of the paper rely must be either included in your submission or deposited in publicly available repositories (where available and ethically appropriate), referencing such data using a unique identifier in the references and in the "Availability of Data and Materials" section of your manuscript.<br><br>Have you have met the above requirement as detailed in our Minimum Standards Reporting Checklist?	Yes

Manuscript

Click here to access/download;Manuscript;Genomic exploration of the endangered oriental stork-clean.docx

Genomic exploration of the endangered oriental stork, *Ciconia boyciana*, shed lights on migration adaptation and future conservation

Shangchen Yang[1,2†], Yan Liu[3†], Xiaoqing Zhao[4,5†], Jin Chen[1], Haimeng Li[1,6], Hongrui Liang[3], Mengchao Zhou[1], Shiqing Wang[1], Xiaotian Zhang[3], Minhui Shi[1], Lei Han[1], Mingyuan Yu[3], Yaxian Lu[1], Boyang Liu[1], Yu Xu[3‡], Tianming Lan[1,6*], Zhijun Hou[1*]

[1]College of Wildlife and Protected Area, Northeast Forestry University, Harbin, 150040, China

[2]College of Life Sciences, Zhejiang University, Hangzhou 310058, China

[3]Center for Biological Disaster Prevention and Control, National Forestry and Grassland Administration, Shenyang 110034, China

[4]Inner Mongolia Academy of Agricultural & Animal Husbandry Sciences, Hohhot 010031, Inner Mongolia, China;

[5]Key Laboratory of Black Soil Protection and Utilization (Hohhot), Ministry of Agriculture and Rural Affairs, P.R. China, Hohhot 010031, Inner Mongolia, China;

[6]Heilongjiang Key Laboratory of Complex Traits and Protein Machines in Organisms, Harbin 150040, China

[*]**Correspondence address.** Tianming Lan, College of Wildlife and Protected Area, Northeast Forestry University, Harbin, China. E-mail: lantianming1314@126.com; Zhijun Hou, College of Wildlife and Protected Area, Northeast Forestry University, Harbin, China. E-mail: houzhijundb@163.com

[†]These authors contributed equally to this work.

[‡]This author jointly supervised the work.

Abstract

Background: The oriental stork, *Ciconia boyciana*, is an endangered migratory bird on the IUCN Red List. They experienced a rapidly decline in the past decades, with nest locations and stop-over sites largely degraded due to human-bird conflicts. The genome-wide genetic status of this threatened bird population is critical to make future conservation strategies but lack of investigation.

Findings: We presented the first chromosome-scale genome for the oriental stork with high quality, contiguity, and accuracy. The assembled genome size was 1.24 Gb with a scaffold N50 of 103 Mb, and 1.23 Gb contigs (99.32%) were anchored to 35 chromosomes. We didn't find a genetic structure in the wild population. Genome-wide genetic diversity (π = 0.0012) of the oriental stork was at a moderate to high level among threatened bird species and inbreeding risk was not concerning (F_{ROH} = 5.56±5.30%). Demographic history reconstruction indicated a rapid recent decline likely driven by human activities. Genomic selective signals of the migratory trait were identified in genes related to the long-term potentiation, photoreceptor cell, circadian rhythm, muscle development and energy metabolism, indicating the essential interplay between genetic and ecological adaptation.

Conclusions: The first chromosome-scale genome of the oriental stork expands genomic resource of endangered birds, providing a genomic basis for understanding its genetic background, extinction risk and the migratory characteristic, which will further facilitate the decision of future conservation plans for such a migratory bird.

Keywords: oriental stork, comparative genome, population genetics, endangered species, migration

Introduction

Ecosystem degradation and biodiversity decline occur as a feature throughout the Anthropocene and seems to be accelerated in the coming years [1]. Human-induced habitat loss, overexploitation, and pollution largely speed up the pace of the sixth mass extinction and now more than 32% of extant species (~44,000) are threatened with extinction [2]. This global crisis in turn harm to human well-being, and urgently need more conservation efforts to stop and reverse current situation.

Birds are effective wildlife indicator of the biodiversity on earth [3]. Long-term record from BirdLife International raised significant concern for the world's birds: 49% of bird species (5,412) populations are declining, including both endangered and unendangered birds, and many species are at an escalating risk of extinction. Nearly 45% of Important Bird and Biodiversity Areas (IBAs) are identified in danger due to pervasive and unsustainable human activities, like agricultural expansion and intensification, logging, and hunting [4-6]. In particular, wetlands in the East Asian-Australasian Flyway (EAAF) (Fig. 1A) are being heavily destroyed, directly causing key sites loss and bird mortality [7, 8]. This flyway is used by 492 migratory bird species, totally more than 50 million individuals, from Arctic Russia and Alaska, to Australia and New Zealand [9, 10]. Due to human-bird conflicts in Asia, around half of the migratory waterbirds suffering population falling, including the endangered oriental stork, *Ciconia boyciana* [11] and black-faced spoonbill, *Platalea minor* [12], the vulnerable white-naped crane, *Antigone vipio* [13]. Migratory birds are wildlife without boundaries and serve as a powerful safeguard for ecosystems. The decline of bird species in EAAF largely reduce the energy connectivity and mobility between Arctic nutrient-poor terrestrial system and southern coastline ecosystem. Although government has taken actions to protect coastal wetlands and further to protect migratory birds, there still many gaps remained to be filled [14]. For example, for most of these threatened migratory birds, the whole-genome data for population is lacking and thus difficult to assess their genome-wide genetic background, bringing obstacles to design scientific recovery actions.

Oriental stork is a large wetland shorebird in the EAAF. It has been listed as "Endangered" on the IUCN Red List since 1994, with an estimated population size of c. 3000 individuals and a single population (Eastern Asia population) [15]. They are full migrants, breeding in southeastern Siberia, mainly along the Russia-China border and annually migrating to Bohai Bay (1,500 km) and Poyang Lake (2,600 km) in autumn (Fig. 1B) [16]. Oriental storks were once widely distributed across northeast Asia, however, the wild population dramatically declined in 1868-1935 [17]. In 1970s, wild storks in Japan and Korea disappeared, with the remaining individuals breeding in more constricted areas in Russian Far East and China. In 1960s, there were more than 1000 oriental storks breeding in Heilongjiang Province. However, the number decreased to 123 in 1986 and was less than 50 in 1990 [18, 19]. Habitat loss is considered as the major reason for their population decline. Deforestation, agricultural development, and spring fires severely destroy their nest trees in Russia. Reclamation of wetlands, and overfishing in the stop-over and wintering sites in China lead to a decreased refueling rate and an increased mortality rate, particularly for juveniles [15, 20]. Future conservation actions for the oriental stork are undoubtedly need to be further enhanced. However, the population status of the oriental stork was largely underexplored, particularly for genome-wide investigation, including their genetic diversity, inbreeding status, as well as the genomic basis for the adaptation of migration traits.

Here, we presented the first chromosome-level genome for the oriented stork, and re-sequenced 29 wild and 15 captive individuals to extensively explore genetic characteristics of this endangered bird. We systematically investigated the genetic background for these individuals to measure possible genome-wide extinction risks. We also scanned the genic regions to find genomic signatures of evolution and adaptation for the migratory-related characteristics, which is of vital importance for the population viability. This study provides a valuable genomic resource to assess the ecological adaption and conservation status of the oriental stork and will aid for the future conservation.

Methods

Samples and ethics statement

Blood samples from 16 captive-born and 3 wild-rescued oriental storks were collected at Harbin North Forest Zoo, Harbin, Heilongjiang, China. The blood sample for a captive-born individual (sample ID: N1170) was used for reference genome assembly. The 26 wild-rescued oriental storks were collected around the Bohai Bay, China, and the muscle samples from these individuals were collected after their natural death. Research and blood/tissue collection were approved by the Institutional Review Board of Northeast Forest University (No.2024WPE05). We also downloaded whole-genome sequencing data of two published individuals from Kanagawa, Japan (*n*=1) and San Diego Zoo, the USA (*n*=1). Finally, our data set was consisted of 46 samples, including 26 wild individuals from Bohai Bay, 18 from Harbin North Forest Zoo (3 wild and 15 captive), 1 captive from Japan and 1 captive from the USA.

Nucleic acid extraction, library construction and sequencing

For ONT sequencing, high molecular weight genomic DNA was extracted by the DNeasy Blood and Tissue kit (Qiagen,USA), and 8-10 μg DNA was size-selected (> 50 kb) according to the manufacturer's instructions for ONT library preparation. 800 ng DNA library were used for sequencing on the PromethION sequencer (Oxford Nanopore Technologies, UK). For Hi-C sequencing, cross-link process with formaldehyde was firstly conducted using the blood sample and then Hi-C library was constructed following the protocol of Lieberman-Aiden et al[21]. Total RNA was extracted using TRlzol reagent (Invitrogen), and Agilent 2100 Bioanalyser system (Agilent, USA) and Qubit 3.0 (Life Technologies, USA) were used for RNA quality and quantity evaluation. DNA libraries with short insert sizes were prepared according the manufacturer's instruction of the MGI platform (MGI, Shenzhen, China). These libraries were finally sequenced on the DNBSEQ-T1 sequencer for 100-bp paired-end reads.

De novo assembly, annotation, and assessment

We first estimated the genome size of the oriental stork by *k*-mer frequency method based on WGS data of the assembled individual[22]. Then we performed the genome assembly: 1) *de novo* assembly was conducted using ONT long reads by NextDenovo(v2.5.0, https://github.com/Nextomics/NextDenovo). Two core modules were utilized to generate a primary assembly: the NextCorrect module was for raw ONT long-reads correction and consensus sequence extraction; the NextGraph module was for preliminary assembly. Here, we set the read cutoff as 1 Kb and maintained other default parameters in NextDenovo. 2) Contigs were polished using NextPolish(v1.4.0)[23] with ONT long reads. 3) Hi-C reads were mapped to the genome using Burrows-Wheeler aligner *mem* (BWA, v0.7.17)[24] with default parameters. 3d-DNA pipeline(v180,922) was applied to convert the contigs into a chromosome-level genome. 4) WGS reads were remapped to the assembly[25] to error-correct the mis-sequenced bases introduced from the long-read sequencing. 5) Benchmarking Universal Single-Copy Orthologs (BUSCO) analysis[26] was performed to evaluate the completeness of our assembly using aves_odb10 database. 6) WGS, Hi-C and RNA-seq data were mapped to the final genome to check mapping rate, bases coverage and sequencing depth by BWA *mem* algorithm with default parameters.

De novo and homology-based methods were combined to identify repetitive elements in the final assembly. Firstly, *de novo* prediction was performed using LTR finder(v1.0.6)[27], MITE-hunter(v4.07)[28] and RepeatModeler2(v2.0.1)[29] software with default parameters. Then, the results were merged into the RepBase as known repeats. Next, RepeatMasker(v4.0.5)[30] was used to identify and classify transposable elements by searching RepBase library[31]. Tandem repeats were identified using Tandem Repeats Finder (TRF, v4.09)[32].

All repetitive elements were masked to annotate the protein-coding genes. We used a combination of *de novo*, homology-based and transcript mapping methods to conduct gene annotation. *De novo* prediction was carried out using SNAP(v1.0)[33], glimmerHMM(v3.0.3)[34] and AUGUSTUS(v2.5.5)[35] software. RNA data was filtered by Trimmomatic(v0.27)[36] and assembled by Trinity(v2.9.0)[37] and then mapped to the reference genome to

predict gene structure using Program to Assemble Spliced Alignments (PASA, v2.2.0)[38]. For homology-based prediction, we used protein sequences from *Gallus gallus*, *Anas platyrhynchos*, *Ciconia maguari*, *Meleagris gallopavo*, *Pavo muticus*, *Taeniopygia guttata* and *Homo sapiens*, to align against our genome by Blastall(v2.2.26)[39] with E-value cut-off of 1e-5 and then gene models were confirmed using GeneWise(v2.4.1)[40]. The results of three approaches above were finally combined to generate a comprehensive gene set by Maker(v 3.01.03)[41]. These genes were aligned in the databases of SwissProt, TrEMBL, InterPro, Gene ontology (GO) and Kyoto Encyclopedia of Genes and Genomes (KEGG) for functional annotation.

Identification of sex-linked regions

Sex chromosomes (Z and W) were firstly identified by checking the sequencing depth of male and female individuals. Then we examined syntenic relationships with sex chromosomes of *G. gallus* (GenBank ID: GCA_016699485.1) and *T. guttata* (GenBank ID: GCF_003957565.2). Alternative splicing of each gene on the chromosomes were filtered for the three species. We aligned the longest protein sequence of *G. gallus* and *T. guttata* against our assembled Z and W chromosomes using blastp in BLASTtools(v2.2.26)[42] with the parameter of "-evalue 1e-5". And then, synteny blocks were identified using MCScanX[43] and visualized by Circos(v0.69-9)[44] software.

Phylogeny reconstruction and divergence time estimation

We performed an alignment of protein sequence from 24 species (*H. sapiens*, *Anolis carolinensis*, *Alligator sinensis*, *G. gallus*, *Cygnus olor*, *Asarcornis scutulata*, *A. platyrhynchos*, *T. guttata*, *Strigops habroptilus*, *Falco peregrinus*, *Herpetotheres cachinnans*, *Spizaetus tyrannus*, *Accipiter gentilis*, *Haliaeetus albicilla*, *Charadrius vociferus*, *Rostratula benghalensis*, *Larus smithsonianus*, *Balearica regulorum*, *Grus americana*, *C. maguari*, oriental stork, *Scopus umbrette*, *Nipponia nippon*, *Egretta garzetta*). The longest protein sequence of each gene was selected in this alignment by BLASTtools(v2.2.26)[42] blastp function with "-evalue 1e-5". A total of 1,800 shared single-copy

genes were used to construct a phylogenetic tree by IQTREE(v1.6.12)[45] using the maximum-likelihood algorithm. Divergence time among these species was estimated by MCMCTREE(v4.5) in PAML[46] software with multiple fossil time points used for time calibration[47].

Variants calling and quality control

Whole-genome sequencing data of each individual was mapped to our assembled genome using the BWA *mem* with default parameters. Reads sorting, reordering and deduplication were carried out with Picard(v2.1.1) software and then the variant calling was performed using Sentieon(v202010.01)[48] DNAseq Haplotyper. Bam files and genomic Variant Call Format (gVCF) file were generated for each individual and joint calling was conducted using Sentieon DNAseq GVCFtyper to generate a combined VCF file covering all individuals. Variants were filtered using the following procedures: 1) InDels and multi-allelic variants were removed; 2) hard filtering with the parameters: QD < 2.0 || FS > 60.0 || MQ < 40.0 || MQRankSum < -12.5 || ReadPosRankSum < -8.0 –filter-name snp_filter; 3) genotype quality of a site lower than 20 were marked as missing; 4) genotype missing rate larger than 10% were removed from the variation set. Single-nucleotide polymorphism (SNP) sites on the Z and W chromosomes were also removed for the downstream population genomic analysis.

Population structure analysis

The VCF file was converted into PLINK format files with VCFtools(v0.1.16)[49] and then Principal component analysis (PCA) was performed with PLINK(v1.9)[50] software. Inference of ancestral components was conducted with ADMIXTURE(v1.3.0)[51] and *K* value was set from one to five with "-cv" flag to calculate the cross-validation (CV) error. Phylogenetic tree was constructed using IQTREE(v1.6.12) with 1000 bootstraps. The tree layout was visualized by using the online tool iTOL(http://itol.embl.de).

Genetic diversity and inbreeding

Genome-wide heterozygosity (*H*) of each individual genome was the proportion of heterozygous SNPs in the assembled autosomal genome, which was calculated by the VCFtools(v0.1.16). Nucleotide diversity (π) was calculated by a non-overlapping 5 Mb sliding window along all autosomes using VCFtools(v0.1.16). Runs of homozygosity (ROHs) were identified by PLINK(v1.9) with the following parameters: --homozyg-window-snp 20 --homozyg-kb 100 --homozyg-density 50. Inbreeding coefficient was estimated as the proportion of genome fraction that in ROH region (F_{ROH}). Comparison between wild and captive populations was conducted using two-sided pairwise *t*-test in R(v 4.1.2).

Mutational load

We used alleles in *C. maguari* genome, the closest relative of oriental stork, to serve as ancestral state across the genome. The reference of *C. maguari* (GenBank ID: GCA_013399255.1) was transformed to a 100 bp FASTQ file by sliding a nonoverlapping window across the genome and then short reads were mapped to our assembled genome using BWA *mem* with the parameter: -B 3. Only reads mapped uniquely to our genome were kept by SAMtools(v1.3)[52] view function with "-F 4 -q 20". Finally, we generated a consensus sequence to represent ancestral alleles on the oriental stork genome using SAMtools(v1.3) mpileup function with depth filter of ≤ 1×. We obtained a new VCF file containing 6,028,662 derived SNPs after replacing the reference alleles by a custom Perl script.

SnpEff (v4.3) software was used to annotated the derived SNPs into three categories: (1) synonymous mutations; (2) missense mutations; (3) loss of function (LoF) mutations. Here, we considered "stop_gained", "splice_donor_variant" and "splice_acceptor_variant", "start_lost", "stop_lost" and "splice_region_variant" as LoF mutations. Next, we counted the number of derived SNPs per individual in homozygous and heterozygous state, respectively. The proportion of homozygous derived SNPs was measured following the formula: 2 × homozygous sites / (2 ×

homozygous sites + heterozygous sites)[53]. Derived allele frequency was calculated with 15 randomly selected individuals in each population to avoid the potential bias from sample size.

Inference of population demography

We combined Pairwise Sequentially Markovian Coalescent (PSMC, v0.6.5)[54], SMC++(v1.13.1)[55] and approximate Bayesian computation (ABC) methods to track the population dynamics of wild population over generations. For the PSMC analysis, we converted the bam file of each individual to a fasta format sequence using SAMtools(v1.3)[52] mpileup function, and sequencing depth ≥ 1/3 and ≤ 2 was retained. PSMC software was then run with the parameters: -N25 -t5 -r5 -p 4+25*2+4+6. For the SMC++ method, we randomly selected two individuals from wild population to generate a mask file of uncovered regions by bamCaller.py. SMC++ was applied based on covered sites to infer population history with the following parameters: --cores 8 --knots 24 --timepoints 20 100000. For the PopSizeABC analysis, SNP sites with a MAF > 0.2 were used as an input file for PopSizeABC(v2.1)[56] software with the parameters: mac (minor allele count threshold for AFS and IBS statistics computation) = 0; mac_ld (minor allele count threshold for LD statistics computation) equals 3,4,5 respectively; L (size of each segment, in bp) = 4,000,000; nb_rep (number of simulated data sets) = 500; nb_seg (number of independent segments in each data set) = 30. The output results of three methods were visualized with a generation time of 16 years and the mutation rate of 4.0×10^{-9} substitutions per site per generation [15].

Comparative genomic analysis related to migration

To understand the genome evolution in oriental stork, we conducted comparative genomic analyses with un-migratory bird species, also compared with other migratory birds. Unique adaptative signals detected in the oriental stork with un-migratory group, which were absent for the oriental stork when compared to other migrants, were regarded as potential genetic factors contributing to the migratory phenotype. Here, we looked for expanded gene

families, positively selected genes (PSGs) and rapidly evolving genes (REGs). We used Treefam(v1.4)[57] and CAFÉ(v4.2.1)[58] to identify expanded and contracted gene families. PSGs and REGs were identified under a branch model and a branch-site model based on the single-copy gene sets in the CodeML of PAML(v4.8)[46] with the threshold of the false discovery rate adjusted *P*-value set as 0.05. GO and KEGG enrichment analyses were performed using the “cluserProfiler” package in R (v4.0.2)[59, 60]. Networks of GO terms were visualized by REVIGO to summarize redundant terms[61].

Detecting genomic signatures of recent adaptation

SNPs in the wild population were phased by BEAGLE(v5.0)[62] with the default parameters. Recently positive selection was detected using the integrated haplotype score (iHS, version 1.3)[63] method and iHS scores were normalized by subtracting the genome-wide mean iHS score and dividing by the standard deviation by using the software WHAMM (http://coruscant.itmat.upenn.edu/whamm/index.html). SNPs with the highest or lowest 0.1% standardized iHS scores were considered as candidate ancestral or derived alleles that were under strong positive selection. We used three methods to select genes that were under recently positive selection: 1) Genes in a 5 Kb flanking region around candidate SNPs; 2) Sliding 100 Kb windows across the whole genome, genes were selected as candidates if they intersected with 100 Kb windows containing candidate SNPs; 3) Sliding 50 SNP windows across the whole genome, genes were selected as candidates if they intersected with 50 SNP windows that contained candidate SNPs; 4) Genes harboring candidate SNPs.

Results

Chromosome-level genome assembly and annotation

A reference genome for a female oriental stork was assembled by combining ONT long reads (~89.81-fold), DNB short reads (~69.77-fold) and Hi-C reads (~98.20-fold) (Supplementary Table S1). The genome size of the oriental

stork was estimated to be 1.29 Gb (Supplementary Fig. S1). The final assembled chromosome-scale genome had a genome size of 1.24 Gb with 35 chromosomes (Fig. 1C, Table 1 and Supplementary Fig. S2).The scaffold N50 of this genome was 102.77 Mb and the contig N50 was 35.79 Mb (Supplementary Table S2). More than 99.32% of all contigs were successfully anchored onto the chromosomes. The GC content of the oriental stork genome was 42.40%, very close to that of its related species, *C. maguari* (GenBank ID: GCA_013399255.1, 40.90%) and *S. umbrette* (GenBank ID: GCA_013400535.1, 41.50%). Assembly in this study also showed high completeness with a BUSCO score of 97.6% (Supplementary Table S3). Lastly, 99.73%, 99.89% and 94.80% of the WGS, Hi-C and RNA-seq reads could be successfully mapped onto the final assembly (Supplementary Table S4). We observed that depth ratio of male/female on the Chr8 and Chr16 were about 2 and 0, respectively, consistent with the mapping pattern of Z and W chromosomes (Supplementary Fig. S3 A-C). Syntenic analysis with *G. gallus* and *T. guttata* also supported that Chr8 and Chr16 were Z and W chromosomes, respectively (Supplementary Fig. S3 D and E).

Table 1: Statistics of the genome assembly for the oriental stork.

**Genomic features**	**Parameters**
Assembled genome size (bp)	1,240,615,254
Contig N50 (bp)	35,788,150
Scaffold N50 (bp)	102,765,642
Longest contig (bp)	131,589,000
Longest scaffold (bp)	220,403,942
GC content (%)	42.40
Percent of repetitive sequences (%)	10.41
Number of gene models	15,609

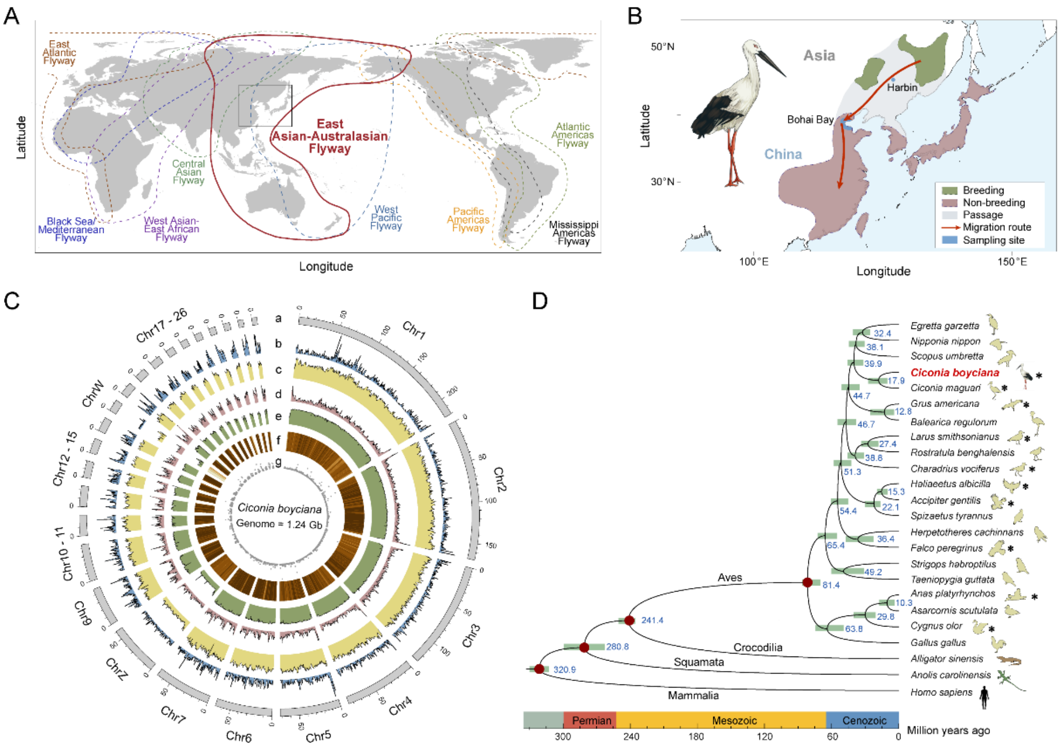

Figure 1: Landscape of the genome assembly and phylogenetic placement of the oriental stork. (A) Nine major flyways across the globe [6]. Black box indicated the range of oriental storks. (B) Breeding and wintering regions, migratory route and sampling site (Bohai Bay) of the wild oriental storks in this study. (C) Genomic features of the oriental stork. (a) The 26 chromosomes larger than 5 Mb. (b) Gene count. (c) Depth of Hi-C reads. (d) GC content density. (e) Depth of WGS reads. (f) Repeat number. (g) Depth of RNA reads. The statistics were calculated using a 500-kbp window. (D) Phylogenetic relationship of 24 species and the estimated divergence time. Asterisks represented full migrants while others are not migrants.

We identified 10.41% of genome sequences as repetitive elements (129.09 Mb), including LTRs (3.46%), LINEs (5.64%), DNA elements (0.73%), SINEs (0.13%) and unknown repeats (0.10%) (Supplementary Table S5-S7). After masking these repeat elements, a total of 15,609 protein-coding genes were predicted in our assembly, and the average gene length, intron length and exon length were 24.53 kb, 2.58 kb and 173.96 bp (9.84 exons per gene), respectively, which were comparable to other avian species (Supplementary Table S8, Supplementary Fig. S4). All

predicted genes (100%) were functionally annotated in at least one of the five databases we used (Supplementary Fig. S5, Supplementary Table S9). Additionally, 208 miRNA, 152 rRNA, 440 tRNA and 274 snRNA were predicted in this study (Supplementary Table S10). A phylogenetic tree was constructed based on 1800 shared single-copy gene families. Aves and Crocodilian were sister clades that diverged at c. 241.4 Mya, and the oriental stork split with *C. maguari* at c. 17.9 Mya (Fig. 1D, Supplementary Fig. S6).

Population structure, genetic diversity and inbreeding

In order to assess the genome-wide genetic background for the oriented stork population, we mapped paired-end sequencing data of 46 individuals (29 wild and 17 captive) to the assembled reference genome. Average sequencing coverage and depth for these individuals were 97.80% and 22.81-fold, respectively (Supplementary Table S11). After filtering low-quality variants and variants in sex chromosomes, we obtained 6,525,198 qualified SNPs across 33 autosomes.

PCA, admixture and phylogenetic tree all supported that wild individuals formed a single cluster while captive individuals were scattered (Fig. 2A and B). Five captive individuals including three individuals from Harbin, one Japan individual and one USA individual were clustered into the wild population, implying that the five individuals have very similar genetic background with the wild population. The lowest CV error for K=2 suggested there were two dominant ancestral components (Fig. 2B, Supplementary Fig. S7), and admixture analysis for larger K values revealed that wild individuals might have more complex ancestral components.

We further calculated the genetic diversity and inbreeding level for the oriental stork population, which were two essential indexes for the assessment of threatened status for species. Average genome-wide heterozygosity (H) of all 46 individuals was estimated to be $1.20\times10^{-3} \pm 6.32\times10^{-5}$, which was a relatively high level among endangered avian species (crested ibis: 4.30×10^{-4}[53]; saker falcon: 8.00×10^{-4}[64]; Chatham Island black robin: 4.80×10^{-4}[65]; and

kākāpō: 5.00×10^{-4}[66]) (Fig. 2C, Supplementary Table S12 and S13). The *H* for wild individuals was slightly higher than that of captive individuals ($H_{wild}=1.21\times10^{-3} \pm 2.04\times10^{-5}$; $H_{captive}=1.18\times10^{-3} \pm 9.72\times10^{-5}$), but with no significant difference. The nucleotide diversity (π) of the wild and the captive populations was also higher than brown eared pheasant (*Crossoptilon mantchuricum*, 9.60×10^{-5})[67] and green peafowl (*Pavo muticus*, 4.70×10^{-4}) [68] populations (Supplementary Fig. S8).

Although the oriental stork genomes presented a relatively high genome-wide genetic diversity, we still found runs of homozygosity (ROHs) fragments across the genome with an average $F_{ROH \geqslant 100\ kb}$ value of 5.56±5.30% (wild: 4.44±1.35%; captive: 7.47±8.19%) (Fig. 2D). Long ROH larger than 1 Mb was rare in these individuals with an average value of 2.29±4.86% (wild: 1.18±0.96%; captive: 4.18±7.54%). Significant difference was found between the wild and the captive populations on $F_{ROH \geqslant 1Mb}$ ($p = 0.04$). Unexpectedly, two captive individuals seemed to be highly inbred (F_{ROH}: ~30%), which was consistent with their lower *H* than others (Fig. 2C). Overall, the low-level F_{ROH} of oriental storks suggested a surprisingly low inbreeding risk, much lower than many other reported small populations of mammals, reptiles and avian [66, 69, 70].

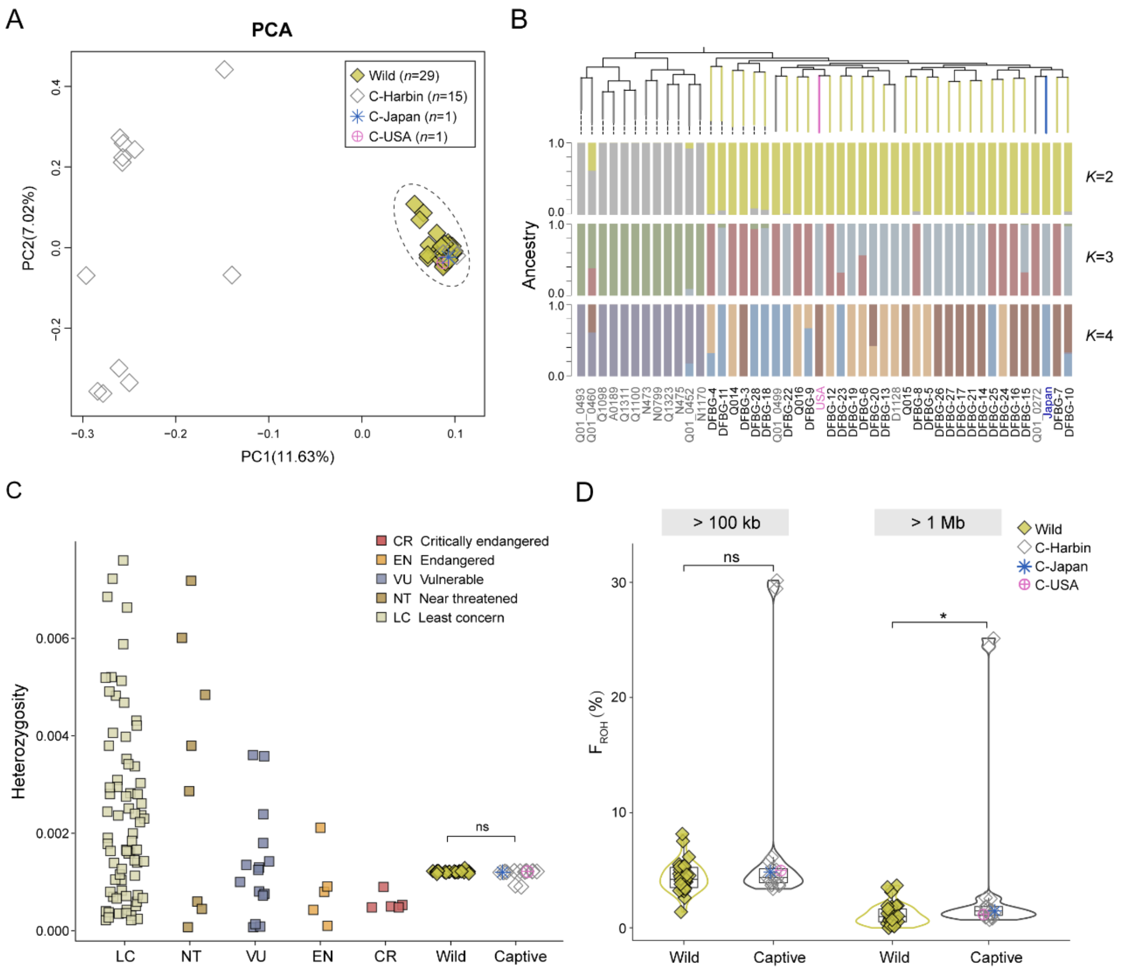

Figure 2: Population genetic structure, genome-wide heterozygosity, and inbreeding level of the oriental storks. (A) PCA analysis of 46 individuals by the first and second principal components. (B) Phylogenetic relationship and admixture analysis of the 46 individuals with a *K* value from 2 to 4 ancestral components. (C) Comparison of whole-genome heterozygosity in bird species with different threatened status defined by IUCN. (D) Individual inbreeding coefficients inferred by F_{ROH}. The F_{ROH} for ROH ⩾ 100 Kb and ROH ⩾ 1 Mb are shown here (two-sided *t*-test, ns = non-significant, **p* < 0.05).

Higher mutational load in the wild population

Mutational load is a genetic factor associated with fitness cost of a species. Mutational load was calculated in each individual and we generally identified a large number of sites carrying derived synonymous, missense and loss of function (LoF) mutations (Fig. 3A, Supplementary Fig. S9). We found that average values of both heterozygous and

homozygous derived alleles in the wild population were higher than that in captive individuals (Supplementary Table S14). In particularly, homozygous LoFs were significantly increased in the wild population (172.00±8.14) compared to the captive population (139.76±33.64). The LoF number scaled by synonymous mutation also supported higher frequencies in the wild population (Supplementary Fig. S10). As to putatively genes influenced by missense and LoF, respectively, 71.2% and 62.3% of them could be found in both of the wild and the captive populations, while the former showed an excess of private genes carrying these non-synonymous mutations (Supplementary Fig. S11A). Fortunately, for the unique genes harboring LoF mutations in the wild, we didn't find any GO terms that involved in life activities or fitness (Supplementary Fig. S11B).

Next, we explored the folded site frequency spectrum (SFS) of derived alleles in the oriental storks to detect the effect of genetic drift on the accumulation of mutations (Fig. 3B). Both the wild and the captive populations displayed "L-shaped" lines in the three categories of mutations, presenting a large fraction of rare derived alleles in both populations, which indicate the mutation drift equilibrium [71]. However, we observed the low-frequency alleles was relatively deficient while the medium-frequency was excessive in the captive population compared to the wild population, indicating that the genetic drift seemed to be slightly stronger in the captivity [67].

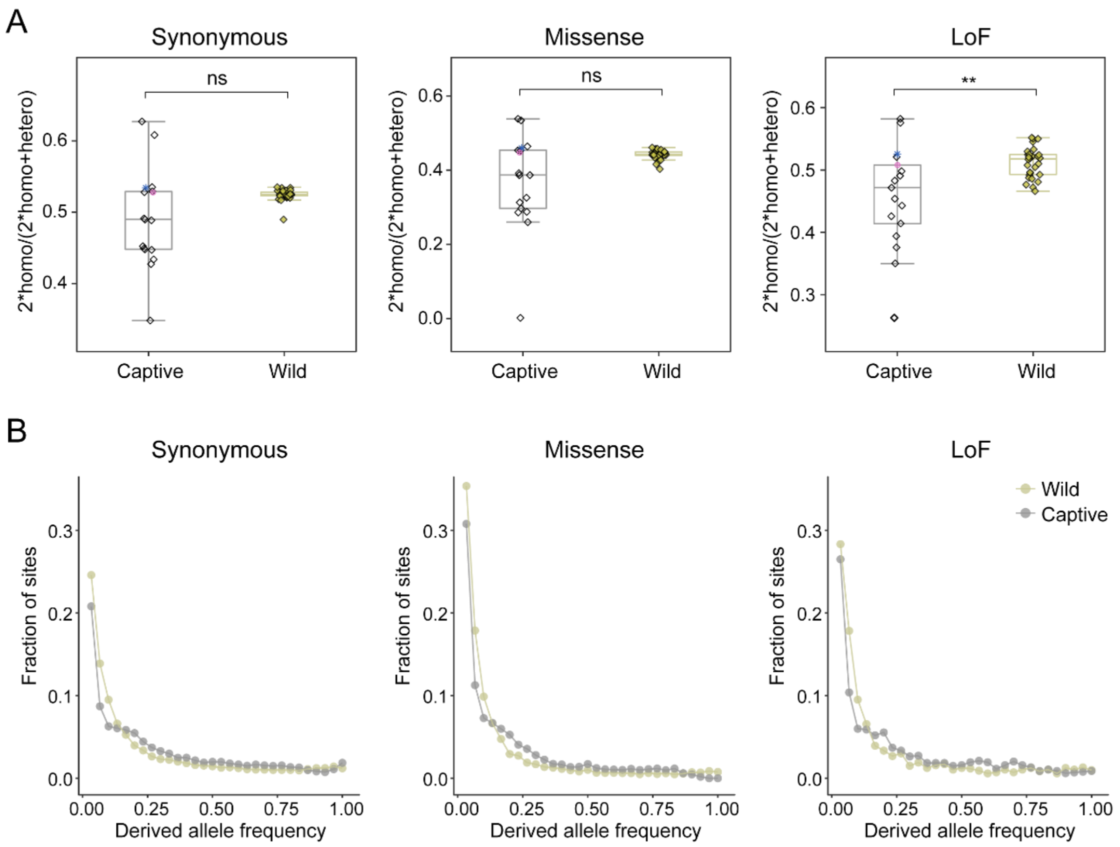

Figure 3: Mutational load of the wild and the captive populations. (A) Statistics of derived alleles, including synonymous, missense and LoF mutations. The ratio of homozygous derived alleles in each individual genome were shown here (two-sided *t*-test, ns = non-significant, ***p* < 0.01). (B) Folded SFS of synonymous, missense and LoF mutations of the wild and the captive populations. The proportion of loci (y axis) is shown for each derived allele frequency (x axis).

Historical population dynamics

The historical population dynamics is closely related to the accumulation of genetic load [72]. In order to evaluate the change of effective population sizes (N_e) of oriental storks over its evolutionary history, we reconstructed their demographic trajectory since 6 million years before present (BP). The whole population history of the oriental stork was characterized by two population expansion and two population decline. Wild population experienced the first increase at around 800-200 ka BP after a long steady period and then a serious decreasing occurred ca. 200-6 ka BP (Fig. 3A and B). After that, a slight recovery occurred at 6-3 ka BP. The most recent decrease started at 3 ka BP and

the final N_e dropped to approximately 1,000 (Fig. 3C). The N_e/N_c (census size, N_c) ratio was about 0.33 for the contemporary wild population, which fell within the range of most species (0.5-0.10) [73].

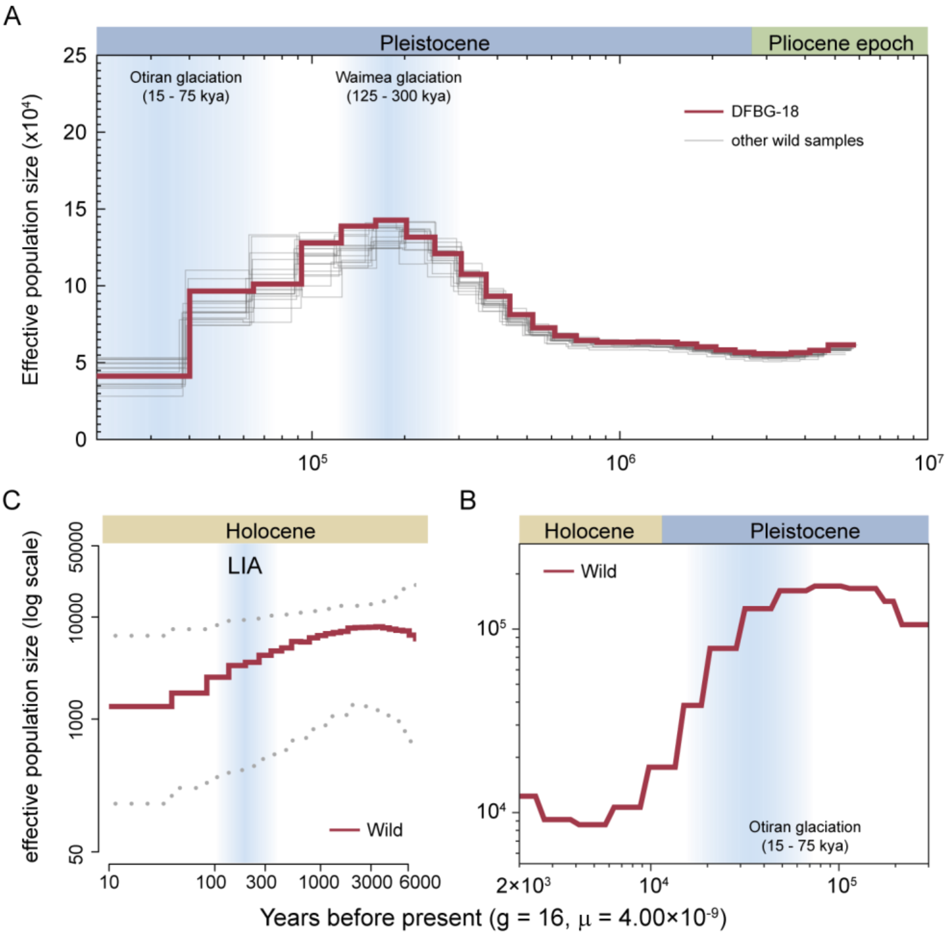

Figure 4: Estimated demographic history for the wild population of oriental stork. (A) Large-scale demographic fluctuation from 6 million years ago (Mya) to 20 ka BP inferred by PSMC for each wild individual. (B) Recent population history over the past 200-0.3 ka estimated by SMC++ with 29 wild individuals. (C) Recent effective population size for the wild inferred by PopSizeABC. Dotted lines indicate a 90% confidence interval. Light-blue shadows depict several glacial periods including Waimea glaciation, Otiran glaciation and the little ice age (LIA).

Genomic insights for bird migration

Long-distance migratory birds are under significant selective pressure from their early life [74]. The interplay between genetics, learning and spatial memory has a critical role in shaping the complex migration behavior [75, 76]. The ability of migration for the oriental stork is closely related with its survival, and a better understanding of the genomic basis of this biological adaptation is expected to facilitate the future conservation of this bird species.

We compared the oriental stork with other avian species with/without the migratory trait (Supplementary Fig. S12). We totally identified 526 expanded gene families, 107 PSGs, and 308 REGs in the oriental stork genome compared with un-migratory birds (uM group). The potential genes were locked to 557 genes in expanded families, 90 PSGs and 279 REGs when we used other full-migratory birds (M group) as comparisons to expectedly find more likely genomics signatures on the migration in the oriental stork (Supplementary Fig. S13). Therefore, only the genes that were outstanding when compared to the uM group but not presented when compared to the M group were more likely to be closely related to the genomic basis for the migratory trait of the oriental stork. We further found a portion of these genes having functions related to the migratory-related traits (Fig. 5A, Supplementary Table S15-S17). GO enrichment analysis of expanded gene families showed that a series of GO terms were enriched in sensory system development and peripheral nervous system development (Fig. 5B), which may be helpful to increase the sensitivity to environmental changes and transmit these signals to central nervous system. Notably, trigeminal nerve development (GO:0021559) was distinctly enriched here, which was previously proved to be vital for the map sense in night-migratory songbirds [77]. Of particular interest, radical pairs of cryptochromes are magnetically sensitive and CRY4 is responsible for the light-dependent magnetic compass in the night-migratory European robin [78]. Here, we found that CRY2 gene family was expanded in oriental stork while didn't show in non-migratory birds (Supplementary Fig. S14). Long-term potentiation in the hippocampus is closely related to memory and learning, which contributes to the migratory route formation in peregrine falcons [76]. As for the oriental stork, we found two PSGs (*SPG11* and *EPHA1*) and nine REGs (*ITGB3*, *NSUN5*, *KCTD16*, *PRKCI*, *ATAD1*, *EPHA1*, *GRM1*, *ADGRL3*,

and *NEXMIF*) involved in synaptic plasticity. *NSUN5* gene is essential in NMDAr-dependent long-term potentiation and *Nsun5*-KO mice showed spatial cognitive deficits [79]. *ATAD1* gene encodes ATPase family AAA domain-containing protein 1, which controls AMPA receptor (AMPAR) internalization that regulates synaptic activity. Absence of *ATAD1* would affect the amplitude of miniature excitatory postsynaptic currents and finally cause deficits in learning and memory [80].

For long-distance migrants, their breast or flight muscles are red muscles with a high concentration of the pigment myoglobin, more capillaries and mitochondria, which are slow muscles that could supply long periods of oxidative metabolism [81, 82]. Here, we identified genomic signals in genes related to heme (REG: *UROS*; expanded gene families: *CYC* and *EPO* genes) and muscle (expanded gene families: *TTN*, *ENB*, and *INPP5F* genes), which might be the genic basis of the muscle development. In terms of immunity, we identified several genes involving in the pathogen clearance, which was of vital importance to the innate immune response in a changing environment.

Additionally, we identified 10,312 SNPs under recent positive selection detected by iHS method based on population genomic sequencing data, and 25.92% of these SNPs were distributed within genic region (1,453 genes) (Fig. 5C, Supplementary Fig. S15). GO and KEGG enrichment analyses revealed a series of biological functions and pathways associated with the neurons, including learning and memory (GO:0007611), synaptic plasticity (GO:0048167), axon development (GO:0061564), glutamatergic synapse (hsa04724), dopaminergic synapse (hsa04728), GABAergic synapse (hsa04727) (Fig. 5D, Supplementary Table S18 and S19). Noteworthily, we observed that the well-known *ADCY8* gene was also presented in the oriental storks with a selected SNP, which was important to the long-distance migratory peregrine populations [76]. Besides of the *ADCY8* gene, there were another 12 recently positive selected genes related to the long-term potentiation pathway: four genes (*GRIA1*, *GRIN2A*, *CACNA1C* and *GRM1*) encoding membrane receptors, five genes (*PRKACB*, *PPP3CB*, *PLCB1*, *ITPR2* and *ITPR3*) responsible for intracellular signal transduction and three genes (*RPS6KA3*, *CREBBP* and *EP300*) affecting nucleus transcription activity, which

provided a stepstone to uncover the genetic factor in shaping the migratory route (Fig. 5E).

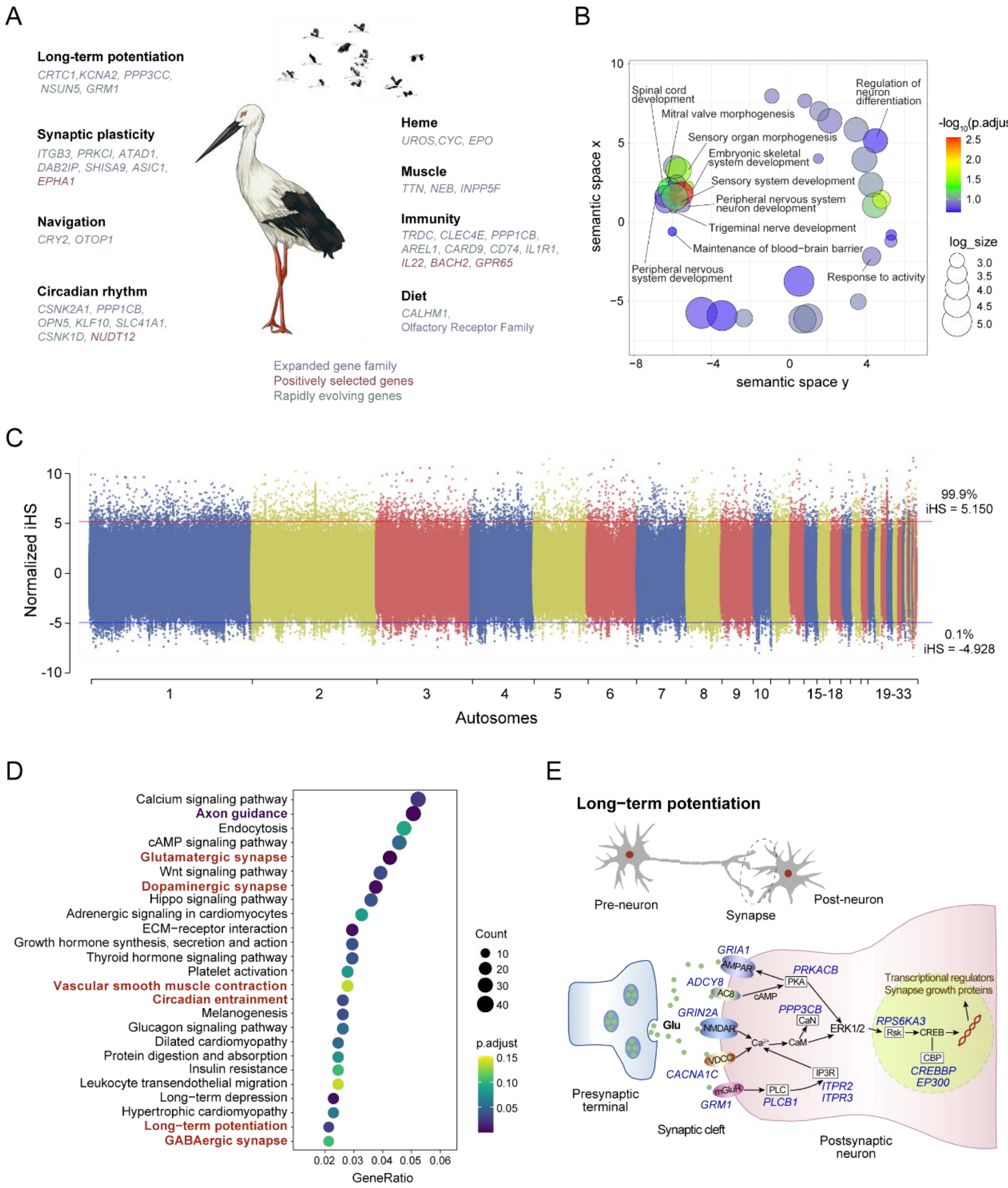

Figure 5: Genomic signatures relevant to migration in the oriental stork. (A) Genes or pathways that were detected may contribute to the migratory traits. (B) GO items representing biological process by REVIGO for expanded gene families in the oriental stork genome. Semantic similar GO terms clustered together. (C) Normalized iHS score indicating candidate SNPs under recent positive selection in the wild population. Red and blue lines represented the 99.9th and 0.1th quartile of iHS scores, respectively. (D) KEGG pathway analysis for recent positive selective genes. (E) Genes with recent positive selective signals that related to the long-term

potentiation pathway.

Discussion

Species are disappearing at an accelerated rate with the pervasive anthropogenic impacts [83, 84]. Record for the past 600 years reflected a peak of bird extinction rate in nineteenth century, firstly occurred at the Pacific islands [85], although avian is a relatively young clade undergoing fast substantial diversification [86]. Conservation efforts have been enhanced with the rapid development of genome sequencing technologies [87, 88]. However, genomic resources and relative genomic investigations are still lacking for many threatened species[89]. Here we assembled a high-quality reference genome and conducted a population resequencing for the endangered oriental stork, which would largely compensate the knowledge of the genetic background for both the wild and captive populations and further benefit the future conservation plans.

The genome assembly was consisted by 33 autosomes, Z and W chromosomes, which was consistent with the previous karyotypical study for the oriental stork (2n = 68) [90, 91]. Sex chromosomes and microchromosomes are relatively difficult to assemble and deserve special attention, and some synteny analysis could help confirm the accuracy of the assembly and the unanchored scaffolds like in the wood stork (*Mycteria americana*) study [92]. Here the Z chromosome might be not completely assembled in this study, because the assembled length was shorter than the reported karyotype. Microchromosomes were clearly divided into 23 contacted scaffold groups, showing the effectiveness of Hi-C data. Our assembled genome can be regarded as a new representatively reference genome and provided valuable genomic resource for the studies of ecology, evolution, and conservation of the oriental stork, as well as for other stock species.

The wild population of the oriental stork experienced serious decline in the last century and the population census size didn’t increase until in recent several years under conservation efforts. It is still classified as an “endangered”

species, with no more than 2500 mature individuals and more than 95% individuals live in a single subpopulation [15]. Consistently, we didn't detect any genetic structure within the wild population, and fortunately, with high genetic diversity and low inbreeding level (F_{ROH} < 10%), which may result from their migration every year along the EAAF, in contrast to some geographically isolated and highly inbred animal populations [66, 93]. Interestingly, we identified two highly inbred captive individuals (F_{ROH}≈30%) with decreased heterozygosity, and a significant higher level of long ROH fragments (>1Mb), which was more likely originated from the recent inbreeding. This phenomenon alarmed the importance of the scientific pedigree management in the small breeding populations with limited founders.

Endangered species with different historical population dynamics faced different extinction risk caused by relative genetic factors, claiming the necessity of introducing genomic parameters to precisely assess the threatened status of a species [94, 95]. It has been demonstrated that small populations experiencing long-term declines would have low genetic diversity and high-level inbreeding. On the contrary, a recent rapid decline has less effects on the genetic diversity [96]. For the oriental stork, the wild population increased during 6-3 ka BP and started to decline at 3 ka BP, approximately 187 generations(g) ago, explaining the relatively high genetic diversity of the wild population nowadays. The relatively large N_e (from 10,000 to 1,000) over the recent 3 ky also help avoid inbreeding and reduce the power of genetic drift in the wild population. The relatively high genetic diversity and a recent decline history supported that the oriental stork population has strong genetic capacity to recover. The rapid recent decline of the oriental stork might be resulted from both of the climate change and the human activities. Their breeding area, the Amur River basin, spanned Russia, China and Mongolia, was originally covered by temperate forests and populated by a relatively small number of nomadic people living on hunting and fishing. Around 3 kya, the climate in this region turned cold and dry from warm and humid. The forest system converted to wetlands and thus, the nest trees for the oriental storks became much less [97]. As the human society moved from the Bronze Age to the Iron Age at

2.6 kya, the increased human activities might also drove the bird population decline and habitat degeneration [97]. Over the past several centuries, the intensity of human activities has largely increased. The colonization of Russia in the 17th century brought modern agriculture and economic development [98]. Agricultural activities in the northeast China started from two waves of exploitation in the Liao-Jin period and in Qing Dynasty [99]. Human settlements in this region experienced the most rapid expansion in the past 100 years with large-scale agricultural reclamation process in the China side of the Amur River basin [100]. Wetlands in the Sanjiang plain have declined in area by 86% from 108,900 km^2 prior to the 20th century to some 14,800 km^2 in 2000 [101]. Moreover, the most important stop-over site of the oriental storks, the Bohai Bay, is an area with the most concentrated coastal reclamation activities in China [102]. Natural wetlands experienced striking environmental changes driven by rapid industrialization and urbanization in the last century [103]. Habitat degradation due to recent intensive human activities may explain the serious decline of the oriental stork population, also for other birds along the EAAF.

Migrants may adaptively adjust migratory routes and wintering areas in response to climate change and anthropogenic influence [104]. Improved evolutionary insights on the migration can facilitate the conservation for migratory birds. Billions of animals migrate annually across the planet, in pursuit of improved foraging opportunities, safety and reproductive output [105]. Migrants face selective pressure to arrive early to occupy high-quality territories at the stop-over/wintering sites [106]. Efficient migration requires coordinated support of the brain function and physical conditions [76, 78, 107]. Specific phenotypes and physiological functions are often hypothesized to be attributed to the evolution of the underlying protein-coding genes [108, 109]. We detected a series of expanded gene families, PSGs, and REGs that related to learning and memory, navigation, muscle development and energy metabolism in the oriental stork. Recent work has revealed that adult birds shape their migratory route through individual exploring and learning over a lifetime [75]. Long-term potentiation is critical for the large-scale spatial memory. For example, the long-distance migratory population of peregrine falcon experienced distinct

selection on *ADCY8* gene, which regulates downstream memory-related genes [76]. Here, we also found that *ADCY8* gene, together with other genes along the long-term potentiation pathway, was naturally selected in the oriental storks, and several pathways of synapse were overrepresented. These genomic signatures provide new insights into the migratory performance of birds, the flexible capability from evolution to adapt to the environmental changes.

Data Availability

The final genome assembly data, RNA-seq data and raw resequencing genome data are available in the NCBI BioProject repository (accession number: PRJNA1036389).

Additional Files

Supplementary Fig. S1. The distribution of 21-mer for estimation the genome size of the oriental stork.

Supplementary Fig. S2. Heatmap of Hi-C chromosomal interaction density among all 35 chromosomes.

Supplementary Fig. S3. Identification of sex chromosomes in the oriental stork genome.

Supplementary Fig. S4. Comparison of gene characteristics of avian species and human.

Supplementary Fig. S5. Venn diagram representing the functional annotation of the oriental stork gene set.

Supplementary Fig. S6. Comparison of the gene repertoires of 24 vertebrate genomes.

Supplementary Fig. S7. Cross validation (CV) error in the ADMIXTURE analysis.

Supplementary Fig. S8. Nucleotide diversity (π) across 25 autosomes in wild and captive populations, respectively by sliding a 5-Mb window.

Supplementary Fig. S9. Statistics of the number of heterozygous and homozygous for synonymous, missense and LoF mutations in each individual.

Supplementary Fig. S10. Statistics of the frequency of heterozygous, homozygous and site for missense and LoF mutations, scaled by synonymous mutations.

Supplementary Fig. S11. Genes harbored putatively missense and LoF mutations.

Supplementary Fig. S12. The expanded and contracted gene families in each bird genome and the comparison of their gene repertoires.

Supplementary Fig. S13. Comparison of detected genomic signals in the oriental stork genome compared with un-migratory birds and migratory birds, including expanded families, positively selected genes and rapidly evolving genes.

Supplementary Fig. S14. The phylogenetic tree of *CRY2* gene constructed by the maximum likelihood method. The red clade represented three *CRY2* gene in oriental stork.

Supplementary Fig. S15. Venn diagram for four methods to identify genes affected by candidate SNPs under recent selection.

Supplementary Table. S1. Statistics of sequencing data for genome assembly and gene annotation.

Supplementary Table. S2. Genomic statistics of the *C. boyciana* assembly.

Supplementary Table. S3. BUSCO analysis of the whole genome and the gene set of the *C. boyciana* genome.

Supplementary Table. S4. Statistics of sequencing data mapped to the *C. boyciana* genome which were used for the assembly and gene annotation.

Supplementary Table. S5. Transposable elements (TEs) statistics in our assembly.

Supplementary Table. S6. Statistics of identified Repeats by *De* novo method in *C. boyciana* genome.

Supplementary Table. S7. Transposable elements in the *C. boyciana* assembly.

Supplementary Table. S8. Statistics of annotations for the *C. boyciana* gene set.

Supplementary Table. S9. Statistics on functional annotation of the *C. boyciana* gene set.

Supplementary Table. S10. Statistics of ncRNA annotation.

Supplementary Table. S11. List of 46 oriental stork samples used for re-sequencing.

Supplementary Table. S12. Whole-genome heterozygosity and F_{ROH} of 46 re-sequenced samples.

Supplementary Table. S13. Whole-genome heterozygosity for published birds presented in Fig. 2C.

Supplementary Table. S14. Average number of heterozygous, homozygous and all sites for derived alleles in wild and captive populations.

Supplementary Table. S15. Expanded gene families in *C. boyciana* genome compared with un-migratory birds.

Supplementary Table. S16. Rapidly evolving genes in *C. boyciana* genome compared with un-migratory birds.

Supplementary Table. S17. Positively selected genes in *C. boyciana* genome compared with un-migratory birds.

Supplementary Table. S18. GO enrichment of genes directly affected by positively selected SNPs.

Supplementary Table. S19. KEGG pathway enrichment of genes directly affected by positively selected SNPs.

Abbreviations

IBA: Important Bird and Biodiversity Area; EAAF: East Asian-Australasian Flyway; IUCN: International Union for Conservation of Nature; ONT: Oxford Nanopore Technology; Hi-C: high-throughput chromosome

conformation capture; WGS: whole genome sequencing; Kb: kilobase pairs; BUSCO: Benchmarking Universal Single-Copy Orthologs; RNA-seq: RNA sequencing; BWA: Burrows-Wheeler aligner; gVCF: genomic Variant Call Format; SNP: single-nucleotide polymorphism; PCA: principal component analysis; cross-validation: CV; iHS: integrated haplotype score; *H*: heterozygosity; ROH: run of homozygosity; LoF: loss of function; PSMC: Pairwise Sequentially Markovian Coalescent; ABC: approximate Bayesian computation; MAF: minor allele frequency; GO: Gene ontology; KEGG: Kyoto Encyclopedia of Genes and Genomes; BLAST: Basic Local Alignment Search Tool; Gb: gigabase pairs; Mb: megabase pairs; Chr: chromosome; SFS: site frequency spectrum; N_e: the effective size; N_c: the census size; PSG: positively selected gene; REG: rapidly evolving gene; LTR: long terminal repeat; LINE: long interspersed nuclear element; SINE: short interspersed nuclear element; BP: before present; LIA: little ice age; *CRY2*: Cryptochrome Circadian Regulator 2; NCBI: National Center for Biotechnology Information.

Competing Interests

The authors declare no competing interests.

Funding

This work was financially supported by funding from Surveillance of Wildlife Diseases from the State Forestry Administration of China (2023057) and the Leading Talent Project of "Science and Technology Leading Talent Team Project of Inner Mongolia Autonomous Region (2022LJRC0010).

Acknowledgments

We thank all staffs for their work in the collection of animal samples

Authors' Contributions

T.L. and Z.H. conceived and designed the research. H.L., M.Z., S.W. and X.Z. organized and collected the samples. M.S., L.H., M.Y., Y.L. and B.L. prepared the sequencing library. J.C. and H.L. performed genome assembly and annotation. S.Y., Y.L. and X.Z. conducted comparative genomic analysis and population genetic analysis. S.Y. wrote the manuscript. Y.X. and T.L. extensively revised the manuscript. T.L. and Z.H. supervised the study. All authors have read and approved the final manuscript.

References

1. Johnson CN, Balmford A, Brook BW, Buettel JC, Galetti M, Guangchun L, et al. Biodiversity losses and conservation responses in the Anthropocene. Science. 2017;356 6335:270-5. doi:doi:10.1126/science.aam9317.
2. Paez S, Kraus RHS, Shapiro B, Gilbert MTP, Jarvis ED, Group VGPC, et al. Reference genomes for conservation. 2022;377 6604:364-6. doi:doi:10.1126/science.abm8127.
3. Gregory RD, Noble D, Field R, Marchant J, Raven M and Gibbons D. Using birds as indicators of biodiversity. Ornis hungarica. 2003;12 13:11-24.
4. Kirby JS, Stattersfield AJ, Butchart SHM, Evans MI, Grimmett RFA, Jones VR, et al. Key conservation issues for migratory land- and waterbird species on the world's major flyways. Bird Conservation International. 2008;18 S1:S49-S73. doi:10.1017/S0959270908000439.
5. International B. State of the World's Birds 2018—taking the pulse of the planet. Cambridge, UK: BirdLife International. 2018.
6. Boere GC and Piersma T. Flyway protection and the predicament of our migrant birds: A critical look at international conservation policies and the Dutch Wadden Sea. Ocean & Coastal Management. 2012;68:157-68. doi:https://doi.org/10.1016/j.ocecoaman.2012.05.019.
7. Si Y, Xu F, Wei J, Zhang L, Murray N, Yang R, et al. A systematic network-based migratory bird monitoring and protection system is needed in China. Sci Bull (Beijing). 2021;66 10:955-7.
8. Ma Z, Melville DS, Liu J, Ying C, Yang H, Ren W, et al. Rethinking China's new great wall. Science. 2014;346 6212:912-4.
9. Li J, Hughes AC and Dudgeon D. Correction: Mapping wader biodiversity along the East Asian-Australasian flyway. PLoS One. 2019;14 4:e0215877. doi:10.1371/journal.pone.0215877.
10. Bamford M, Watkins D, Bancroft W, Tischler G and Wahl J. Migratory shorebirds of the East Asian-Australasian flyway : Population estimates and internationally important sites. . Canberra: Wetlands International, Oceania. 2008:pp 237.
11. Zheng H, Shen G, Shang L, Lv X, Wang Q, McLaughlin N, et al. Efficacy of conservation strategies for endangered oriental white storks (Ciconia

boyciana) under climate change in Northeast China. Biological Conservation. 2016;204:367-77. doi:https://doi.org/10.1016/j.biocon.2016.11.004.

12. Cano-Alonso LS, Grace MK, Yu Y-t and Chan S. Reversing the Decline in a Threatened Species: The Black-Faced Spoonbill Platalea minor. Diversity. 2023;15 2:217.

13. Gilbert M, Buuveibaatar B, Fine AE, Jambal L and Strindberg S. Declining breeding populations of White-naped Cranes in Eastern Mongolia, a ten-year update. Bird Conservation International. 2016;26 4:490-504. doi:10.1017/S0959270915000301.

14. Xu W, Xiao Y, Zhang J, Yang W, Zhang L, Hull V, et al. Reply to Yang et al.: Coastal wetlands are not well represented by protected areas for endangered birds. PNAS. 2017;114 28:E5493-E. doi:doi:10.1073/pnas.1706515114.

15. International B. The IUCN Red List of Threatened Species 2018: e.T22697695A131942061. 2018; doi:https://dx.doi.org/10.2305/IUCN.UK.2018-2.RLTS.T22697695A131942061.en.

16. Garidi, Fan SJ, Cao L, Zhang BX, Wang YX, Zhu BG, et al. Migration strategy of the Bohai Bay wintering population of juvenile Oriental Storks (Ciconia boyciana). Biodiversity Science. 2022;30 5:21232. doi:10.17520/biods.2021232.

17. Ma X. *Research on captive oriental white stork (Ciconia boyciana) in energy of digestion and fledgling growth*. Northeast Forestry University, 2007.

18. Liu Z and Li X. The research progress of oriental white stork. Territory & Natural Resources Study. 2008; 01:77-8. doi:10.16202/j.cnki.tnrs.2008.01.001.

19. Zeng S, Cheng L and Li X. The numerical distribution and conservation of oriental white stork in China. Territory & Natural Resources Study. 2003; 01:71-2. doi:10.16202/j.cnki.tnrs.2003.01.035.

20. Van den Bossche W, Berthold P, Darman Y, Andronov V, Parilov M and Querner U. Satellite-tracking helps to discover stopover sites of the threatened Oriental White Stork (Ciconia boyciana). Microwave Telemetry, Inc Newsletter. 2001;2 1:3-4.

21. Lieberman-Aiden E, Berkum NV, Williams L, Imakaev M, Ragoczy T, Telling A, et al. Comprehensive Mapping of Long-Range Interactions Reveals Folding Principles of the Human Genome. Science. 2009;326 5950:289.

22. Lander ES and Waterman MS. Genomic mapping by fingerprinting random clones: a mathematical analysis. Genomics. 1988;2 3:231-9. doi:10.1016/0888-7543(88)90007-9.

23. Hu J, Fan J, Sun Z and Liu S. NextPolish: a fast and efficient genome polishing tool for long-read assembly. Bioinformatics. 2020;36 7:2253-5. doi:10.1093/bioinformatics/btz891.

24. Li H and Durbin R. Fast and accurate long-read alignment with Burrows - Wheeler transform. Bioinformatics. 2010;26 5:589-95. doi:10.1093/bioinformatics/btp698.

25. Durand NC, Shamim MS, Machol I, Rao SS, Huntley MH, Lander ES, et al. Juicer provides a one-click system for analyzing loop-resolution Hi-C experiments. Cell systems. 2016;3 1:95-8.
26. Manni M, Berkeley MR, Seppey M, Simão FA and Zdobnov EM. BUSCO Update: Novel and Streamlined Workflows along with Broader and Deeper Phylogenetic Coverage for Scoring of Eukaryotic, Prokaryotic, and Viral Genomes. Molecular biology and evolution. 2021;38 10:4647-54. doi:10.1093/molbev/msab199.
27. Xu Z and Wang H. LTR_FINDER: an efficient tool for the prediction of full-length LTR retrotransposons. Nucleic Acids Research. 2007;35 suppl_2:W265-W8. doi:10.1093/nar/gkm286 %J Nucleic Acids Research.
28. Han Y and Wessler SR. MITE-Hunter: a program for discovering miniature inverted-repeat transposable elements from genomic sequences. Nucleic Acids Research. 2010;38 22:e199-e. doi:10.1093/nar/gkq862 %J Nucleic Acids Research.
29. Flynn JM, Hubley R, Goubert C, Rosen J, Clark AG, Feschotte C, et al. RepeatModeler2 for automated genomic discovery of transposable element families. Proceedings of the National Academy of Sciences. 2020;117 17:9451-7.
30. Tarailo-Graovac M and Chen N. Using RepeatMasker to identify repetitive elements in genomic sequences. Current protocols in bioinformatics. 2009;Chapter 4:Unit 4.10. doi:10.1002/0471250953.bi0410s25.
31. Jurka J, Kapitonov VV, Pavlicek A, Klonowski P, Kohany O and Walichiewicz J. Repbase Update, a database of eukaryotic repetitive elements. Cytogenetic and genome research. 2005;110 1-4:462-7. doi:10.1159/000084979.
32. Benson G. Tandem repeats finder: a program to analyze DNA sequences. Nucleic acids research. 1999;27 2:573-80. doi:10.1093/nar/27.2.573.
33. Korf I. Gene finding in novel genomes. BMC Bioinformatics. 2004;5 1:59. doi:10.1186/1471-2105-5-59.
34. Majoros WH, Pertea M and Salzberg SL. TigrScan and GlimmerHMM: two open source ab initio eukaryotic gene-finders. Bioinformatics. 2004;20 16:2878-9. doi:10.1093/bioinformatics/bth315 %J Bioinformatics.
35. Keller O, Kollmar M, Stanke M and Waack S. A novel hybrid gene prediction method employing protein multiple sequence alignments. Bioinformatics. 2011;27 6:757-63. doi:10.1093/bioinformatics/btr010.
36. Bolger AM, Lohse M and Usadel B. Trimmomatic: a flexible trimmer for Illumina sequence data. Bioinformatics. 2014;30 15:2114-20. doi:10.1093/bioinformatics/btu170 %J Bioinformatics.
37. Haas BJ, Papanicolaou A, Yassour M, Grabherr M, Blood PD, Bowden J, et al. De novo transcript sequence reconstruction from RNA-seq using the Trinity platform for reference generation and analysis. Nature Protocols. 2013;8 8:1494-512. doi:10.1038/nprot.2013.084.
38. Haas BJ, Salzberg SL, Zhu W, Pertea M, Allen JE, Orvis J, et al. Automated eukaryotic gene structure annotation using EVidenceModeler and the Program

to Assemble Spliced Alignments. Genome Biology. 2008;9 1:R7-R22. doi:10.1186/gb-2008-9-1-r7.

39. Mount DW. Using the Basic Local Alignment Search Tool (BLAST). CSH protocols. 2007;2007:pdb.top17. doi:10.1101/pdb.top17.

40. Birney E, Clamp M and Durbin R. GeneWise and Genomewise. Genome research. 2004;14 5:988-95. doi:10.1101/gr.1865504.

41. Campbell MS, Holt C, Moore B and Yandell M. Genome Annotation and Curation Using MAKER and MAKER-P. Current Protocols Bioinformatics. 2014;48 1:4.11.1-4..39. doi:https://doi.org/10.1002/0471250953.bi0411s48.

42. Altschul SF, Gish W, Miller W, Myers EW and Lipman DJ. Basic local alignment search tool. Journal of molecular biology. 1990;215 3:403-10.

43. Wang Y, Tang H, Debarry JD, Tan X, Li J, Wang X, et al. MCScanX: a toolkit for detection and evolutionary analysis of gene synteny and collinearity. Nucleic acids research. 2012;40 7:e49. doi:10.1093/nar/gkr1293.

44. Krzywinski M, Schein J, Birol I, Connors J, Gascoyne R, Horsman D, et al. Circos: An information aesthetic for comparative genomics. Genome Research. 2009;19:1639-45.

45. Lam-Tung N, Schmidt HA, Arndt VH, Quang MB and Evolution. IQ-TREE: A Fast and Effective Stochastic Algorithm for Estimating Maximum-Likelihood Phylogenies. Molecular Biology and Evolution. 2015;32 1:268-74.

46. Yang Z. PAML 4: Phylogenetic Analysis by Maximum Likelihood. Molecular Biology and Evolution. 2007;24 8:1586 - 91.

47. Benton MJ and Donoghue PC. Paleontological evidence to date the tree of life. Molecular biology and evolution. 2007;24 1:26-53. doi:10.1093/molbev/msl150.

48. Freed D, Aldana R, Weber JA and Edwards JS. The Sentieon Genomics Tools - A fast and accurate solution to variant calling from next-generation sequence data. bioRxiv. 2017:115717. doi:10.1101/115717 %J bioRxiv.

49. Danecek P, Auton A, Abecasis G, Albers CA, Banks E, DePristo MA, et al. The variant call format and VCFtools. Bioinformatics. 2011;27 15:2156-8. doi:10.1093/bioinformatics/btr330.

50. Chang CC, Chow CC, Tellier LC, Vattikuti S, Purcell SM and Lee JJ. Second-generation PLINK: rising to the challenge of larger and richer datasets. Gigascience. 2015;4:7. doi:10.1186/s13742-015-0047-8.

51. Alexander DH, Novembre J and Lange K. Fast model-based estimation of ancestry in unrelated individuals. Genome Research. 2009;19 9:1655-64.

52. Li H, Handsaker B, Wysoker A, Fennell T, Ruan J, Homer N, et al. The Sequence Alignment/Map format and SAMtools. Bioinformatics. 2009;25 16:2078-9. doi:10.1093/bioinformatics/btp352.

53. Feng S, Fang Q, Barnett R, Li C, Han S, Kuhlwilm M, et al. The Genomic Footprints of the Fall and Recovery of the Crested Ibis. Current Biology. 2019;29:340-9. doi:10.1016/j.cub.2018.12.008.

54. Li H and Durbin R. Inference of human population history from individual whole-genome sequences. Nature. 2011;475 7357:493-6.

doi:10.1038/nature10231.

55. Terhorst J, Kamm JA and Song YS. Robust and scalable inference of population history from hundreds of unphased whole genomes. Nature Genetics. 2016;49 2:303-9. doi:10.1038/ng.3748.

56. Boitard S, Rodriguez W, Jay F, Mona S and Austerlitz F. Inferring Population Size History from Large Samples of Genome-Wide Molecular Data - An Approximate Bayesian Computation Approach. PLoS Genetics. 2016;12 3:e1005877. doi:10.1371/journal.pgen.1005877.

57. Li H, Coghlan A, Ruan J, Coin LJ, Hériché J-K, Osmotherly L, et al. TreeFam: a curated database of phylogenetic trees of animal gene families. Nucleic Acids Research. 2006;34 suppl_1:D572-D80. doi:10.1093/nar/gkj118 %J Nucleic Acids Research.

58. De Bie T, Cristianini N, Demuth JP and Hahn MW. CAFE: a computational tool for the study of gene family evolution. Bioinformatics. 2006;22 10:1269-71. doi:10.1093/bioinformatics/btl097 %J Bioinformatics.

59. Team RDC. R: A language and environment for statistical computing. R Foundation for Statistical Computing. 2012.

60. Wu T, Hu E, Xu S, Chen M, Guo P, Dai Z, et al. clusterProfiler 4.0: A universal enrichment tool for interpreting omics data. The Innovation. 2021;2 3:100141. doi:10.1016/j.xinn.2021.100141.

61. Supek F, Bošnjak M, Škunca N and Šmuc T. REVIGO summarizes and visualizes long lists of gene ontology terms. PLoS One. 2011;6 7:e21800. doi:10.1371/journal.pone.0021800.

62. Browning BL, Zhou Y and Browning SR. A One-Penny Imputed Genome from Next-Generation Reference Panels. The American Journal of Human Genetics. 2018;103 3:338-48. doi:10.1016/j.ajhg.2018.07.015.

63. Voight BF, Kudaravalli S, Wen X and Pritchard JK. A map of recent positive selection in the human genome. PLoS Biology. 2006;4 3:e72. doi:10.1371/journal.pbio.0040072.

64. Zhan X, Pan S, Wang J, Dixon A, He J, Muller MG, et al. Peregrine and saker falcon genome sequences provide insights into evolution of a predatory lifestyle. Nature Genetics. 2013;45 5:563-6. doi:10.1038/ng.2588.

65. von Seth J, van der Valk T, Lord E, Sigeman H, Olsen R-A, Knapp M, et al. Genomic trajectories of a near-extinction event in the Chatham Island black robin. BMC Genomics. 2022;23 1:747. doi:10.1186/s12864-022-08963-1.

66. Dussex N, van der Valk T, Morales HE, Wheat CW, Díez-del-Molino D, von Seth J, et al. Population genomics of the critically endangered kākāpō. Cell Genomics. 2021;1 doi:10.1016/j.xgen.2021.100002.

67. Wang P, Burley JT, Liu Y, Chang J, Chen, Lu Q, et al. Genomic Consequences of Long-Term Population Decline in Brown Eared Pheasant. Molecular Biology and Evolution. 2021;38 1:263-73. doi:10.1093/molbev/msaa213.

68. Dong F, Kuo H-C, Chen G-L, Wu F, Shan P-F, Wang J, et al. Population genomic, climatic and anthropogenic evidence suggest the role of human forces in endangerment of green peafowl. Proceedings of the Royal Society

B: Biological sciences. 2021;288 1948:20210073. doi:doi:10.1098/rspb.2021.0073.

69. Seth Jv, Dussex N, Diez-Del-Molino D, van der Valk T, Kutschera VE, Kierczak M, et al. Genomic insights into the conservation status of the world's last remaining Sumatran rhinoceros populations. Nature Communications. 2021;12:2393. doi:10.1038/s41467-021-22386-8.

70. Yang S, Lan T, Zhang Y, Wang Q, Li H, Dussex N, et al. Genomic investigation of the Chinese alligator reveals wild-extinct genetic diversity and genomic consequences of their continuous decline. Molecular Ecology Resources. 2022:1-18. doi:https://doi.org/10.1111/1755-0998.13702.

71. Khan A, Patel K, Shukla H, Viswanathan A, van der Valk T, Borthakur U, et al. Genomic evidence for inbreeding depression and purging of deleterious genetic variation in Indian tigers. Proceedings of the National Academy of Sciences of the United States of America. 2021;118 49 doi:10.1073/pnas.2023018118.

72. Bertorelle G, Raffini F, Bosse M, Bortoluzzi C, Iannucci A, Trucchi E, et al. Genetic load: genomic estimates and applications in non-model animals. Nature reviews Genetics. 2022;23 8:492-503. doi:10.1038/s41576-022-00448-x.

73. Hare MP, Nunney L, Schwartz MK, Ruzzante DE, Burford M, Waples RS, et al. Understanding and estimating effective population size for practical application in marine species management. Conservation biology. 2011;25 3:438-49. doi:10.1111/j.1523-1739.2010.01637.x.

74. Brønnvik H, Safi K, Vansteelant WM, Byholm P and Nourani E. Experience does not change the importance of wind support for migratory route selection by a soaring bird. Royal Society Open Science. 2022;9 12:220746.

75. Aikens EO, Nourani E, Fiedler W, Wikelski M and Flack A. Learning shapes the development of migratory behavior. Proceedings of the National Academy of Sciences. 2024;121 12:e2306389121. doi:doi:10.1073/pnas.2306389121.

76. Gu Z, Pan S, Lin Z, Hu L, Dai X, Chang J, et al. Climate-driven flyway changes and memory-based long-distance migration. Nature. 2021;591 7849:259-64. doi:10.1038/s41586-021-03265-0.

77. Kishkinev D, Chernetsov N, Heyers D and Mouritsen H. Migratory reed warblers need intact trigeminal nerves to correct for a 1,000 km eastward displacement. PLoS One. 2013;8 6:e65847.

78. Xu J, Jarocha LE, Zollitsch T, Konowalczyk M, Henbest KB, Richert S, et al. Magnetic sensitivity of cryptochrome 4 from a migratory songbird. Nature. 2021;594 7864:535-40. doi:10.1038/s41586-021-03618-9.

79. Zhang T, Chen P, Li W, Sha S, Wang Y, Yuan Z, et al. Cognitive deficits in mice lacking Nsun5, a cytosine-5 RNA methyltransferase, with impairment of oligodendrocyte precursor cells. Glia. 2019;67 4:688-702. doi:https://doi.org/10.1002/glia.23565.

80. Zhang J, Wang Y, Chi Z, Keuss MJ, Pai YM, Kang HC, et al. The AAA+ ATPase Thorase regulates AMPA receptor-dependent synaptic plasticity and behavior. Cell. 2011;145 2:284-99. doi:10.1016/j.cell.2011.03.016.

81. Cassens RG and Cooper CC. Red and White Muscle. In: Chichester CO, Mrak EM and Stewart GF, editors. Advances in Food Research. Academic Press; 1971. p. 1-74.

82. Barge L and Mark EW. Muscle fiber types in a migratory and a non-magratory avian species. In: 2012.

83. Di Marco M, Venter O, Possingham HP and Watson JEM. Changes in human footprint drive changes in species extinction risk. Nature Communications. 2018;9 1:4621. doi:10.1038/s41467-018-07049-5.

84. Pimm SL, Jenkins CN, Abell R, Brooks TM, Gittleman JL, Joppa LN, et al. The biodiversity of species and their rates of extinction, distribution, and protection. Science. 2014;344 6187:1246752. doi:doi:10.1126/science.1246752.

85. Lees AC, Haskell L, Allinson T, Bezeng SB, Burfield IJ, Renjifo LM, et al. State of the World's Birds. Annual Review of Environment and Resources. 2022;47 1:231-60. doi:10.1146/annurev-environ-112420-014642.

86. Green RE, Braun EL, Armstrong J, Earl D, Nguyen N, Hickey G, et al. Three crocodilian genomes reveal ancestral patterns of evolution among archosaurs. Science. 2014;346 6215:1254449. doi:10.1126/science.1254449.

87. Formenti G, Theissinger K, Fernandes C, Bista I, Bombarely A, Bleidorn C, et al. The era of reference genomes in conservation genomics. Trends in ecology & evolution. 2022;37 3:197-202. doi:10.1016/j.tree.2021.11.008.

88. Ouborg NJ, Pertoldi C, Loeschcke V, Bijlsma RK and Hedrick PW. Conservation genetics in transition to conservation genomics. Trends in genetics : TIG. 2010;26 4:177-87. doi:10.1016/j.tig.2010.01.001.

89. Kitts PA, Church DM, Thibaud-Nissen F, Choi J, Hem V, Sapojnikov V, et al. Assembly: a resource for assembled genomes at NCBI. Nucleic acids research. 2016;44 D1:D73-80. doi:10.1093/nar/gkv1226.

90. Takagi N and Sasaki M. A phylogenetic study of bird karyotypes. Chromosoma. 1974;46 1:91-120. doi:10.1007/BF00332341.

91. Francisco M and Galetti Jr P. First karyotypical description of two American Ciconiiform birds, Mycteria americana (Ciconiidae) and Platalea ajaja (Threskiornithidae) and its significance for the chromosome evolutionary and biological conservation approaches. Genetics and Molecular Biology. 2000;23 4:799-801. doi:10.1590/S1415-47572000000400015.

92. Flamio R, Jr. and Ramstad KM. Chromosome-level genome of the wood stork (Mycteria americana) provides insight into avian chromosome evolution. The Journal of heredity. 2024;115 2:230-9. doi:10.1093/jhered/esad077.

93. Yang S, Lan T, Zhang Y, Wang Q, Li H, Dussex N, et al. Genomic investigation of the Chinese alligator reveals wild-extinct genetic diversity and genomic consequences of their continuous decline. Molecular Ecology Resources. 2023;23 1:294-311. doi:10.1111/1755-0998.13702.

94. Robinson JA, Kyriazis CC, Nigenda-Morales SF, Beichman AC, Rojas-Bracho L, Robertson KM, et al. The critically endangered vaquita is not doomed to extinction by inbreeding depression. Science. 2022;376 6593:635-9.

doi:doi:10.1126/science.abm1742.

95. Westbury MV, Petersen B, Garde E, Heide-Jørgensen MP and Lorenzen ED. Narwhal genome reveals long-term low genetic diversity despite current large abundance size. IScience. 2019;15:592-9.

96. Wang P, Hou R, Wu Y, Zhang Z, Que P and Chen P. Genomic status of yellow-breasted bunting following recent rapid population decline. iScience. 2022;25 7:104501. doi:10.1016/j.isci.2022.104501.

97. Gao C, Xing W, Liu H, Wang C, Han D and Wang G. Holocene wetland evolution in Northeast China and its responses to global change. Quaternary Sciences. 2018;38 4:854-63.

98. Ye Y and Yao C. Geographical discovery and early economic development in the Far East Pacific region of Russia. Journal of Heihe University. 2023;14 12:19-23.

99. Han M. Agricultural exploitation and population migration in northeast China in history. Chinese Landscape Architecture. 2021;37 10:6-10. doi:10.19775/j.cla.2021.10.0006.

100. Cui H, Wu T, Liu J, Liu W, Li Z, Cheng X, et al. Effects of succession processes of marsh wetland and farmland on groundwater in the Sanjiang Plain. Hydrogeology & Engineering Geology. 2023;50 06:51-8. doi:10.16030/j.cnki.issn.1000-3665.202306022.

101. Dahmer TD. Sanjiang Plain and Wetlands Along the Ussuri and Amur Rivers: Amur River Basin (Russia and China). In: Finlayson CM, Milton GR, Prentice RC and Davidson NC, editors. The Wetland Book: II: Distribution, Description and Conservation. Dordrecht: Springer Netherlands; 2016. p. 1-13.

102. Wei F, Han M, Han G, Wang M, Tian L, Zhu J, et al. Reclamation-oriented spatiotemporal evolution of coastal wetland along Bohai Rim, China. Acta Oceanologica Sinica. 2022;41 9:192-204. doi:10.1007/s13131-022-1987-3.

103. Zhong X and Kang H. Recent geo-environmental changes in the Bohai Bay coast. Quaternary Sciences. 2002;22 02:131-5+97-98.

104. Ga R. *The distribution and population size and trend of Oriential Stork (Ciconia boyciana) and the habitat selection of the overwintering group in Bohai Bay*. Master, Inner Mongolia Normal University, 2021.

105. Bauer S and Hoye BJ. Migratory animals couple biodiversity and ecosystem functioning worldwide. Science. 2014;344 6179:1242552. doi:10.1126/science.1242552.

106. Kokko H. Competition for early arrival in migratory birds. Journal of Animal Ecology. 1999;68 5:940-50.

107. Flack A, Aikens EO, Kölzsch A, Nourani E, Snell KRS, Fiedler W, et al. New frontiers in bird migration research. Current biology. 2022;32 20:R1187-R99. doi:10.1016/j.cub.2022.08.028.

108. Shao Y, Wang X-B, Zhang J-J, Li M-L, Wu S-S, Ma X-Y, et al. Genome and single-cell RNA-sequencing of the earthworm Eisenia andrei identifies cellular mechanisms underlying regeneration. Nature Communications. 2020;11

1:2656. doi:10.1038/s41467-020-16454-8.
109. Cole TL, Zhou C, Fang M, Pan H, Ksepka DT, Fiddaman SR, et al. Genomic insights into the secondary aquatic transition of penguins. Nature Communications. 2022;13 1:3912. doi:10.1038/s41467-022-31508-9.

Figure1

Click here to access/download;Figure;Figure1.pdf

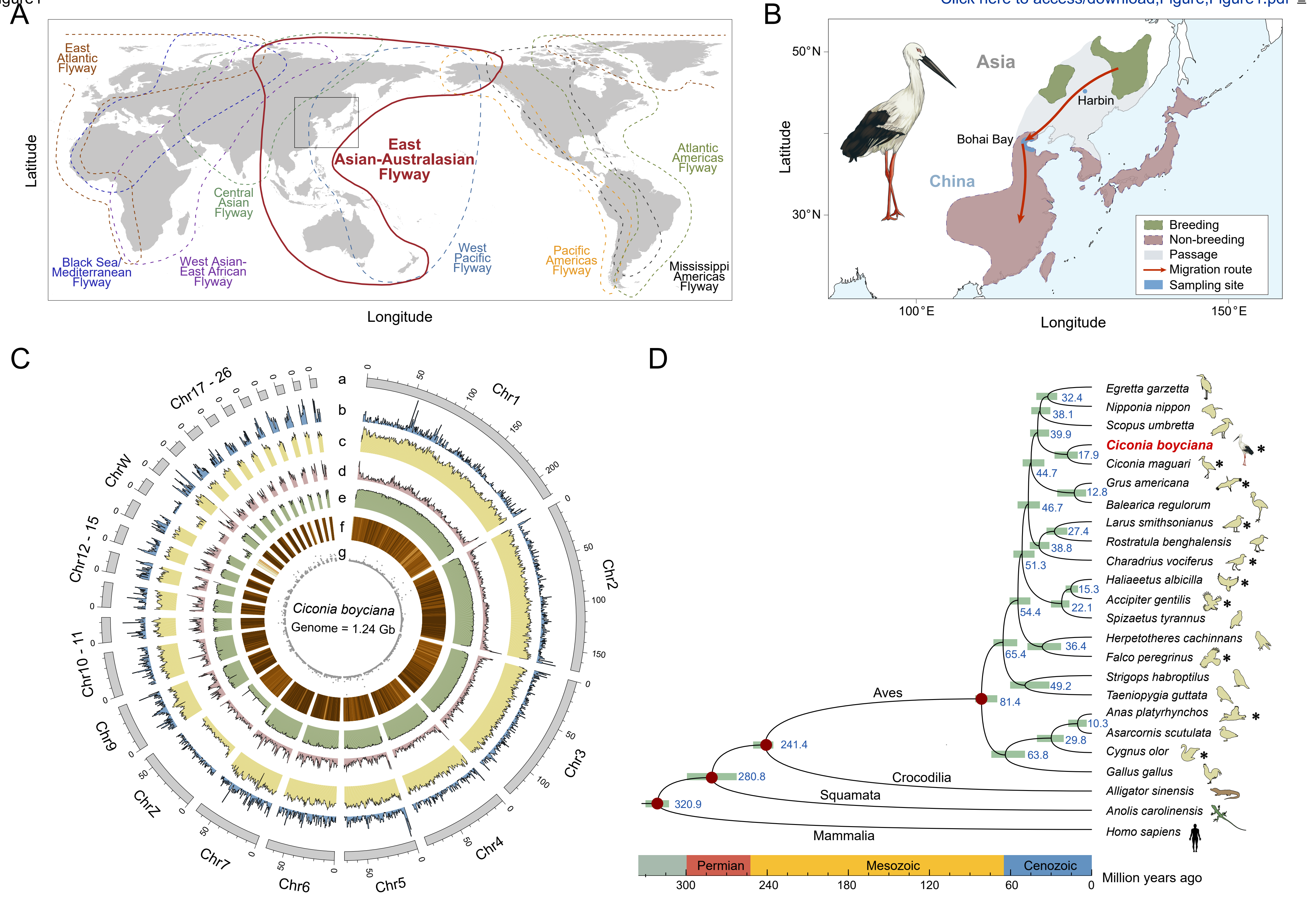

Figure2

Click here to access/download;Figure;Figure2.pdf

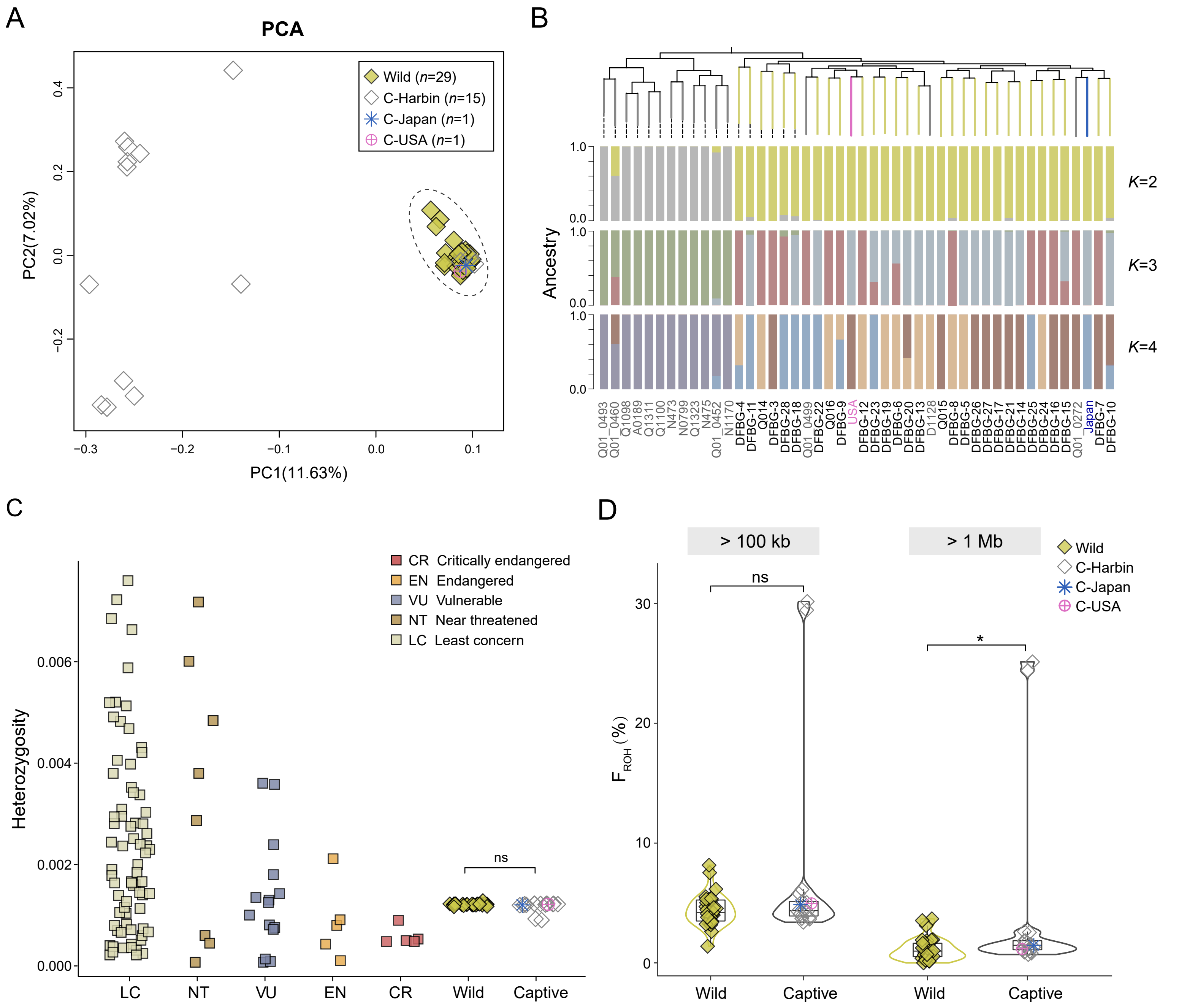

Figure3

Click here to access/download;Figure;Figure3.pdf

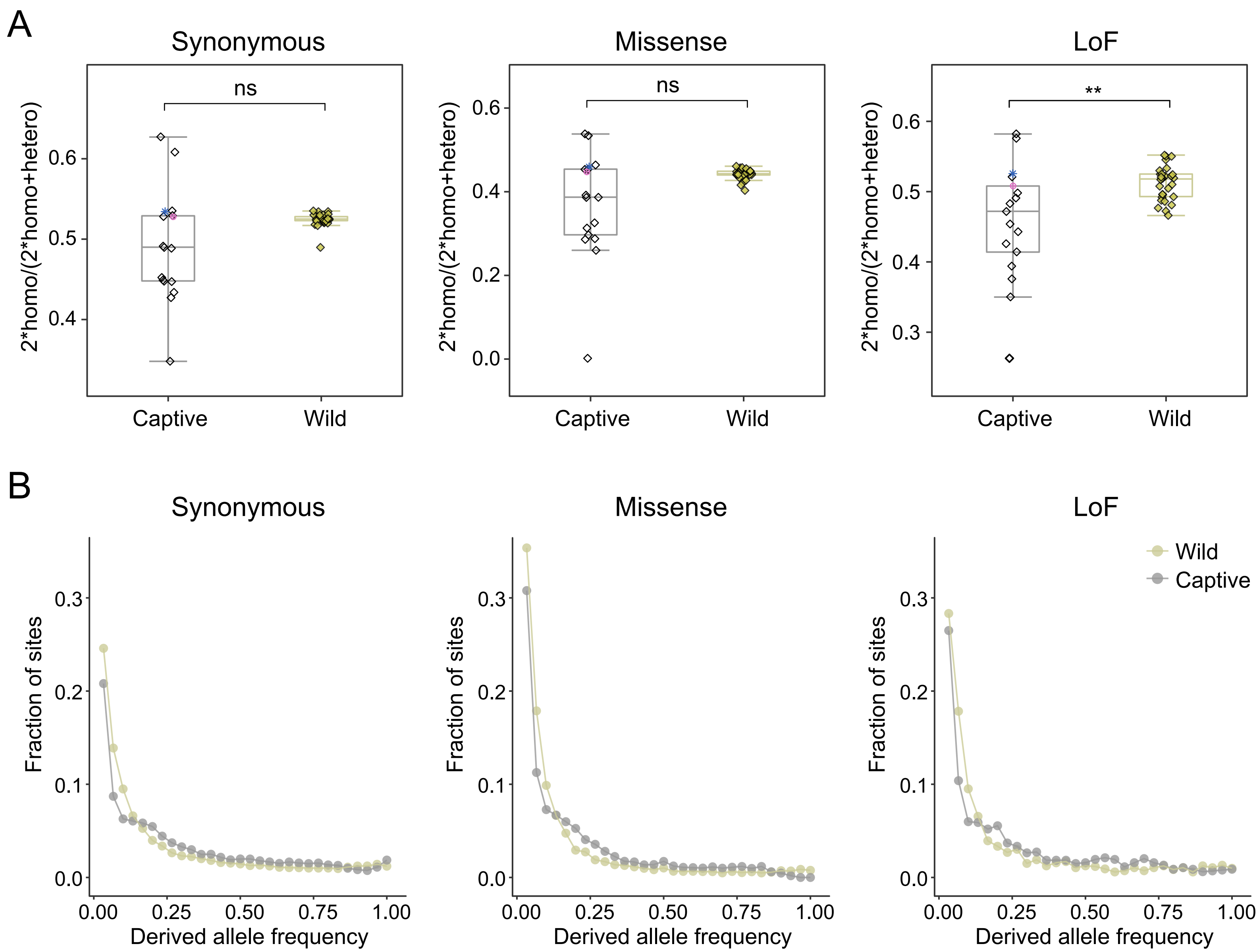

Figure4

Click here to access/download;Figure;Figure4.pdf

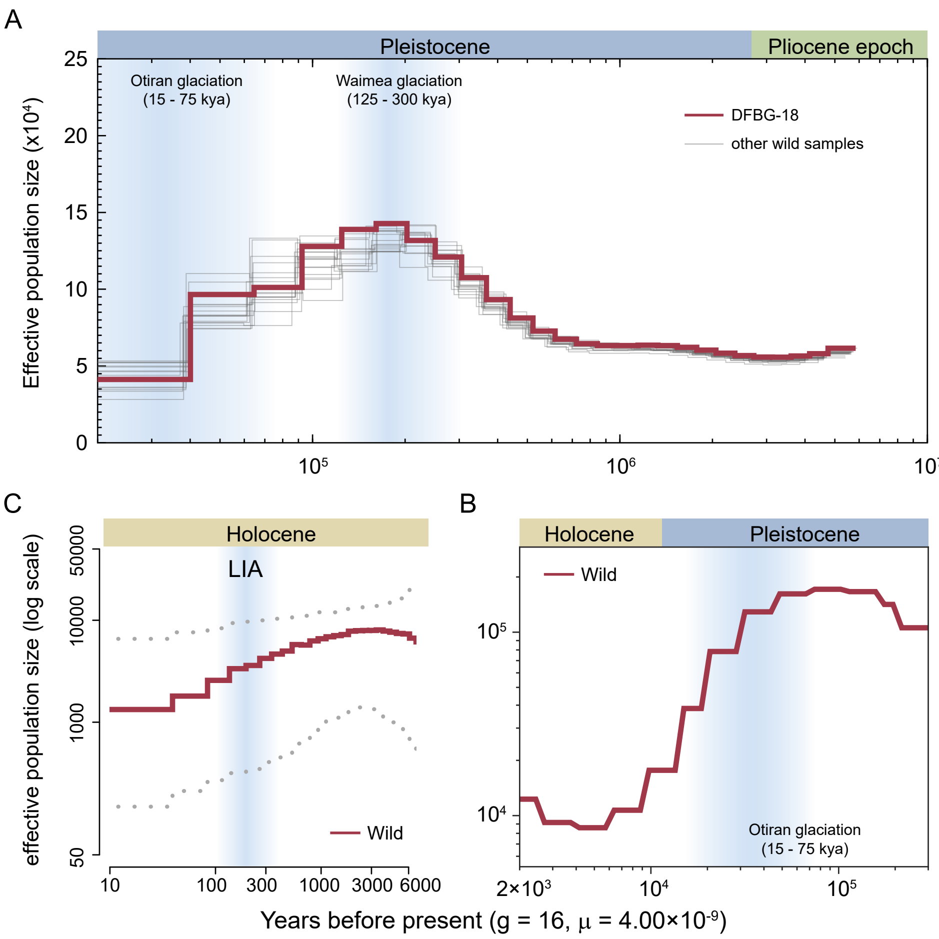

Figure5 Click here to access/download;Figure;Figure5.pdf

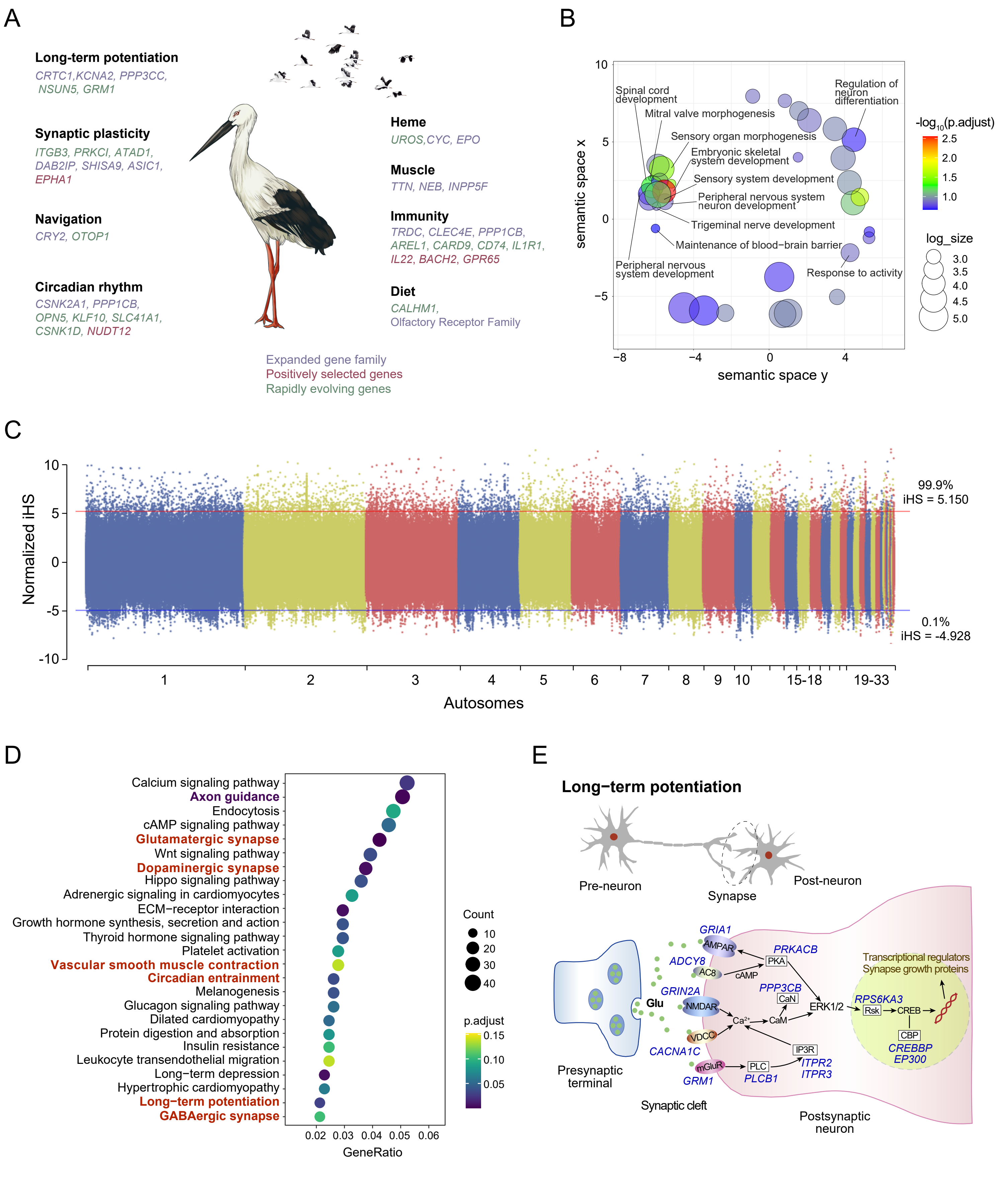

Click here to access/download
Supplementary Material
Supplementary-tables.xlsx

Click here to access/download
Supplementary Material
Supplementary-Figures.docx

Click here to access/download
Supplementary Material
Manuscript-track change.docx

Click here to access/download
Supplementary Material
Response to reviewer comments.docx

Cover letter
Click here to access/download;Personal Cover;Cover letter-1106.docx

November 06, 2023

Dr. Scott Edmunds
Editor-in-Chief
GigaScience

Dear Editor:

We hereby submit a manuscript entitled "**Genomic exploration of the endangered oriental stork, *Ciconia boyciana*, shed lights on migration adaptation and future conservation**" to be considered for publication in ***GigaScience***.

The decline of East Asian-Australasian Flyway (EAAF) seriously impacts the function and service of ecosystem due to human-bird conflict and climate change. Genomic resources and comprehensive assessment of endangered birds in EAAF are almost vacant, bringing difficulties in understanding their true threatened status and taking further conservation actions. Here, we focus on the well-known endangered migratory oriental stork, *Ciconia boyciana*, filling species and population-level genomic data gaps to benefit future bird protection. We present a high-quality chromosome-level genome assembly of the oriental stork and successfully identify Z and W chromosomes. We find a series of genomic signals related to the migratory trait, which imply an integrate work of brain synapses, photoreceptor cell, circadian rhythm, muscle tissue and energy metabolism. We also collect samples from wild and captive individuals and find that in fact oriental storks have a relative-high genetic diversity and low inbreeding level while wild population have a higher mutational load than captive-born animals. Their short-term decline hasn't resulted in serious genomic consequences till now and propose natural recovery hope.

I am authorized on behalf of all the authors of this article to confirm that no author has any conflict of interest to disclose. All authors have approved the version submitted for publication. The work in this article is original and has not been published previously, and the article is not under consideration by any other journal. We have discussed this manuscript with **Scott Edmunds and Hongling Zhou**, this July in BGI center. We thank you for your kind consideration of our article. Looking forward to hearing from you.

Sincerely,

Zhijun Hou, PhD.

Professor

College of Wildlife and Protected Area

Northeast Forestry University

Email: houzhijundb@163.com
