# Supplementary material for: Genomic exploration of the endangered oriental stork, Ciconia boyciana, sheds light on migration adaptation and future conservation
Source: Gigascience. 2024 Oct 22;13:giae081. doi: 10.1093/gigascience/giae081 (PMC11494145; doi:10.1093/gigascience/giae081)
Supplement: giae081_GIGA-D-23-00340_Revision_2 [file giae081_giga-d-23-00340_revision_2.pdf]

GigaScience

Genomic exploration of the endangered oriental stork, Ciconia boyciana, shed lights on migration adaptation and future conservation

--Manuscript Draft--

**Manuscript Number:**	GIGA-D-23-00340R2
**Full Title:**	Genomic exploration of the endangered oriental stork, Ciconia boyciana, shed lights on migration adaptation and future conservation
**Article Type:**	Research
**Funding Information:**	Surveillance of Wildlife Diseases from the State Forestry Administration of China (2023057) — Professor Zhijun Hou
	Leading Talent Project of “Science and Technology Leading Talent Team Project of Inner Mongolia Autonomous Region (2022LJRC0010) — Professor Zhijun Hou
	Start-up Scientific Foundation of Northeast Forestry University (60201524043) — Dr Tianming Lan
**Abstract:**	Abstract<br>Background: The oriental stork, Ciconia boyciana, is an endangered migratory bird listed on the IUCN Red List. They experienced a rapidly population decline in the past decades, with nest locations and stop-over sites largely degraded due to human-bird conflicts. The genome-wide genetic background of this threatened bird species is critical to make future conservation strategies but lack of investigation.<br>Findings: In this study, the first chromosome-scale reference genome was presented for the oriental stork with high quality, contiguity, and accuracy. The assembled genome size was 1.24 Gb with a scaffold N50 of 103 Mb, and 1.23 Gb contigs (99.32%) were anchored to 35 chromosomes. Population genomic analysis did not show a genetic structure in the wild population. Genome-wide genetic diversity (π = 0.0012) of the oriental stork was at a moderate to high level among threatened bird species and the inbreeding risk was also not significant (FROH = 5.56±5.30%). Reconstruction of demographic history indicated a rapidly recent population decline likely driven by human activities. Genes that were under positive selection for the migratory trait were identified in related to the long-term potentiation, photoreceptor cell, circadian rhythm, muscle development and energy metabolism, indicating the essential interplay between genetic and ecological adaptation.<br>Conclusions: The first chromosome-scale genome of the oriental stork expands genomic resource of endangered birds, providing a genomic basis for understanding its genetic background, extinction risk and the migratory characteristic, which will further facilitate the decision of future conservation plans for this species.<br>Keywords: oriental stork, comparative genomics, conservation genomics, endangered species, migration
**Corresponding Author:**	Shangchen Yang<br>Zhejiang University<br>Hangzhou City, CHINA
**Corresponding Author Secondary Information:**	
**Corresponding Author's Institution:**	Zhejiang University
**Corresponding Author's Secondary Institution:**	
**First Author:**	Shangchen Yang
**First Author Secondary Information:**	
**Order of Authors:**	Shangchen Yang
	Yan Liu

Powered by Editorial Manager® and ProduXion Manager® from Aries Systems Corporation

	Xiaoqing Zhao
	Jin Chen
	Haimeng Li
	Hongrui Liang
	Jiale Fan
	Mengchao Zhou
	Shiqing Wang
	Xiaotian Zhang
	Minhui Shi
	Lei Han
	Mingyuan Yu
	Yaxian Lu
	Boyang Liu
	Yu Xu
	Tianming Lan
	Zhijun Hou
**Order of Authors Secondary Information:**	
**Response to Reviewers:**	Reviewer reports:<br>Reviewer #1: The revised manuscript has done a lot of effort to make it better and clearer now. I agree with the second reviewer "The article has a large number of data lists, which is a common problem in genomics articles these days." However, we can see the author's efforts in this article, they compared the wild and the captive populations of Ciconia boyciana, also compared this bird with other birds in danger.<br><br>Thank you very much for your comments, and our manuscript has been much improved after this revision according to your suggestions, as well as comments from the reviewer 2. We hope this manuscript could provide useful genomic resources and insights for the future conservation of this endangered species. In this round of revision, we also comprehensively revised this manuscript as your comments and make a point-to-point response for your continence to trace the revision. We are looking forward to seeing your next round of comments, thank you very much.<br><br>Here are some additional comments,<br><br>Q1 avoid to use discuss results in terms of subjective statements through the MS, e.g. Abstract: We didn't find a genetic structure in the wild population. Result: We identified 10.41% of genome sequences as repetitive elements<br><br>Response: Thank you for pointing out this question, we have rephrased all sentences involved in this issue throughout the manuscript. All the places we revised has been highlighted in the track-change version.<br><br>Q2 Please list and discuss about some examples about protection and rescue of endanger species with the help of genomic analysis, which can make the meaning of this job more significant.<br><br>Response: Thank you, we agree. Genomic assessment is of great importance in the protection of endangered species. In the revised manuscript, we added a small paragraph in the introduction part to introduce the representative examples of how the genomics have been applied in the conservation efforts of endangered animals. Please see Line 74-83.<br><br>Q3 Line 282 The nucleotide diversity (π) of the wild and the captive populations, still,

	we didn't know the detail number for Ciconia boyciana.<br><br>Response: Thank you, we revised. This time we added the detailed number of the nucleotide diversity of Ciconia boyciana populations to make this comparison more reasonable and clearer. Please see Line 285.<br><br>Q4 Line428-430, The Discussion section, might be relevant to state the reason for the extinction of oriental storks in Japan and Korea?<br><br>Response: Yes, thank you for the advice. We searched some literatures and related the extinction of this bird species in Japan and Korea last century to the climate change and human activity, especially to the fast development of this two areas on the economy and industrialization. Please see Line 445-451.
**Additional Information:**	
**Question**	**Response**
Are you submitting this manuscript to a special series or article collection?	No
**Experimental design and statistics**<br><br>Full details of the experimental design and statistical methods used should be given in the Methods section, as detailed in our Minimum Standards Reporting Checklist. Information essential to interpreting the data presented should be made available in the figure legends.<br><br>Have you included all the information requested in your manuscript?	Yes
**Resources**<br><br>A description of all resources used, including antibodies, cell lines, animals and software tools, with enough information to allow them to be uniquely identified, should be included in the Methods section. Authors are strongly encouraged to cite Research Resource Identifiers (RRIDs) for antibodies, model organisms and tools, where possible.<br><br>Have you included the information requested as detailed in our Minimum Standards Reporting Checklist?	Yes
**Availability of data and materials**	Yes

All datasets and code on which the conclusions of the paper rely must be either included in your submission or deposited in publicly available repositories (where available and ethically appropriate), referencing such data using a unique identifier in the references and in the "Availability of Data and Materials" section of your manuscript.<br><br>Have you have met the above requirement as detailed in our Minimum Standards Reporting Checklist?	

Powered by Editorial Manager® and ProduXion Manager® from Aries Systems Corporation

Click here to access/download;Manuscript;Endangered oriental stork- Sep 26.docx

Genomic exploration of the endangered oriental stork, *Ciconia boyciana*, sheds light on migration adaptation and future conservation

Shangchen Yang[1,2†], Yan Liu[3†], Xiaoqing Zhao[4,5†], Jin Chen[1], Haimeng Li[1,6], Hongrui Liang[3], Jiale Fan[1], Mengchao Zhou[1], Shiqing Wang[1], Xiaotian Zhang[3], Minhui Shi[1], Lei Han[1], Mingyuan Yu[3], Yaxian Lu[1], Boyang Liu[1], Yu Xu[3‡], Tianming Lan[1,6*], Zhijun Hou[1*]

[1]College of Wildlife and Protected Area, Northeast Forestry University, Harbin, 150040, China

[2]College of Life Sciences, Zhejiang University, Hangzhou 310058, China

[3]Center for Biological Disaster Prevention and Control, National Forestry and Grassland Administration, Shenyang 110034, China

[4]Inner Mongolia Academy of Agricultural & Animal Husbandry Sciences, Hohhot 010031, Inner Mongolia, China;

[5]Key Laboratory of Black Soil Protection and Utilization (Hohhot), Ministry of Agriculture and Rural Affairs, P.R. China, Hohhot 010031, Inner Mongolia, China;

[6]Heilongjiang Key Laboratory of Complex Traits and Protein Machines in Organisms, Harbin 150040, China

[*]**Correspondence address.** Tianming Lan, College of Wildlife and Protected Area, Northeast Forestry University, Harbin, China. E-mail: lantianming1314@126.com; Zhijun Hou, College of Wildlife and Protected Area, Northeast Forestry University, Harbin, China. E-mail: houzhijundb@163.com

[†]These authors contributed equally to this work.

[‡] Senior author.

Shangchen Yang [0000-0001-8802-3028]; Yan Liu; Xiaoqing Zhao [0000-0002-6631-2870]; Jin Chen [0009-0005-2453-6912]; Haimeng Li [0000-0003-4355-5906]; Hongrui Liang [0000-0001-6064-1377]; Mengchao Zhou [0000-0001-5641-0955]; Shiqing Wang; Xiaotian Zhang; Minhui Shi [0009-0002-9582-6018]; Lei Han [0000-0001-6487-

7520]; Mingyuan Yu [0009-0008-2943-7392]; Yaxian Lu [0000-0003-0660-5409]; Boyang Liu; Yu Xu [0000-0003-2135-9875]; Tianming Lan [0000-0002-6934-0439]; Zhijun Hou [0000-0002-8704-1651];

Abstract

Background: The oriental stork, *Ciconia boyciana*, is an endangered migratory bird listed on the IUCN Red List. The bird population has experienced a rapid decline in the past decades, with nest locations and stop-over sites largely degraded due to human-bird conflicts. Multipronged conservation efforts are required to secure the future of oriental storks. We propose that a thorough understanding of the genome-wide genetic background of this threatened bird species is critical to make future conservation strategies.

Findings: In this study, the first chromosome-scale reference genome was presented for the oriental stork with high quality, contiguity, and accuracy. The assembled genome size was 1.24 Gb with a scaffold N50 of 103 Mb, and 1.23 Gb contigs (99.32%) were anchored to 35 chromosomes. Population genomic analysis did not show a genetic structure in the wild population. Genome-wide genetic diversity (π = 0.0012) of the oriental stork was at a moderate to high level among threatened bird species and the inbreeding risk was also not significant (F_{ROH} = 5.56±5.30%). Reconstruction of demographic history indicated a rapid recent population decline likely driven by human activities. Genes that were under positive selection associated with the migratory trait were identified in related to the long-term potentiation, photoreceptor cell organization, circadian rhythm, muscle development and energy metabolism, indicating the essential interplay between genetic and ecological adaptation.

Conclusions: Our study presents the first chromosome-scale genome assembly of the oriental stork, provides a genomic basis for understanding genetic background of the oriental stork, the population’s extinction risks and the migratory characteristics, which will facilitate the decision of future conservation plans for this species.

Keywords: oriental stork, comparative genomics, conservation genomics, endangered species, migration

Introduction

Ecosystem degradation and biodiversity decline occur throughout the Anthropocene and accelerated in the recent decades [1]. Human activities have led to habitat loss, speeding up the pace of the sixth mass extinction. More than 32% of extant species (~44,000) are threatened with extinction [2]. This global crisis in turn poses a threat to human well-being, and calls for more conservation efforts to stop and reverse the current situation.

Birds are effective indicators of biodiversity condition on earth [3]. Long-term records from BirdLife International have raised significant concern for the world's birds: populations of 49% bird species (5,412) are declining, including both endangered and unendangered birds, and for many species, the risk of extinction is escalating. Nearly 45% of Important Bird and Biodiversity Areas (IBAs) are identified to be in danger due to pervasive and unsustainable human activities, such as agricultural expansion, logging, and hunting [4-6]. In particular, wetlands along the East Asian-Australasian Flyway (EAAF) (Fig. 1A) are being heavily destroyed, leading to the loss of key wintering sites and bird mortality [7, 8]. The EAAF flyway is used by 492 migratory bird species, more than 50 million individual birds use the flyway during their annual flight from Arctic Russia and Alaska, to Australia and New Zealand [9, 10]. Intensification of human-bird conflicts in Asia have led to a massive decline in the numbers of many migratory waterbirds including the endangered oriental stork, *Ciconia boyciana* (NCBI:txid52775) [11], black-faced spoonbill, *Platalea minor* [12], and the vulnerable white-naped crane, *Antigone vipio* [13]. Migratory birds are wildlife without habitat boundaries and serve as a powerful safeguard for ecosystems. The decline of bird species in the EAAF reduce the energy connectivity and mobility between Arctic nutrient-poor terrestrial system and the southern coastline ecosystem. Although governments have taken actions to protect coastal wetlands and migratory birds, there are still gaps [14]. For example, for most of these threatened migratory birds, the population-level and genome-wide genetic

data is lacking and thus difficult to assess their genome-wide genetic background, bringing obstacles to design scientific recovery actions.

The oriental stork is a large wetland shorebird in the EAAF. It has been listed as “Endangered” on the IUCN Red List since 1994. The wild bird population comprises a single population with an estimated population size of c. 3000 individuals (Eastern Asia population) [15]. They are full migrants, breeding in southeastern Siberia, mainly along the Russia-China border, and migrating annually to Bohai Bay (1,500 km) and Poyang Lake (2,600 km) in autumn (Fig. 1B) [16]. Oriental storks were once widely distributed across northeast Asia, however, the wild population dramatically declined in 1868-1935 [17]. In 1970s, wild storks in Japan and Korea disappeared, with the remaining individuals breeding in more constricted areas in the Russian Far East and China. In 1960s, there were more than 1000 oriental storks breeding in the Heilongjiang Province. However, their numbers decreased to 123 in 1986 and was less than 50 in 1990 [18, 19]. Habitat loss is considered as the major reason for their population decline. Deforestation, agricultural development, and spring fires severely destroy their nest trees in Russia. Reclamation of wetlands, and overfishing in the stop-over and wintering sites in China lead to a decreased refueling rate and an increased mortality rate, particularly for juveniles [15, 20].

In this decade, genomic technology has become a promising tool in the field of conservation, and conservation genetics is in transition to the conservation genomics with the rapid development of the sequencing technology and plummeting sequencing costs [21-24]. Investigating genetic backgrounds of endangered species by genomic approaches can inform conservation efforts [25]. The Iberian lynx (*Lynx pardinus*) is one example of endangered species that benefitted from conservation genomics. Developing a high-quality reference genome and population genomic studies have generated a high-quality variation map and a catalog of deleterious mutations for the Iberian lynx, which are now used for evaluating population fitness and monitoring the genetic diversity of the reintroduced population [26-29]. Other examples include conservation genomics studies of the Florida panther [30], the Indian

tiger [31], and the Kākāpō [32]. However, population genetic studies of the oriental stork were largely underexplored for genome-wide investigation, including their genetic diversity, inbreeding level, as well as the genomic basis for the adaptation of migration traits.

In this study, the first chromosome-level genome assembly was presented for the oriental stork. The genomes of 29 wild and 15 captive birds were re-sequenced for extensively exploring the genetic characteristics of this endangered bird. The genetic background of these birds was then systematically investigated to measure possible genome-wide extinction risks. Genomic signatures of evolution and adaptation for the migratory-related characteristics were scanned across the genome and related to population viability.

Methods

Samples and ethics statement

Blood samples from 16 captive-born and 3 wild-rescued oriental storks were collected at Harbin North Forest Zoo, Harbin, Heilongjiang, China. The blood sample from a captive-born oriental stork (sample ID: N1170) was used for reference genome assembly. Additional 26 wild-rescued oriental storks were collected around the Bohai Bay, China, and the muscle samples from these individuals were collected after their natural death. Research and blood/tissue collection were approved by the Institutional Review Board of Northeast Forest University (No.2024WPE05). We also downloaded whole-genome sequencing data generated from two bird individuals: one from Kanagawa, Japan (n=1) and another from San Diego Zoo, the United States of America (USA) (n=1). Our final data set consisted of 46 samples, including 26 wild birds from Bohai Bay, 18 from Harbin North Forest Zoo (3 wild and 15 captive birds), 1 captive bird from Japan and 1 captive bird from USA.

Nucleic acid extraction, library construction and sequencing

For ONT long-read sequencing, high molecular weight genomic DNA was extracted using the DNeasy Blood and

Tissue kit (Qiagen, USA), and 8-10 μg DNA was size-selected (> 50 kb) according to the manufacturer's instructions for the ONT library preparation. 800 ng of library DNA were used for sequencing on the PromethION sequencer (Oxford Nanopore Technologies, UK). For Hi-C sequencing, cross-link process with formaldehyde was firstly conducted using the blood sample and then Hi-C library was constructed following the protocol of Lieberman-Aiden *et al* [33]. Total RNA was extracted using TRlzol reagent (Invitrogen). We then used the Agilent 2100 Bioanalyser system (Agilent, USA) and Qubit 3.0 (Life Technologies, USA) to evaluate the quality and quantity of the extracted RNA. DNA libraries with short insert sizes were prepared according to the manufacturer's instructions on the MGI platform (MGI, Shenzhen, China). These libraries were finally sequenced on the DNBSEQ-T1 sequencer for 100-bp paired-end reads.

De novo **assembly, annotation, and assessment**

The genome size of the oriental stork was first estimated by *k*-mer frequency method based on whole genome sequencing (WGS) data of the assembled individual [34]. The genome was then assembled as per the following steps: 1) *de novo* assembly was conducted using ONT long reads by NextDenovo (v2.5.0) (RRID:SCR_025033). Two core modules were utilized to generate a primary assembly: the NextCorrect module was used to correct raw ONT long-reads and extract the consensus sequences. NextGraph module was used for the preliminary assembly. A read cutoff of 1 Kb was set and other default parameters were maintained in NextDenovo. 2) Contigs were polished using NextPolish (v1.4.0) (RRID:SCR_025232) [35] with ONT long reads. 3) Hi-C reads were mapped to the genome using Burrows-Wheeler aligner *mem* (BWA, v0.7.17) [36] algorithm with default parameters. 3d-DNA pipeline (v180,922) (RRID:SCR_017227) was applied to generate a chromosome-level genome assembly. 4) WGS reads were remapped to the assembly [37] to correct mis-sequenced bases introduced by long-read sequencing. 5) Benchmarking Universal Single-Copy Orthologs (BUSCO) (RRID:SCR_015008) analysis [38] was performed to evaluate the completeness of our assembly using aves_odb10 database. 6) WGS, Hi-C and RNA-seq data were

mapped to the final genome to check mapping rate, bases coverage and sequencing depth by BWA *mem* algorithm with default parameters.

De novo and homology-based methods were combined to identify repetitive elements in the genome assembly. Firstly, *de novo* predictions were performed using LTR finder (v1.0.6) (RRID:SCR_015247) [39], MITE-hunter (v4.07) [40] and RepeatModeler2 (v2.0.1) [41] softwares with default parameters. The results were merged into RepBase as known repeats. Next, RepeatMasker (v4.0.5) (RRID:SCR_012954) [42] was used to identify and classify transposable elements by searching the RepBase library [43]. Tandem repeats were identified using Tandem Repeats Finder (TRF, v4.09) [44].

All repetitive elements were masked across the genome for annotation of the protein-coding genes. A combination of *de novo*, homology-based and transcript mapping methods were used to conduct gene annotation. *De novo* predictions were carried out using SNAP (v1.0) (RRID:SCR_002127) [45], glimmerHMM (v3.0.3) (RRID:SCR_002654) [46] and AUGUSTUS (v2.5.5) (RRID:SCR_008417) [47] softwares. We used Trimmomatic (v0.27) (RRID:SCR_011848) [48] to filter the RNA data and assembled the data using Trinity (v2.9.0) (RRID:SCR_013048) [49]. The data set was then mapped to the reference genome to predict gene structure using the Program to Assemble Spliced Alignments (PASA, v2.2.0) (RRID:SCR_014656) [50]. For homology-based prediction, protein sequences from *Gallus gallus*, *Anas platyrhynchos*, *Ciconia maguari*, *Meleagris gallopavo*, *Pavo muticus*, *Taeniopygia guttata* and *Homo sapiens* were aligned to our genome using Blastall (v2.2.26) [51] with an E-value cut-off of 1e-5. The gene models were confirmed using GeneWise (v2.4.1) [52]. The results obtained from the above three approaches were finally combined to generate a comprehensive gene set using Maker (v 3.01.03) [53]. These genes were aligned to the databases of SwissProt, TrEMBL, InterPro, Gene ontology (GO) and Kyoto Encyclopedia of Genes and Genomes (KEGG) for functional annotation.

Identification of sex-linked regions

The two sex chromosomes (Z and W chromosomes) were identified by checking the sequencing depth of the individual male and female birds. The syntenic relationships with the sex chromosomes of *G. gallus* (GenBank ID: GCA_016699485.1) and *T. guttata* (GenBank ID: GCF_003957565.2) were then examined. Alternative splicing of each gene on the chromosomes were filtered for the three species. The longest protein sequences of *G. gallus* and *T. guttata* were aligned against our assembled Z and W chromosomes using blastp in BLASTtools (v2.2.26) [54] with the parameter of "-evalue 1e-5". Synteny blocks were then identified using MCScanX [55] and visualized by Circos(v0.69-9) [56] software.

Phylogeny reconstruction and divergence time estimation

Protein sequences from 24 species (*H. sapiens*, *Anolis carolinensis*, *Alligator sinensis*, *G. gallus*, *Cygnus olor*, *Asarcornis scutulata*, *A. platyrhynchos*, *T. guttata*, *Strigops habroptilus*, *Falco peregrinus*, *Herpetotheres cachinnans*, *Spizaetus tyrannus*, *Accipiter gentilis*, *Haliaeetus albicilla*, *Charadrius vociferus*, *Rostratula benghalensis*, *Larus smithsonianus*, *Balearica regulorum*, *Grus americana*, *C. maguari*, *C. boyciana*, *Scopus umbrette*, *Nipponia nippon*, *Egretta garzetta*) were aligned to identify homologous gene families. The longest protein sequence translated from each gene was selected in this alignment by blastp function in the BLASTtools (v2.2.26) [54] with the parameter of "-evalue 1e-5". A total of 1,800 shared single-copy genes were used to construct a maximum-likelihood phylogenetic tree by IQTREE (v1.6.12) [57]. Divergence time among these species was estimated by MCMCTREE (v4.5) in PAML [58] software with multiple fossil time points used for time calibration [59].

Variants calling and quality control

Whole-genome sequencing data from individual bird samples used in this study were mapped to our assembled

reference genome using the BWA *mem* algorithm with default parameters. Reads sorting, reordering and deduplication were carried out using Picard (v2.1.1) (RRID:SCR_006525) . Variant calling was performed using DNAseq Haplotyper in Sentieon (v202010.01) [60]. Bam files and genomic Variant Call Format (gVCF) files were generated for each individual and joint calling was conducted using GVCFtyper algorithm in Sentieon DNAseq pipeline to generate a combined VCF file covering all individuals. Variants were filtered using the following procedures: 1) InDels and multi-allelic variants were removed; 2) hard filtering with the parameters: QD < 2.0 || FS > 60.0 || MQ < 40.0 || MQRankSum < -12.5 || ReadPosRankSum < -8.0 –filter-name snp_filter; 3) genotype missing rate larger than 10% were removed from the variant set. Single-nucleotide polymorphism (SNP) sites on the Z and W chromosomes were also removed for the downstream population genomic analysis.

Population structure analysis

The VCF files were converted into PLINK format files with VCFtools (v0.1.16) (RRID:SCR_001235) [61]. Principal component analysis (PCA) was then performed with PLINK (v1.9) [62] software. Inference of ancestral components was conducted with ADMIXTURE (v1.3.0) [63] and *K* value was set from one to five with "-cv" flag to calculate the cross-validation (CV) error. A phylogenetic tree was constructed using IQTREE (v1.6.12) with 1000 bootstraps. The tree layout was visualized using the online tool iTOL (RRID:SCR_018174).

Genetic diversity and inbreeding

Genome-wide heterozygosity (*H*) of each individual genome was calculated by the VCFtools (v0.1.16). Nucleotide diversity (π) was calculated by a non-overlapping 5 Mb sliding window along all autosomes using VCFtools (v0.1.16). Runs of homozygosity (ROHs) were identified using PLINK (v1.9) with the following parameters: --homozyg-window-snp 20 --homozyg-kb 100 --homozyg-density 50. Inbreeding coefficient was estimated as the proportion of genome present in ROH region (F_{ROH}). Comparison between wild and captive populations was

conducted using two-sided pairwise *t*-test in R (v 4.1.2).

Mutational load

The alleles in the *C. maguari* genome, the closest relative of oriental stork, were used to serve as the ancestral state of the oriental stork genome. The reference genome of *C. maguari* (GenBank ID: GCA_013399255.1) was transformed to a 100 bp FASTQ file by sliding a nonoverlapping window across the genome and the short reads were then mapped to our assembled genome using BWA *mem* with the parameter: -B 3. Only reads uniquely mapped to our genome were kept by SAMtools/BCFtools (RRID:SCR_005227) (v1.3) [64] view function with "-F 4 -q 20". Finally, a consensus sequence was generated to represent ancestral alleles on the oriental stork genome using SAMtools mpileup function with depth > 1×. A new VCF file containing 6,028,662 derived SNPs was obtained after replacing the reference alleles by a custom Perl script.

SnpEff (v4.3) software was used to annotate the derived SNPs into three categories: (1) synonymous mutations; (2) missense mutations; (3) loss of function (LoF) mutations. Here, the "stop_gained", "splice_donor_variant" and "splice_acceptor_variant", "start_lost", "stop_lost" and "splice_region_variant" were considered as LoF mutations. Next, the number of SNPs per individual in homozygous and heterozygous state were counted, respectively. The proportion of homozygous derived alleles was measured following the formula: 2 × homozygous sites / (2 × homozygous sites + heterozygous sites) [65]. Derived allele frequency was calculated with 15 randomly selected individuals from the wild and captive bird population to avoid the potential bias from sample size.

Inference of population demography

Combination of Pairwise Sequentially Markovian Coalescent (PSMC, v0.6.5) [66], SMC++ (v1.13.1) [67] and approximate Bayesian computation (ABC) methods were used to track the population dynamics of the wild population over generations. For the PSMC analysis, the bam file of each individual was converted to a fasta format

sequence using SAMtools mpileup function with the depth setting to ≥ 1/3 and ≤ 2 of the average sequencing depth. PSMC software was then run with the parameters: -N25 -t5 -r5 -p 4+25*2+4+6. For the SMC++ method, two individuals from the wild population were randomly selected to generate a mask file of uncovered regions by bamCaller.py. SMC++ was applied based on covered sites to infer population history with the following parameters: --cores 8 --knots 24 --timepoints 20 100000. For the PopSizeABC analysis, SNP sites with a MAF > 0.2 were used as the input file for PopSizeABC (v2.1) [68] software with the parameters: mac (minor allele count threshold for AFS and IBS statistics computation) = 0; mac_ld (minor allele count threshold for LD statistics computation) equals 3,4,5 respectively; L (size of each segment, in bp) = 4,000,000; nb_rep (number of simulated data sets) = 500; nb_seg (number of independent segments in each data set) = 30. The results generated by the three methods were visualized with a generation time of 16 years and the mutation rate of 4.0×10^{-9} substitutions per site per generation [15].

Comparative genomic analysis related to migration

To understand the possible genomic basis of the migration characteristics of the oriental stork, comparative genomic analyses were performed with non-migratory bird species and other migratory birds. Unique adaptative signals detected in the oriental stork when compared with the non-migratory birds, which were absent in the dataset comparing the oriental stork with other migratory birds, were regarded as the potential genetic basis contributing to their migratory phenotype. Here, we focused on expanded gene families, positively selected genes (PSGs) and rapidly evolving genes (REGs). The Treefam (v1.4) [69] and CAFÉ (v4.2.1) [70] were used to identify expanded gene families. PSGs and REGs were identified under a branch model and a branch-site model based on the single-copy genes in the CodeML of PAML (v4.8) [58] with the threshold of the adjusted *P*-value for false discovery rate set as 0.05. GO and KEGG enrichment analyses were performed using the “cluserProfiler” package in R (v4.0.2) [71, 72]. Networks of GO terms were visualized by REVIGO to summarize redundant terms [73].

Detecting genomic signatures of recent adaptation

SNPs in the wild population were phased by BEAGLE (v5.0) [74] with the default parameters. Recent positive selection signals were detected using the integrated haplotype score (iHS, version 1.3) [75] method and iHS scores were normalized by subtracting the genome-wide mean iHS score and dividing by the standard deviation (calculated by the software WHAMM). SNPs with the highest or lowest 0.1% standardized iHS scores were considered as candidate ancestral or derived alleles under strong positive selection. Four methods were used to select genes that were under recently positive selection: 1) Genes in a 5 Kb flanking region around the candidate SNPs; 2) Sliding 100 Kb windows across the whole genome, genes were selected as candidates if they intersected with the 100 Kb windows containing candidate SNPs; 3) Sliding 50 SNP windows across the whole genome, genes were selected as candidates if they intersected with the 50 SNP windows that contained candidate SNPs; 4) Genes harboring candidate SNPs.

Results

Chromosome-level genome assembly and annotation

A reference genome for a female oriental stork was assembled by combining ONT long reads (~89.81-fold), DNB short reads (~69.77-fold), and Hi-C reads (~98.20-fold) (Supplementary Table S1). The genome size of the oriental stork was estimated to be 1.29 Gb (Supplementary Fig. S1), and the final chromosome-scale genome assembly had a size of 1.24 Gb (Fig. 1C, Table 1 and Supplementary Fig. S2). The scaffold N50 of this genome was 102.77 Mb and the contig N50 was 35.79 Mb (Supplementary Table S2). More than 99.32% of all contigs were successfully anchored onto 35 chromosomes. The GC content of the oriental stork genome was 42.40%, very close to that of its related species, *C. maguari* (GenBank ID: GCA_013399255.1, 40.90%) and *S. umbrette* (GenBank ID: GCA_013400535.1, 41.50%). The chromosome-level genome assembly generated in this study also showed high

completeness with a BUSCO score of 97.6% (Supplementary Table S3). Lastly, 99.73%, 99.89% and 94.80% of the WGS, Hi-C and RNA-seq reads, respectively, could be successfully mapped onto the final assembly (Supplementary Table S4). The depth ratio of male/female on the Chr8 and Chr16 were observed to be about 2 and 0, respectively, consistent with the mapping pattern of Z and W chromosomes (Supplementary Fig. S3 A-C). Syntenic analysis with *G. gallus* and *T. guttata* also supported that Chr8 and Chr16 were Z and W chromosomes, respectively (Supplementary Fig. S3 D and E).

Table 1: Statistics of the genome assembly for the oriental stork.

**Genomic features**	**Parameters**
Assembled genome size (bp)	1,240,615,254
Contig N50 (bp)	35,788,150
Scaffold N50 (bp)	102,765,642
Longest contig (bp)	131,589,000
Longest scaffold (bp)	220,403,942
GC content (%)	42.40
Percent of repetitive sequences (%)	10.41
Number of gene models	15,609

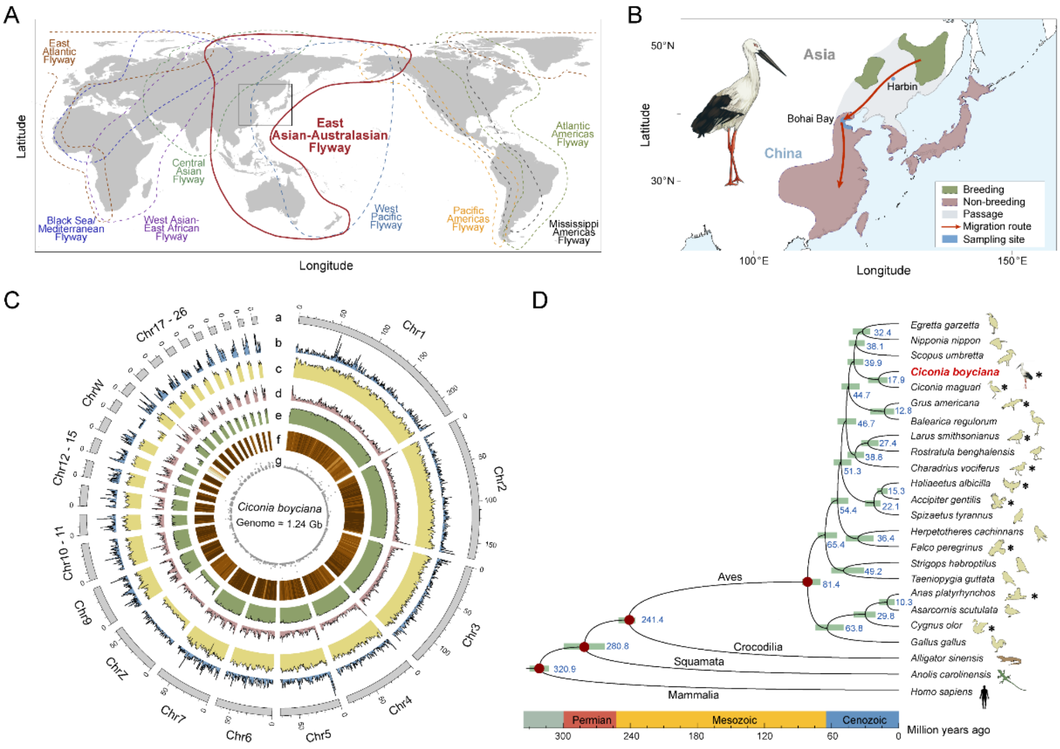

Figure 1: Landscape of the genome assembly and phylogenetic placement of the oriental stork. (A) Nine major flyways across the globe [6]. Black box indicates the range of the oriental storks. (B) Breeding and wintering regions, migratory route and sampling site (Bohai Bay) of the wild oriental storks used in this study. (C) Genomic features of the oriental stork. (a) The 26 chromosomes larger than 5 Mb. (b) Gene count. (c) Depth of Hi-C reads. (d) GC content density. (e) Depth of WGS reads. (f) Repeat number. (g) Depth of RNA reads. The statistics were calculated using a 500-kbp window. (D) Phylogenetic relationship of the 24 species and the estimated divergence time. Asterisks represented full migrants while others are not migrants.

Approximately 10.41% of genome sequences were identified as repetitive elements (129.09 Mb), including LTRs (3.46%), LINEs (5.64%), DNA elements (0.73%), SINEs (0.13%) and unknown repeats (0.10%) (Supplementary Table S5-S7). After masking these repeat elements, a total of 15,609 protein-coding genes were predicted in our assembly, and the average gene length, intron length and exon length were 24.53 kb, 2.58 kb and 173.96 bp (9.84 exons per gene), respectively, which were comparable to other avian species (Supplementary Table S8, Supplementary Fig. S4). All predicted genes (100%) were functionally annotated in at least one of the five databases

used in this study (Supplementary Fig. S5, Supplementary Table S9). Additionally, 208 miRNAs, 152 rRNAs, 440 tRNAs and 274 snRNAs were predicted in this study (Supplementary Table S10). A phylogenetic tree was constructed based on 1,800 shared single-copy gene families. Aves and Crocodilian were sister clades that diverged at c. 241.4 Mya, and the oriental stork split with *C. maguari* at c. 17.9 Mya (Fig. 1D, Supplementary Fig. S6).

Population structure, genetic diversity and inbreeding

In order to assess the genomic background of the oriental stork population, paired-end sequencing data of 46 individual birds (29 wild and 17 captive) were mapped to the assembled reference genome. Average sequencing coverage and depth for the 46 birds were 97.80% and 22.81-fold, respectively (Supplementary Table S11). After filtering low-quality variants and variants in the sex chromosomes, 6,525,198 qualified SNPs were obtained across 33 autosomes.

PCA, admixture and phylogenetic tree all supported that the wild birds belonged to a single genetic cluster while the captive individuals presented a scattered distribution (Fig. 2A and B). Five captive birds including the three Harbin birds, one bird from Japan and another from USA were clustered into the wild population, implying that the five birds have very similar genetic background with the wild population. The lowest CV error for K=2 suggested that there should be two dominant ancestral components (Fig. 2B, Supplementary Fig. S7), and admixture analysis for larger K values revealed that wild birds might have more complex ancestral components.

Furthermore, the genetic diversity and inbreeding levels were calculated for the oriental stork population to assess the threatened status of this species. Average genome-wide heterozygosity (H) of all 46 individual birds was estimated to be $1.20\times10^{-3} \pm 6.32\times10^{-5}$, which was at a relatively high level among the endangered avian species (crested ibis: 4.30×10^{-4} [65]; saker falcon: 8.00×10^{-4} [76]; Chatham Island black robin: 4.80×10^{-4} [77]; and kākāpō: 5.00×10^{-4} [32]) (Fig. 2C, Supplementary Table S12 and S13). The H of the wild birds was slightly higher than that

of the captive birds (H_{wild}=1.21×10^{-3} ± 2.04×10^{-5}; $H_{captive}$=1.18×10^{-3} ± 9.72×10^{-5}), but with no significant difference. The nucleotide diversity (π) of the wild and the captive populations ($\pi_{captive}$=1.12×10^{-3}; π_{wild}=1.02×10^{-3}) was also higher than that of the brown eared pheasant (*Crossoptilon mantchuricum*, 9.60×10^{-5}) [78] and the green peafowl (*P. muticus*, 4.70×10^{-4}) [79] populations (Supplementary Fig. S8).

Although the oriental stork genomes presented a relatively high genome-wide genetic diversity, the average $F_{ROH \geqslant 100\ kb}$ across the genome was around 5.56±5.30% (wild: 4.44±1.35%; captive: 7.47±8.19%) (Fig. 2D). ROH fragments larger than 1 Mb were rare in these individuals with an average value of 2.29±4.86% (wild: 1.18±0.96%; captive: 4.18±7.54%). Significant differences were found between the wild and the captive populations for $F_{ROH \geqslant 1Mb}$ (p = 0.04). Unexpectedly, two captive birds seemed to be highly inbred (F_{ROH}: ~30%), which was consistent with their lower H than others (Fig. 2C). Overall, the low-level F_{ROH} of oriental storks suggested a surprisingly low inbreeding risk.

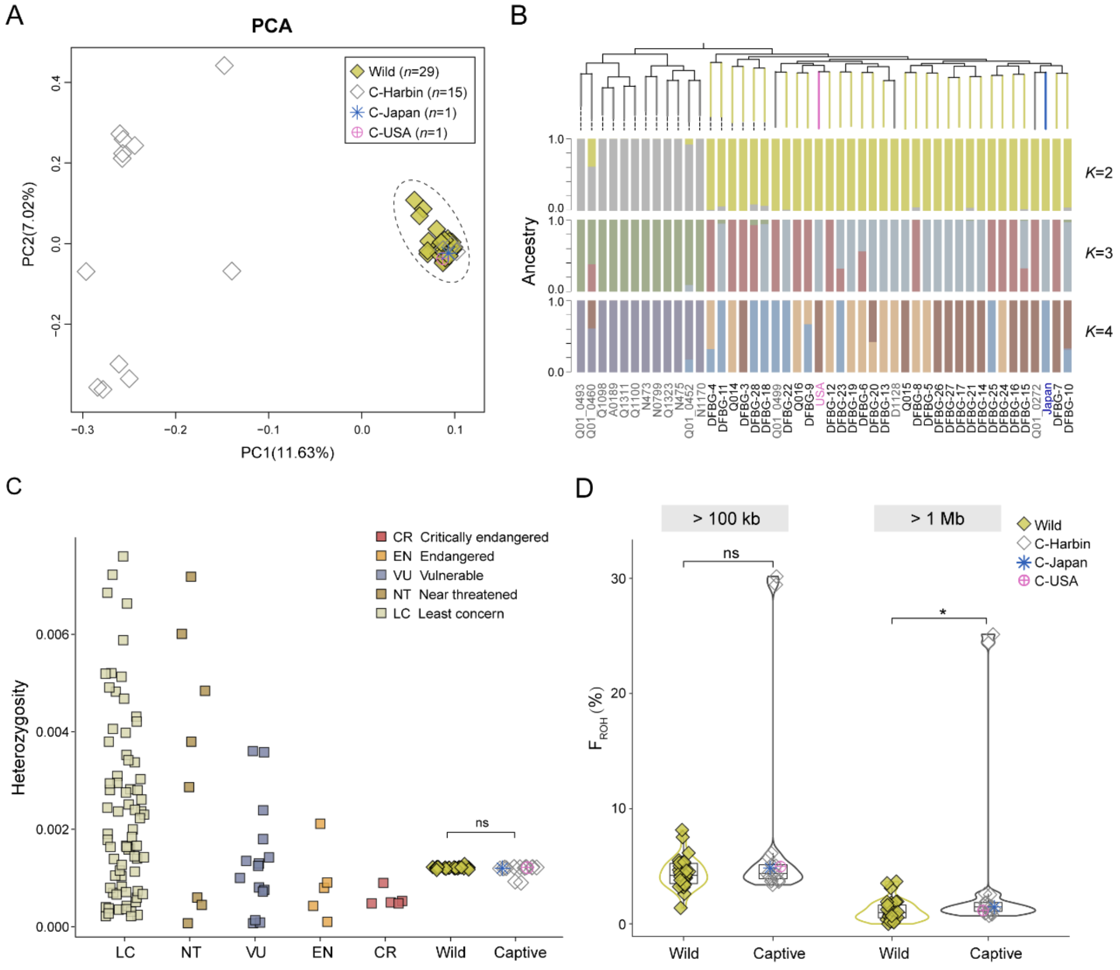

Figure 2: Population genetic structure, genome-wide heterozygosity, and inbreeding level of the oriental storks. (A) PCA result for the 46 birds presented with the first and second principal components. (B) Phylogenetic relationship and admixture analysis of the 46 individuals with a *K* value from 2 to 4. (C) Comparison of whole-genome heterozygosity in bird species with different threatened status defined by IUCN. (D) Individual inbreeding coefficients inferred by F_{ROH}. The F_{ROH} for ROH ⩾ 100 Kb and ROH ⩾ 1 Mb are shown here (two-sided *t*-test, ns = non-significant, *$p < 0.05$).

Higher mutational load in the wild population

Mutational load is a genetic factor associated with the fitness cost of a species. In this study, mutational load was screened across the genome in each bird and a large number of missense and LoF mutations were identified (Fig. 3A, Supplementary Fig. S9). The average values of the derived mutational load present in both heterozygous and

homozygous states in the wild population were higher than that found in the captive individuals (Supplementary Table S14). In particular, homozygous LoFs were significantly increased in the wild population (172.00±8.14) compared to the captive population (139.76±33.64). The frequency of LoF mutations scaled by synonymous mutations was also higher in the wild than the captive population (Supplementary Fig. S10). 71.2% and 62.3% of all genes influenced by missense and LoF mutations were shared by the wild and captive populations. The wild stork population also had more private genes carrying missense mutations (Supplementary Fig. S11A). The unique genes interrupted by LoF mutations in the wild birds were not enriched in any GO terms that were related with life activities or fitness (Supplementary Fig. S11B).

We then preformed folded site frequency spectrum (SFS) analysis for the derived alleles to determine genetic drift (Fig. 3B). Both the wild and the captive populations displayed "L-shaped" lines in the three categories of mutations, and presented a large fraction of rare derived alleles indicating a mutation drift equilibrium [31]. However, low-frequency alleles were relatively deficient while medium-frequency alleles were excessive in the captive population when compared to the wild population, indicating that the genetic drift seemed to be slightly stronger in the captive oriental storks [78].

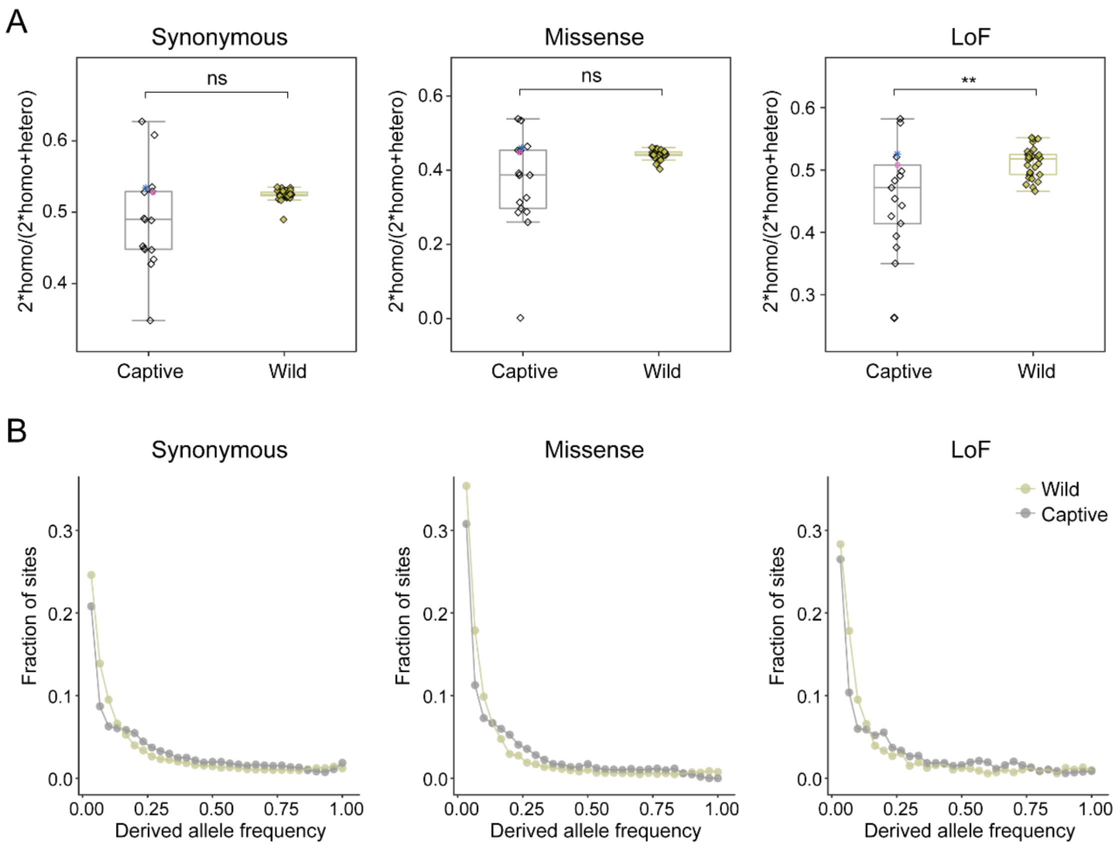

Figure 3: Mutational load of the wild and the captive populations. (A) Statistics of derived alleles, including synonymous, missense and LoF mutations. The ratio of homozygous derived alleles in each individual genome is shown here (two-sided *t*-test, ns = non-significant, $^{**}p < 0.01$). (B) Folded SFS of synonymous, missense and LoF mutations in the wild and the captive populations. The proportion of loci (y axis) is shown for each derived allele frequency (x axis).

Historical population dynamics

The historical population dynamics is closely related to the accumulation of genetic load [80]. In order to evaluate the change in effective population sizes (N_e) of oriental storks over its evolutionary history, the demographic trajectory for the last 6 million years (My)was reconstructed in this study. The entire population history of the oriental stork was characterized by two population expansions and two population declines. Wild oriental storks experienced the first population expansion at around 800-200 thousand years before present (ka BP) after a long period of retaining a steady population size, and then a serious decline occurred ca. 200-6 ka BP (Fig. 3A and B). A slight

recovery occurred at 6-3 ka BP. The most recent decrease started at 3 ka BP and the final N_e dropped to approximately 1,000 (Fig. 3C). The N_e/N_c (census size, N_c) ratio was about 0.33 for the contemporary wild population, which fell within the range of most species (0.5-0.10) [81].

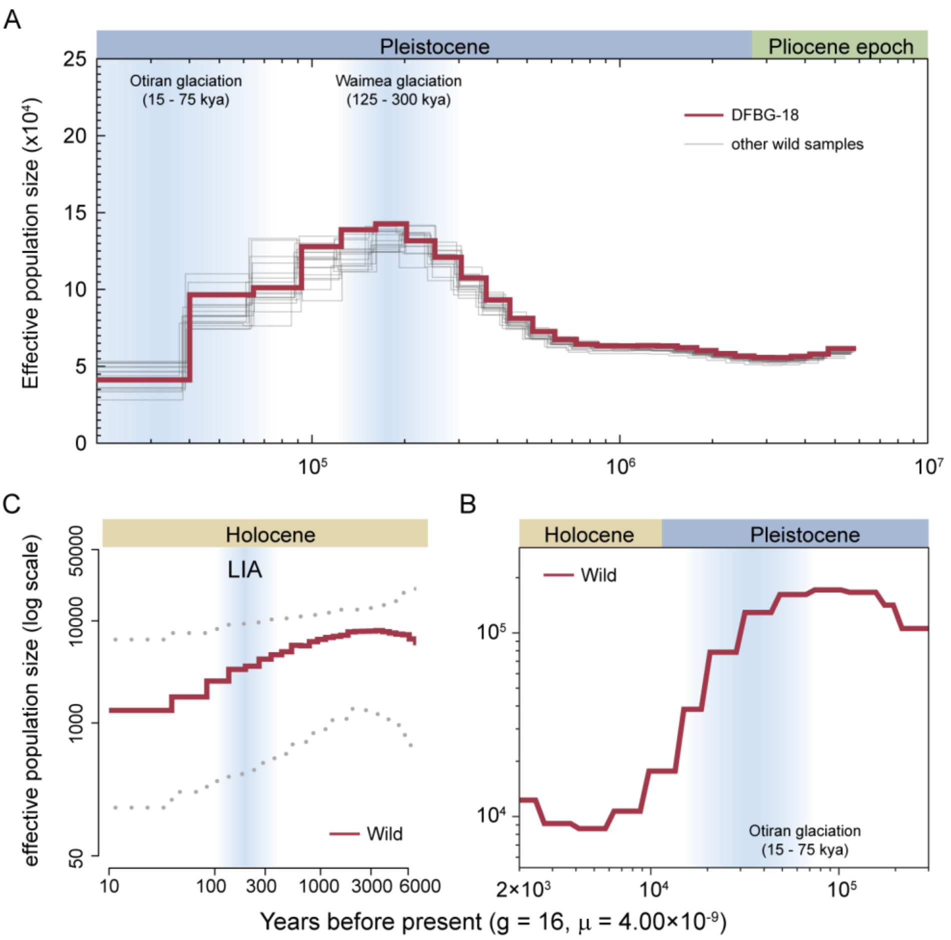

Figure 4: Estimated demographic history of the wild population of oriental stork. (A) Large-scale demographic fluctuation from 6 million years ago (Mya) to 20 ka BP was inferred by PSMC. (B) Recent population history over the past 200-0.3 ka estimated by SMC++ with 29 wild individuals. (C) Recent effective population size for the wild population inferred by PopSizeABC. Dotted lines indicate a 90% confidence interval. Light-blue shadows depict several glacial periods including Waimea glaciation, Otiran glaciation and the little ice age (LIA).

Genomic insights for bird migration

Long-distance migratory birds are under significant selective pressure from their early life [82]. The interplay

between genetics, learning and spatial memory plays a critical role in shaping the complex migration behavior [83, 84]. The migratory ability of the oriental stork is closely related with its survival, and a better understanding of the genomic basis for this biological adaptation is expected to facilitate the future conservation of this bird species.

Here, the oriental stork was compared with other avian species with or without the migratory trait (Supplementary Fig. S12). A total of 526 expanded gene families, 107 PSGs, and 308 REGs were identified in the oriental stork genome compared with non-migratory birds (uM group). Meanwhile, a total of 557 expanded families, 90 PSGs and 279 REGs were identified in the oriental stork genome when other full-migratory birds (M group) were used for comparisons (Supplementary Fig. S13). Here, only the genes found in the analysis with the uM group but not found in the analysis with the M group were considered as candidate genes that were more likely the genomic basis for the migratory traits associated with the oriental stork. Our findings suggest a portion of these genes with functions related to the migration (Fig. 5A, Supplementary Table S15-S17). GO enrichment analysis of the expanded gene families showed that a series of GO terms were enriched in the sensory system development and peripheral nervous system development (Fig. 5B), which may be important to increase the sensitivity to environmental changes and transmit these signals to central nervous system. Notably, the trigeminal nerve development (GO:0021559) was distinctly enriched in the GO analysis, which was previously shown to be vital for the development of map sense in night-migratory songbirds [85]. Of particular interest, radical pairs of cryptochromes are magnetically sensitive and CRY4 is responsible for the light-dependent magnetic compass in the night-migratory European robin [86]. Here, the CRY2 gene family was found to be expanded in oriental stork but was not found in non-migratory birds (Supplementary Fig. S14). Long-term potentiation in the hippocampus is closely related to memory and learning, which contributes to the migratory route formation in peregrine falcons [84]. As for the oriental stork, two PSGs (*SPG11* and *EPHA1*) and nine REGs (*ITGB3*, *NSUN5*, *KCTD16*, *PRKCI*, *ATAD1*, *EPHA1*, *GRM1*, *ADGRL3*, and *NEXMIF*) were found involved in synaptic plasticity. The *NSUN5* gene product is essential for NMDAr-dependent long-term potentiation

and *Nsun5*-KO mice showed spatial cognitive deficits [87]. *ATAD1* gene encodes ATPase family AAA domain-containing protein 1, which controls AMPA receptor (AMPAR) internalization that regulates synaptic activity. Absence of *ATAD1* would affect the amplitude of miniature excitatory postsynaptic currents and finally cause deficits in learning and memory [88].

For long-distance migrants, their breast or flight muscles comprise red muscles with a high concentration of myoglobin. The muscles are highly vascularized and the cells contain a high concentration of mitochondria. These adaptations enable the birds to carry on oxidative metabolism for prolonged periods of time when the birds are in flight [89, 90]. We identified genes related to heme biosynthesis (REG: *UROS*; expanded gene families: *CYC* and *EPO* genes) and genes that code for proteins involved in muscle development (expanded gene families: *TTN*, *ENB*, and *INPP5F* genes). In terms of immunity, several genes involved in pathogen clearance were identified, which are of vital importance to the innate immune response.

Additionally, 10,312 SNPs were identified to be under recent positive selection detected by iHS method based on population genomic sequencing data, and 25.92% of these SNPs were distributed within genic regions (1,453 genes) (Fig. 5C, Supplementary Fig. S15). GO and KEGG enrichment analyses revealed a series of biological functions and pathways associated with the neurons, including learning and memory (GO:0007611), synaptic plasticity (GO:0048167), axon development (GO:0061564), glutamatergic synapse (hsa04724), dopaminergic synapse (hsa04728), GABAergic synapse (hsa04727) (Fig. 5D, Supplementary Table S18 and S19). Our finding confirmed the presence of a positively selected SNP in the *ADCY8* gene. The *ADCY8* gene was previously shown to be important in the long-distance migratory peregrine populations [84]. Besides the *ADCY8* gene, there were another 12 positively selected genes functioning in the long-term potentiation pathway: four genes (*GRIA1*, *GRIN2A*, *CACNA1C* and *GRM1*) encoding membrane receptors, five genes (*PRKACB*, *PPP3CB*, *PLCB1*, *ITPR2* and *ITPR3*) responsible for intracellular signal transduction, and three genes (*RPS6KA3*, *CREBBP* and *EP300*) affecting nucleus

transcription activity. The discovery of these genes and their associated pathways provide clues to uncover the genetic factors shaping the migratory route of oriental storks (Fig. 5E).

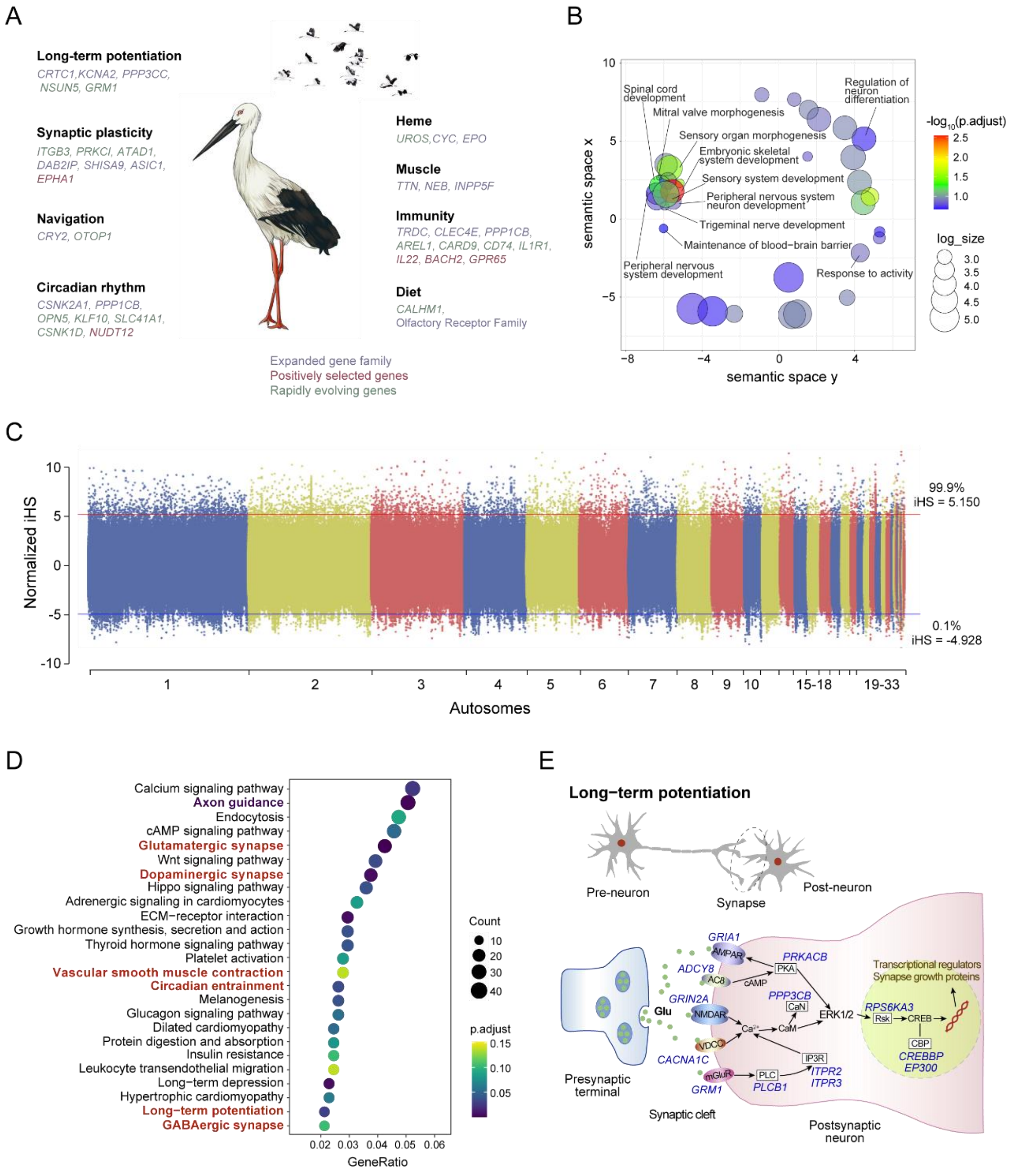

Figure 5: Genomic signatures relevant to migration in the oriental stork. (A) Genes or pathways that were detected may contribute to the migratory traits. (B) GO items representing biological processes by REVIGO for expanded gene families in the oriental stork genome. Semantic similar GO terms clustered together. (C) Normalized iHS scores indicating candidate SNPs under recent positive selection in the wild population. Red and blue lines represented the 99.9th and 0.1th quartile of iHS scores, respectively. (D) KEGG

pathway analysis for recently positive selection. (E) Genes with recent positive selective signals that related to the long-term potentiation pathway.

Discussion

Species are disappearing at an accelerated rate due to pervasive anthropogenic impacts [91, 92]. Record of the past 600 years reflected a peak in bird extinction rates in the nineteenth century, which occurred first in the Pacific islands [93]. This extinction contrasts the fast substantial diversification of the relatively young avian clade [94]. Conservation efforts have been enhanced with the rapid development of genome sequencing technologies [23, 95]. However, genomic resources and genomic investigations are still lacking for many threatened species [96]. Here we assembled a high-quality reference genome and sequenced the genomes of both wild and captive cohorts of the endangered oriental stork. The findings of our study would make up for the knowledge deficit surrounding the genetic background of both the wild and captive populations of oriental storks. The knowledge from the study will further benefit future conservation plans.

The assembled genome comprised of 33 autosomes, and the Z and W sex chromosomes. Our assembly was consistent with the previous karyotypical study for the oriental stork ($2n$ = 68) [97, 98]. Sex chromosomes and microchromosomes are relatively difficult to be assembled and deserve special attention, and the synteny analysis could help confirm the accuracy of the assembly and unanchored scaffolds like those found in the wood stork (*Mycteria americana*) study [99]. The Z chromosome could not be not completely assembled in this study, because the assembled length was shorter than the reported karyotype. Microchromosomes were clearly divided into 23 contacted scaffold groups, showing the effectiveness of Hi-C data. Our assembled genome can be regarded as a new representative reference genome and provides valuable genomic resource for further ecological and evolutionary studies, and conservation efforts of the oriental stork, as well as other stork species.

The wild population of the oriental stork experienced serious decline in the last century and the population size started to increase in recent years under conservation efforts. Oriental storks are still classified as an "endangered" species, with no more than 2,500 mature individuals. More than 95% of the individuals live in a single subpopulation [15]. Consistently, population genomic analysis did not show a genetic structure in the wild population. And fortunately, the wild population was found to possess high genetic diversity and low inbreeding level ($F_{ROH} < 10\%$), which may result from their migration every year along the EAAF. This contrasts with some geographically isolated and highly inbred animal populations [32, 100]. Interestingly, two highly inbred captive birds ($F_{ROH} \approx 30\%$) with decreased heterozygosity and a significantly higher level of long ROH fragments (>1Mb) were identified, which more likely originated from the recent inbreeding. This phenomenon sheds light on the importance of the scientific pedigree management in the small breeding populations with limited founders.

Endangered species with different historical population dynamics usually face different extinction risks [101, 102]. It has been demonstrated that small populations experiencing long-term declines would have low genetic diversity and high-levels of inbreeding. In contrast, a recent rapidly decline has less effects on the genetic diversity [103]. For the oriental stork, the wild population increased in size during 6-3 ka BP and started to decline at 3 ka BP, approximately 187 generations(g) ago. The population history may explain the relatively high genetic diversity found in the wild population nowadays. The relatively large N_e (from 10,000 to 1,000) over the recent 3 ky might be responsible for the low levels of inbreeding and reducing genetic drift in the wild population.

The relatively high genetic diversity and a recent history of population decline in oriental storks has endowed population with a strong genetic capacity to recover. The rapid recent decline of the oriental stork population might have resulted from both climate change and human activities. Their breeding area, including the Amur River basin, spanned Russia, China and Mongolia. These areas were originally covered by temperate forests and populated by a relatively small number of nomadic people living on hunting and fishing. Around 3 kya, the climate in this region

turned cold and dry. The forest system was converted to wetlands and thus, the nest trees for the oriental storks became scarce [104]. As human society moved from Bronze Age to Iron Age around 2.6 kya, an increase in human activities might have led to habitat loss causing a decline in the bird population [104]. Over the past several centuries, the intensity of human activities has increased manifold. Colonization of Russia in the 17th century brought modern agriculture and economic development [105]. Agricultural activities in the northeast China started in two waves in the Liao-Jin period and in Qing Dynasty [106]. Human settlements in this region experienced the most rapid expansion in the past 100 years with large-scale agricultural reclamation process in the China side of the Amur River basin [107]. Wetlands in the Sanjiang plain have declined in area by 86% from 108,900 km^2 prior to the 20th century to some 14,800 km^2 in 2000 [108]. Moreover, the most important stop-over site of the oriental storks, the Bohai Bay, is an area with the most concentrated coastal reclamation activities in China [109]. Natural wetlands experienced striking environmental changes driven by rapid industrialization and urbanization in the last century [110]. In the Japanese and Korean peninsulas, the climate has been changing periodically. In the recent century, rapid industrialization and economic expansion in both Japan and Korea have destroyed many natural habitats. The rapidly industrialized agriculture and heavy use of chemical fertilizers and pesticides have reduced the natural food sources of the oriental stork, contributing to the extinction of this bird species in both Japan and Korea since 1971. In summary, habitat degradation due to recent human activities may explain the serious decline in the oriental stork population and other birds along the EAAF.

Migrants may adaptively adjust migratory routes and wintering areas in response to climate change and anthropogenic influence [111]. Improved evolutionary insights on the migration can facilitate the conservation for migratory birds. Billions of animals migrate annually across the planet, in pursuit of improved foraging opportunities, safety, and reproductive output [112]. Migrants face selective pressure to arrive early to occupy high-quality territories at the stop-over/wintering sites [113]. Efficient migration requires coordinated support of the brain

function and physical conditions [84, 86, 114]. Specific phenotypes and physiological functions are often hypothesized to be attributed to the selection of the underlying protein-coding genes [115, 116]. A series of expanded gene families, PSGs, and REGs related to learning and memory, navigation, muscle development and energy metabolism were identified in the oriental stork. Recent work has revealed that adult birds shape their migratory route through individual exploring and learning over a lifetime [83]. Long-term potentiation is critical for the large-scale spatial memory. For example, the long-distance migratory population of peregrine falcon experienced distinct selection on *ADCY8* gene, which regulates downstream memory-related genes [84]. Here, we also found that *ADCY8* gene, together with other genes along the long-term potentiation pathway, were under positive selection in the oriental storks, and several biological pathways responsible for neuronal synapse formation were overrepresented. These findings provide new insights into the genomic basis of the migratory performance of the oriental stork, as well as the possible flexibility of this species in responding to the environmental changes through learning and memory.

Data Availability

The final genome assembly data, RNA-seq data and raw resequencing genome data are available in the NCBI BioProject repository (accession number: PRJNA1036389). All additional supporting data are available in the *GigaScience* repository, GigaDB [117].

Additional Files

Supplementary Fig. S1. The distribution of 21-mer for the genome size estimation of the oriental stork.

Supplementary Fig. S2. Heatmap of Hi-C chromosomal interaction density among all 35 chromosomes.

Supplementary Fig. S3. Identification of sex chromosomes in the oriental stork genome.

Supplementary Fig. S4. Comparison of gene characteristics of avian species and human.

Supplementary Fig. S5. Venn diagram representing the functional annotation of the oriental stork gene set.

Supplementary Fig. S6. Comparison of the gene repertoires of 24 vertebrate genomes.

Supplementary Fig. S7. Cross validation (CV) error in the ADMIXTURE analysis.

Supplementary Fig. S8. Nucleotide diversity (π) across 25 autosomes in wild and captive populations, respectively, by sliding a 5-Mb window.

Supplementary Fig. S9. Statistics of the number of heterozygous and homozygous loci for synonymous, missense and LoF mutations in each individual.

Supplementary Fig. S10. Statistics of the frequency of heterozygous, homozygous loci and sites of missense and LoF mutations, scaled by synonymous mutations.

Supplementary Fig. S11. Genes harboring putative missense and LoF mutations.

Supplementary Fig. S12. The expanded and contracted gene families in each bird genome and the comparison of their gene repertoires.

Supplementary Fig. S13. Comparison of detected genomic signals in the oriental stork genome compared with non-migratory birds and migratory birds, including expanded families, positively selected genes and rapidly evolving genes.

Supplementary Fig. S14. The phylogenetic tree of *CRY2* gene constructed by the maximum likelihood method. The red clade represented three *CRY2* genes in oriental stork.

Supplementary Fig. S15. Venn diagram of the four methods used to identify genes affected by candidate SNPs

under recent selection.

Supplementary Table. S1. Statistics of sequencing data for genome assembly and gene annotation.

Supplementary Table. S2. Genomic statistics of the *C. boyciana* assembly.

Supplementary Table. S3. BUSCO analysis of the whole genome and the gene set of the *C. boyciana* genome.

Supplementary Table. S4. Statistics of sequencing data mapped to the *C. boyciana* genome which were used for the assembly and gene annotation.

Supplementary Table. S5. Transposable elements (TEs) statistics in our assembly.

Supplementary Table. S6. Statistics of identified Repeats by *De* novo method in *C. boyciana* genome.

Supplementary Table. S7. Transposable elements in the *C. boyciana* assembly.

Supplementary Table. S8. Statistics of annotations for the *C. boyciana* gene set.

Supplementary Table. S9. Statistics on functional annotation of the *C. boyciana* gene set.

Supplementary Table. S10. Statistics of ncRNA annotation.

Supplementary Table. S11. List of 46 oriental stork samples used for re-sequencing.

Supplementary Table. S12. Whole-genome heterozygosity and F_{ROH} of 46 re-sequenced samples.

Supplementary Table. S13. Whole-genome heterozygosity for the published birds presented in Fig. 2C.

Supplementary Table. S14. Average number of heterozygous, homozygous loci and all sites for derived alleles in wild and captive populations.

Supplementary Table. S15. Expanded gene families in *C. boyciana* genome compared with non-migratory birds.

Supplementary Table. S16. Rapidly evolving genes in *C. boyciana* genome compared with non-migratory birds.

Supplementary Table. S17. Positively selected genes in *C. boyciana* genome compared with non-migratory birds.

Supplementary Table. S18. GO enrichment of genes directly affected by positively selected SNPs.

Supplementary Table. S19. KEGG pathway enrichment of genes directly affected by positively selected SNPs.

Abbreviations

IBA: Important Bird and Biodiversity Area; EAAF: East Asian-Australasian Flyway; IUCN: International Union for Conservation of Nature; ONT: Oxford Nanopore Technology; Hi-C: high-throughput chromosome conformation capture; WGS: whole genome sequencing; Kb: kilobase pairs; BUSCO: Benchmarking Universal Single-Copy Orthologs; RNA-seq: RNA sequencing; BWA: Burrows-Wheeler aligner; gVCF: genomic Variant Call Format; SNP: single-nucleotide polymorphism; PCA: principal component analysis; cross-validation: CV; iHS: integrated haplotype score; H: heterozygosity; ROH: run of homozygosity; LoF: loss of function; PSMC: Pairwise Sequentially Markovian Coalescent; ABC: approximate Bayesian computation; MAF: minor allele frequency; GO: Gene ontology; KEGG: Kyoto Encyclopedia of Genes and Genomes; BLAST: Basic Local Alignment Search Tool; Gb: gigabase pairs; Mb: megabase pairs; Chr: chromosome; SFS: site frequency spectrum; N_e: the effective size; N_c: the census size; PSG: positively selected gene; REG: rapidly evolving gene; LTR: long terminal repeat; LINE: long interspersed nuclear element; SINE: short interspersed nuclear element; BP: before present; Mya: million year ago; LIA: little ice age; *CRY2*: Cryptochrome Circadian Regulator 2; NCBI: National Center for Biotechnology Information.

Competing Interests

The authors declare no competing interests.

Funding

This work was financially supported by funding from Surveillance of Wildlife Diseases from the State Forestry Administration of China (2023057), the Leading Talent Project of “Science and Technology Leading Talent Team Project of Inner Mongolia Autonomous Region (2022LJRC0010), and the Start-up Scientific Foundation of Northeast Forestry University (60201524043).

Acknowledgments

We thank all staffs for their work in the collection of animal samples.

Authors’ Contributions

T.L. and Z.H. conceived and designed the research. H.L., M.Z., S.W. and X.Z. organized and collected the samples. M.S., L.H., M.Y., Y.L. and B.L. prepared the sequencing library. J.C. and H.L. performed genome assembly and annotation. S.Y., Y.L. and X.Z. conducted comparative genomic analysis and population genetic analysis. S.Y. wrote the manuscript. Y.X. and T.L. extensively revised the manuscript. T.L. and Z.H. supervised the study. All authors have read and approved the final manuscript.

References

1. Johnson CN, Balmford A, Brook BW, Buettel JC, Galetti M, Guangchun L, et al. Biodiversity losses and conservation responses in the Anthropocene. Science 2017;**356**(6335):270-5. doi:10.1126/science.aam9317.
2. Paez S, Kraus RH, Shapiro B, Gilbert MTP, Jarvis ED, Group VGPC, et al. Reference genomes for conservation. Science 2022;**377**(6604):364-6. doi:10.1126/science.abm8127.
3. Gregory RD, Noble D, Field R, Marchant J, Raven M and Gibbons D. Using birds as indicators of biodiversity. Ornis hungarica 2003;**12**(13):11-24.
4. Kirby JS, Stattersfield AJ, Butchart SHM, Evans MI, Grimmett RFA, Jones VR, et al. Key conservation issues for migratory land- and waterbird species on the world's major flyways. Bird Conservation International 2008;**18**(S1):S49-S73.

doi:10.1017/S0959270908000439.

5. International B. State of the World's Birds 2018—taking the pulse of the planet. Cambridge, UK: BirdLife International 2018. https://www.birdlife.org/papers-reports/state-of-the-worlds-birds/. Accessed 1 Nov, 2023.
6. Boere GC and Piersma T. Flyway protection and the predicament of our migrant birds: A critical look at international conservation policies and the Dutch Wadden Sea. Ocean Coast Manage 2012;**68**:157-68. doi:10.1016/j.ocecoaman.2012.05.019.
7. Si Y, Xu F, Wei J, Zhang L, Murray N, Yang R, et al. A systematic network-based migratory bird monitoring and protection system is needed in China. Sci Bull (Beijing) 2021;**66**(10):955-7. doi:10.1016/j.scib.2021.01.033.
8. Ma Z, Melville DS, Liu J, Chen Y, Yang H, Ren W, et al. Rethinking China's new great wall. Science 2014;**346**(6212):912-4. doi:10.1126/science.1257258.
9. Li J, Hughes AC and Dudgeon D. Correction: Mapping wader biodiversity along the East Asian-Australasian flyway. PLoS One 2019;**14**(4):e0215877. doi:10.1371/journal.pone.0215877.
10. Bamford M, Watkins D, Bancroft W, Tischler G and Wahl J. Migratory shorebirds of the East Asian-Australasian flyway : Population estimates and internationally important sites. . Canberra: Wetlands International, Oceania 2008:pp 237. https://www.wetlands.org/publication/migratory-shorebirds-of-the-east-asian-australasian-flyway-population-estimates-and-internationally-important-sites/. Accessed 1 Nov, 2023.
11. Zheng H, Shen G, Shang L, Lv X, Wang Q, McLaughlin N, et al. Efficacy of conservation strategies for endangered oriental white storks (Ciconia boyciana) under climate change in Northeast China. Biol Conserv 2016;**204**:367-77. doi:10.1016/j.biocon.2016.11.004.
12. Cano-Alonso LS, Grace MK, Yu Y-t and Chan S. Reversing the Decline in a Threatened Species: The Black-Faced Spoonbill Platalea minor. Diversity 2023;**15**(2):217. doi:10.3390/d15020217.
13. Gilbert M, Buuveibaatar B, Fine AE, Jambal L and Strindberg S. Declining breeding populations of White-naped Cranes in Eastern Mongolia, a ten-year update. Bird Conservation International 2016;**26**(4):490-504. doi:10.1017/S0959270915000301.
14. Xu W, Xiao Y, Zhang J, Yang W, Zhang L, Hull V, et al. Reply to Yang et al.: Coastal wetlands are not well represented by protected areas for endangered birds. Proc Natl Acad Sci U S A 2017;**114**(28):E5493-E. doi:10.1073/pnas.1706515114.
15. International B. The IUCN Red List of Threatened Species 2018: e.T22697695A131942061. 2018; doi:https://dx.doi.org/10.2305/IUCN.UK.2018-2.RLTS.T22697695A131942061.en.
16. Garidi, Fan SJ, Cao L, Zhang BX, Wang YX, Zhu BG, et al. Migration strategy of the Bohai Bay wintering population of juvenile Oriental Storks (Ciconia boyciana). Biodiv Sci 2022;**30**(5):21232. doi:10.17520/biods.2021232.
17. Ma X. *Research on captive oriental white stork (Ciconia boyciana) in energy of digestion and fledgling growth*. Master thesis. Northeast Forestry University. 2007.
18. Liu Z and Li X. The research progress of oriental white stork. Territory & Natural Resources Study 2008;(01):77-8. doi:10.16202/j.cnki.tnrs.2008.01.001.
19. Zeng S, Cheng L and Li X. The numerical distribution and conservation of oriental white

stork in China. Territory & Natural Resources Study 2003;(01):71-2. doi:10.16202/j.cnki.tnrs.2003.01.035.

20. Van den Bossche W, Berthold P, Darman Y, Andronov V, Parilov M and Querner U. Satellite-tracking helps to discover stopover sites of the threatened Oriental White Stork (Ciconia boyciana). Microwave Telemetry, Inc Newsletter 2001;**2**(1):3-4. https://hdl.handle.net/11858/00-001M-0000-002C-52EA-9. Accessed 1 Nov, 2023.
21. Theissinger K, Fernandes C, Formenti G, Bista I, Berg PR, Bleidorn C, et al. How genomics can help biodiversity conservation. Trends Genet 2023;**39**(7):545-59. doi:10.1016/j.tig.2023.01.005.
22. Supple MA and Shapiro B. Conservation of biodiversity in the genomics era. Genome Biol 2018;**19**(1):131. doi:10.1186/s13059-018-1520-3.
23. Formenti G, Theissinger K, Fernandes C, Bista I, Bombarely A, Bleidorn C, et al. The era of reference genomes in conservation genomics. Trends Ecol Evol 2022;**37**(3):197-202. doi:10.1016/j.tree.2021.11.008.
24. Allendorf FW, Hohenlohe PA and Luikart G. Genomics and the future of conservation genetics. Nat Rev Genet 2010;**11**(10):697-709. doi:10.1038/nrg2844.
25. Theissinger K, Fernandes C, Formenti G, Bista I, Berg PR, Bleidorn C, et al. How genomics can help biodiversity conservation. Trends in Genetics 2023;**39**(7):545-59. doi:10.1016/j.tig.2023.01.005.
26. Kleinman-Ruiz D, Lucena-Perez M, Villanueva B, Fernandez J, Saveljev AP, Ratkiewicz M, et al. Purging of deleterious burden in the endangered Iberian lynx. Proc Natl Acad Sci U S A 2022;**119**(11):e2110614119. doi:10.1073/pnas.2110614119.
27. Kleinman-Ruiz D, Martínez-Cruz B, Soriano L, Lucena-Perez M, Cruz F, Villanueva B, et al. Novel efficient genome-wide SNP panels for the conservation of the highly endangered Iberian lynx. BMC Genomics 2017;**18**(1) doi:10.1186/s12864-017-3946-5.
28. Abascal F, Corvelo A, Cruz F, Villanueva-Cañas JL, Vlasova A, Marcet-Houben M, et al. Extreme genomic erosion after recurrent demographic bottlenecks in the highly endangered Iberian lynx. Genome Biol 2016;**17**(1):251. doi:10.1186/s13059-016-1090-1.
29. Lucena-Perez M, Kleinman-Ruiz D, Marmesat E, Saveljev AP, Schmidt K and Godoy JA. Bottleneck-associated changes in the genomic landscape of genetic diversity in wild lynx populations. Evol Appl 2021;**14**(11):2664-79. doi:10.1111/eva.13302.
30. Saremi NF, Supple MA, Byrne A, Cahill JA, Coutinho LL, Dalen L, et al. Puma genomes from North and South America provide insights into the genomic consequences of inbreeding. Nat Commun 2019;**10**(1):4769. doi:10.1038/s41467-019-12741-1.
31. Khan A, Patel K, Shukla H, Viswanathan A, van der Valk T, Borthakur U, et al. Genomic evidence for inbreeding depression and purging of deleterious genetic variation in Indian tigers. Proc Natl Acad Sci U S A 2021;**118**(49) doi:10.1073/pnas.2023018118.
32. Dussex N, van der Valk T, Morales HE, Wheat CW, Díez-Del-Molino D, von Seth J, et al. Population genomics of the critically endangered kākāpō. Cell Genom 2021;**1**(1):100002. doi:10.1016/j.xgen.2021.100002.
33. Lieberman-Aiden E, Berkum NV, Williams L, Imakaev M, Ragoczy T, Telling A, et al. Comprehensive Mapping of Long-Range Interactions Reveals Folding Principles of the Human Genome. Science 2009;**326**(5950):289. doi:10.1126/science.1181369.
34. Lander ES and Waterman MS. Genomic mapping by fingerprinting random clones: a

mathematical analysis. Genomics 1988;**2**(3):231-9. doi:10.1016/0888-7543(88)90007-9.
35. Hu J, Fan J, Sun Z and Liu S. NextPolish: a fast and efficient genome polishing tool for long-read assembly. Bioinformatics 2020;**36**(7):2253-5. doi:10.1093/bioinformatics/btz891.
36. Li H and Durbin R. Fast and accurate long-read alignment with Burrows–Wheeler transform. Bioinformatics 2010;**26**(5):589-95. doi:10.1093/bioinformatics/btp698.
37. Durand NC, Shamim MS, Machol I, Rao SS, Huntley MH, Lander ES, et al. Juicer provides a one-click system for analyzing loop-resolution Hi-C experiments. Cell Syst 2016;**3**(1):95-8. doi:10.1016/j.cels.2016.07.002.
38. Manni M, Berkeley MR, Seppey M, Simão FA and Zdobnov EM. BUSCO Update: Novel and Streamlined Workflows along with Broader and Deeper Phylogenetic Coverage for Scoring of Eukaryotic, Prokaryotic, and Viral Genomes. Mol Biol Evol 2021;**38**(10):4647-54. doi:10.1093/molbev/msab199.
39. Xu Z and Wang H. LTR_FINDER: an efficient tool for the prediction of full-length LTR retrotransposons. Nucleic Acids Res 2007;**35**(Web Server issue):W265-8. doi:10.1093/nar/gkm286.
40. Han Y and Wessler SR. MITE-Hunter: a program for discovering miniature inverted-repeat transposable elements from genomic sequences. Nucleic Acids Res 2010;**38**(22):e199. doi:10.1093/nar/gkq862.
41. Flynn JM, Hubley R, Goubert C, Rosen J, Clark AG, Feschotte C, et al. RepeatModeler2 for automated genomic discovery of transposable element families. Proc Natl Acad Sci U S A 2020;**117**(17):9451-7. doi:10.1073/pnas.1921046117.
42. Tarailo-Graovac M and Chen N. Using RepeatMasker to identify repetitive elements in genomic sequences. Curr Protoc Bioinformatics 2009;**Chapter 4**:Unit 4.10. doi:10.1002/0471250953.bi0410s25.
43. Jurka J, Kapitonov VV, Pavlicek A, Klonowski P, Kohany O and Walichiewicz J. Repbase Update, a database of eukaryotic repetitive elements. Cytogenet Genome Res 2005;**110**(1-4):462-7. doi:10.1159/000084979.
44. Benson G. Tandem repeats finder: a program to analyze DNA sequences. Nucleic Acids Res 1999;**27**(2):573-80. doi:10.1093/nar/27.2.573.
45. Korf I. Gene finding in novel genomes. BMC Bioinformatics 2004;**5**(1):59. doi:10.1186/1471-2105-5-59.
46. Majoros WH, Pertea M and Salzberg SL. TigrScan and GlimmerHMM: two open source ab initio eukaryotic gene-finders. Bioinformatics 2004;**20**(16):2878-9. doi:10.1093/bioinformatics/bth315.
47. Keller O, Kollmar M, Stanke M and Waack S. A novel hybrid gene prediction method employing protein multiple sequence alignments. Bioinformatics 2011;**27**(6):757-63. doi:10.1093/bioinformatics/btr010.
48. Bolger AM, Lohse M and Usadel B. Trimmomatic: a flexible trimmer for Illumina sequence data. Bioinformatics 2014;**30**(15):2114-20. doi:10.1093/bioinformatics/btu170.
49. Haas BJ, Papanicolaou A, Yassour M, Grabherr M, Blood PD, Bowden J, et al. De novo transcript sequence reconstruction from RNA-seq using the Trinity platform for reference generation and analysis. Nat Protoc 2013;**8**(8):1494-512. doi:10.1038/nprot.2013.084.

50. Haas BJ, Salzberg SL, Zhu W, Pertea M, Allen JE, Orvis J, et al. Automated eukaryotic gene structure annotation using EVidenceModeler and the Program to Assemble Spliced Alignments. Genome Biol 2008;**9**(1):R7-R22. doi:10.1186/gb-2008-9-1-r7.
51. Mount DW. Using the Basic Local Alignment Search Tool (BLAST). CSH protocols 2007;**2007**:pdb.top17. doi:10.1101/pdb.top17.
52. Birney E, Clamp M and Durbin R. GeneWise and Genomewise. Genome Res 2004;**14**(5):988-95. doi:10.1101/gr.1865504.
53. Campbell MS, Holt C, Moore B and Yandell M. Genome Annotation and Curation Using MAKER and MAKER-P. Curr Protoc Bioinformatics 2014;**48**(1):4.11.1-4..39. doi:10.1002/0471250953.bi0411s48.
54. Altschul SF, Gish W, Miller W, Myers EW and Lipman DJ. Basic local alignment search tool. J Mol Biol 1990;**215**(3):403-10. doi:10.1016/S0022-2836(05)80360-2.
55. Wang Y, Tang H, Debarry JD, Tan X, Li J, Wang X, et al. MCScanX: a toolkit for detection and evolutionary analysis of gene synteny and collinearity. Nucleic Acids Res 2012;**40**(7):e49. doi:10.1093/nar/gkr1293.
56. Krzywinski M, Schein J, Birol I, Connors J, Gascoyne R, Horsman D, et al. Circos: An information aesthetic for comparative genomics. Genome Res 2009;**19**(9):1639-45. doi:10.1101/gr.092759.109.
57. Lam-Tung N, Schmidt HA, Arndt VH, Quang MB and Evolution. IQ-TREE: A Fast and Effective Stochastic Algorithm for Estimating Maximum-Likelihood Phylogenies. Mol Biol Evol 2015;**32**(1):268-74. doi:10.1101/gr.092759.109.
58. Yang Z. PAML 4: Phylogenetic Analysis by Maximum Likelihood. Mol Biol Evol 2007;**24**(8):1586–91. doi:10.1101/gr.092759.109.
59. Benton MJ and Donoghue PC. Paleontological evidence to date the tree of life. Mol Biol Evol 2007;**24**(1):26-53. doi:10.1093/molbev/msl150.
60. Freed D, Aldana R, Weber JA and Edwards JS. The Sentieon Genomics Tools - A fast and accurate solution to variant calling from next-generation sequence data. bioRxiv 2017:115717. doi:10.1101/115717.
61. Danecek P, Auton A, Abecasis G, Albers CA, Banks E, DePristo MA, et al. The variant call format and VCFtools. Bioinformatics 2011;**27**(15):2156-8. doi:10.1093/bioinformatics/btr330.
62. Chang CC, Chow CC, Tellier LC, Vattikuti S, Purcell SM and Lee JJ. Second-generation PLINK: rising to the challenge of larger and richer datasets. Gigascience 2015;**4**:7. doi:10.1186/s13742-015-0047-8.
63. Alexander DH, Novembre J and Lange K. Fast model-based estimation of ancestry in unrelated individuals. Genome Res 2009;**19**(9):1655-64. doi:10.1101/gr.092759.109.
64. Danecek P, Bonfield JK, Liddle J, Marshall J, Ohan V, Pollard MO, et al. Twelve years of SAMtools and BCFtools. Gigascience. 2021,10(2):giab008. doi: 10.1093/gigascience/giab008
65. Feng S, Fang Q, Barnett R, Li C, Han S, Kuhlwilm M, et al. The Genomic Footprints of the Fall and Recovery of the Crested Ibis. Curr Biol 2019;**29**:340-9. doi:10.1016/j.cub.2018.12.008.
66. Li H and Durbin R. Inference of human population history from individual whole-genome sequences. Nature 2011;**475**(7357):493-6. doi:10.1038/nature10231.

67. Terhorst J, Kamm JA and Song YS. Robust and scalable inference of population history from hundreds of unphased whole genomes. Nat Genet 2016;**49**(2):303-9. doi:10.1038/ng.3748.
68. Boitard S, Rodriguez W, Jay F, Mona S and Austerlitz F. Inferring Population Size History from Large Samples of Genome-Wide Molecular Data - An Approximate Bayesian Computation Approach. PLoS Genet 2016;**12**(3):e1005877. doi:10.1371/journal.pgen.1005877.
69. Li H, Coghlan A, Ruan J, Coin LJ, Hériché JK, Osmotherly L, et al. TreeFam: a curated database of phylogenetic trees of animal gene families. Nucleic Acids Res 2006;**34**(Database issue):D572-80. doi:10.1093/nar/gkj118.
70. De Bie T, Cristianini N, Demuth JP and Hahn MW. CAFE: a computational tool for the study of gene family evolution. Bioinformatics 2006;**22**(10):1269-71. doi:10.1093/bioinformatics/btl097.
71. Team RDC. R: A language and environment for statistical computing. R Foundation for Statistical Computing 2012.
72. Wu T, Hu E, Xu S, Chen M, Guo P, Dai Z, et al. clusterProfiler 4.0: A universal enrichment tool for interpreting omics data. The Innovation 2021;**2**(3):100141. doi:10.1016/j.xinn.2021.100141.
73. Supek F, Bošnjak M, Škunca N and Šmuc T. REVIGO summarizes and visualizes long lists of gene ontology terms. PLoS One 2011;**6**(7):e21800. doi:10.1371/journal.pone.0021800.
74. Browning BL, Zhou Y and Browning SR. A One-Penny Imputed Genome from Next-Generation Reference Panels. Am J Hum Genet 2018;**103**(3):338-48. doi:10.1016/j.ajhg.2018.07.015.
75. Voight BF, Kudaravalli S, Wen X and Pritchard JK. A map of recent positive selection in the human genome. PLoS Biol 2006;**4**(3):e72. doi:10.1371/journal.pbio.0040072.
76. Zhan X, Pan S, Wang J, Dixon A, He J, Muller MG, et al. Peregrine and saker falcon genome sequences provide insights into evolution of a predatory lifestyle. Nat Genet 2013;**45**(5):563-6. doi:10.1038/ng.2588.
77. von Seth J, van der Valk T, Lord E, Sigeman H, Olsen R-A, Knapp M, et al. Genomic trajectories of a near-extinction event in the Chatham Island black robin. BMC Genomics 2022;**23**(1):747. doi:10.1186/s12864-022-08963-1.
78. Wang P, Burley JT, Liu Y, Chang J, Chen, Lu Q, et al. Genomic Consequences of Long-Term Population Decline in Brown Eared Pheasant. Mol Biol Evol 2021;**38**(1):263-73. doi:10.1093/molbev/msaa213.
79. Dong F, Kuo H-C, Chen G-L, Wu F, Shan P-F, Wang J, et al. Population genomic, climatic and anthropogenic evidence suggest the role of human forces in endangerment of green peafowl. Proc Biol Sci 2021;**288**(1948):20210073. doi:doi:10.1098/rspb.2021.0073.
80. Bertorelle G, Raffini F, Bosse M, Bortoluzzi C, Iannucci A, Trucchi E, et al. Genetic load: genomic estimates and applications in non-model animals. Nat Rev Genet 2022;**23**(8):492-503. doi:10.1038/s41576-022-00448-x.
81. Hare MP, Nunney L, Schwartz MK, Ruzzante DE, Burford M, Waples RS, et al. Understanding and estimating effective population size for practical application in marine species management. Conserv Biol 2011;**25**(3):438-49. doi:10.1111/j.1523-

1739.2010.01637.x.
82. Brønnvik H, Safi K, Vansteelant WM, Byholm P and Nourani E. Experience does not change the importance of wind support for migratory route selection by a soaring bird. R Soc Open Sci 2022;**9**(12):220746. doi:10.1098/rsos.220746.
83. Aikens EO, Nourani E, Fiedler W, Wikelski M and Flack A. Learning shapes the development of migratory behavior. Proc Natl Acad Sci U S A 2024;**121**(12):e2306389121. doi:10.1073/pnas.2306389121.
84. Gu Z, Pan S, Lin Z, Hu L, Dai X, Chang J, et al. Climate-driven flyway changes and memory-based long-distance migration. Nature 2021;**591**(7849):259-64. doi:10.1038/s41586-021-03265-0.
85. Kishkinev D, Chernetsov N, Heyers D and Mouritsen H. Migratory Reed Warblers Need Intact Trigeminal Nerves to Correct for a 1,000 km Eastward Displacement. PLoS One 2013;**8**(6):e65847. doi:10.1371/journal.pone.0065847.
86. Xu J, Jarocha LE, Zollitsch T, Konowalczyk M, Henbest KB, Richert S, et al. Magnetic sensitivity of cryptochrome 4 from a migratory songbird. Nature 2021;**594**(7864):535-40. doi:10.1038/s41586-021-03618-9.
87. Zhang T, Chen P, Li W, Sha S, Wang Y, Yuan Z, et al. Cognitive deficits in mice lacking Nsun5, a cytosine-5 RNA methyltransferase, with impairment of oligodendrocyte precursor cells. Glia 2019;**67**(4):688-702. doi:10.1002/glia.23565.
88. Zhang J, Wang Y, Chi Z, Keuss MJ, Pai YM, Kang HC, et al. The AAA+ ATPase Thorase regulates AMPA receptor-dependent synaptic plasticity and behavior. Cell 2011;**145**(2):284-99. doi:10.1016/j.cell.2011.03.016.
89. Cassens RG and Cooper CC. Red and White Muscle. In: Chichester CO, Mrak EM and Stewart GF, editors. Advances in Food Research. Academic Press; 1971. p. 1-74.
90. Barge L and Mark EW. Muscle fiber types in a migratory and a non-magratory avian species. In: 2012. https://esirc.emporia.edu/handle/123456789/1372. Accessed 1 Nov, 2023.
91. Di Marco M, Venter O, Possingham HP and Watson JEM. Changes in human footprint drive changes in species extinction risk. Nat Commun 2018;**9**(1):4621. doi:10.1038/s41467-018-07049-5.
92. Pimm SL, Jenkins CN, Abell R, Brooks TM, Gittleman JL, Joppa LN, et al. The biodiversity of species and their rates of extinction, distribution, and protection. Science 2014;**344**(6187):1246752. doi:10.1126/science.1246752.
93. Lees AC, Haskell L, Allinson T, Bezeng SB, Burfield IJ, Renjifo LM, et al. State of the World's Birds. Annu Rev Environ Resour 2022;**47**:231-60. doi:10.1146/annurev-environ-112420-014642.
94. Green RE, Braun EL, Armstrong J, Earl D, Nguyen N, Hickey G, et al. Three crocodilian genomes reveal ancestral patterns of evolution among archosaurs. Science 2014;**346**(6215):1254449. doi:10.1126/science.1254449.
95. Ouborg NJ, Pertoldi C, Loeschcke V, Bijlsma RK and Hedrick PW. Conservation genetics in transition to conservation genomics. Trends Genet 2010;**26**(4):177-87. doi:10.1016/j.tig.2010.01.001.
96. Kitts PA, Church DM, Thibaud-Nissen F, Choi J, Hem V, Sapojnikov V, et al. Assembly: a resource for assembled genomes at NCBI. Nucleic Acids Res 2016;**44**(D1):D73-80.

doi:10.1093/nar/gkv1226.
97. Takagi N and Sasaki M. A phylogenetic study of bird karyotypes. Chromosoma 1974;**46**(1):91-120. doi:10.1007/BF00332341.
98. Francisco M and Galetti Jr P. First karyotypical description of two American Ciconiiform birds, Mycteria americana (Ciconiidae) and Platalea ajaja (Threskiornithidae) and its significance for the chromosome evolutionary and biological conservation approaches. Genet Mol Biol 2000;**23**(4):799-801. doi:10.1590/S1415-47572000000400015.
99. Flamio R, Jr. and Ramstad KM. Chromosome-level genome of the wood stork (Mycteria americana) provides insight into avian chromosome evolution. J Hered 2024;**115**(2):230-9. doi:10.1093/jhered/esad077.
100. Yang S, Lan T, Zhang Y, Wang Q, Li H, Dussex N, et al. Genomic investigation of the Chinese alligator reveals wild-extinct genetic diversity and genomic consequences of their continuous decline. Mol Ecol Resour 2023;**23**(1):294-311. doi:10.1111/1755-0998.13702.
101. Robinson JA, Kyriazis CC, Nigenda-Morales SF, Beichman AC, Rojas-Bracho L, Robertson KM, et al. The critically endangered vaquita is not doomed to extinction by inbreeding depression. Science 2022;**376**(6593):635-9. doi:10.1126/science.abm1742.
102. Westbury MV, Petersen B, Garde E, Heide-Jørgensen MP and Lorenzen ED. Narwhal Genome Reveals Long-Term Low Genetic Diversity despite Current Large Abundance Size. iScience 2019;**15**:592-9. doi:10.1016/j.isci.2019.03.023.
103. Wang P, Hou R, Wu Y, Zhang Z, Que P and Chen P. Genomic status of yellow-breasted bunting following recent rapid population decline. iScience 2022;**25**(7):104501. doi:10.1016/j.isci.2022.104501.
104. Gao C, Xing W, Liu H, Wang C, Han D and Wang G. Holocene wetland evolution in Northeast China and its responses to global change. Quaternary Sciences 2018;**38**(4):854-63. doi:10.11928/j.issn.1001-7410.2018.04.05.
105. Ye Y and Yao C. Geographical discovery and early economic development in the Far East Pacific region of Russia. Journal of Heihe University 2023;**14**(12):19-23.
106. Han M. Agricultural exploitation and population migration in northeast China in history. Chinese Landscape Architecture 2021;**37**(10):6-10. doi:10.19775/j.cla.2021.10.0006.
107. Cui H, Wu T, Liu J, Liu W, Li Z, Cheng X, et al. Effects of succession processes of marsh wetland and farmland on groundwater in the Sanjiang Plain. Hydrogeology & Engineering Geology 2023;**50**(06):51-8. doi:10.16030/j.cnki.issn.1000-3665.202306022.
108. Dahmer TD. Sanjiang Plain and Wetlands Along the Ussuri and Amur Rivers: Amur River Basin (Russia and China). In: Finlayson CM, Milton GR, Prentice RC and Davidson NC, editors. The Wetland Book: II: Distribution, Description and Conservation. Dordrecht: Springer Netherlands; 2016. p. 1-13. doi: 10.1007/978-94-007-4001-3
109. Wei F, Han M, Han G, Wang M, Tian L, Zhu J, et al. Reclamation-oriented spatiotemporal evolution of coastal wetland along Bohai Rim, China. Hai Yang Xue Bao 2022;**41**(9):192-204. doi:10.1007/s13131-022-1987-3.
110. Zhong X and Kang H. Recent geo-environmental changes in the Bohai Bay coast. Quaternary Sciences 2002;**22**(02):131-5+97-98.
111. Ga R. *The distribution and population size and trend of Oriential Stork (Ciconia boyciana) and the habitat selection of the overwintering group in Bohai Bay*. Master

thesis. Inner Mongolia Normal University, 2021.

112. Bauer S and Hoye BJ. Migratory animals couple biodiversity and ecosystem functioning worldwide. Science 2014;**344**(6179):1242552. doi:10.1126/science.1242552.

113. Kokko H. Competition for early arrival in migratory birds. J Anim Ecol 1999;**68**(5):940-50. doi:10.1046/j.1365-2656.1999.00343.x.

114. Flack A, Aikens EO, Kölzsch A, Nourani E, Snell KRS, Fiedler W, et al. New frontiers in bird migration research. Curr Biol 2022;**32**(20):R1187-R99. doi:10.1016/j.cub.2022.08.028.

115. Shao Y, Wang XB, Zhang JJ, Li ML, Wu SS, Ma XY, et al. Genome and single-cell RNA-sequencing of the earthworm Eisenia andrei identifies cellular mechanisms underlying regeneration. Nat Commun 2020;**11**(1):2656. doi:10.1038/s41467-020-16454-8.

116. Cole TL, Zhou C, Fang M, Pan H, Ksepka DT, Fiddaman SR, et al. Genomic insights into the secondary aquatic transition of penguins. Nat Commun 2022;**13**(1):3912. doi:10.1038/s41467-022-31508-9.

117. Yang S, Liu Y, Zhao X, Chen J, Li H, Liang H, et al. Supporting data for "Genomic exploration of the endangered oriental stork, Ciconia boyciana, sheds light on migration adaptation and future conservation" GigaScience Database. 2024. https://doi.org/10.5524/102556

Click here to access/download;Figure;Figure1.pdf

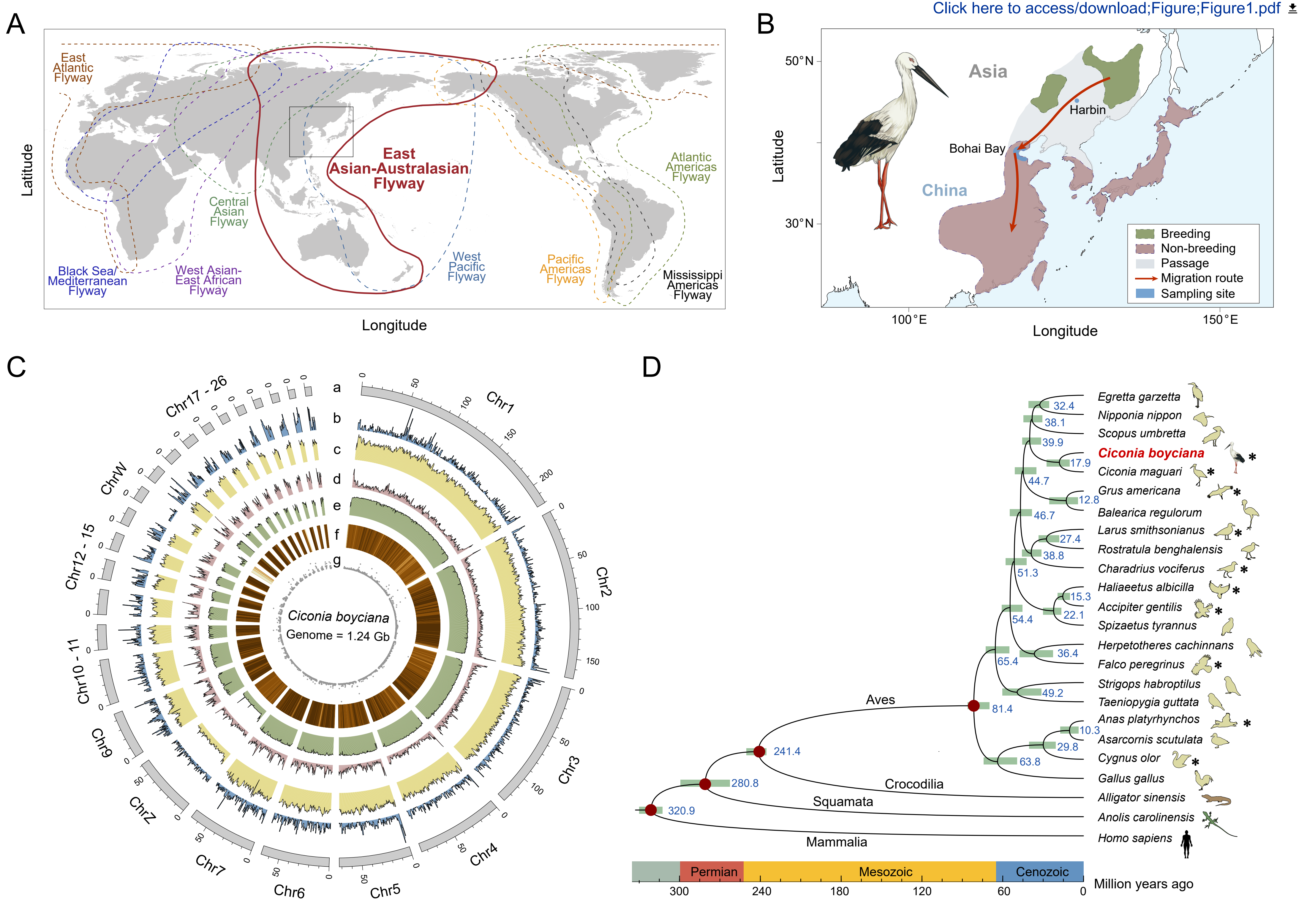

Click here to access/download;Figure;Figure2.pdf

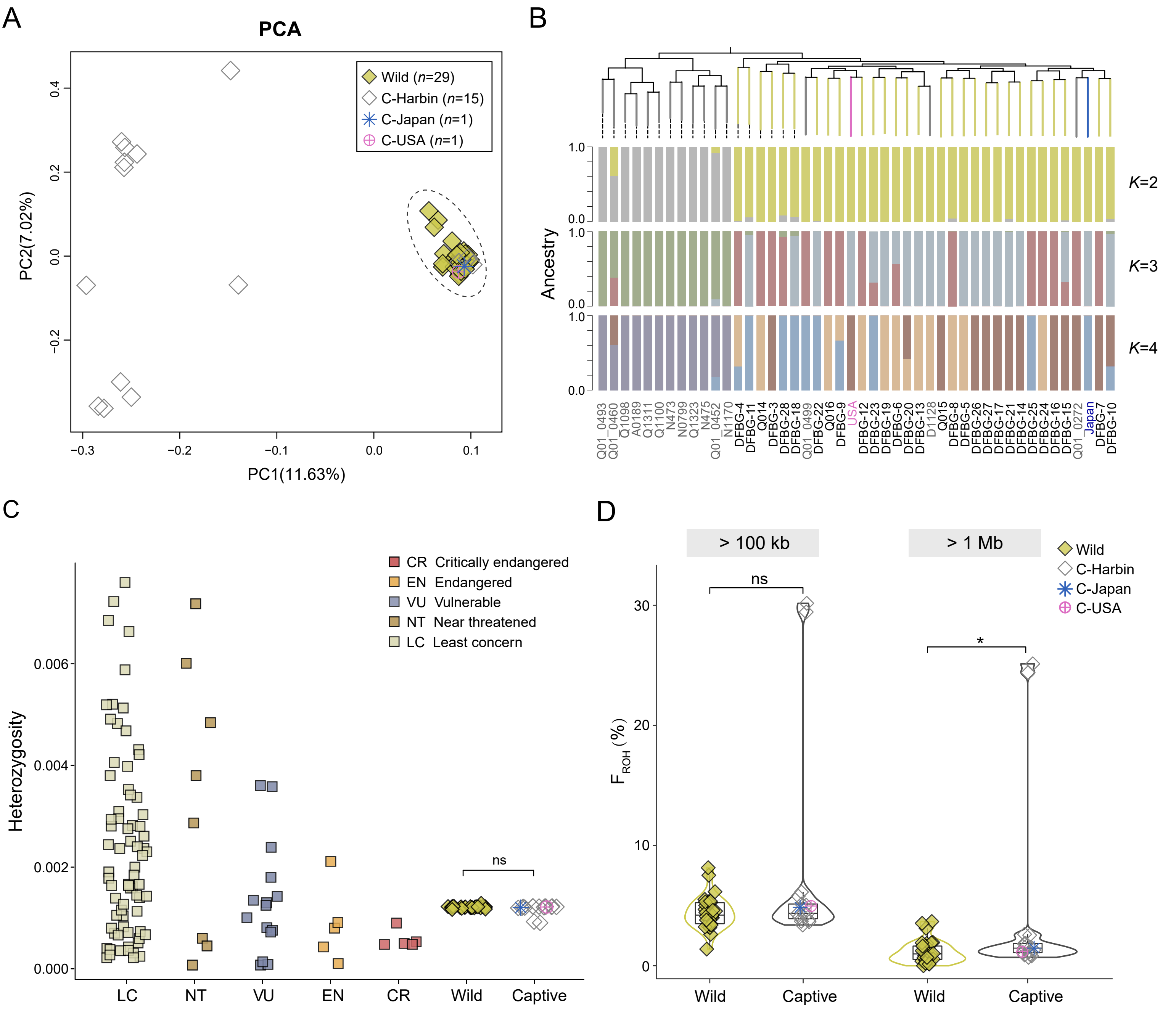

Click here to access/download;Figure;Figure3.pdf

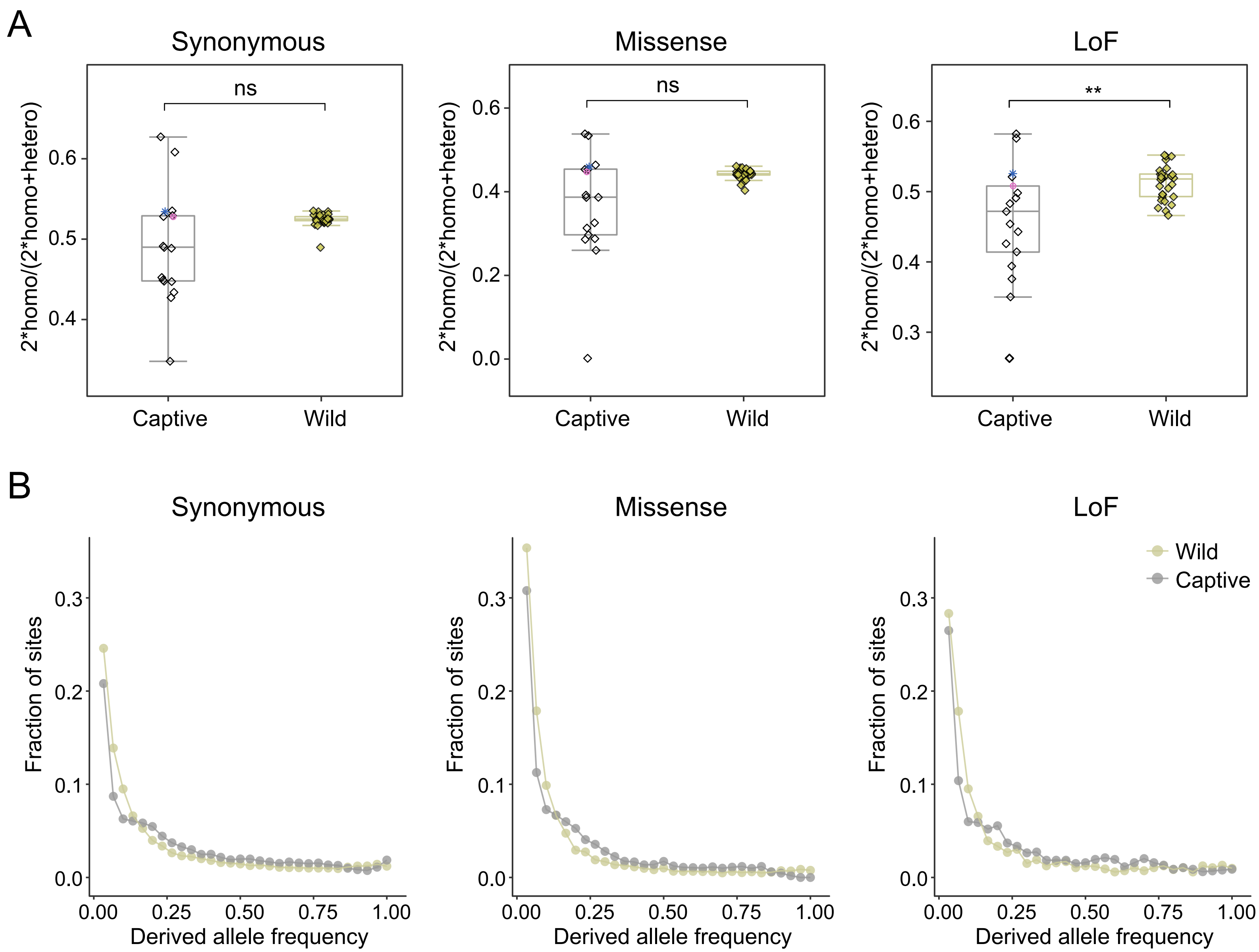

Click here to access/download;Figure;Figure4.pdf

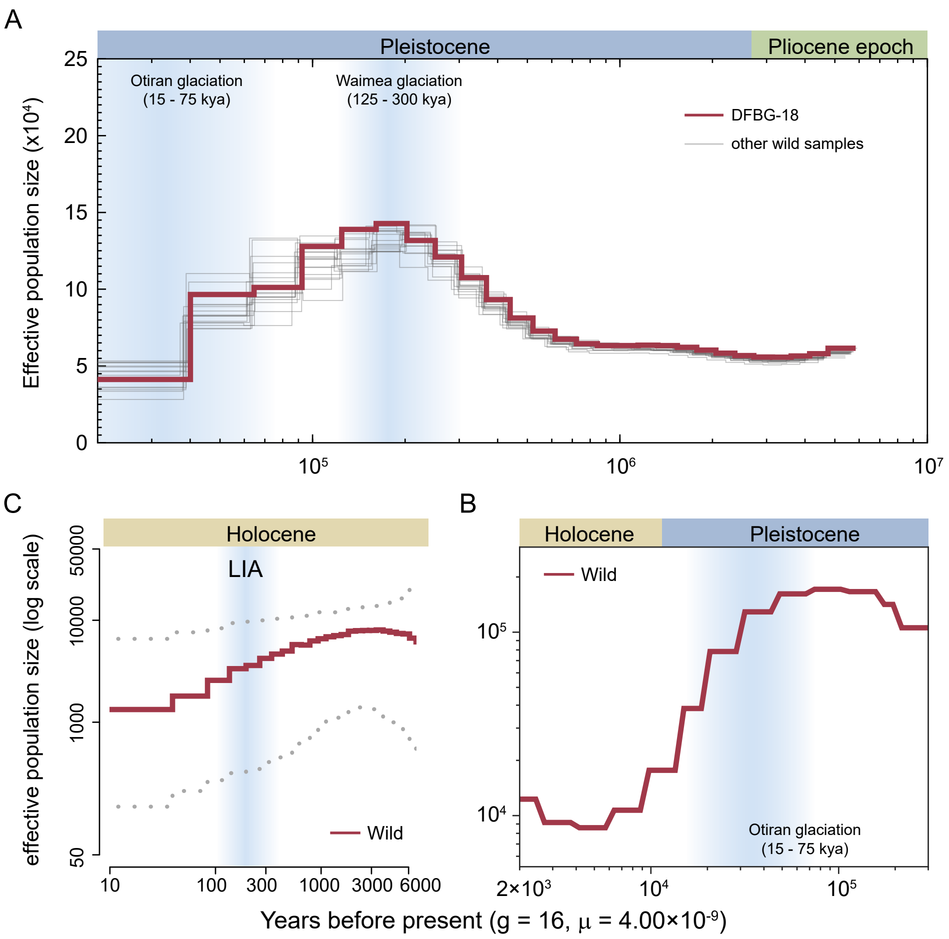

Click here to access/download;Figure;Figure5.pdf

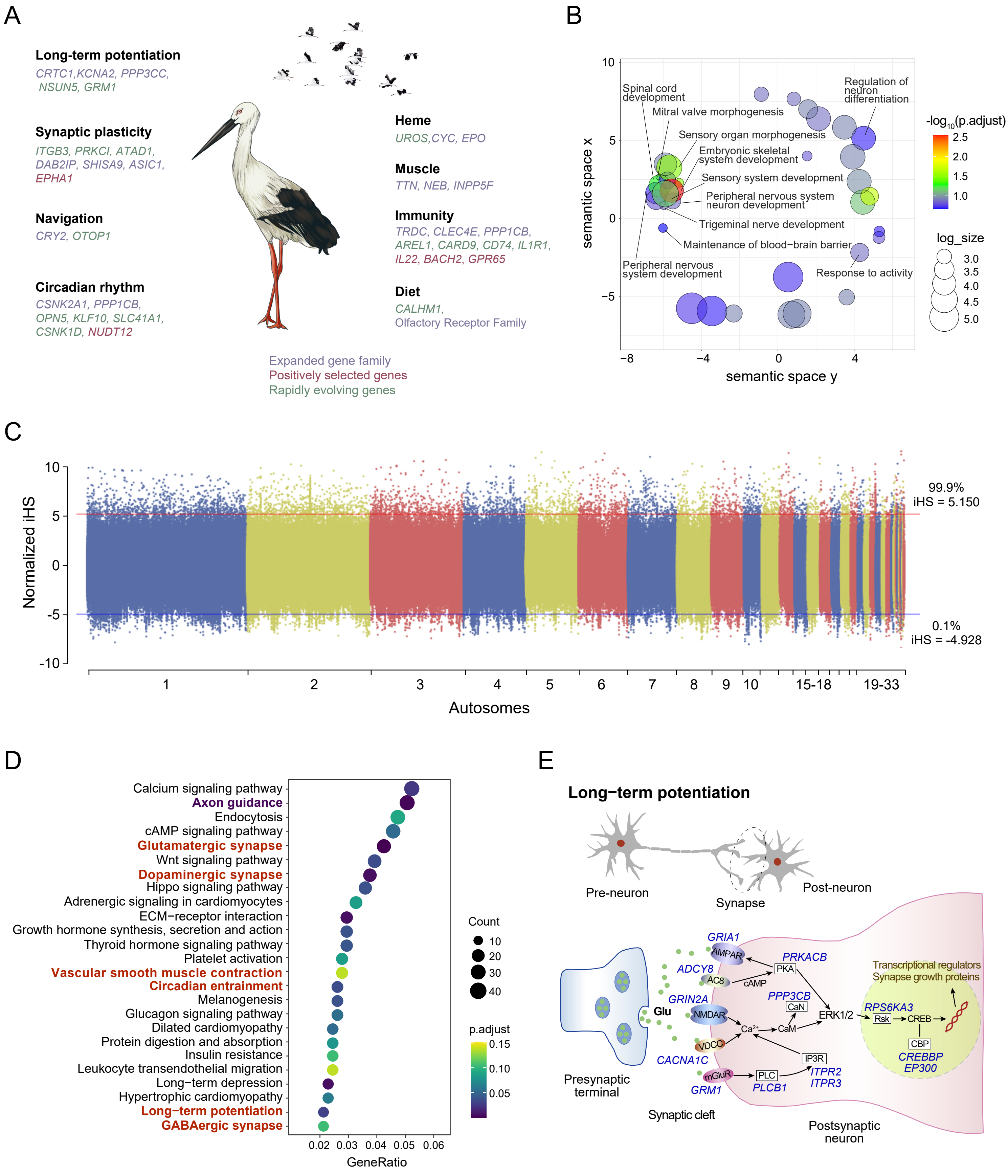

Click here to access/download
Supplementary Material
Supplementary-Figures.docx

Click here to access/download
Supplementary Material
Supplementary-tables.xlsx
