# Supplementary material for: Genomic exploration of the endangered oriental stork, Ciconia boyciana, sheds light on migration adaptation and future conservation
Source: Gigascience. 2024 Oct 22;13:giae081. doi: 10.1093/gigascience/giae081 (PMC11494145; doi:10.1093/gigascience/giae081)
Supplement: giae081_Supplemental_Files [file giae081_supplemental_files.zip › Supplementary-Figures.docx]

**Genomic exploration of the endangered oriental stork, *Ciconia boyciana*, shed lights on migration adaptation and future conservation**

**Table of Contents:**

| Supplementary Figure S1 | Page 1 |
| --- | --- |
| Supplementary Figure S2 | Page 2 |
| Supplementary Figure S3 | Page 3 |
| Supplementary Figure S4 | Page 4 |
| Supplementary Figure S5 | Page 5 |
| Supplementary Figure S6 | Page 6 |
| Supplementary Figure S7 | Page 7 |
| Supplementary Figure S8 | Page 8 |
| Supplementary Figure S9 | Page 9 |
| Supplementary Figure S10 | Page 10 |
| Supplementary Figure S11 | Page 11 |
| Supplementary Figure S12 | Page12 |
| Supplementary Figure S13 | Page13 |
| Supplementary Figure S14 | Page14 |
| Supplementary Figure S15 | Page15 |


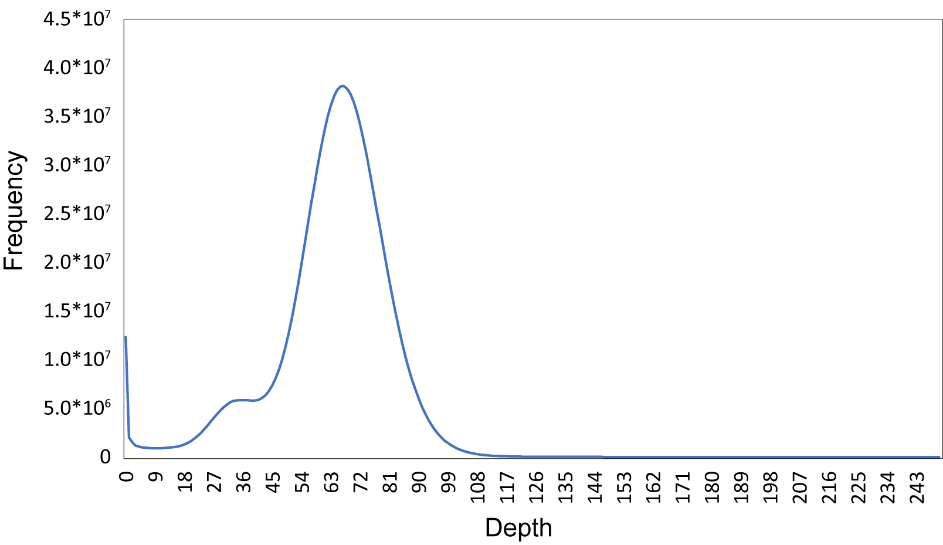


**Figure S1. The distribution of 21-mer for the genome size estimation of the oriental stork.**

The X-axis is the depth of K-mers derived from the sequencing reads and the Y-axis is the frequency of the K-mer depth.


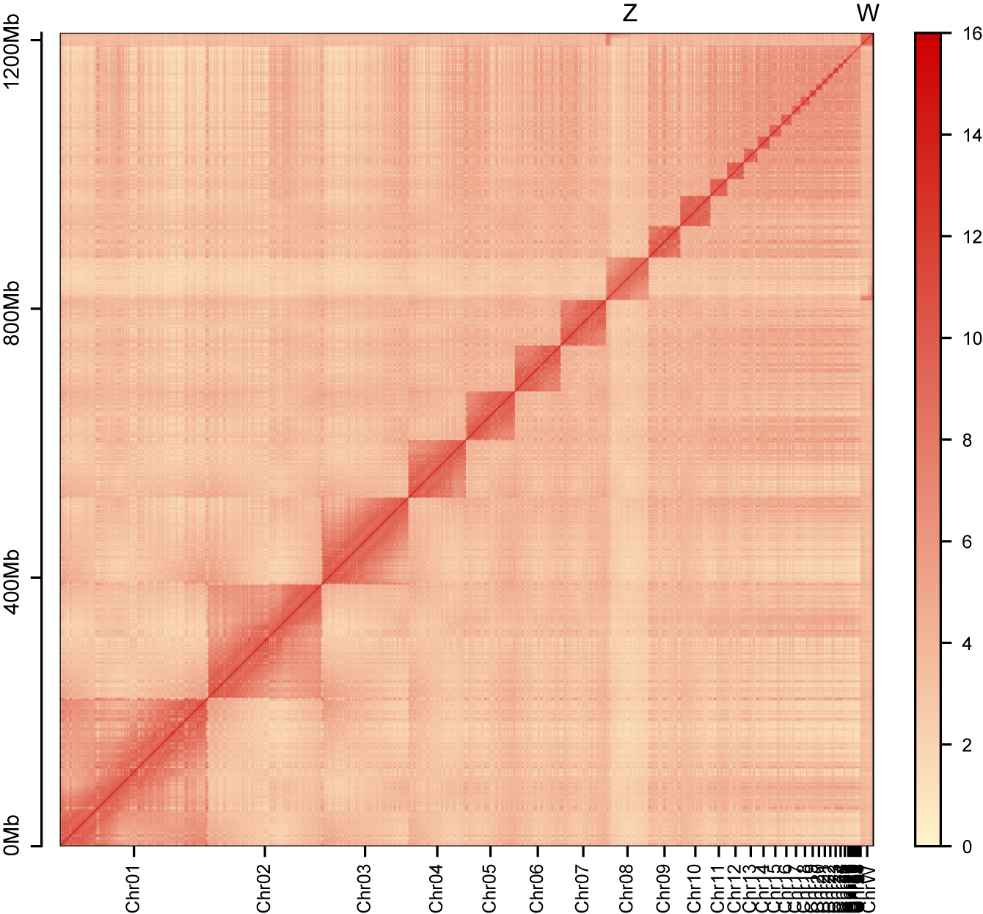


**Figure S2. Heatmap of Hi-C chromosomal interaction density among all 35 chromosomes.**


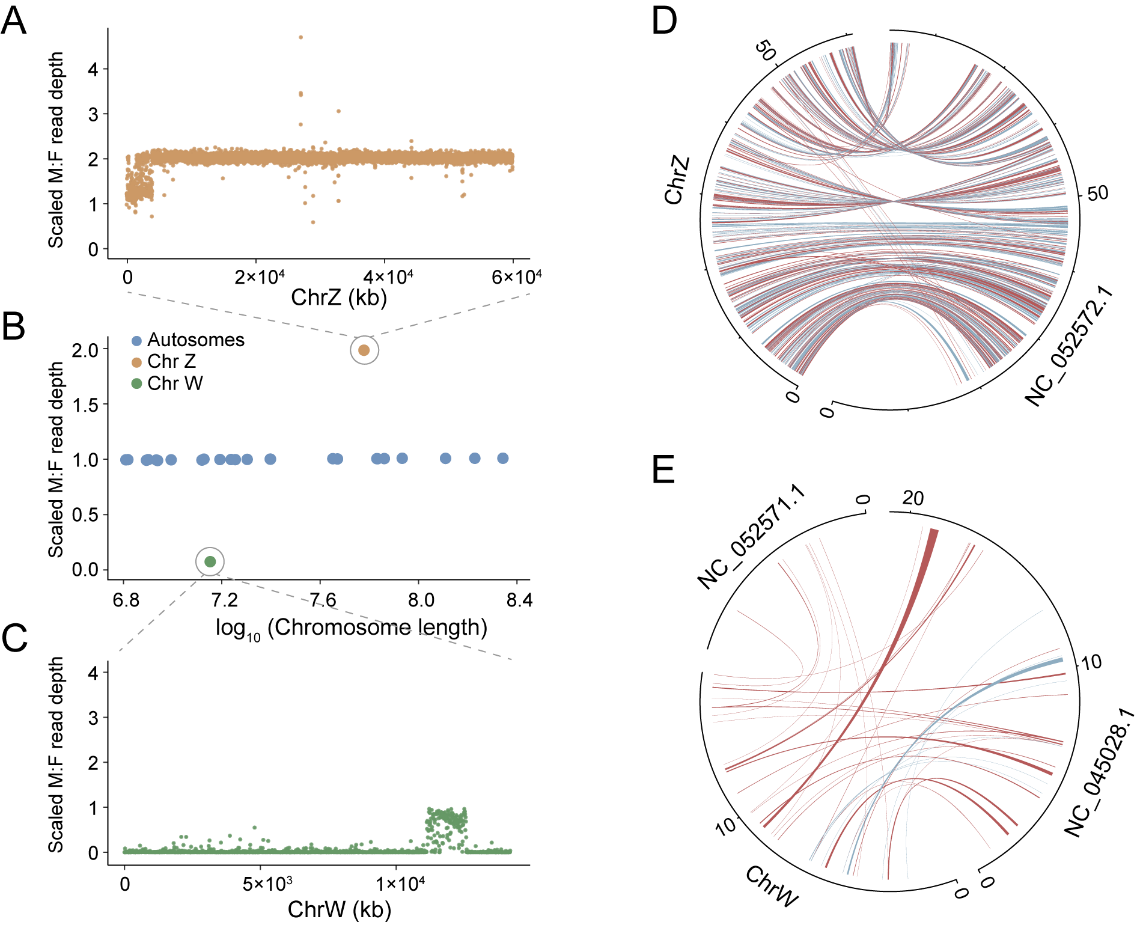


**Figure S3. Identification of sex chromosomes in the oriental stork genome.** (A) The ratio of sequencing depth between male and female individuals in a 10kbp window across the Z chromosome. (B) The ratio of the sequencing depth between male and female individuals of each chromosome larger than 5 Mb. The orange dot represented the ChrZ, the green dot represented the ChrW, and the blue dots represented autosomes. The expected ratio is 1:1, the Z chromosome is expected to be 2:1 and the W chromosome is 0:1. (C) The ratio of sequencing depth between male and female individuals in a 10kbp window across the W chromosome. (D) Synteny analysis of Z chromosome between the oriental stork and *G. gallus*. (E) Synteny analysis of W chromosome between the oriental stork and *G. gallus*, and *T. guttata*. Red lines indicated genes on the positive strand and blue lines indicated genes on the negative strand of oriental stork genome aligned with that of the other bird genome.


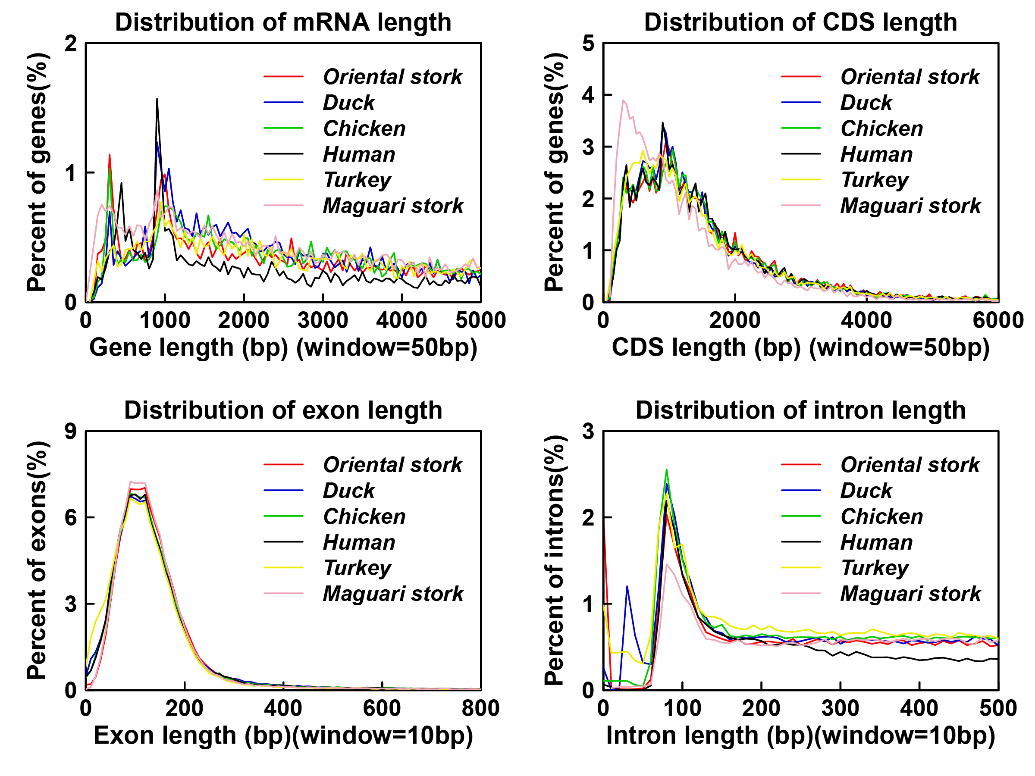


**Figure S4. Comparison of gene characteristics of avian species and human.**


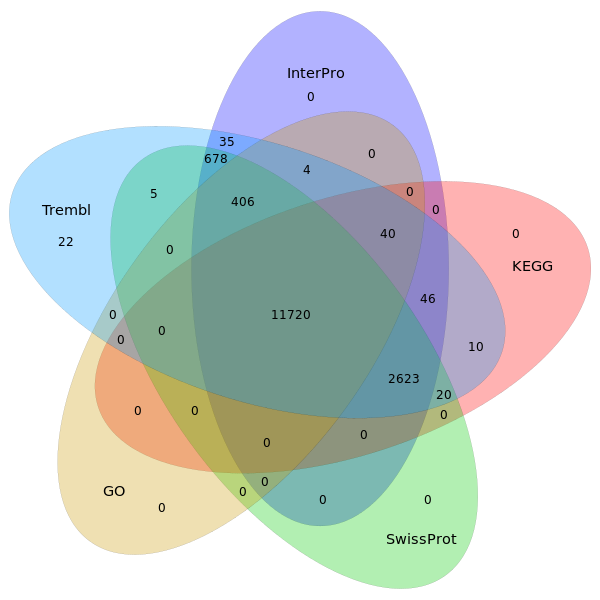


**Figure S5. Venn diagram representing the functional annotation of the oriental stork gene set.**


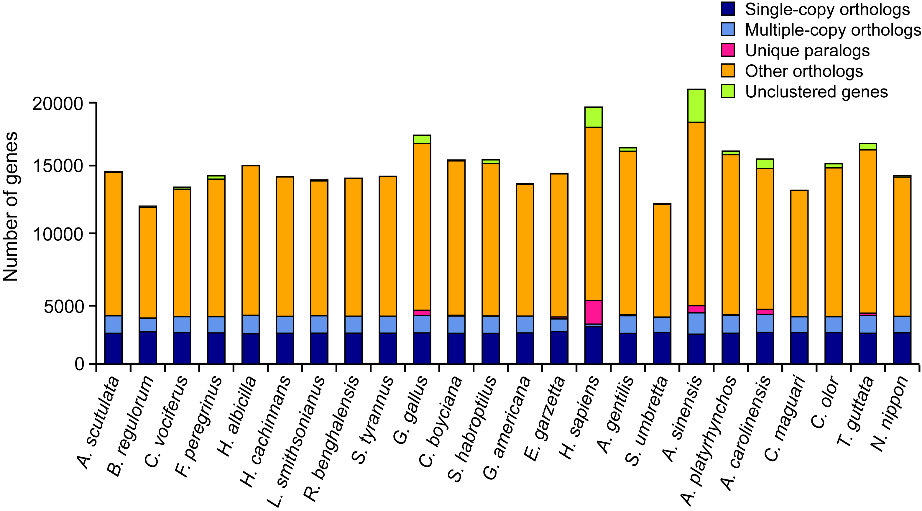


**Figure S6. Comparison of the gene repertoires of 24 vertebrate genomes.** Single-copy orthologues, multi-copy orthologues, unique paralogs, other orthologs and unclustered genes were shown in dark blue, sky blue, rose red, orange and light green, respectively.

**
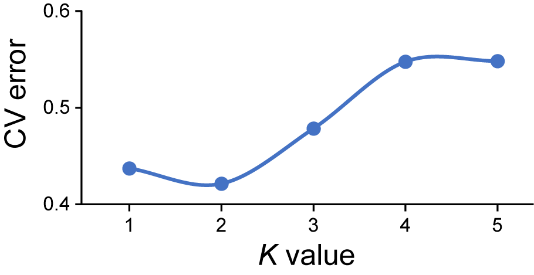
**

**Figure S7. Cross validation (CV) error in the ADMIXTURE analysis.** The lowest value was obtained when *K*=2.

**
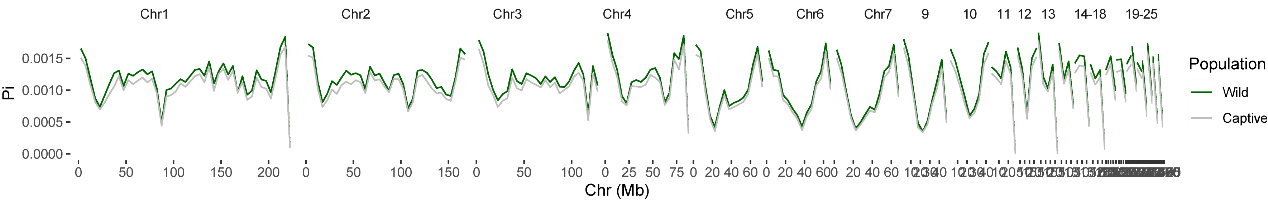
**

**Figure S8. Nucleotide diversity (π) across 25 autosomes in wild and captive populations, respectively, by sliding a 5-Mb window.**


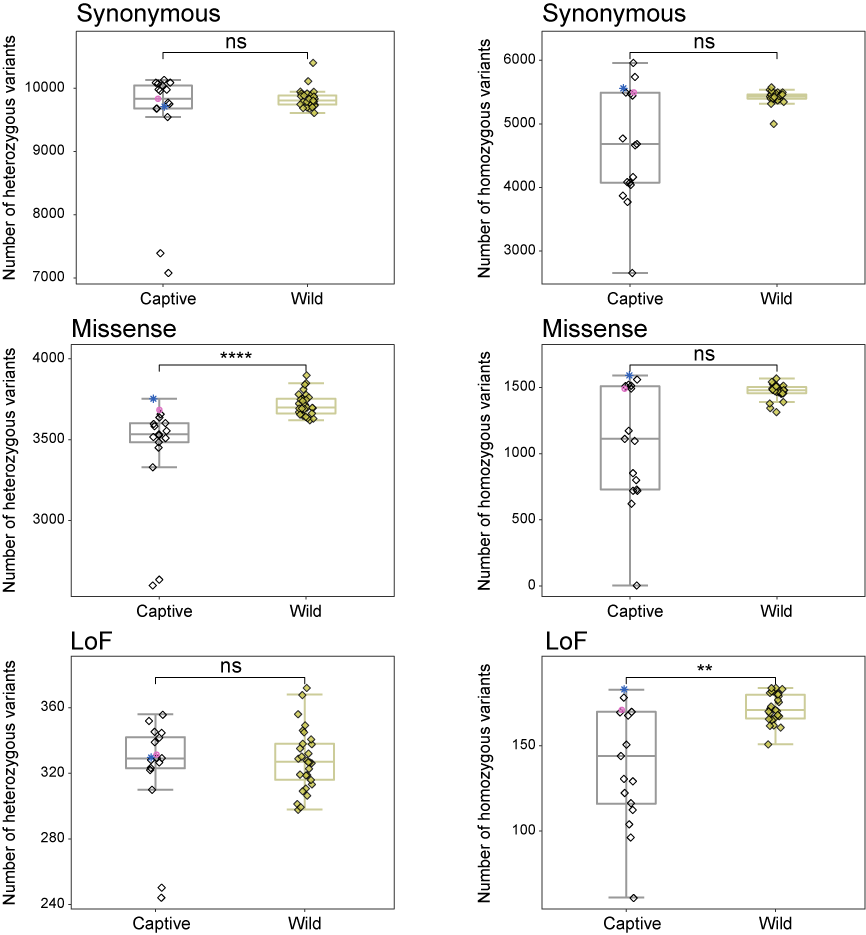


**Figure S9. Statistics of the number of heterozygous and homozygous loci for synonymous, missense and LoF mutations in each individual.**


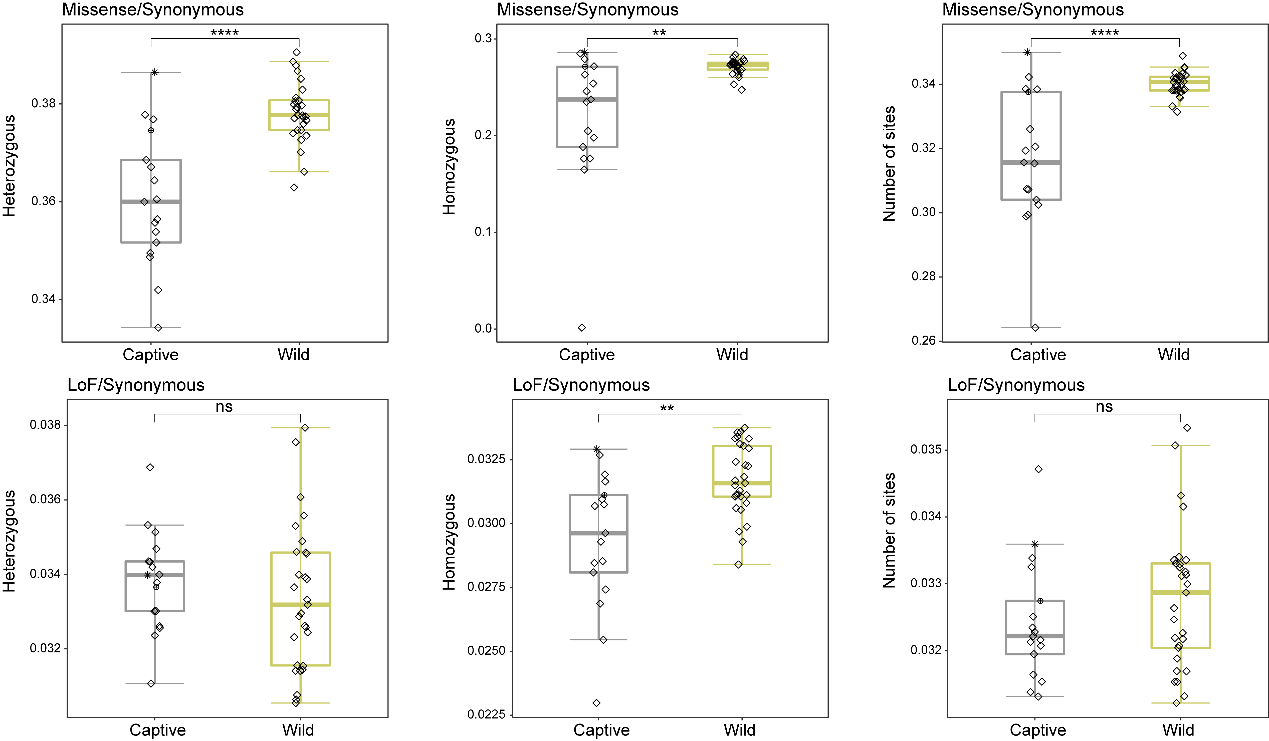


**Figure S10. Statistics of the frequency of heterozygous, homozygous loci and sites of missense and LoF mutations, scaled by synonymous mutations.**

**
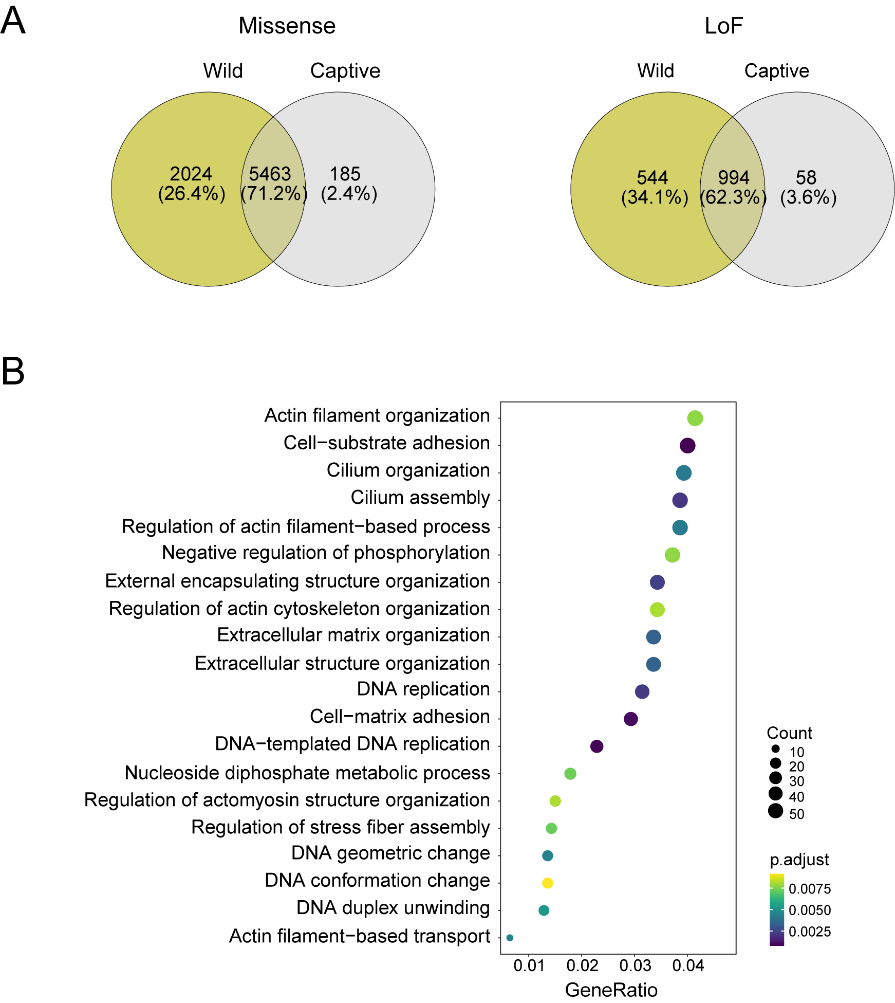
**

**Figure S11. Genes harboring putative missense and LoF mutations.** (A) Shared and uniqe genes in the wild and captive populations with missense and LoF mutations; (B) GO enrichment for the unique genes with LoF mutations in the wild population.


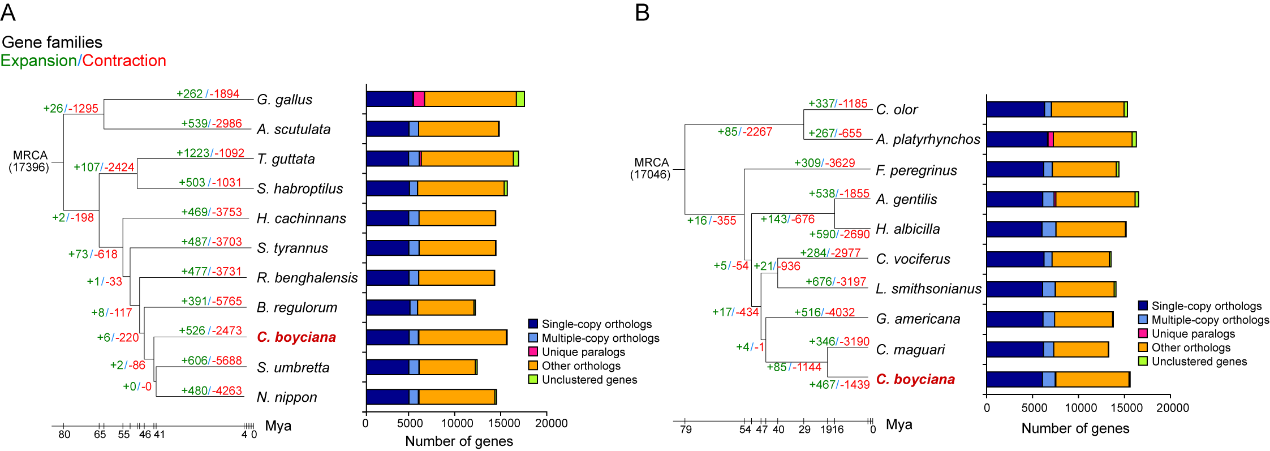


**Figure S12. The expanded and contracted gene families in each bird genome and the comparison of their gene repertoires.** (A) *C. boyciana* and ten birds donnot migrate; (B) Ten migratory birds including *C. boyciana*.

**
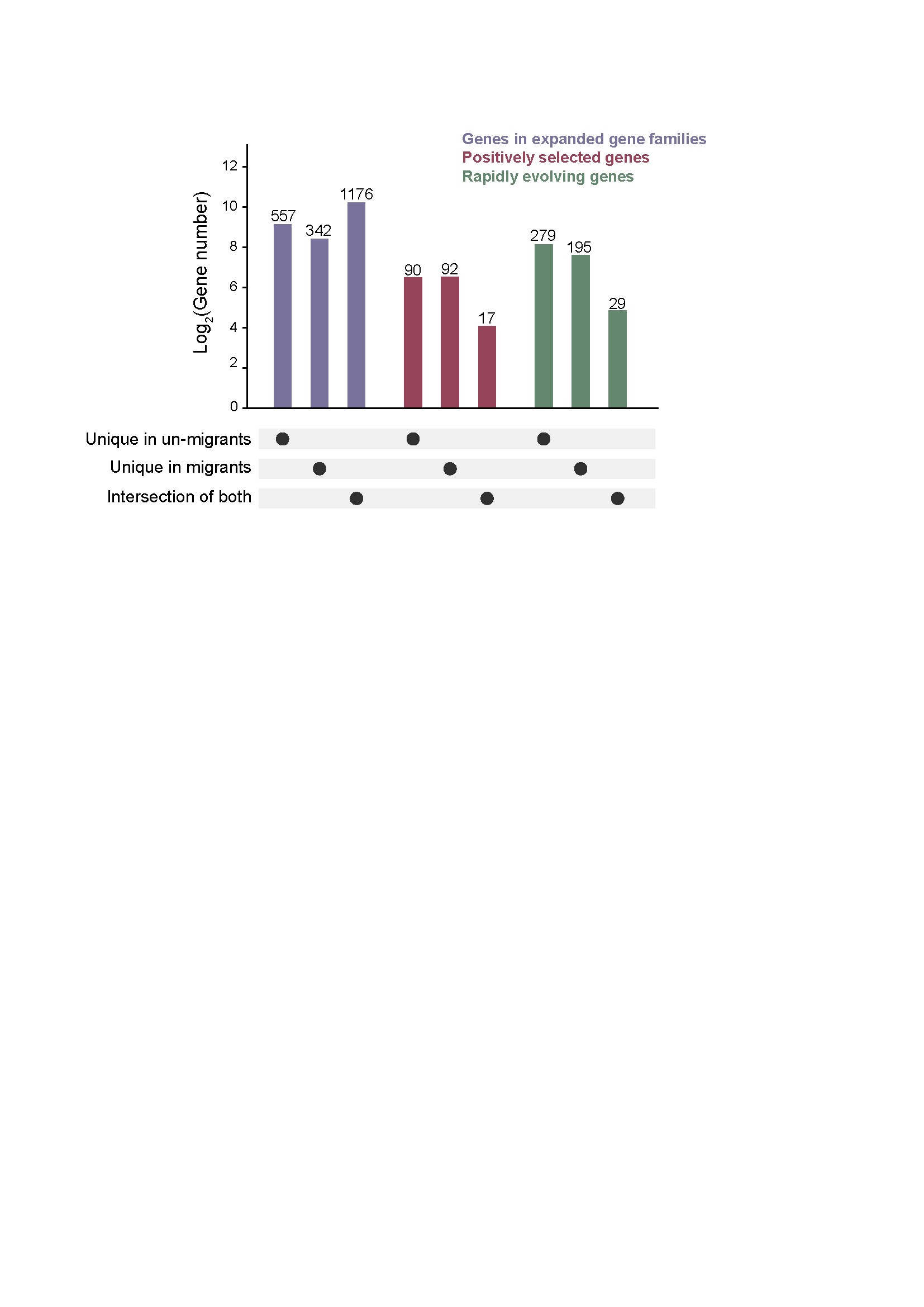
**

**Figure S13. Comparison of detected genomic signals in the oriental stork genome compared with non-migratory birds and migratory birds, including expanded families, positively selected genes and rapidly evolving genes.** Unique in un-migrants: genes detected in the oriental stork only when compared to the uM group; Unique in migrants: genes detected in the oriental stork only when compared to the M group; Intersection of both: genes detected in the oriental stork both when compared to the uM group and M group. uM: non-mgratory; M: migratory.

**
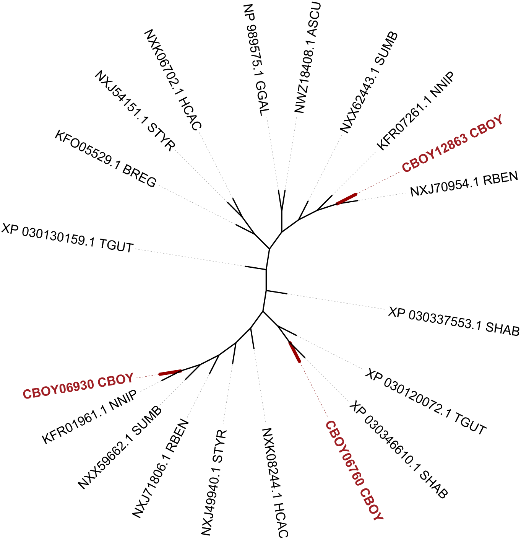
**

**Figure S14. The phylogenetic tree of *CRY2* gene constructed by the maximum likelihood method. The red clade represented three *CRY2* genes in the oriental stork genome.**

**
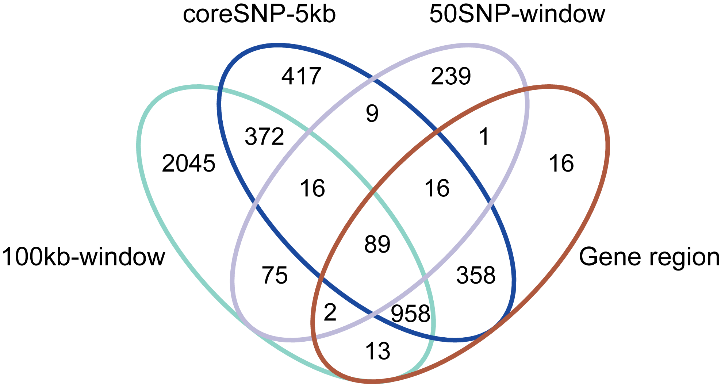
**

**Figure S15. Venn diagram for four methods used to identify genes affected by candidate SNPs under recent selection.**
