# Supplementary material for: Genomic exploration of the endangered oriental stork, Ciconia boyciana, sheds light on migration adaptation and future conservation
Source: Gigascience. 2024 Oct 22;13:giae081. doi: 10.1093/gigascience/giae081 (PMC11494145; doi:10.1093/gigascience/giae081)
Supplement: giae081_Response_to_Reviewer_Comments_Original_Submission [file giae081_response_to_reviewer_comments_original_submission.pdf]

Author's Response To Reviewer Comments

Response to the editor:
Thank you very much for giving us another chance to submit our revised manuscript. We appreciate the valuable comments and suggestions from the two reviewers, which grasped the main points and improved our study so much. We have prepared a point-by-point response as follows and extensively revised our manuscript according to the points reviewers concerned. We hope this revised manuscript could satisfy the reviewers and we are looking forward to your further information.

Reviewer reports:

Reviewer #1: minor

Response to reviewer 1: Thank you very much for the detailed suggestions you proposed. These comments are very important, like introducing the population dynamic of the wild population. We believe our manuscript is clearer after this revision. Thank you.

Question 1. Line 57, I don't think it is a good idea to mention critically endangered spoon-billed sandpiper Calidris pygmaea here, it makes it easy to think that that's a species worth more attention than your bird, Ciconia boyciana.

Response: Thank you for pointing out this issue, we agree. We selected other bird species with less endangered status than the Ciconia boyciana and reorganized the description to highlight the bird in our study. Please see Line 56-58.

Question 2. Line 65, what about the population number before 2018? maybe you can use decades of data to show us how the species is changing, as you mention in Line 71 "such decline".

Response: Yes, you are right, thanks for your suggestion. It is very important to record the wild population size for endangered species conservation. We first discussed the wild population with scientists of Northeast Forestry University who are working on field investigation, but they told us the information of historical population dynamics over the recent decades are very limited. We then searched the literatures for answers:
Oriental storks were widely distributed in Northeast Asia in the history, while in 1868-1935, wild individuals began to decrease[1]. In 1970s, wild populations in Japan and Korea were extinct. And the breeding areas in the Russian Far East, Heilongjiang and Jilin Provinces also became narrow. In 1960s, there were more than 1000 oriental storks breeding in Heilongjiang Province. However, this number decreased to 123 in 1986 and less than 50 in 1990. In late 1980s, there were no storks breeding in Jilin Province[2, 3]. The population size is estimated at 3,000 individuals from 1990s-2010s [4].
However, we did not find continuous yearly records for the overall number of wild oriental storks worldwide. We added more detailed description about the threatened state of oriental stork. Please see Line 68-71.

Question 3. Line 87, the sample? we cannot get the detail information for individual of the reference genome assembly, is it captive-born or rescued wild one? Furth more, the detail information about individuals were listed in Line 167-168, maybe it can be removed to "Samples and ethics statement" part

Response: Thank you, yes, it would be much better if we clarify the sample information used in this study in the Method part. The individual for assembly is a captive-born oriental stork (sample ID: N1170) collected in Harbin North Forest Zoo. In the revised version, we added this information, please see Line 88. And we also moved the description of re-sequenced individuals from Line 167-168 to the "Samples and ethics statement" part, please see Line 93-95.

Question 4. Line 134 Identification of sex-linked regions, I am not sure what is the significance of this part of the analysis? it is interesting to see the phylogeny reconstruction and demographic dynamics, maybe the results (270-276) can be less?

Response: Thank you, we simplified the results of sex-linked regions, please see Line 256-258. Sex chromosomes are relative difficult to be assembled compared to autosomes, because Z and W chromosomes have more complex regions and a large number of repetitive sequences regions. However, accurate identification of sex chromosomes is very important, and many analyses of population re-sequencing need remove the variants on the sex chromosomes, including genetic structure, inbreeding, genetic diversity and demographic dynamics. Some parameters of sex chromosomes are different from autosomes such as selection stress, recombination rate, …. In our practice, especially for large sex chromosomes, SNPs on them would substantially change the genetic structure results. Also, the sex chromosomes could be used in other studies related with sex determination and reproduction. So, this is a very basic but may be valuable genomic resource for other people to cite.
In this study, we identified sex chromosomes and remove these sex-linked regions when carrying out population genomic analysis to avoid bias. As your comment, we simplified the description of the results and moved the figures (previous Fig. 1D-H) to the supplementary materials (Supplementary Fig. S3), please see Line 241-244.

Question 5. Line 295-299, the authors compared the expanded gene families/ PSGs, and REGs in migratory and non-migratory birds, but only numbers listed in the paragraph. Is it possible to find some common feature, e.g. both expanded gene families in migratory but not in non-migratory? maybe it can be more meaningful.

Response: Yes, thank you, we agree. We revised the description and added supplementary figure S13. In fact, we thought exactly the same. What we identified is the genes selected in migratory but not in non-migratory, we just use the oriental stork as a target species.
The expanded gene family/PSGs/REGs identified in the oriental storks when compared to non-migratory birds represent a gene set including migratory-relevant genes (set A) and other unique traits-related (set B) in the oriental stork. Then, we compared the oriental stork with other migratory birds, the potential migratory-relevant genes would not be identified as expanded gene family/PSGs/REGs only in the oriental stork genome, because migratory genes may be shared in the migratory birds, commonly selected in the migratory. So, we used the comparison with other migratory birds to remove the false-positive (set B). The remained genes may represent the genetic mechanisms associated with migration in the oriental stork. Please see Line 343-349 and Supplementary Fig. S13.

Question 6. Line 300. the authors listed and discussed about "collaborative approaches in nervous system and physical energy supply", and listed some other genes inferred may contribute to migration in oriental stork in Fig2B, what about the immune genes? The birds also need to adjust their immune system for migratory.

Response: Yes, thank you, we added. We found several immune genes in the expanded gene families, and we also found some immune related genes that were under positive selection or rapidly evolved, please see Line 370-371. We added them to the Supplementary Table S15-S17, and Fig. 5A.

Question 7. Line 364 In fig4, there are 12 diamond shapes outside the wild circle, however, 16 captive-born individuals from Harbin, and the authors wrote "five captive individuals from Harbin, Japan and USA clustered into the wild population" . The numbers are correct. I am wondering do the authors took these five ones as wild-source or captive-source in the following steps? I have no idea about them.

Response: Thank you, we revised and described more clearly, please see Line 271-273. Here the five captive individuals clustered into the wild population are captive-source, they are all captive-born. The PCA result just implied that their genetic background was very similar to the wild population. In population genomic analysis, we divided the samples into groups according to their origins (wild or captive-born), instead of the genetic background.

Question 8. Line 373, "They seemed to have a high heterozygosity when compared with other endangered

avian species". There still have some critically endangered/endangered/vulnerable species with higher values than Ciconia boyciana, and the readers cannot distinguish the endangered, endangered, vulnerable from the figure, maybe more comment or distinguish method will be clearer.

Response: Yes, thank you, we revised. In this version, we tried to include more threatened bird species and split the CR, EN and VU to distinguish the three threatened categories, please see Fig. 2C and Supplementary Table S13.

Question 9. Line 376, crested ibis, brown eared pheasant, and kakapo, please list the detail numbers.

Response: Thank you, we revised. Because the calculation method for the crested ibis is not a common method in Feng's paper [5], it is difficult to compare the value, so we deleted the crested ibis. For the kakapo, Nicolas et al [6] just give a figure to show the low genetic diversity of the kakapo population, we cannot extract the exact π value, so here we also deleted the kakapo. Finally, we used the genetic diversity of the brown eared pheasant and the green peafowl instead, please see Line 282-284.

Question 10. It is interesting to get the results "captive individuals have complex sources" line 465. Maybe more information about pedigree can be useful, if the zoo have these information.

Response: Thank you. We have discussed with the staff and researchers
in Harbin North Forest Zoo, who were in charge of animal keeping and sample collection. The captive-born individuals increased quickly in recent thirty years, from 1 (in 1975) to 383 (in 2006) (Response Figure 1), due to the success of assist-breeding technology. For the following large generations, there weren't clear pedigree. They told us that till now, they have bred 10-13 generations stem from the wild founders.

Response Figure 1. The number of captive oriental storks in all zoos from 1975 to 2006. (In the uploaded supplementary materials: Response to reviewer comments)

We also searched the literature but failed to find any pedigree records. We just found two origin information of the wild founders. The first reproduction in the Harbin Forest Zoo was in 1991 between two wild individuals from the Sanjiang Plain, Heilongjiang Province (130.12°-135.09°E, 43.83°-48.67°N)[7]. And in 2008, there had another ten wild individuals from Honghe National Nature Reserve (133°37′-133°45′E, 47°43′-47°52′N), Heilongjiang Province [8].

Reviewer #2: The paper describes the first reference genome built for the Oriental stork. The authors have then resequenced storks to asks a number of important questions about the species and its conservation. The Methods are sound and the findings are interesting. However, the paper suffers from a number of shortcomings that together would qualify as major revision. The paper will also need a thorough revision for proper grammar. More specific comments are below.

Response to reviewer 2: Thank you very much for your support. And we have made an extensive revision as your comments and we believe the current manuscript has been improved a lot with your help. We learned a lot from the questions you raised, particularly for the microchromosomes, Ne/Nc and genetic drift. The most importantly, we thank for the suggestion on the whole manuscript organization and the discussion section. We hope this revision could address the questions. We also invited a native speaker to polish the language (Dr. Sunil).

Question 1. The paper often lacks clear links between the specific analyses and, therefore, the questions being addressed
The authors have done a tremendous amount of work on a very thorough analysis. At present, however, the paper reads like a collection of disparate analyses with tenuous links between many of them. For example, how does the gene ontology analysis relate to the demographic modeling and how do these relate to stork conservation?

Response: Thank you very much, this is a very important suggestion which would much improve our manuscript. We reorganized our analysis and made each part more correlated and make this study more like a conservation-oriented study.
Firstly, only with a high-quality chromosome-level reference genome can we conduct a precise assessment on many genetic parameters in conservation genetics. So, the first foundational part was the reference genome assembly.
Secondly, our central part of this manuscript was to assess the genetic status and extinction risk of the oriental stork population, which was measured by population structure, demographic history, genetic diversity, inbreeding level and mutational load. So, these analyses are the second large part that was close related to conservation genomics.
Thirdly, conservation and evolution are closely connected. Migratory birds are under selective pressure from the early life, particularly juveniles need to response to the condition change in the first fall. They need to migrate efficiently to arrive early to the stop-over/wintering sites because the habitat/food resources are limited. Behavior study has proved that learning plays an important role in shaping the migratory route through a lifetime [9]. We thought that it was essential to understand the genomic basis of the migratory adaptation. Because the ecological adaptation is vital for the population survival, which may have evolved for millions of years. Therefore, the investigation on the migratory trait of the oriental stork is the third part in our study.
We reorganized each section and made the manuscript clearer.

Question 2. The paper needs a more fully developed Discussion section

Response: Thank you, we extensively revised the Discussion section and made it more thoughtful and more reasonable, please see Line391-458. Especially, we added the detailed development history of the southeast Sibera and the Bohai Bay, to connect genomic result with ecological information, please see Line427-443.

Question 3. Are all individuals migratory? Or is this species a partial migrants like the American wood stork? See Picardi et al 2020 https://doi.org/10.1002/ecs2.3054

Response: Thank you for this question. The oriental stork, Ciconia boyciana, is a full-migratory avian, assessed by BirdLife International (https://www.iucnredlist.org/species/22697695/131942061) and other researches by GPS/GSM tracking (Response Figure 2) [10, 11]. We didn't find any studies claiming that some oriental storks wintered in their breeding area in northeastern Asia.

Response Figure 2. Individual autumn migration routes and stop-over sites of 18 oriental storks derived from GPS/GSM telemetry devices [10]. (In the uploaded supplementary materials: Response to reviewer comments)

Question 4. The authors argue that drift is weak in this large single population of storks. What is the Ne/Nc ratio? Does that support their argument?

Response: Thank you. We redo the related analysis and added more analysis. The Ne of the wild populations has been about 1,000 for the past decades (Fig. 4C). And in recent twenty years, the census size (Nc) was 3,000. The Ne/Nc ratio is 0.33, which fell within the range of most species [12], please see Line 329-330.
We also calculated the folded SFS of derived alleles, which showed a "L-shaped" in the wild and captive populations (Fig. 3B). This result also proved that singletons were abundant in the population, and so genetic drift seems not concerning [13]? But we noticed that low-frequency alleles were deficient while the medium-frequency was excessive in the captive population compared to the wild population. Thus, the genetic drift seemed to be slightly stronger in the captivity, please see Line 311-316. This point can also be reflected by the dispersal PCA points while a unified ancestral component in the Admixture plot of captive individuals.

Question 5. Lines 443-445- what gaps will your genome fill that will allow for targeted recovery plans?

Response: Thank you for this question. Conservation efforts for the threatened species can be enhanced by a high-quality reference genome. We can obtain a more precise and more detailed measures for many key genetic factors in conservation genomics [14]. For example, the evaluation of inbreeding by measuring runs of homozygosity (ROH) highly depends on the high-quality reference genome with outstanding contiguity. This is due to the ROH in small populations with high-level inbreeding often spans over several millions of base pairs [15-17], and hardly be detected based on fragmented genomes assembled from short reads. Additionally, the genetic diversity(π) could also be promoted by a higher-quality reference genome, and we could often detect more variants across the genome based on the Long-read assembled genome than the Short-read assembled genome, because long reads could 1) span much more complex genomic regions[14] and 2) generate much longer contigs than short reads[18, 19]. Many genomic regions that cannot be assembled from short reads can be assembled from long reads, and these regions may contain important variants. Additionally, longer contigs facilitate a higher number of reads aligning accurately to the reference genome. Both of the two aspects could contribute to enhancing the accuracy of genetic diversity calculations. As we mentioned in Q4 of reviewer 1, the high-quality genome could be also well used to the identification of sex chromosomes [20]. As for our genome, we comprehensively assessed the genetic diversity and inbreeding, revealing a relatively positive genetic status with high heterozygosity, low inbreeding, and recent population decline. We found two captive-born oriental storks with higher inbreeding levels (~30%) than others (<10%). And recent inbreeding (reflected by long ROHs, > 1 Mb) was significantly more serious in captive-born oriental storks than the wild (Fig. 2D). These findings provided an alarming that captive-breeding programs should be more cautious on the pedigree management.

Question 6. The authors may wish to cite Flamio, R, Jr & Ramstad, KM. 2023. Chromosome-level genome of the wood stork (Mycteria americana) provides insight into avian chromosome evolution. Journal of Heredity, doi:10.1093/jhered/esad077. How does your genome compare? And how did you treat microchromosomes?

Response: Thank you for this question. We checked the Hi-C heatmap, fortunately all chromosomes were successfully assembled including 23 microchromosomes (Response Figure 3). Perhaps due to the high sequencing depth of Hi-C data (~98.20X), we didn't observe obvious blank gaps within large scaffolds that could be anchored onto chromosomes (Response Figure 4). There were still some small scaffolds having limited contact with other chromosomes, we just leave them (0.68% of the total length of the assembled scaffolds) as unanchored genomic fragments.
We didn't pay special attention to microchromosomes because this study did not discuss the avian chromosome evolution. We applied the same methods to the microchromosomes as the macrochromosomes in each analysis. But we thought that the microchromosomes deserved more detailed description on results and discussion, we referred to this the wood stork study and added some sentences, please see Line 401-406.

Response Figure 3. Heatmap of Hi-C chromosomal interaction density among all 35 chromosomes (33 autosomes + ZW, 2n=68). (In the uploaded supplementary materials: Response to reviewer comments)

Response Figure 4. Heatmap of Hi-C chromosomal interaction density among 23 microchromosomes.

Additional comments:
Question 7. Please provide additional information on the captive storks sampled. How many generations of captive breeding did they stem from?

Response: Thank you. We have discussed with the staff and researchers in the Harbin North Forest Zoo, who were in charge of animal keeping and sample collection. They told us that the captive-born oriental storks were 10-13 generations stem from the wild founders. We added the information of captive samples in supplementary table S11.

Question 8. Lines 86 & 89 - what does 'rescued' mean in this context?

Response: Thank you, we revised the description here and added the detailed rescue situation. The three wild samples from Harbin North Forest Zoo (Line 87), were successfully rescued from the wild and now live in the veterinary hospital of the zoo. The 26 wild individuals in Line89 were found to be ill/starving/dying when they were rescued in Bohai Bay in migratory season of 2022, and fail to be rescued and were dead then, so we could collect the muscle samples after death. We added the rescue information in supplementary table S11.

Question 9. Clarify if the selection analysis was to identify loci under selection in Oriental storks, storks more broadly, or migratory birds. Lines 156-158 could be expanded and the text throughout modified to be very specific in this regard.

Response: Thank you, we revised. The comparative genomic analysis was to identify loci under positive selection in oriental stork. We redescribed these sentences to make it more readable and clearer.

Question 10. Reduce the number of or simplify the figures where possible

Response: Thank you, we revised and simplified the Figures 1, 3, 4 and 5. Other subfigures were moved to supplementary materials.

Question 11. Lines 242-247 - this text should be moved to the Methods section

Response: Thank you, we polished these sentences. We maintained the important assembly results here and moved other description to the Methods section.

Question 12. Table 1 caption - change 'of this' to 'the genome'

Response: Corrected, thank you.

Question 13. Figure 4A - what does this analysis look like if you include only the wild individuals?

Response: Thank you, we tested. We just used the 29 individuals from the wild to conduct the PCA analysis, please see Response Figure 3 (from left to right: PC1PC2, PC1PC3, and PC2PC3). We didn't observe a population structure within the wild individuals, all wild individuals were scattered in the PCA plot. In fact, we think this is consistent with the actual population survey, because there is just one Eastern Asia population of wild oriental stork, and they share the same breeding areas and migratory routes [21].

Response Figure 5. The PCA analysis of the 29 wild oriental storks. (In the uploaded supplementary materials: Response to reviewer comments)

--
Please also take a moment to check our website at https://www.editorialmanager.com/giga/l.asp?i=154222&l=K4I56MXD for any additional comments that were saved as attachments. Please note that as GigaScience has a policy of open peer review, you will be able to see the names of the reviewers.

In compliance with data protection regulations, you may request that we remove your personal registration

details at any time. (Use the following URL: https://www.editorialmanager.com/giga/login.asp?a=r). Please contact the publication office if you have any questions.

1. Ma X. Research on captive oriental white stork (Ciconia boyciana) in energy of digestion and fledgling growth. Northeast Forestry University, 2007.
2. Liu Z and Li X. The research progress of oriental white stork. Territory & Natural Resources Study. 2008; 01:77-8. doi:10.16202/j.cnki.tnrs.2008.01.001.
3. Zeng S, Cheng L and Li X. The numerical distribution and conservation of oriental white stork in China. Territory & Natural Resources Study. 2003; 01:71-2. doi:10.16202/j.cnki.tnrs.2003.01.035.
4. International B. The IUCN Red List of Threatened Species 2018: e.T22697695A131942061. 2018; doi:https://dx.doi.org/10.2305/IUCN.UK.2018-2.RLTS.T22697695A131942061.en.
5. Feng S, Fang Q, Barnett R, Li C, Han S, Kuhlwilm M, et al. The Genomic Footprints of the Fall and Recovery of the Crested Ibis. Current Biology. 2019;29:340-9. doi:10.1016/j.cub.2018.12.008.
6. Dussex N, van der Valk T, Morales HE, Wheat CW, Díez-del-Molino D, von Seth J, et al. Population genomics of the critically endangered kākāpō. Cell Genomics. 2021;1 doi:10.1016/j.xgen.2021.100002.
7. LI L, Wei H and Gao Z. Preliminary observation on the reproduction ecology of captive oriental white stork. Chinese Journal of Wildlife. 1996; 06:14-7. doi:10.19711/j.cnki.issn2310-1490.1996.06.004.
8. Zan S, Zhou L, Jiang H, Zhang B, Wu Z and Hou Y. Genetic structure of the oriental white stork (Ciconia boyciana): implications for a breeding colony in a non-breeding area. Integrative zoology. 2008;3 3:235-44. doi:10.1111/j.1749-4877.2008.00096.x.
9. Aikens EO, Nourani E, Fiedler W, Wikelski M and Flack A. Learning shapes the development of migratory behavior. Proceedings of the National Academy of Sciences. 2024;121 12:e2306389121. doi:doi:10.1073/pnas.2306389121.
10. Fan S, Zhao Q, Li H, Zhu B, Dong S, Xie Y, et al. Cyclical helping hands: seasonal tailwinds differentially affect migrating Oriental Storks (Ciconia boyciana) travel speed. Avian Research. 2020;11 1:10. doi:10.1186/s40657-020-00196-8.
11. Yang Z, Chen L, Jia R, Xu H, Wang Y, Wei X, et al. Migration routes of the endangered Oriental Stork (Ciconia boyciana) from Xingkai Lake, China, and their repeatability as revealed by GPS tracking. Avian Research. 2023;14 2:100090. doi:10.1016/j.avrs.2023.100090.
12. Hare MP, Nunney L, Schwartz MK, Ruzzante DE, Burford M, Waples RS, et al. Understanding and estimating effective population size for practical application in marine species management. Conservation biology. 2011;25 3:438-49. doi:10.1111/j.1523-1739.2010.01637.x.
13. Wang P, Burley JT, Liu Y, Chang J, Chen, Lu Q, et al. Genomic Consequences of Long-Term Population Decline in Brown Eared Pheasant. Molecular Biology and Evolution. 2021;38 1:263-73. doi:10.1093/molbev/msaa213.
14. Formenti G, Theissinger K, Fernandes C, Bista I, Bombarely A, Bleidorn C, et al. The era of reference genomes in conservation genomics. Trends in ecology & evolution. 2022;37 3:197-202. doi:10.1016/j.tree.2021.11.008.
15. Saremi NF, Supple MA, Byrne A, Cahill JA, Coutinho LL, Dalen L, et al. Puma genomes from North and South America provide insights into the genomic consequences of inbreeding. Nature communications. 2019;10 1:4769. doi:10.1038/s41467-019-12741-1.
16. Xie HX, Liang XX, Chen ZQ, Li WM, Mi CR, Li M, et al. Ancient Demographics Determine the Effectiveness of Genetic Purging in Endangered Lizards. Molecular biology and evolution. 2022;39 1 doi:10.1093/molbev/msab359.
17. Dussex N, Van Der Valk T, Morales HE, Wheat CW, Díez-del-Molino D, Von Seth J, et al. Population genomics of the critically endangered kākāpō. Cell Genomics. 2021;1 1.
18. Zhang L, Lan T, Lin C, Fu W, Yuan Y, Lin K, et al. Chromosome-scale genomes reveal genomic consequences of inbreeding in the South China tiger: A comparative study with the Amur tiger. Molecular ecology resources. 2022; doi:10.1111/1755-0998.13669.
19. Yang S, Lan T, Zhang Y, Wang Q, Li H, Dussex N, et al. Genomic investigation of the Chinese alligator reveals wild-extinct genetic diversity and genomic consequences of their continuous decline. Molecular ecology resources. 2022; doi:10.1111/1755-0998.13702.
20. Paez S, Kraus RHS, Shapiro B, Gilbert MTP, Jarvis ED, Group VGPC, et al. Reference genomes for conservation. 2022;377 6604:364-6. doi:doi:10.1126/science.abm8127.

21. Ga R. The distribution and population size and trend of Oriential Stork (Ciconia boyciana) and the habitat selection of the overwintering group in Bohai Bay. Master, Inner Mongolia Normal University, 2021.
