# Supplementary material for: Genomic exploration of the endangered oriental stork, Ciconia boyciana, sheds light on migration adaptation and future conservation
Source: Gigascience. 2024 Oct 22;13:giae081. doi: 10.1093/gigascience/giae081 (PMC11494145; doi:10.1093/gigascience/giae081)
Supplement: giae081_Response_to_Reviewer_Comments_Revision_1 [file giae081_response_to_reviewer_comments_revision_1.pdf]

Author's Response To Reviewer Comments

Reviewer reports:
Reviewer #1: The revised manuscript has done a lot of effort to make it better and clearer now. I agree with the second reviewer "The article has a large number of data lists, which is a common problem in genomics articles these days." However, we can see the author's efforts in this article, they compared the wild and the captive populations of Ciconia boyciana, also compared this bird with other birds in danger.

Thank you very much for your comments, and our manuscript has been much improved after this revision according to your suggestions, as well as comments from the reviewer 2. We hope this manuscript could provide useful genomic resources and insights for the future conservation of this endangered species. In this round of revision, we also comprehensively revised this manuscript as your comments and make a point-to-point response for your continence to trace the revision. We are looking forward to seeing your next round of comments, thank you very much.

Here are some additional comments,

Q1 avoid to use discuss results in terms of subjective statements through the MS, e.g. Abstract: We didn't find a genetic structure in the wild population. Result: We identified 10.41% of genome sequences as repetitive elements

Response: Thank you for pointing out this question, we have rephrased all sentences involved in this issue throughout the manuscript. All the places we revised has been highlighted in the track-change version.

Q2 Please list and discuss about some examples about protection and rescue of endanger species with the help of genomic analysis, which can make the meaning of this job more significant.

Response: Thank you, we agree. Genomic assessment is of great importance in the protection of endangered species. In the revised manuscript, we added a small paragraph in the introduction part to introduce the representative examples of how the genomics have been applied in the conservation efforts of endangered animals. Please see Line 74-83.

Q3 Line 282 The nucleotide diversity (π) of the wild and the captive populations, still, we didn't know the detail number for Ciconia boyciana.

Response: Thank you, we revised. This time we added the detailed number of the nucleotide diversity of Ciconia boyciana populations to make this comparison more reasonable and clearer. Please see Line 285.

Q4 Line428-430, The Discussion section, might be relevant to state the reason for the extinction of oriental storks in Japan and Korea?

Response: Yes, thank you for the advice. We searched some literatures and related the extinction of this bird species in Japan and Korea last century to the climate change and human activity, especially to the fast development of this two areas on the economy and industrialization. Please see Line 445-451.
