# Supplementary material for: Genomic exploration of the endangered oriental stork, Ciconia boyciana, sheds light on migration adaptation and future conservation
Source: Gigascience. 2024 Oct 22;13:giae081. doi: 10.1093/gigascience/giae081 (PMC11494145; doi:10.1093/gigascience/giae081)
Supplement: giae081_Reviewer_1_Report_Original_Submission — Ke He -- 1/22/2024 Reviewed [file giae081_reviewer_1_report_original_submission.pdf]

Reviewer Report

Title: Genomic exploration of the endangered oriental stork, Ciconia boyciana, shed lights on migration adaptation and future conservation

Version: Original Submission **Date:** 1/22/2024

Reviewer name: Ke He

Reviewer Comments to Author:

minor
1 Line 57, I don't think it is a good idea to mention critically endangered spoon-billed sandpiper Calidris pygmaea here, it makes it easy to think that that's a species worth more attention than your bird, Ciconia boyciana.
2 Line 65, what about the population number before 2018? maybe you can use decades of data to show us how the species is changing, as you mention in Line 71 "such decline".
3 Line 87, the sample? we cannot get the detail information for individual of the reference genome assembly, is it captive-born or rescued wild one? Furth more, the detail information about individuals were listed in Line 167-168, maybe it can be removed to "Samples and ethics statement" part.
4 Line 134 Identification of sex-linked regionsï¼ŒI am not sure what is the significance of this part of the analysis? it is interesting to see the phylogeny reconstruction and demographic dynamics, maybe the results (270-276) can be less?
5 Line 295-299, the authors compared the expanded gene families/ PSGs, and REGs in migratory and non-migratory birds, but only numbers listed in the paragraph. Is it possible to find some common feature, e.g. both expanded gene families in migratory but not in non-migratory? maybe it can be more meaningful.
6 Line 300. the authors listed and discussed about "collaborative approaches in nervous system and physical energy supply", and listed some other genes inferred may contribute to migration in oriental stork in Fig2B, what about the immune genes? The birds also need to adjust their immune system for migratory.
7 Line 364 In fig4, there are 12 diamond shapes outside the wild circle, however, 16 captive-born individuals from Harbin, and the authors wrote "five captive individuals from Harbin, Japan and USA clustered into the wild population" . The numbers are correct. I am wondering do the authors took these five ones as wild-source or captive-source in the following steps? I have no idea about them.
8 Line 373, "They seemed to have a high heterozygosity when compared with other endangered avian species". There still have some critically endangered/endangered/vulnerable species with higher values than Ciconia boyciana, and the readers cannot distinguish the endangered, endangered, vulnerable from the figure, maybe more comment or distinguish method will be clearer.
9 Line 376, crested ibis, brown eared pheasant, and kakapo, please list the detail numbers.
10 It is interesting to get the results "captive individuals have complex sources" line 465. Maybe more information about pedigree can be useful, if the zoo have these information.

Methods

Are the methods appropriate to the aims of the study, are they well described, and are necessary controls included? Choose an item.

Conclusions

Are the conclusions adequately supported by the data shown? Choose an item.

Reporting Standards

Does the manuscript adhere to the journal's guidelines on minimum standards of reporting? Choose an item.

Choose an item.

Statistics

Are you able to assess all statistics in the manuscript, including the appropriateness of statistical tests used? Choose an item.

Quality of Written English

Please indicate the quality of language in the manuscript: Choose an item.

Declaration of Competing Interests

Please complete a declaration of competing interests, considering the following questions:

- Have you in the past five years received reimbursements, fees, funding, or salary from an organisation that may in any way gain or lose financially from the publication of this manuscript, either now or in the future?
- Do you hold any stocks or shares in an organisation that may in any way gain or lose financially from the publication of this manuscript, either now or in the future?
- Do you hold or are you currently applying for any patents relating to the content of the manuscript?
- Have you received reimbursements, fees, funding, or salary from an organization that holds or has applied for patents relating to the content of the manuscript?
- Do you have any other financial competing interests?
- Do you have any non-financial competing interests in relation to this paper?

If you can answer no to all of the above, write 'I declare that I have no competing interests' below. If your reply is yes to any, please give details below.

I declare that I have no competing interests.

I agree to the open peer review policy of the journal. I understand that my name will be included on my report to the authors and, if the manuscript is accepted for publication, my named report including any attachments I upload will be posted on the website along with the authors' responses. I agree for my report to be made available under an Open Access Creative Commons CC-BY license (http://creativecommons.org/licenses/by/4.0/). I understand that any comments which I do not wish to be included in my named report can be included as confidential comments to the editors, which will not be published.

Choose an item.

To further support our reviewers, we have joined with Publons, where you can gain additional credit to further highlight your hard work (see: https://publons.com/journal/530/gigascience). On publication of this paper, your review will be automatically added to Publons, you can then choose whether or not to claim your Publons credit. I understand this statement.

Yes Choose an item.
