# Supplementary material for: Genomic exploration of the endangered oriental stork, Ciconia boyciana, sheds light on migration adaptation and future conservation
Source: Gigascience. 2024 Oct 22;13:giae081. doi: 10.1093/gigascience/giae081 (PMC11494145; doi:10.1093/gigascience/giae081)
Supplement: giae081_Reviewer_2_Report_Original_Submission — Kristina M. Ramstad -- 1/23/2024 Reviewed [file giae081_reviewer_2_report_original_submission.pdf]

Reviewer Report

Title: Genomic exploration of the endangered oriental stork, Ciconia boyciana, shed lights on migration adaptation and future conservation

Version: Original Submission **Date:** 1/23/2024

Reviewer name: Kristina M. Ramstad

Reviewer Comments to Author:

The paper describes the first reference genome built for the Oriental stork. The authors have then resequenced storks to asks a number of important questions about the species and its conservation. The Methods are sound and the findings are interesting. However, the paper suffers from a number of shortcomings that together would qualify as major revision. The paper will also need a thorough revision for proper grammar. More specific comments are below.

1. The paper often lacks clear links between the specific analyses and, therefore, the questions being addressed

The authors have done a tremendous amount of work on a very thorough analysis. At present, however, the paper reads like a collection of disparate analyses with tenuous links between many of them. For example, how does the gene ontology analysis relate to the demographic modeling and how do these relate to stork conservation?

2. The paper needs a more fully developed Discussion section

Are all individuals migratory? Or is this species a partial migrants like the American wood stork? See Picardi et al 2020 https://doi.org/10.1002/ecs2.3054

The authors argue that drift is weak in this large single population of storks. What is the Ne/Nc ratio? Does that support their argument?

Lines 443-445- what gaps will your genome fill that will allow for targeted recovery plans?

The authors may wish to cite Flamio, R, Jr & Ramstad, KM. 2023. Chromosome-level genome of the wood stork (Mycteria americana) provides insight into avian chromosome evolution. Journal of Heredity, doi:10.1093/jhered/esad077. How does your genome compare? And how did you treat microchromosomes?

Additional comments:

* Please provide additional information on the captive storks sampled. How many generations of captive breeding did they stem from?
* Lines 86 & 89 - what does 'rescued' mean in this context?
* Clarify if the selection analysis was to identify loci under selection in Oriental storks, storks more broadly, or migratory birds. Lines 156-158 could be expanded and the text throughout modified to be very specific in this regard.
* Reduce the number of or simplify the figures where possible
* Lines 242-247 - this text should be moved to the Methods section
* Table 1 caption - change 'of this' to 'the genome'

* Figure 4A - what does this analysis look like if you include only the wild individuals?

Methods

Are the methods appropriate to the aims of the study, are they well described, and are necessary controls included? Choose an item.

Conclusions

Are the conclusions adequately supported by the data shown? Choose an item.

Reporting Standards

Does the manuscript adhere to the journal's guidelines on [minimum standards of reporting?](minimum standards of reporting) Choose an item.

Choose an item.

Statistics

Are you able to assess all statistics in the manuscript, including the appropriateness of statistical tests used? Choose an item.

Quality of Written English

Please indicate the quality of language in the manuscript: Choose an item.

Declaration of Competing Interests

Please complete a declaration of competing interests, considering the following questions:

- Have you in the past five years received reimbursements, fees, funding, or salary from an organisation that may in any way gain or lose financially from the publication of this manuscript, either now or in the future?
- Do you hold any stocks or shares in an organisation that may in any way gain or lose financially from the publication of this manuscript, either now or in the future?
- Do you hold or are you currently applying for any patents relating to the content of the manuscript?
- Have you received reimbursements, fees, funding, or salary from an organization that holds or has applied for patents relating to the content of the manuscript?
- Do you have any other financial competing interests?
- Do you have any non-financial competing interests in relation to this paper?

If you can answer no to all of the above, write 'I declare that I have no competing interests' below. If your reply is yes to any, please give details below.

I declare that I have no competing interest.

I agree to the open peer review policy of the journal. I understand that my name will be included on my report to the authors and, if the manuscript is accepted for publication, my named report including any attachments I upload will be posted on the website along with the authors' responses. I agree for my report to be made available under an Open Access Creative Commons CC-BY license (http://creativecommons.org/licenses/by/4.0/). I understand that any comments which I do not wish to be included in my named report can be included as confidential comments to the editors, which will not be published.

Choose an item.

To further support our reviewers, we have joined with Publons, where you can gain additional credit to further highlight your hard work (see: https://publons.com/journal/530/gigascience). On publication of this paper, your review will be automatically added to Publons, you can then choose whether or not to claim your Publons credit. I understand this statement.

Yes Choose an item.
